# Estimation of individual cumulative ultraviolet exposure using a geographically-adjusted, openly-accessible tool

**DOI:** 10.1186/s12895-016-0038-1

**Published:** 2016-01-20

**Authors:** Gefei A. Zhu, Inbar Raber, Sukolsak Sakshuwong, Shufeng Li, Angela S. Li, Caroline Tan, Anne Lynn S. Chang

**Affiliations:** Department of Dermatology, Stanford University School of Medicine, 450 Broadway St., Redwood City, CA 94063 USA; Department of Computer Science, 353 Serra Mall, Stanford, CA 94305 USA

**Keywords:** Clinical research, Survey, Case–control, Sun exposure, Skin cancer, Ultraviolet radiation

## Abstract

**Background:**

Estimates of an individual’s cumulative ultraviolet (UV) radiation exposure can be useful since ultraviolet radiation exposure increases skin cancer risk, but a comprehensive tool that is practical for use in the clinic does not currently exist.

The objective of this study is to develop a geographically-adjusted tool to systematically estimate an individual’s self-reported cumulative UV radiation exposure, investigate the association of these estimates with skin cancer diagnosis, and assess test reliability.

**Methods:**

A 12-item online questionnaire from validated survey items for UV exposure and skin cancer was administered to online volunteers across the United States and results cross-referenced with UV radiation indices. Cumulative UV exposure scores (CUES) were calculated and correlated with personal history of skin cancer in a case–control design. Reliability was assessed in a separate convenience sample.

**Results:**

1,118 responses were included in the overall sample; the mean age of respondents was 46 (standard deviation 15, range 18 – 81) and 150 (13 %) reported a history of skin cancer. In bivariate analysis of 1:2 age-matched cases (*n* = 149) and controls (*n* = 298), skin cancer cases were associated with (1) greater CUES prior to first skin cancer diagnosis than controls without skin cancer history (242,074 vs. 205,379, *p* = 0.003) and (2) less engagement in UV protective behaviors (*p* < 0.01). In a multivariate analysis of age-matched data, individuals with CUES in the lowest quartile were less likely to develop skin cancer compared to those in the highest quartile. In reliability testing among 19 volunteers, the 2-week intra-class correlation coefficient for CUES was 0.94. We have provided the programming code for this tool as well as the tool itself via open access.

**Conclusions:**

CUES is a useable and comprehensive tool to better estimate lifetime ultraviolet exposure, so that individuals with higher levels of exposure may be identified for counseling on photo-protective measures.

**Electronic supplementary material:**

The online version of this article (doi:10.1186/s12895-016-0038-1) contains supplementary material, which is available to authorized users.

## Background

Ultraviolet (UV) radiation exposure increases risk for cutaneous cancers and photo-aging, [1–7] however, systematic estimation of whether an individual has higher-than-average sun exposure is difficult in the clinical setting. UV radiation is an environmental carcinogen, much like cigarette smoke is an environmental carcinogen whose exposure can be estimated using lifetime cumulative “pack-years” in the clinical setting [8]. The objective of this study was to create a tool that systematically estimates cumulative UV exposures, and accounts for geographic locale.

Calculating an individual’s lifetime UV radiation exposure can be challenging [9] in the clinical setting, as it requires information on (1) the frequency and duration of UV exposures which can vary over a person’s lifetime, and (2) the residential history, since UV index varies by geographic location. In the research setting, data on (1) has been gathered through face-to-face [3, 5, 6, 10–15] or telephone interviews, [16, 17] which can take 45 min just to collect sun exposure-related variables, [13] and is too time-consuming for a fast paced dermatology clinic. Individual diary formats and dosimetry are suitable for collecting data for short periods of time, but not feasible for recording lifetime exposures [18–21] over decades. Finally, though shorter instruments exist, such as the Sun Exposure Behavior Inventory, [22] these questionnaires do not collect an complete residential and exposure history to allow for assessment of lifetime ultraviolet exposure.

With regard to data collection and analysis, an accurate, clinically-useful tool should rapidly parse residential histories, including the durations of residence, and link them to UV indices, as all these factors can vary significantly across a lifetime [20, 22, 23]. Individuals living in the United States frequently change their geographic region of residence over their lifetimes, with over one-third of the population residing in a state different than the one they were born in [24, 25]. Therefore, accounting for regional differences in UV index when estimating an individual’s lifetime UV radiation exposure is critical. Indeed, the average annual UV index varies widely within the United States, from 1.9 in Anchorage, Alaska, to 3.3 in Seattle, Washington to 9.3 in Honolulu, Hawaii and must be accounted for when assessing cumulative exposure (Additional file 1: Table S1).

In this three-part study, we developed an internet-based, self-administered questionnaire for (1) estimating cumulative UV exposure (which we term the cumulative UV exposure score or CUES) and (2) assessed for associations with sun protective behaviors and skin cancer to establish a connection with clinically relevant endpoints, and (3) since sun protective behaviors are known to change after skin cancer diagnosis, [19, 26–28] we retrospectively assessed the CUES before and after first skin cancer diagnosis to examine whether CUES could detect a significant difference, as would be expected from data in the medical literature.

## Methods

### Recruitment of participants

Following approval by the Stanford University Institutional Review Board (Protocol #29695), volunteers for this case–control study were recruited online between March and November 2014 to ensure geographic diversity in residence and travel throughout the United States. Volunteers were contacted using ResearchMatch, a national health volunteer registry created by several academic institutions and supported by the United States National Institutes of Health as part of the Clinical Translational Science Award (CTSA) program. ResearchMatch curates a database of volunteers who have consented to be contacted by researchers about health-related studies for which they may be eligible.

Individuals aged 18 years or older who have not resided outside of the United States for more than one year in their lifetime were eligible for the study. Those residing outside the United States for greater than one year were excluded, as UV index data from countries outside the United States were not always directly comparable due to differences in measurement methodology. All eligible subjects within the 50 states were sent an email with an invitation to complete the study questionnaire. The random selection feature was utilized in the recruitment process.

ResearchMatch did not allow us to confirm whether recruitment emails were received, opened, or read by recipients, precluding calculation of a traditional response rate. In addition, we were unable to access the demographics of individuals who did not participate in the study to probe for potential bias of responders versus non-responders. Subjects were not compensated for their participation in this study.

Study cases were defined as those with a personal history of skin cancer diagnosed after age 18, and controls were defined as those without a personal history of skin cancer. Because initial testing indicated that CUES is strongly correlated with age, cases and controls were matched on age. Specifically, age at first skin cancer diagnosis among cases was matched with age at time of survey administration among controls. For each case, two age-matched controls were randomly selected from the study cohort using standard propensity score matching.

To test the reliability of the questionnaire responses, a convenience sample of 19 individuals who were 18 years and older from the Stanford academic community, and separate from ResearchMatch participants, was asked to complete the questionnaire twice with an intervening two-week period between administrations, with results compared between the two time points. The sample size of 19 and the two-week time separation were determined after literature review of other questionnaire reliability studies. Since there are no published reports of *minimum* time interval and sample size requirements specifically related to ultraviolet radiation exposure surveys, we utilized test-retest time interval and sample size reported in similar types of studies consisting of self-reported data outside of dermatology. The two-week time interval between test and retest for reliability studies is a generally accepted time frame referenced in multiple clinical studies [29–32]. For sample size determination for test-retest reliability studies, a well-designed study by Hobart and colleagues [33] have reported similar intra-class correlation coefficients across a sample size of 20 versus 120 in a study of self-reported patient symptoms.

### Questionnaire development and administration

Following a PubMed search and review of validated sun-exposure and skin cancer questionnaire items, a survey was created consisting of 17 total items. Out of the 17 questions, the first eleven items were used to ascertain clinically relevant phenomena such as history of skin cancer or sun protective behaviors for the purposes of this study.

In actual non-study use, we envision that only items under the heading “Estimated outdoor exposure time” and “Estimated location data” would need to be answered to calculate CUES (although the first eleven items could be clinically useful for the health care provider, they are not needed to calculate CUES). Skip patterns were automatically implemented and the actual number of questions answered could be as few as 10, depending on the responses entered. The questionnaire was administered using a dynamic website interface (see screenshots included in the Additional file 2 and live online demo at http://gefeizhu.github.io/cues-study/). Demographic information collected from respondents was limited to age and self-reported Fitzpatrick skin type, based on questions on propensity for tanning and burning [34, 35]. Next, respondents were asked about whether there was a personal or family history of skin cancer, and if so, what type of cancer (possible choices were “basal cell carcinoma,” “squamous cell carcinoma,” “melanoma,” “other,” or “don’t know”). Respondents with a personal history of skin cancer were asked about age and type of skin cancer at first diagnosis. All respondents answered questions on lifetime frequency of (1) tanning bed use (possible choices were “0,” “1 – 5,” “6 – 10,” “11 – 100, “and “greater than 100”), (2) blistering sunburns (possible choices were “0,” “1 – 5,” “6 – 10,” and “greater than 10”), (3) use of sunscreen, (4) use of long-sleeved shirts, (5) use of a hat, and (6) shade-seeking behaviors (possible choices for items 3 – 6 were “never,” “rarely,” sometimes,” “often,” and “always”); some of these items were adapted from previously validated instruments [16, 22] and modified for use in our questionnaire. For individuals with a personal history of skin cancer, the 6 items mentioned above appeared twice to capture behaviors both before and after the date of diagnosis of their first skin cancer. To minimize recall bias, identical before and after question pairs related to sun protective behaviors and skin blistering were separated by four questions not related to the question pairs.

Respondents were asked about duration of sun exposure during peak sunlight hours (10 am – 4 pm) during weekdays and weekends, expressed in hours per day. To facilitate recall, exposure duration data were collected for discrete age ranges (0 – 13, 13 – 20, 20 – 40, 40 – 65, 65 – 80, and 80+) that roughly correspond with life milestones.[10, 31, 36] To account for geographic differences in UV exposure across the lifetime, respondents were queried on location of residence and the ages during which they lived in each location. The questionnaire was programmed to require a full and complete lifetime residential history before respondents were allowed to submit the responses.

The final item of the online survey tool solicited subjective feedback from respondents in narrative form.

Integration of data validation tools into our online questionnaire allowed for real-time delivery of feedback to respondents, minimized unanalyzable and missing data (99.8 % of all responses were included for analysis, see Results section) and ensured proper implementation of inclusion and exclusion criteria.

### Calculation of Cumulative UV Exposure Score (CUES)

CUES was calculated from data taken in the sections of the questionnaire entitled “Estimated outdoor exposure time” and “Estimated location data”. The most recent data available from the National Oceanic and Atmospheric Administration (2009–2012) at the time of questionnaire development was used to calculate average annual UV indices for 58 anchor cities across the United States (Additional file 1: Table S1). During this period, the lowest UV index was 1.9 (Anchorage, AK) and the highest UV index was 10.3 (San Juan, PR). The UV index is a standardized, linear measure of erythemally-weighted irradiance which can theoretically fall between 0 (at night) and 43 [37] but annual UV indices in the United States generally fall between 1 and 11, with each unit equal to 0.025 W/m^2^ [38]. CUES was calculated according to CUES = Σ (Hours of Exposure × UV Index). CUES, which has formal units of W*h/m^2^, is directly proportional to standard radiant exposure units (J/m^2^). As the CUES is an estimate only, and not data from dosimetry, we omit the actual units in the report of the CUES. Rather the CUES can be viewed as a relative measure, akin to the pack-year.

The participant’s lifetime was divided into one-year intervals, each of which was assigned a duration of exposure and a location of exposure based on questionnaire responses. Next, the location of exposure (in “City, State” format) was resolved into longitude and latitude coordinates using the publically-available Google Maps application program interface (API) and the closest anchor city was determined using the Haversine formula for great-circle distances. For each one-year interval, the hours of exposure was multiplied by the UV index for the nearest anchor city to obtain an annual UV dose estimate. This method was repeated for each one-year interval and the annual doses were summed to obtain a CUES. To minimize human error during this process, data acquisition, validation and CUES calculation was completely automated using an open source JavaScript program developed by the authors. We have provided the ~1,700 lines of code used to collect questionnaire data and calculate CUES as an open access resource at https://github.com/gefeizhu/cues-study so that future UV indices can be substituted, should they change significantly.

### Association of CUES with clinically relevant endpoints

We compared the CUES of individuals with skin cancer history prior to their first skin cancer diagnosis with the CUES of individuals without skin cancer history, in age-matched fashion. This enabled us to assess whether CUES could detect differences in lifetime ultraviolet exposures between those with and without skin cancer, recapitulating a well-known association in the medical literature [5, 6, 39].

Second, since sun protective behaviors and UV exposures have been reported in the literature to change after skin cancer diagnosis, [28, 40, 41] we assessed the ability of our instrument to detect differences in CUES among cases before and after first skin cancer diagnosis, as well as changes in tanning and sun protective behaviors before and after first skin cancer diagnosis.

### Statistical analysis

Student’s t-tests or chi-square tests were used in bivariate analyses on most continuous and categorical variables, respectively, for comparisons between individuals with a personal history of skin cancer and those without. Because age and CUES were not normally distributed, Wilcoxon’s rank-sum tests were used as nonparametric alternatives to t-tests in these comparisons. Individuals could report one or more types of family history of skin cancer, and therefore two-sample Student’s t-tests were used to compare these variables.

Each case was matched with 2 controls on age using standard propensity score matching. One case was excluded because the respondent had a first diagnosis of skin cancer before age 18. To estimate the odds of developing skin cancer, conditional logistic regression was applied in univariate and multivariate analyses. Interactions and multi-collinearity were assessed in determining the final multivariate model. Wilcoxon’s signed-rank test was used to compare CUES and Bowker’s test was used to compare sun protective behaviors, tanning bed use, and lifetime history of blistering sunburns before and after diagnosis of skin cancer among cases.

Test-retest reliability was assessed by calculating an intraclass correlation coefficient (ICC) for the CUES score and weighted kappa coefficients for categorical survey items. In general, kappa scores of 0.75 or greater reflect excellent agreement, scores of 0.40 to 0.75 reflect fair-to-good agreement, and scores below 0.4 reflect poor agreement [42]. The raw and standardized Cronbach’s alpha for the questionnaire items was 0.59.

In light of the Cronbach’s alpha score, factor analysis was performed on use of hat, use of long-sleeved clothing, shade-seeking behavior, use of sunscreen, Fitzpatrick skin type, lifetime history of blistering sunburns, and use of tanning beds to assess dimensionality. After applying eigenvalue selection criteria, one factor was retained, comprising: use of hat, use of long-sleeved clothing, shade-seeking behavior, use of sunscreen, Fitzpatrick skin type, lifetime number of blistering sunburns, and use of tanning beds.

All statistical analyses were conducted using SAS (Version 9.4, SAS Institute, Inc., Cary, North Carolina).

## Results

ResearchMatch sent emails to 60,480 individuals for this study. 1,120 (1.9 %) completed the study questionnaire, of which 1,118 (99.8 %) were completely filled out and met inclusion criteria by age and residential history.

This group was analyzed for the study and individuals residing in 47 states (see Additional file 3: Figure S1 for distribution of states). Both of the excluded questionnaires reported location(s) of residence outside of the United States for ≥1 year. A standard response rate could not be calculated from the online format, as there was no way to ascertain that the email had been read by the potential respondents in their inboxes.

The mean age of all study participants was 46 years (range 18 – 81), and 150 (13 %) individuals reported a personal history of skin cancer. Approximately 70 % of individuals participating in this study self-reported Fitzpatrick skin type 2–3, with 20 % self-reporting Fitzpatrick skin type 4–6. Other demographic information is presented in Table 1. The annual UV indices in the reported locales of study participants in the United States ranged from 1.88 to 10.29. (Additional file 1: Table S1).

**Table 1:** Characteristics for all participants by personal history of skin cancer, *N* = 1,118

Parameter	Negative personal history of skin cancer	Positive personal history of skin cancer	*p*
	(*n* = 968)	(*n* = 150)	
Age, mean (SD), in years	45.4 (14.6)	61.6 (11.6)	<0.0001
Family history of skin cancer, n (%)	286 (30)	82 (55)	<0.0001
Type of family history of skin cancer, n (%)^c^			
Basal cell carcinoma	123 (43)	66 (80)	<0.0001
Squamous cell carcinoma	58 (20)	31 (38)	0.0012
Melanoma	73 (26)	19 (23)	0.6528
Other	1 (0.4)	1 (1.2)	0.3464
Don’t know	80 (28)	7 (8)	0.0002
Skin type (Fitzpatrick), n (%)			0.001
1	71 (7)	9 (6)	
2	350 (36)	80 (53)	
3	315 (33)	35 (23)	
4 + 5 + 6	232 (24)	26 (17)	
Tanning bed use by number of visits,^a^ n (%)			0.1017
0	537 (55)	100 (67)	
1–5	157 (16)	18 (12)	
6–10	85 (9)	11 (7)	
11–100	152 (16)	19 (13)	
>100	37 (4)	2 (1)	
Lifetime number of blistering sunburns, n (%)			<0.0001
0	165 (17)	14 (9)	
1–5	524 (54)	72 (48)	
6–10	162 (17)	25 (17)	
>10	117 (12)	39 (26)	
Age at first skin cancer diagnosis, mean (SD), in years	NA	51.4 (13.8)	NA
Type of first skin cancer diagnosis, n (%)			
Basal cell carcinoma	NA	93 (62)	NA
Squamous cell carcinoma		20 (13)	
Melanoma		17 (11)	
Other		7 (5)	
Don’t know		13 (9)	
Factor score, median (range)^d^	0.2 (−2.0 – 2.2)	−0.2 (−2.3 – 1.4)	<0.0001
Lifetime total hours of exposure, median (range)	37,128 (2,496 – 156,520)	55,172 (14,976 – 157,248)	<0.0001
CUES^b^, median (range)	180,708 (11,288 – 778,332)	241,873 (44,858 – 898,344)	<0.0001

^a^Total number of tanning bed visits per lifetime (negative personal history of skin cancer) or total number of tanning bed visits up until first skin cancer diagnosis (positive personal history of skin cancer)

^b^Total CUES (negative personal history of skin cancer) or CUES prior to first skin cancer diagnosis (positive personal history of skin cancer)

^c^Individuals could report a family history of more than one type of cancer, therefore chi-squared testing was not possible

^d^A single factor was identified during factor analysis, which incorporated Fitzpatrick skin type, use of tanning bed, lifetime number of blistering sunburns, use of hat, use of long-sleeved clothing, shade-seeking behavior, and use of sunscreen

In bivariate analyses, individuals with a personal history of skin cancer were significantly older than those without a personal history of skin cancer (mean age 61.6 vs. 45.4, *p* < 0.0001), were more likely to have a family history of skin cancer (55 % vs. 30 %, *p* < 0.0001), and more likely to have Fitzpatrick skin types I or II (59 % vs. 43 %, *p* = 0.001).

To further characterize the relationship between development of skin cancer and clinical and UV exposure-related behavioral variables, factor analysis was performed on the questionnaire items. One factor was identified, with standardized scoring coefficients presented in Additional file 4: Table S2. Median factor scores of individuals with a personal history of skin cancer were significantly lower than those without (median −0.2 vs. 0.2, *p* < 0.0001), suggesting an inverse relationship between factor scores and skin cancer risk (Table 1).

### Association of CUES with personal history of skin cancer

The median CUES of individuals with a personal history of skin cancer was significantly higher than those without personal history of skin cancer (241,873 vs. 180,708, *p* = 0.0001), as was the total hours of exposure (55,172 vs. 37,128, *p* < 0.0001). The CUES distributions of individuals with and without a personal history of skin cancer overlapped significantly, as expected for a disease process with a multifactorial etiology (Table 1).

To assess if CUES is associated with development of skin cancer, a case–control format was utilized. In this part of the study, 149 cases (individuals with a personal history of skin cancer) were matched to 298 controls (individuals with no personal history of skin cancer), with exclusion of only one individual who reported a history of skin cancer prior to age 18 years. CUES at time of first skin cancer diagnosis (for those with a personal history of skin cancer) and total CUES (for those without) were divided into quartiles and used as the predictor of interest. In age-matched case–control analyses, individuals in the lowest quartile of CUES were less likely to report a positive history of skin cancer compared to those in the highest quartile (OR 0.4, 95 % CI 0.2 – 0.7). Cases had significantly greater CUES prior to first skin cancer diagnosis compared to total CUES of controls in matched analyses (242,074 vs. 205,379, *p* = 0.003) (Table 2).

**Table 2:** Sun-Protective behaviors and CUES among skin cancer cases and age-matched controls, *N* = 447

Parameter	Controls (*n* = 298)	Cases (*n* = 149)	Unadjusted Odds Ratio (95 % confidence interval)^a^	Adjusted Odds Ratio (95 % confidence interval)^b,c^
Fitzpatrick Skin Type, n (%)				
1	25 (8)	8 (5)	Ref	Incorporated into factor score^e^
2	112 (38)	80 (54)	2.26 (0.97 – 5.26)	
3	102 (34)	35 (23)	1.10 (0.46 – 2.63)	
4 + 5 + 6	59 (20)	26 (17)	1.40 (0.58 – 3.41)	
Use of tanning bed, n (%)				
0	177 (59)	99 (66)	Ref	Incorporated into factor score^e^
1–5	42 (14)	18 (12)	0.76 (0.41 – 1.42)	
6–10	29 (10)	11 (7)	0.66 (0.30 – 1.42)	
11–100	43 (14)	19 (13)	0.77 (0.42 – 1.42)	
>100	7 (2)	2 (1)	0.54 (0.11 – 2.59)	
Lifetime number of blistering sunburns, n (%)				
0	41 (14)	14 (9)	Ref	Incorporated into factor score^e^
1 –5	156 (52)	72 (48)	1.3 (0.7 – 2.6)	
- 10	58 (19)	24 (16)	1.2 (0.5 – 2.6)	
>10	43 (14)	39 (26)	2.6 (1.2 – 5.5)	
Use of hat, n (%)				
Never	78 (26)	32 (21)	Ref	Incorporated into factor score^e^
Rarely	77 (26)	63 (42)	1.89 (1.11 – 3.23)	
Sometimes	80 (27)	25 (17)	0.74 (0.39 – 1.41)	
Often	45 (15)	23 (15)	1.20 (0.62 – 2.32)	
Always	18 (6)	6 (4)	0.79 (0.28 – 2.21)	
Use of long-sleeved clothing, n (%)				
Never	13 (4)	19 (13)	Ref	Incorporated into factor score^e^
Rarely	34 (11)	31 (21)	0.70 (0.29 – 1.66)	
Sometimes	58 (19)	39 (26)	0.56 (0.25 – 1.26)	
Often	112 (38)	37 (25)	0.29 (0.14 – 0.63)	
Always	81 (27)	23 (15)	0.24 (0.11 – 0.55)	
Shade seeking, n (%)				
Never	25 (8)	25 (17)	Ref	Incorporated into factor score^e^
Rarely	68 (23)	50 (34)	0.80 (0.42 – 1.54)	
Sometimes	120 (40)	52 (35)	0.50 (0.27 – 0.93)	
Often	79 (27)	21 (14)	0.31 (0.16 – 0.63)	
Always	6 (2)	1 (1)	0.18 (0.02 – 1.76)	
Use of sunscreen, n (%)				
Never	16 (5)	13 (9)	Ref	Incorporated into factor score^e^
Rarely	55 (18)	44 (30)	1.01 (0.44 – 2.34)	
Sometimes	84 (28)	62 (41)	0.90 (0.41 – 1.97)	
Often	97 (33)	29 (19)	0.34 (0.15 – 0.82)	
Always	46 (15)	1 (1)	0.03 (0.003 – 0.23)	
Factor score, median (range)^e^	0.2 (−2.0 – 2.2)	−0.2 (−2.3 – 1.4)	0.56 (0.43 – 0.72)	0.54 (0.41 –0.71)
Family history of skin cancer, n (%)	92 (31)	81 (54)	2.63 (1.73 – 3.99)	3.06 (1.92 – 4.88)
CUES^d^, median (range)	205,379 (28,439 – 764,540)***	242,074 (44,858 – 898,344)***		
CUES^d^, quartilized, n (%)				
4^th^ Quartile	112 (25)	112 (25)	Ref	Ref
1^st^ Quartile	111 (25)	111 (25)	0.36 (0.19 – 0.69)	0.48 (0.24 – 0.97)
2^nd^ Quartile	112 (25)	112 (25)	0.62 (0.34 – 1.12)	0.76 (0.38 – 1.49)
3^rd^ Quartile	112 (25)	112 (25)	0.63 (0.37 – 1.10)	0.75 (0.41 – 1.37)
Lifetime total hours of exposure, median (range)	43,342 (6,630 – 156,520)	55,328 (14,976 – 157,248)		

^a^Univariate logistic regression

^b^Multivariate logistic regression

^c^Adjusted for quartilized CUES, family history of skin cancer, and a single factor comprising Fitzpatrick skin type, use of tanning bed, lifetime number of blistering sunburns, use of hat, use of long-sleeved clothing, shade-seeking, and use of sunscreen

^d^Total CUES (controls) or CUES prior to first skin cancer diagnosis (cases)

***p = 0.003 by Wilcoxon rank-sum test

^e^A single factor was identified during factor analysis, which incorporated Fitzpatrick skin type, use of tanning bed, lifetime number of blistering sunburns, use of hat, use of long-sleeved clothing, shade-seeking, and use of sunscreen

A multivariate model using conditional logistic regression showed that CUES was an independent risk factor for development of skin cancer, while controlling for family history of skin cancer and the factor score, which included Fitzpatrick skin type and several ultraviolet exposure-related variables (Additional file 4: Table S2). Individuals with CUES in the 1^st^ quartile (adjusted odds ratio [AOR] 0.5, 95 % CI 0.2 – 0.97) were less likely to develop skin cancer compared to those in the highest quartile independent of covariates (Table 2).

### Association of sun protective behaviors with personal history of skin cancer

In univariate analyses, cases were more likely to report “rarely” using hats (OR 1.9, 95 % CI 1.1 – 3.2) compared to controls. Cases were *less* likely to report “often” seeking shade (OR 0.3, 95 % CI 0.2 – 0.6), “often” (OR 0.3, 95 % CI 0.1 – 0.6) or “always” (OR 0.2, 95 % CI 0.1 – 0.6) using long-sleeved clothing, and “often” (OR 0.3, 95 % CI 0.2 – 0.8) or “always” (OR 0.03, 95 % CI 0.003 – 0.23) using sunscreen. Positive history of tanning bed use was not significantly different between cases and controls (Table 2).

In multivariate analysis using conditional logic regression, Fitzpatrick skin type and many UV exposure-related variables were collapsed into a single factor score. Cases reported a significantly lower factor score compared to controls (AOR 0.5, 95 % CI 0.4 – 0.7), consistent with the UV exposure-related risk factors included in the calculation of this factor (Table 2 and Additional file 4: Table S2).

After a skin cancer diagnosis, individuals with a personal history of skin cancer reported significantly increased engagement in sun-protective behaviors, including tanning bed avoidance (*p* = 0.0005), use of sunscreen (*p* < 0.0001), use of long-sleeved clothing (*p* < 0.0001), use of hat (*p* < 0.0001), shade seeking behavior (*p* < 0.0001), and reduced number of blistering sunburns (*p* < 0.0001). Additionally, there was a concordant decrease in the median annual CUES calculated after skin cancer diagnosis compared to before (4,970 vs. 3,087, *p* < 0.0001) (Table 3).

**Table 3:** Sun-Protective behaviors and CUES before and after skin cancer diagnosis among cases, *N* = 149

Parameter	Before skin cancer	After skin cancer	Percentage of absolute agreement	*P*
Annual CUES, median (range) units*	4,970 (1,057 – 16,042)	3,087 (0 – 13,353)		
Use of tanning bed, n (%)				
0	100 (67)	136 (91)	64 %	0.0005
1–5	17 (11)	0 (0)		
6–10	11 (7)	5 (3)		
11–100	18 (12)	6 (4)		
>100	2 (1)	1 (1)		
Use of sunscreen, n (%)				
Never	13 (9)	2 (1)	29 %	<0.0001
Rarely	44 (30)	9 (6)		
Sometimes	62 (42)	39 (26)		
Often	29 (19)	69 (46)		
Always	2 (1)	31 (21)		
Use of long-sleeved clothing, n (%)				
Never	19 (13)	7 (5)	41 %	<0.0001
Rarely	31 (21)	11 (7)		
Sometimes	39 (26)	28 (19)		
Often	37 (25)	45 (30)		
Always	24 (16)	59 (40)		
Use of hat, n (%)				
Never	32 (21)	10 (7)	29 %	<0.0001
Rarely	64 (43)	15 (10)		
Sometimes	25 (17)	37 (25)		
Often	23 (15)	55 (37)		
Always	6 (4)	33 (22)		
Shade seeking, n (%)				
Never	25 (17)	10 (7)	35 %	<0.0001
Rarely	50 (34)	16 (11)		
Sometimes	52 (35)	43 (29)		
Often	22 (15)	64 (43)		
Always	1 (1)	17 (11)		
Lifetime number of blistering sunburns, n (%)				
0	14 (9)	127 (85)	12 %	<0.0001
1 – 5	72 (48)	19 (13)		
6 – 10	25 (17)	1 (1)		
>10	39 (26)	3 (2)		

*P*-value determined by Bowker’s test

To promote the ease of use of the CUES score, a summary of scores (rounded to the nearest thousand units) relative to study participants is provided in Table 4, so that individuals can see whether their scores are above the median of study participants across the United States.

**Table 4:** How your CUES compares to individuals in the study

Your CUES^a^	How Your CUES compares to Individuals in the study
<205,000 units	Below median score for those without history of skin cancer
205,001–242,000 units	Above median score for those without history of skin cancer and below the median score for those with skin cancer
>242,001 units	Above median score for those with skin cancer

^a^The CUES reported in this table have been rounded to the nearest thousand

**Table 5:** Test-retest reliability, *N* = 19

Survey Item	Weighted kappa/ICC	95 % Confidence intervals	Percentage of absolute agreement
CUES	0.94^a^	0.86–0.98	NA
Fitzpatrick skin type	0.90	0.79–1.00	72 %
Family history of skin cancer	1.00	1.00–1.00	100 %
Use of sunscreen	0.84	0.71–0.98	72 %
Use of long-sleeved clothing	0.73	0.55–0.91	72 %
Use of hat	0.96	0.88–1.00	94 %
Shade seeking	0.43	0.23–1.00	83 %
History of blistering sunburns	0.87	0.70–1.00	83 %
Residential history	0.99	0.99–1.00	94 %

^a^Intraclass correlation coefficient (ICC)

### Reliability of CUES

To assess the reliability of CUES, a convenience sample of 19 individuals was recruited outside of ResearchMatch from the Stanford academic community and asked to fill out identical questionnaires before and after a 2 week period to assess the reliability of survey items. The weighted kappa/ICC for CUES was 0.94, (95 % CI, 0.86 – 0.98) indicating excellent reliability. Weighted kappa scores for other questionnaire items ranged from 0.43 (95 % CI 0.23 – 1.00) for shade seeking behavior to 1.00 (95 % CI 1.00 – 1.00) for family history of skin cancer (Table 5). While the weighted kappa for CUES is good, the small sample size from the Stanford academic community could limit its generalizability to the national survey population.

Feedback from respondents indicated the questionnaire and its online implementation was easy to understand, logical, and brief. All the respondents took less than 10 min to complete the survey.

## Discussion

We report an open-source questionnaire-based method of calculating cumulative UV radiation exposure that can be used to identify individuals with higher lifetime UV radiation exposures (e.g. >240,000 units, the median CUES of those with a history of skin cancer) and accounts for the variety of geographic differences across the United States. Increased CUES was associated with a positive personal history of skin cancer in the overall sample (*n* = 1,118) (Table 1) as well as the age-matched case–control subset analysis (*n* = 447) in univariate and multivariate analyses (Table 2). We also report a decrease in annual CUES and a concordant increase in sun protective habits following skin cancer diagnosis,(Table 3), thereby demonstrating the ability of CUES to detect changes in sun exposure after skin cancer diagnoses that is well known from previous studies [19, 26–28].

Current methods in the clinic to estimate an individual’s UV exposure are often uni-dimensional (such as “how many blistering sunburns have you had in your lifetime?”) or subjective (such as “do you spend a lot of time outdoors?”), and CUES enables systematic estimation that can be performed in the clinic waiting room such as with a patient’s own smartphone or tablet by logging onto the publically available website. CUES can then be incorporated as part of the discussion with dermatologists on whether more aggressive sun-protective measures may be beneficial. Of course, skin cancer risk is multifactorial, and includes factors other than cumulative UV exposure such as patient’s co-morbid medical conditions, family history, or Fitzpatrick skin type. Moreover, CUES does not contribute information about the intermittency of exposure events, which has been positively correlated with an increased risk of developing melanoma [43]. This tool does not replace clinical judgment to assess individual risk for skin cancer.

Nevertheless, a potential use of the CUES may be to motivate at-risk patients to reduce ongoing UV exposure through immediate, personalized feedback in clinic or through other means of conveying such information (e.g. secure email or messaging systems). The ability of immediate feedback to alter sun protective behaviors has been shown, for instance through a mobile phone application that delivered real-time, location-based UV index data and encouraged sun protection [44]. Whether receipt of a CUES upon completion of the survey tool improves sun protective behaviors is the subject of a forthcoming study.

Several important simplifying assumptions were made in designing this study. First, since average annual UV indices were used, we did not account for important diurnal, seasonal or yearly differences in the UV index. Second, we were not able to adjust our CUES using traditional means, such as applying corrections for body posture (the UV index is typically measured using dosimeters installed on a horizontal surface). Finally, we assumed that self-reported frequencies of exposure (expressed in hours per week) were constant for epochs spanning several years. Though future refinements may enable these assumptions to be diminished or eliminated, a statistically-significant difference in CUES was detectable between those with more or less sun-protective behaviors and with skin cancer diagnosis, even with the aforementioned assumptions.

CUES is not intended to substitute for precise measurements of UV exposure through dosimetry. While dosimetry is the gold standard method of estimating UV exposure in short-term research studies, it is not currently realistic for individuals to carry a dosimeter over many decades. Dosimetry is also problematic due to possible heterogeneity in the location the device is worn between studies and the potential lack of adherence in wearing the device. We do not claim that CUES is a physical measure of actual UV exposure by the survey respondents but is rather an estimate based on self-reported data. In lieu of testing the content validity of our measure, we elected to validate CUES by reporting its ability to detect a well-established association between UV exposure and sun protective behaviors and skin cancer.

Moreover, since CUES is not a precise measure of actual UV dosage, we avoided reporting the formal units of the CUES. Our intention is for CUES to be used to identify patients at risk for skin cancer in a similar way as “pack-years” is used to stratify lung cancer risk in smokers. While CUES does not reflect a physical measurement of UV dose received by an individual, CUES can be easily incorporated into epidemiological studies in which lifetime UV exposure is a covariate or independent variable of interest. We envision that the CUES will primarily be of interest to clinicians, as it relies on self-reported data ascertainable in clinic waiting rooms. Of course, the CUES is a crude measurement when compared to studies employing dosimetry, diaries, or interviews, which allow for precise quantitation of UV exposure in physical units. However, such methodological rigor does not add to clinical utility, just as knowing the precise number of cigarettes smoked does not substantially alter to the management of a smoker who is known to have a high pack-year history.

There are several important limitations in this study. First, we were unable to detect increased odds of developing skin cancer among individuals reporting tanning bed use, either in the entire sample (Table 1) or in case–control analyses (Table 2). We stratified tanning bed use by age, such as aged ≤54 or >54 years, the median age in our study, but this did not yield different results (data not shown). The strong relationship between tanning bed use and UV-dependent skin cancers, particularly melanoma, is well established in the literature [45]. Our inability to recapitulate these findings in this study is likely a combination of (1) low sample size, given that only 9 individuals reported using tanning beds >100 times in their lifetimes, (2) heterogeneity in the dose and spectral characteristics of tanning bed UV radiation, [46] and (3) our inability to specify the age(s) at which our respondents used tanning beds, as tanning during adolescence and young adulthood is more strongly associated with subsequent skin cancers [45, 47].

A second limitation is potential selection bias, in that only 1.9 % of individuals contacted by ResearchMatch completed the questionnaire. It is possible that there were important undetected differences between responders and nonresponders, however, the ResearchMatch platform did not allow for this type of assessment.

A third and most important limitation is the possibility of recall bias, which is inherent in the retrospective and self-reported nature of this study. This was carefully considered in the design of the study, and measures were taken to minimize this bias, namely (A) separating pairs of identical questions asking about sun protective behaviors and tanning bed use before and after diagnosis of skin cancer among those with a positive history and (B) constraining exposure duration responses to fixed age epochs (0 – 13, 13 – 20, 20 – 40, 40 – 65, 65 – 80, and 80+) rather than having the epochs fall before and after first skin cancer diagnosis. This study would not be feasible to conduct in prospective fashion due to the need to follow large numbers of individuals nationally over many decades with dosimetry data or serial questionnaire administrations. It is not possible to prospectively “blind” patients to their own diagnosis of skin cancer, and therefore it would be impossible to remove this potential recall bias. The retrospective format of CUES is consistent with how it would be used in the clinical setting, that is, as a tool that could be used before or after eliciting the past medical history, itself a retrospective but necessary part of everyday clinical care that is also prone to recall bias.

In fact, multiple clinical measures in fields outside of dermatology in common use rely on patient self-report, such as the New York Heart Association (NYHA) functional classification in cardiology or the Eastern Cooperative Oncology Group (ECOG) performance status score used in oncology. These measures, like the CUES, are subject to recall bias but are used ubiquitously in their respective clinical contexts due to their utility. Nevertheless, we envision that recall bias could be explored in future studies, for example by comparing self-reported data with observer-reported data such as those provided by family members or caregivers, though such data also carry the risk of bias.

## Conclusions

Finally, we make the CUES calculation tool freely available online in two ways. First, the source code is available for modification, for instance, should UV indices change in the future or if customization is needed for specific populations. Second, the CUES calculator can be accessed at the link reported in the Methods section for individual calculation, the result of which can be provided to a dermatologist or other health care provider by the patient in their discussion of photo-protective measures or skin cancer risk.

## Additional files

Additional file 1: Table S1. Calculated Annual Average UV Indices for 58 Anchor Cities in the United States. UV indices in this study ranged from 1.88 to 10.29. (DOC 73 kb) Additional file 2:
**Supplemental Materials.** (PDF 954 kb) Additional file 3: Figure S1. The Pacific Northwest includes WA, OR and CA. Atlantic Northeast includes MA, NY, DE, RI, Washington DC, ME, CT and NH. Atlantic South includes FL, AL, LA, SC, NC, GA, VA. Central U.S. includes all other states besides those previously mentioned. (JPG 60 kb) Additional file 4: Table S2. Scoring Coefficients Used in Factor Analysis. (DOC 30 kb)

## Abbreviations

AOR: Adjusted odds ratio
API: Application program interface
CTSA: Clinical Translational Science Award
CUES: Cumulative ultraviolet exposure score
h: Hour
J: Joule
m: Meter
SD: Standard deviation
UV: Ultraviolet
W: Watt
