# Supplementary material for: Estimation of individual cumulative ultraviolet exposure using a geographically-adjusted, openly-accessible tool
Source: BMC Dermatol. 2016 Jan 20;16:1. doi: 10.1186/s12895-016-0038-1 (PMC4721109; doi:10.1186/s12895-016-0038-1)
Supplement: Additional file 1: Table S1. — Calculated Annual Average UV Indices for 58 Anchor Cities in the United States. UV indices in this study ranged from 1.88 to 10.29. (DOC 73 kb) [file 12895_2016_38_MOESM1_ESM.doc]

Supplemental Table S1. Calculated Annual Average UV Indices for 58 Anchor Cities in the United States. UV indices in this study ranged from 1.88 to 10.29.

| **City** | **State** | **2009 – 2012 Annual Average UV Index** | |
| --- | --- | --- | --- |
|  |  | **Mean** | **Standard Deviation** |
| Anchorage | AK | 1.88 | 0.09 |
| Mobile | AL | 7.00 | 0.36 |
| Little Rock | AR | 5.69 | 0.36 |
| Phoenix | AZ | 6.94 | 0.35 |
| Los Angeles | CA | 6.94 | 0.24 |
| San Francisco | CA | 5.70 | 0.26 |
| Denver | CO | 5.90 | 0.26 |
| Hartford | CT | 4.23 | 0.20 |
| Washington | DC | 4.71 | 0.25 |
| Dover | DE | 4.60 | 0.31 |
| Miami | FL | 8.50 | 0.17 |
| Tampa | FL | 7.77 | 0.23 |
| Jacksonville | FL | 7.09 | 0.21 |
| Atlanta | GA | 5.99 | 0.43 |
| Honolulu | HI | 9.38 | 0.13 |
| Des Moines | IA | 4.41 | 0.15 |
| Boise | ID | 4.79 | 0.26 |
| Chicago | IL | 4.24 | 0.28 |
| Indianapolis | IN | 4.61 | 0.31 |
| Wichita | KS | 5.22 | 0.22 |
| Louisville | KY | 4.83 | 0.30 |
| New Orleans | LA | 7.35 | 0.19 |
| Boston | MA | 4.21 | 0.21 |
| Baltimore | MD | 4.63 | 0.25 |
| Portland | ME | 3.83 | 0.17 |
| Detroit | MI | 4.04 | 0.25 |
| Minneapolis | MN | 3.86 | 0.11 |
| St. Louis | MO | 4.92 | 0.27 |
| Jackson | MS | 6.46 | 0.29 |
| Billings | MT | 4.40 | 0.19 |
| Raleigh | NC | 5.55 | 0.28 |
| Bismarck | ND | 3.91 | 0.20 |
| Omaha | NE | 4.54 | 0.19 |
| Concord | NH | 3.97 | 0.19 |
| Atlantic City | NJ | 4.64 | 0.32 |
| Albuquerque | NM | 7.07 | 0.41 |
| Las Vegas | NV | 6.28 | 0.23 |
| New York | NY | 4.43 | 0.25 |
| Buffalo | NY | 4.01 | 0.24 |
| Cleveland | OH | 4.24 | 0.29 |
| Oklahoma City | OK | 5.64 | 0.25 |
| Portland | OR | 3.55 | 0.24 |
| Philadelphia | PA | 4.41 | 0.26 |
| Pittsburgh | PA | 4.26 | 0.29 |
| San Juan | PU | 10.29 | 0.13 |
| Providence | RI | 4.26 | 0.22 |
| Charleston | SC | 6.35 | 0.31 |
| Sioux Falls | SD | 4.18 | 0.18 |
| Memphis | TN | 5.61 | 0.27 |
| Houston | TX | 6.91 | 0.20 |
| Dallas | TX | 6.02 | 0.22 |
| Salt Lake City | UT | 5.72 | 0.27 |
| Norfolk | VA | 5.38 | 0.30 |
| Burlington | VT | 3.63 | 0.32 |
| Seattle | WA | 3.37 | 0.20 |
| Milwaukee | WI | 4.09 | 0.25 |
| Charleston | WV | 4.69 | 0.27 |
| Cheyenne | WY | 5.50 | 0.33 |
