# Supplementary material for: Estimation of individual cumulative ultraviolet exposure using a geographically-adjusted, openly-accessible tool
Source: BMC Dermatol. 2016 Jan 20;16:1. doi: 10.1186/s12895-016-0038-1 (PMC4721109; doi:10.1186/s12895-016-0038-1)
Supplement: Additional file 2: — Supplemental Materials. (PDF 954 kb) [file 12895_2016_38_MOESM2_ESM.pdf]

Supplemental Materials

This document contains two copies of empty survey instruments, one assuming no personal history of skin cancer and one assuming a personal history of skin cancer. The slight differences between the two survey instruments are due to implementation of skip patterns.

Following these screenshots, a complete transcript of the hypertext markup language (HTML), cascading style sheets (CSS), and Javascript code used to calculate the cumulative ultraviolet exposure score (CUES) as described in the accompanying manuscript is provided. The file “index.html” provides the bulk of the programming code and the user interface for calculating the CUES, and the files “jquery-1.11.3.min.js,” “underscore-min.js,” “backbone-min.js,” “typeahead.jquery.js,” “cities.js,” “svg.min.js,” and “bootstrap.min.css” are ancillary code packages incorporated by reference in “index.html.” Many of these packages are not the original work of the authors, but are made publicly available under various licenses. When appropriate, links to the developer websites of these packages are listed below.

The materials are additionally available electronically at <https://github.com/gefeizhu/cues-study>

Table of Contents

CUES Survey With No Personal History of Skin Cancer.....	2
CUES Survey With A Personal History of Skin Cancer	6
index.html	11
jquery-1.11.3.min.js	43
underscore-min.js	44
backbone-min.js.....	45
typeahead.jquery.js	46
cities.js.....	47
svg.min.js.....	166
bootstrap.min.css.....	167

Cumulative UV Exposure Score Calculator

Instructions

This is a live demonstration of the questionnaire instrument and cumulative ultraviolet exposure score (CUES) calculation methodology for *Zhu et al. 2015*. This demonstration version of the questionnaire was modified so that no data is collected or stored, but is otherwise a exact reproduction of the instrument described in the accompanying manuscript. A CUES (rounded to the nearest 1,000 units for ease of viewing) will be calculated from the entered data once the user hits "Submit" (unrounded data were used for data analysis in the manuscript). This questionnaire was studied for individuals aged 18 - 81 living for no more than 1 year outside of the United States.

Please visit our up-to-date GitHub repository at <http://www.github.com/gefeizhu/cues-study> (<http://www.github.com/gefeizhu/cues-study>) for updated versions of this questionnaire.

Questions

1. What is your age?

years old.

2. How would you rate your skin type - do you burn or tan in the sun?

- ☐ Type I - Always burns, never tans (pale white skin)
- ☐ Type II - Burns easily, tans minimally (white skin)
- ☐ Type III - Burns moderately, tans uniformly (light brown skin)
- ☐ Type IV - Burns minimally, tans well (moderate brown skin)
- ☐ Type V - Rarely burns, tans profusely (dark brown skin)
- ☐ Type VI - Never burns (deeply pigmented dark brown to black skin)

3. Has one or more of your parents, siblings, or children ever been diagnosed with skin cancer? (Skip to question #5 if the answer is 'No'.)

- ☐ Yes
- ☐ No

4. What type of skin cancer was he/she diagnosed with? You may select more than one.

- ☐ Basal cell carcinoma (BCC)
- ☐ Squamous cell carcinoma (SCC)
- ☐ Melanoma
- ☐ Other
- ☐ Don't know

5. Have you ever been diagnosed with a skin cancer? (Skip to question #8 if the answer is 'No'.)

- ☐ Yes
- ☐ No

No

6. How old were you when you had your first skin cancer? years old.**7. When you had your first skin cancer, what type was it?**

- ☐ Basal cell carcinoma (BCC)
☐ Squamous cell carcinoma (SCC)
☐ Melanoma
☐ Other
☐ Don't know

8. How many times have you used a tanning bed in your life?

- ☐ 0
☐ 1 - 5
☐ 6 - 10
☐ 11 - 100
☐ greater than 100

9. How many blistering sunburns have you had in your lifetime?

- ☐ 0
☐ 1 - 5
☐ 6 - 10
☐ greater than 10

10. When you are outdoors in the sun, how often do you use sunscreen?

- ☐ Never
☐ Rarely
☐ Sometimes
☐ Often
☐ Always

11. For the following questions, think about what you do when you are outside during the summer on a warm sunny day.

	Never	Rarely	Sometimes	Often	Always
How often do you wear a shirt with sleeves that cover your shoulders?	☐
How often do you wear a hat?	☐
How often do you stay in the shade or under an umbrella?	☐

Estimated Outdoor Exposure Time

Please fill in the hours you spent outdoors between 10 AM and 4 PM per day. As you fill in the hours of exposure, a blue shading effect will be applied to the timeline below to the corresponding epoch. Fill in this section completely before moving on to the next section, **as you will not be able to change your responses once you start entering**

your locations of residence in the next section.

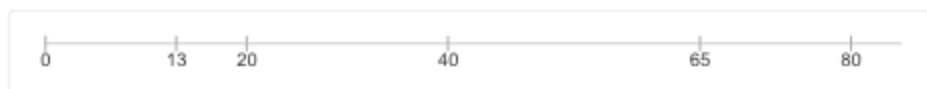

Location	Age Range	Weekdays (Hrs/Day)	Weekends (Hrs/Day)
	0 - 13	____	____
	13 - 20	____	____
	20 - 40	____	____
	40 - 65	____	____
	65 - 80	____	____
	80+	____	____

Exposure Location Data

Fill in the places you have primarily lived in and what age you lived there. More spaces will appear as you enter the first three lines. Your location responses will automatically appear next to the corresponding age ranges in the above table for your convenience.

Location	Starting Age	Ending Age
City, State	0	____
City, State	____	____
City, State	____	____

Feedback

Do you have any feedback about the survey?

Interpreting the CUES

Your personalized CUES will appear here once you complete the questionnaire and click "Submit."

Table 1. How your CUES compares to individuals in the study. *The CUES reported in this table have been rounded to the nearest thousand.

**Your CUES** *	**How Your CUES Compares to Individuals in the Study**
<205,000 units	Below median score for those without history of skin cancer
205,001-242,000 units	Above median score for those without history of skin cancer and below the median score for those with skin cancer
>242,001 units	Above median score for those with skin cancer

References

- Glanz K, Mccarty F, Nehl EJ, et al. Validity of self-reported sunscreen use by parents, children, and lifeguards. Am J Prev Med. 2009;36(1):63-9.
- Glanz K, Yaroch AL, Dancel M, et al. Measures of sun exposure and sun protection practices for behavioral and epidemiologic research. Arch Dermatol. 2008;144(2):217-22.

Cumulative UV Exposure Score Calculator

Instructions

This is a live demonstration of the questionnaire instrument and cumulative ultraviolet exposure score (CUES) calculation methodology for *Zhu et al. 2015*. This demonstration version of the questionnaire was modified so that no data is collected or stored, but is otherwise a exact reproduction of the instrument described in the accompanying manuscript. A CUES (rounded to the nearest 1,000 units for ease of viewing) will be calculated from the entered data once the user hits "Submit" (unrounded data were used for data analysis in the manuscript). This questionnaire was studied for individuals aged 18 - 81 living for no more than 1 year outside of the United States.

Please visit our up-to-date GitHub repository at <http://www.github.com/gefeizhu/cues-study> (<http://www.github.com/gefeizhu/cues-study>) for updated versions of this questionnaire.

Questions

1. What is your age?

years old.

2. How would you rate your skin type - do you burn or tan in the sun?

- ☐ Type I - Always burns, never tans (pale white skin)
- ☐ Type II - Burns easily, tans minimally (white skin)
- ☐ Type III - Burns moderately, tans uniformly (light brown skin)
- ☐ Type IV - Burns minimally, tans well (moderate brown skin)
- ☐ Type V - Rarely burns, tans profusely (dark brown skin)
- ☐ Type VI - Never burns (deeply pigmented dark brown to black skin)

3. Has one or more of your parents, siblings, or children ever been diagnosed with skin cancer? (Skip to question #5 if the answer is 'No'.)

- ☐ Yes
- ☐ No

4. What type of skin cancer was he/she diagnosed with? You may select more than one.

- ☐ Basal cell carcinoma (BCC)
- ☐ Squamous cell carcinoma (SCC)
- ☐ Melanoma
- ☐ Other
- ☐ Don't know

5. Have you ever been diagnosed with a skin cancer? (Skip to question #8 if the answer is 'No'.)

- ☒ Yes
- ☐ No

No

6. How old were you when you had your first skin cancer? years old.**7. When you had your first skin cancer, what type was it?**

- ☐ Basal cell carcinoma (BCC)
- ☐ Squamous cell carcinoma (SCC)
- ☐ Melanoma
- ☐ Other
- ☐ Don't know

8. Before you were diagnosed with skin cancer, how many times did you use a tanning bed?

- ☐ 0
- ☐ 1 - 5
- ☐ 6 - 10
- ☐ 11 - 100
- ☐ greater than 100

9. Before you were diagnosed with skin cancer, how many blistering sunburns did you have?

- ☐ 0
- ☐ 1 - 5
- ☐ 6 - 10
- ☐ greater than 10

10. Before you were diagnosed with skin cancer, how often did you use sunscreen when you were outside?

- ☐ Never
- ☐ Rarely
- ☐ Sometimes
- ☐ Often
- ☐ Always

11. Before you were diagnosed with skin cancer, how often did you do the following when you were outside during the summer on a warm sunny day?

	Never	Rarely	Sometimes	Often	Always
How often did you wear a shirt with sleeves that covered your shoulders?	☐
How often did you wear a hat?	☐
How often did you stay in the shade or under an umbrella?	☐

12. After you were diagnosed with skin cancer, how many times did you use a tanning bed?

- ☐ 0
- ☐ 1 - 5
- ☐ 6 - 10
- ☐ 11 - 100
- ☐

greater than 100

13. **After you were diagnosed with skin cancer, how many blistering sunburns did you have?**

- ☐ 0
☐ 1 - 5
☐ 6 - 10
☐ greater than 10

14. **After you were diagnosed with skin cancer, how often did you use sunscreen when you were outside?**

- ☐ Never
☐ Rarely
☐ Sometimes
☐ Often
☐ Always

15. **After you were diagnosed with skin cancer, how often do you do the following when you are outside during the summer on a warm sunny day?**

	Never	Rarely	Sometimes	Often	Always
How often do you wear a shirt with sleeves that cover your shoulders?	☐
How often do you wear a hat?	☐
How often do you stay in the shade or under an umbrella?	☐

Estimated Outdoor Exposure Time

Please fill in the hours you spent outdoors between 10 AM and 4 PM per day. As you fill in the hours of exposure, a blue shading effect will be applied to the timeline below to the corresponding epoch. Fill in this section completely before moving on to the next section, as you will not be able to change your responses once you start entering your locations of residence in the next section.

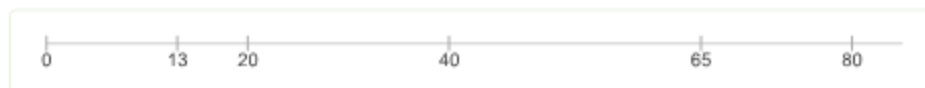

Location	Age Range	Weekdays (Hrs/Day)	Weekends (Hrs/Day)
	0 - 13	____	____
	13 - 20	____	____
	20 - 40	____	____
	40 - 65	____	____
	65 - 80	____	____
	80+	____	____

Exposure Location Data

Fill in the places you have primarily lived in and what age you lived there. More spaces will appear as you enter the first three lines. Your location responses will automatically appear next to the corresponding age ranges in the above table for your convenience.

Location	Starting Age	Ending Age
City, State	0	____
City, State	____	____
City, State	____	____

Feedback

Do you have any feedback about the survey?

Interpreting the CUES

Your personalized CUES will appear here once you complete the questionnaire and click "Submit."

Table 1. How your CUES compares to individuals in the study. *The CUES reported in this table have been rounded to the nearest thousand.

Your CUES*	How Your CUES Compares to Individuals in the Study
<205,000 units	Below median score for those without history of skin cancer
205,001-242,000 units	Above median score for those without history of skin cancer and below the median score for those with skin cancer
>242,001 units	Above median score for those with skin cancer

References

- Glanz K, Mccarty F, Nehl EJ, et al. Validity of self-reported sunscreen use by parents, children, and lifeguards. *Am J Prev Med.* 2009;36(1):63-9.
- Glanz K, Yaroch AL, Dancel M, et al. Measures of sun exposure and sun protection practices for behavioral and epidemiologic research. *Arch Dermatol.* 2008;144(2):217-22.

index.html

```
<!DOCTYPE html>
<html>
<head>
  <title>Cumulative UV Exposure Score Calculator</title>
  <script src="js/jquery-1.11.3.min.js"></script>
  <script src="js/underscore-min.js"></script>
  <script src="js/backbone-min.js"></script>
  <script src="js/typeahead.jquery.js"></script>
  <script src="js/cities.js"></script>
  <script
src="https://maps.googleapis.com/maps/api/js?key=AIzaSyBy3oqwIIvN3UePDleDhaYdAF5HHEHpKsU"
></script>
  <script src="js/svg.min.js"></script>
  <link href="css/bootstrap.min.css" rel="stylesheet">

  <style>
body {
  padding-top: 50px;
}

.etable {
  border-top: 1px solid #ddd;
}

.etable th {
  border-bottom: 1px solid #ddd;
}

.etable td, .etable th {
  padding-top: 4px;
  padding-bottom: 4px;
}

.question {
  font-weight: bold;
}

.qtable {
  border-top: 1px solid #ddd;
  border-bottom: 1px solid #ddd;
}

.qtable th {
  text-align: center;
}

.qtable td {
  border-top: 1px solid #ddd;
  padding: 3px;
}

.qtable tr td {
  text-align: center;
}

.qtable tr td:first-child {
  text-align: left;
}
```

```

.specific-disabled {
  color: #bbbbbb;
}

.specific-hidden {
  display: none;
}

.typeahead,
.tt-query,
.tt-hint {
}

.typeahead {
  background-color: #fff;
}

.tt-dropdown-menu {
  width: 190px;
  margin-top: 0px;
  padding: 0 0;
  background-color: #fff;
  border: 1px solid #ccc;
  border: 1px solid rgba(0, 0, 0, 0.2);
  -webkit-box-shadow: 0 5px 10px rgba(0,0,0,.2);
  -moz-box-shadow: 0 5px 10px rgba(0,0,0,.2);
  box-shadow: 0 5px 10px rgba(0,0,0,.2);
}

.tt-suggestion {
  padding: 3px 4px;
  line-height: 24px;
}

.tt-suggestion.tt-cursor {
  color: #fff;
  background-color: #0097cf;
}

.tt-suggestion p {
  margin: 0;
}
</style>
</head>
<body>

<div class="navbar navbar-inverse navbar-fixed-top" role="navigation">
  <div class="container">
    <div class="navbar-header">
      <button type="button" class="navbar-toggle" data-toggle="collapse" data-
target=".navbar-collapse">
        <span class="sr-only">Toggle navigation</span>
        <span class="icon-bar"></span>
        <span class="icon-bar"></span>
        <span class="icon-bar"></span>
      </button>
      <!--<a class="navbar-brand" href="#">Lifetime UV Exposure</a-->
    </div>
    <div class="collapse navbar-collapse">
      <ul class="nav navbar-nav">

```

```

        <li class="active"><a href="/">Home</a></li>
        <!--<li><a href="#about">About</a></li>-->
    </ul>
</div>
</div>
</div>

```

```

<div class="container">

```

```

<h1>Cumulative UV Exposure Score Calculator</h1>

```

```

<br>
<div class="panel panel-success">
    <div class="panel-heading">
        <h3 class="panel-title">Instructions</h3>
    </div>
    <div class="panel-body">

```

This is a live demonstration of the questionnaire instrument and cumulative ultraviolet exposure score (CUES) calculation methodology for *Zhu et al.* **2015**. This demonstration version of the questionnaire was modified so that no data is collected or stored, but is otherwise a exact reproduction of the instrument described in the accompanying manuscript. A CUES (rounded to the nearest 1,000 units for ease of viewing) will be calculated from the entered data once the user hits "Submit" (unrounded data were used for data analysis in the manuscript). This questionnaire was studied for individuals aged 18 - 81 living for no more than 1 year outside of the United States.

Please visit our up-to-date GitHub repository at <http://www.github.com/gefeizhu/cues-study> for updated versions of this questionnaire.

```

    </div>
</div>

```

```

<h3>Questions</h3>

```

```

<p class="question">1. What is your age?</p>

```

```

<div>
    <input type="text" name="age" id="age" style="width: 40px;" autocomplete="off"
onkeypress="return isNumber(event)" /> years old.
</div>

```

```

<div style="height: 6px;"></div>

```

```

<p class="question">2. How would you rate your skin type - do you burn or tan in the
sun?</p>

```

```

<div class="radio">
    <label><input type="radio" name="skin_type" value="1" onclick="initializeTable()"
/>Type I - Always burns, never tans (pale white skin)</label><br>
    <label><input type="radio" name="skin_type" value="2" onclick="initializeTable()"
/>Type II - Burns easily, tans minimally (white skin)</label><br>
    <label><input type="radio" name="skin_type" value="3" onclick="initializeTable()"
/>Type III - Burns moderately, tans uniformly (light brown skin)</label><br>
    <label><input type="radio" name="skin_type" value="4" onclick="initializeTable()"
/>Type IV - Burns minimally, tans well (moderate brown skin)</label><br>
    <label><input type="radio" name="skin_type" value="5" onclick="initializeTable()"
/>Type V - Rarely burns, tans profusely (dark brown skin)</label><br>

```

```

    <label><input type="radio" name="skin_type" value="6" onclick="initializeTable()"
/>Type VI - Never burns (deeply pigmented dark brown to black skin)</label>
</div>

<div style="height: 6px;"></div>
<p class="question">3. Has one or more of your parents, siblings, or children ever been
diagnosed with skin cancer?
<span style="font-weight: normal;">(Skip to question #5 if the answer is
'No'.)</span></p>

<div class="radio">
    <label><input type="radio" name="family_skin_cancer" value="yes" />Yes</label><br>
    <label><input type="radio" name="family_skin_cancer" value="no" />No</label>
</div>

<span id="family_skin_cancer_specific" class="specific-disabled">

    <div style="height: 6px;"></div>
    <p class="question">4. What type of skin cancer was he/she diagnosed with? You may
select more than one.</p>

    <div class="checkbox">
        <label><input type="checkbox" name="family_skin_cancer_type" value="bcc"
disabled="disabled" />Basal cell carcinoma (BCC)</label><br>
        <label><input type="checkbox" name="family_skin_cancer_type" value="scc"
disabled="disabled" />Squamous cell carcinoma (SCC)</label><br>
        <label><input type="checkbox" name="family_skin_cancer_type" value="melanoma"
disabled="disabled" />Melanoma</label><br>
        <label><input type="checkbox" name="family_skin_cancer_type" value="other"
disabled="disabled" />Other</label><br>
        <label><input type="checkbox" name="family_skin_cancer_type" value="dontknow"
disabled="disabled" />Don't know</label>
    </div>

</span>

<div style="height: 6px;"></div>
<p class="question">5. Have you ever been diagnosed with a skin cancer?
<span style="font-weight: normal;">(Skip to question #8 if the answer is
'No'.)</span></p>

<div class="radio">
    <label><input type="radio" name="skin_cancer" value="yes" />Yes</label><br>
    <label><input type="radio" name="skin_cancer" value="no" />No</label>
</div>

<span id="skin_cancer_specific" class="specific-disabled">

    <div style="height: 6px;"></div>
    <p class="question">6. How old were you when you had your first skin cancer?</p>

    <div>
        <input type="text" name="skin_cancer_age" id="skin_cancer_age" style="width: 40px;"
autocomplete="off" disabled="disabled" onkeypress="return isNumber(event)" /> years old.
        <br>
        <span style="color: red;" id="skin_cancer_age_error"></span>
    </div>

```

```

<div style="height: 6px;"></div>
<p class="question">7. When you had your first skin cancer, what type was it?</p>

<div class="radio">
  <label><input type="radio" name="skin_cancer_type" value="bcc" disabled="disabled"
/>Basal cell carcinoma (BCC)</label><br>
  <label><input type="radio" name="skin_cancer_type" value="scc" disabled="disabled"
/>Squamous cell carcinoma (SCC)</label><br>
  <label><input type="radio" name="skin_cancer_type" value="melanoma"
disabled="disabled" />Melanoma</label><br>
  <label><input type="radio" name="skin_cancer_type" value="other"
disabled="disabled" />Other</label><br>
  <label><input type="radio" name="skin_cancer_type" value="dontknow"
disabled="disabled" />Don't know</label>
</div>

</span>

<div style="height: 6px;"></div>
<p class="question" id="question_tanning_bed">8. How many times have you used a tanning
bed in your life?</p>

<div class="radio">
  <label><input type="radio" name="tanning_bed" value="0" />0</label><br>
  <label><input type="radio" name="tanning_bed" value="1 - 5" />1 - 5</label><br>
  <label><input type="radio" name="tanning_bed" value="6 - 10" />6 - 10</label><br>
  <label><input type="radio" name="tanning_bed" value="11 - 100" />11 - 100</label><br>
  <label><input type="radio" name="tanning_bed" value="> 100" />greater than
100</label>
</div>

<div style="height: 6px;"></div>
<p class="question" id="question_sunburn">9. How many blistering sunburns have you had
in your lifetime?</p>

<div class="radio">
  <label><input type="radio" name="sunburn" value="0" />0</label><br>
  <label><input type="radio" name="sunburn" value="1 - 5" />1 - 5</label><br>
  <label><input type="radio" name="sunburn" value="6 - 10" />6 - 10</label><br>
  <label><input type="radio" name="sunburn" value="> 10" />greater than 10</label>
</div>

<div style="height: 6px;"></div>
<p class="question" id="question_sunscreen">10. When you are outdoors in the sun, how
often do you use sunscreen?</p>

<div class="radio">
  <label><input type="radio" name="sunscreen" value="0" />Never</label><br>
  <label><input type="radio" name="sunscreen" value="1" />Rarely</label><br>
  <label><input type="radio" name="sunscreen" value="2" />Sometimes</label><br>
  <label><input type="radio" name="sunscreen" value="3" />Often</label><br>
  <label><input type="radio" name="sunscreen" value="4" />Always</label>
</div>

<div style="height: 6px;"></div>

```

<p class="question" id="question9">11. For the following questions, think about what you do when you are outside during the summer on a warm sunny day.</p>

```
<table width="700px" class="qtable">
  <tr>
    <th></th>
    <th width="12%">Never</th>
    <th width="12%">Rarely</th>
    <th width="12%">Sometimes</th>
    <th width="12%">Often</th>
    <th width="12%">Always</th>
  </tr>
  <tr>
    <td>How often <span class="v_do">do</span> you wear a shirt with sleeves that <span
class="v_cover">cover</span> your shoulders?</td>
    <td><input type="radio" name="q_shirt" value="0" /></td>
    <td><input type="radio" name="q_shirt" value="1" /></td>
    <td><input type="radio" name="q_shirt" value="2" /></td>
    <td><input type="radio" name="q_shirt" value="3" /></td>
    <td><input type="radio" name="q_shirt" value="4" /></td>
  </tr>
  <tr>
    <td>How often <span class="v_do">do</span> you wear a hat?</td>
    <td><input type="radio" name="q_hat" value="0" /></td>
    <td><input type="radio" name="q_hat" value="1" /></td>
    <td><input type="radio" name="q_hat" value="2" /></td>
    <td><input type="radio" name="q_hat" value="3" /></td>
    <td><input type="radio" name="q_hat" value="4" /></td>
  </tr>
  <tr>
    <td>How often <span class="v_do">do</span> you stay in the shade or under an
umbrella?</td>
    <td><input type="radio" name="q_shade" value="0" /></td>
    <td><input type="radio" name="q_shade" value="1" /></td>
    <td><input type="radio" name="q_shade" value="2" /></td>
    <td><input type="radio" name="q_shade" value="3" /></td>
    <td><input type="radio" name="q_shade" value="4" /></td>
  </tr>
</table>
```

<div id="skin_cancer_specific2" class="specific-hidden">

<div style="height: 20px;"></div>

<p class="question">12. <u>After</u> you were diagnosed with skin cancer, how many times did you use a tanning bed?</p>

```
<div class="radio">
  <label><input type="radio" name="af_tanning_bed" value="0" />0</label><br>
  <label><input type="radio" name="af_tanning_bed" value="1 - 5" />1 - 5</label><br>
  <label><input type="radio" name="af_tanning_bed" value="6 - 10" />6 - 10</label><br>
  <label><input type="radio" name="af_tanning_bed" value="11 - 100" />11 -
100</label><br>
  <label><input type="radio" name="af_tanning_bed" value="&gt; 100" />greater than
100</label>
</div>
```

```

<div style="height: 6px;"></div>
<p class="question">13. <u>After</u> you were diagnosed with skin cancer, how many
blistering sunburns did you have?</p>

<div class="radio">
  <label><input type="radio" name="af_sunburn" value="0" />0</label><br>
  <label><input type="radio" name="af_sunburn" value="1 - 5" />1 - 5</label><br>
  <label><input type="radio" name="af_sunburn" value="6 - 10" />6 - 10</label><br>
  <label><input type="radio" name="af_sunburn" value=" > 10" />greater than
10</label>
</div>

<div style="height: 6px;"></div>
<p class="question">14. <u>After</u> you were diagnosed with skin cancer, how often did
you use sunscreen when you were outside?</p>

<div class="radio">
  <label><input type="radio" name="af_sunscreen" value="0" />Never</label><br>
  <label><input type="radio" name="af_sunscreen" value="1" />Rarely</label><br>
  <label><input type="radio" name="af_sunscreen" value="2" />Sometimes</label><br>
  <label><input type="radio" name="af_sunscreen" value="3" />Often</label><br>
  <label><input type="radio" name="af_sunscreen" value="4" />Always</label>
</div>

<div style="height: 6px;"></div>
<p class="question">15. <u>After</u> you were diagnosed with skin cancer, how often do
you do the following when you are outside during the summer on a warm sunny day?</p>

<table width="700px" class="qtable">
  <tr>
    <th></th>
    <th width="12%">Never</th>
    <th width="12%">Rarely</th>
    <th width="12%">Sometimes</th>
    <th width="12%">Often</th>
    <th width="12%">Always</th>
  </tr>
  <tr>
    <td>How often do you wear a shirt with sleeves that cover your shoulders?</td>
    <td><input type="radio" name="af_q_shirt" value="0" /></td>
    <td><input type="radio" name="af_q_shirt" value="1" /></td>
    <td><input type="radio" name="af_q_shirt" value="2" /></td>
    <td><input type="radio" name="af_q_shirt" value="3" /></td>
    <td><input type="radio" name="af_q_shirt" value="4" /></td>
  </tr>
  <tr>
    <td>How often do you wear a hat?</td>
    <td><input type="radio" name="af_q_hat" value="0" /></td>
    <td><input type="radio" name="af_q_hat" value="1" /></td>
    <td><input type="radio" name="af_q_hat" value="2" /></td>
    <td><input type="radio" name="af_q_hat" value="3" /></td>
    <td><input type="radio" name="af_q_hat" value="4" /></td>
  </tr>
  <tr>
    <td>How often do you stay in the shade or under an umbrella?</td>
    <td><input type="radio" name="af_q_shade" value="0" /></td>
    <td><input type="radio" name="af_q_shade" value="1" /></td>
    <td><input type="radio" name="af_q_shade" value="2" /></td>
    <td><input type="radio" name="af_q_shade" value="3" /></td>
    <td><input type="radio" name="af_q_shade" value="4" /></td>
  </tr>
</table>

```

</table>

</div>

<h3>Estimated Outdoor Exposure Time</h3>

<p>Please fill in the hours you spent outdoors between 10 AM and 4 PM per day. As you fill in the hours of exposure, a blue shading effect will be applied to the timeline below to the corresponding epoch. Fill in this section completely before moving on to the next section, as you will not be able to change your responses once you start entering your locations of residence in the next section.</p>

```
<div class="panel panel-success" style="display: inline-block;">
  <div class="panel-body">
    <svg id="timeline" height="50" width="550"></svg>
  </div>
</div>
```

```
<div id="timeapp">
  <table id="time-list" class="etable">
    <tr>
      <th style="width: 160px;">Location</th>
      <th style="width: 100px;">Age Range</th>
      <th style="width: 190px;">Weekdays (Hrs/Day)</th>
      <th style="width: 190px;">Weekends (Hrs/Day)</th>
    </tr>
  </table>
</div>
```


<h3>Exposure Location Data</h3>

<p>

Fill in the places you have primarily lived in and what age you lived there.
More spaces will appear as you enter the first three lines.

Your location responses will automatically appear next to the corresponding age ranges in the above table for your convenience.

</p>

```
<div id="locationapp">
  <table id="location-list" class="etable">
    <tr>
      <th style="width: 190px;">Location</th>
      <th style="width: 190px;">Starting Age</th>
      <th style="width: 190px;">Ending Age</th>
    </tr>
  </table>
</div>
```


<h3>Feedback</h3>

<p>Do you have any feedback about the survey?</p>

<textarea id="feedback" rows="4" style="width: 400px;"></textarea>

```

<br><br>
<a href="javascript:calculateCLUE()" class="btn btn-primary btn-lg" role="button"
id="calculate">Submit</a>

<div class="modal" id="errorModal" tabindex="-1" role="dialog">
  <div class="modal-dialog">
    <div class="modal-content">
      <div class="modal-header">
        <h4 class="modal-title" id="errorModalLabel">Couldn't submit the survey</h4>
      </div>
      <div class="modal-body" id="errorModalBody">
        ...
      </div>
      <div class="modal-footer">
        <button type="button" class="btn btn-primary" data-dismiss="modal">OK</button>
      </div>
    </div>
  </div>
</div>

<br><br>
<br><br>

<div class="panel panel-info">
  <div class="panel-heading">
    <h3 class="panel-title">Interpreting the CUES</h3>
  </div>
  <div class="panel-body">
<div class="resultsCUES" style="color:red"><b>Your personalized CUES will appear here
once you complete the questionnaire and click "Submit."</b></div>
<br>

    <p><strong>Table 1. How your CUES compares to individuals in the study. *The CUES
reported in this table have been rounded to the nearest thousand.</strong></p>
<table border="1" cellspacing="0" cellpadding="0">
  <tr>
    <td width="168"><p align="center">
      <strong>Your CUES*</strong></p></td>
    <td width="415" valign="top"><p align="center"><strong>How Your CUES Compares to
Individuals in the Study</strong></p></td>
  </tr>
  <tr>
    <td width="168"><p align="center">&lt;205,000 units</p></td>
    <td width="415" valign="top"><p align="center">Below median score for those
without history of skin cancer</p></td>
  </tr>
  <tr>
    <td width="168"><p align="center">205,001-242,000 units</p></td>
    <td width="415" valign="top"><p align="center">Above median score for those
without history of skin cancer and below the median score for those with skin
cancer</p></td>
  </tr>
  <tr>
    <td width="168"><p align="center">&gt;242,001 units</p></td>
    <td width="415" valign="top"><p align="center">Above median score for those with
skin cancer</p></td>
  </tr>
</table>

```

```

    </div>
</div>

<br><br>

<p class="page-header">&nbsp;</p>

<p>References
<ul>
</p>
<li>Glanz K, Mccarty F, Nehl EJ, et al. Validity of self-reported sunscreen use by
parents, children, and lifeguards. Am J Prev Med. 2009;36(1):63-9.</li>
    <li>Glanz K, Yaroch AL, Dancel M, et al. Measures of sun exposure and sun protection
practices for behavioral and epidemiologic research. Arch Dermatol. 2008;144(2):217-
22.</li>
</ul>

<br><br>

<script type="text/template" id="location-template">
    <td valign="top">
        <input type="text" class="location typeahead" value="<%- location %>"
placeholder="City, State" />
        <span class="glyphicon glyphicon-ok ok" style="visibility: hidden; color:
green;"></span>
        <span style="color: red;" class="location_error"></span>
    </td>
    <td valign="top">
        <input type="text" class="start_age" value="<%- start_age %>" onkeypress="return
isNumber(event)" <%= (order == 0) ? 'readonly' : '' %> />
        <span style="color: red;" class="start_age_error"></span>
    </td>
    <td valign="top">
        <input type="text" class="end_age" value="<%- end_age %>" onkeypress="return
isNumber(event)" />
        <span style="color: red;" class="end_age_error"></span>
    </td>
</script>

<script type="text/template" id="time-template">
    <% var style = showsSeparator ? 'style="border-top: 1px #dfdfdf solid;" : '' %>
    <td valign="top" <%= style %>><%= location %></td>
    <td valign="top" <%= style %>><%= start_age + ((end_age >= 1000) ? '+' : (' - ' +
end_age)) %></td>
    <td valign="top" <%= style %>>
        <input type="text" class="d_work" value="<%- d_work %>" onkeypress="return
isNumber(event)" disabled />
        <span style="color: red;" class="d_work_error"></span>
    </td>
    <td valign="top" <%= style %>>
        <input type="text" class="e_work" value="<%- e_work %>" onkeypress="return
isNumber(event)" disabled />
        <span style="color: red;" class="e_work_error"></span>
    </td>
</script>

```

```

<script type="text/template" id="location-detail-template">
  <tr>
    <td><a href="http://maps.google.com/?q=<%- coordinates %>" target="_blank"><%-
location %></a></td>
    <td><%- uvi %></td>
    <td><%- start_age %></td>
    <td><%- end_age %></td>
    <td><%= c %></td>
    <td><%= nc %></td>
  </tr>
</script>

```

```

<script type="text/template" id="time-detail-template">
  <tr>
    <td><%= start_age + ((end_age >= 1000) ? '+' : (' - ' + end_age)) %></td>
    <td><%- d_work %></td>
    <td><%- e_work %></td>
    <td><%= uve %></td>
  </tr>
</script>

```

```

<script type="text/javascript">

```

```

var selectedStartAge = -1;
var disableAgeSectionFlag = 0;

```

```

function initializeTable(){
  if (disableAgeSectionFlag == 0){
    $(".d_work").prop('disabled', false);
    $(".e_work").prop('disabled', false);
    $(".#age").prop('disabled', true);
    disableAgeSectionFlag = 1;
  }
}

```

```

var parse = function(s) {
  var tmp = parseFloat(s);
  return isNaN(tmp) ? 0 : tmp;
}

```

```

// -----
// -----
// -----

```

```

var timeRows;

```

```

var TimeRow = Backbone.Model.extend({
  defaults: function() {
    return {
      start_age: "",
      end_age: "",
      d_work: "",
      e_work: "",
      location: "",
      showsSeparator: false,
      d_work_error: "",
      e_work_error: ""
    }
  }
});

```

```

    };
  },
});

var TimeRowView = Backbone.View.extend({
  tagName: "tr",
  template: _.template($('#time-template').html()),
  events: {
    "focus .d_work": "focus",
    "focus .e_work": "focus",
    "blur .d_work": "close",
    "blur .e_work": "close",
  },
  render: function() {
    var o = this.model.toJSON();
    this.$el.html(this.template(o));
    this.d_work = this.$('.d_work');
    this.e_work = this.$('.e_work');
    return this;
  },
  render_error: function() {
    var showError = function(error, el) {
      if (error.length > 0) {
        el.html("<br>" + error);
      } else {
        el.html('');
      }
    }
    showError(this.model.get('d_work_error'), this.$('.d_work_error'));
    showError(this.model.get('e_work_error'), this.$('.e_work_error'));
  },
  initialize: function() {
    this.listenTo(this.model, 'change:d_work_error', this.render_error);
    this.listenTo(this.model, 'change:e_work_error', this.render_error);
    this.listenTo(this.model, 'destroy', this.remove);
  },
  focus: function() {
    var start_age = this.model.get('start_age');
    selectedStartAge = start_age;
    drawTimeline();
  },
  close: function() {
    this.model.set({d_work: this.d_work.val()});
    this.model.set({e_work: this.e_work.val()});
    selectedStartAge = -1;
    drawTimeline();
    validateAll(false);
  },
});

var TimeRowList = Backbone.Collection.extend({
  model: TimeRow,
});

timeRows = new TimeRowList;

var timeAppView = Backbone.View.extend({
  el: $("#timeapp"),
  initialize: function() {
    this.listenTo(timeRows, 'add', this.addOne);
    timeRows.add([

```

```

        {start_age: 0, end_age: 13},
        {start_age: 13, end_age: 20},
        {start_age: 20, end_age: 40},
        {start_age: 40, end_age: 65},
        {start_age: 65, end_age: 80},
        {start_age: 80, end_age: 1000}
    ]);
},
render: function() {
},
addOne: function(row) {
    var view = new TimeRowView({model: row});
    this.$("#time-list").append(view.render().el);
},
});

var timeApp = new timeAppView;

// -----
// -----
// -----

var rows;

var Row = Backbone.Model.extend({
    defaults: function() {
        return {
            location: "",
            //order: 0,
            start_age: "",
            end_age: "",
            resolved: false,
            location_error: "",
            start_age_error: "",
            end_age_error: ""
        };
    },
    isEmpty: function() {
        return this.get('location') == '' && this.get('start_age') == '' &&
this.get('end_age') == '';
    }
});

var citySource = function(q, cb) {
    var matches = [];
    q = q.replace(/^\s+/, '').toLowerCase();
    var str;
    for (var i = 0; i < cities.length; ++i) {
        str = cities[i];
        if (str.substring(0, q.length).toLowerCase() == q) {
            matches.push({ value: str });
            if (matches.length > 10)
                break;
        }
    }
    cb(matches);
}

var RowView = Backbone.View.extend({
    tagName: "tr",
    template: _.template($('#location-template').html()),

```

```

events: {
  "keyup .location": "updateLocation",
  "keydown .start_age": "updateStartAge",
  "keydown .end_age": "updateEndAge",
  "focus .location": "focus",
  "focus .start_age": "focus",
  "focus .end_age": "focus",
  "blur .location": "close",
  "blur .start_age": "close",
  "blur .end_age": "close",
},
render: function() {
  this.$el.html(this.template(this.model.toJSON()));
  this.location = this.$('.location');
  this.start_age = this.$('.start_age');
  this.end_age = this.$('.end_age');

  this.location.typeahead({
    hint: false,
    highlight: true,
    minLength: 1
  },
  {
    name: 'states',
    displayKey: 'value',
    source: citySource
  });

  return this;
},
render_error: function() {
  var showError = function(error, el) {
    if (error.length > 0) {
      el.html("<br>" + error);
    } else {
      el.html('');
    }
  }
  showError(this.model.get('location_error'), this.$('.location_error'));
  showError(this.model.get('start_age_error'), this.$('.start_age_error'));
  showError(this.model.get('end_age_error'), this.$('.end_age_error'));
},
render_resolved: function() {
  this.$('.ok').css('visibility', this.model.get('resolved') ? 'visible' : 'hidden');
},

initialize: function() {
  this.listenTo(this.model, 'change:start_age', this.startAgeChanged);
  this.listenTo(this.model, 'change:end_age', this.endAgeChanged);
  this.listenTo(this.model, 'change:location_error', this.render_error);
  this.listenTo(this.model, 'change:start_age_error', this.render_error);
  this.listenTo(this.model, 'change:end_age_error', this.render_error);
  this.listenTo(this.model, 'change:resolved', this.render_resolved);
  this.listenTo(this.model, 'destroy', this.remove);
},
startAgeChanged: function() {
  this.$('.start_age').val(this.model.get('start_age'));
},
endAgeChanged: function() {
  this.$('.end_age').val(this.model.get('end_age'));
},

```

```

updateLocation: function(e) {
  this.model.set({resolved: false, location_error: ''});
},
updateStartAge: function(e) {
  this.model.set({start_age_error: ''});
},
updateEndAge: function(e) {
  this.model.set({end_age_error: ''});
},
focus: function() {
  var order = this.model.get('order');
  if (order == rows.length - 1) {
    rows.add([{location: "", order: rows.length}]);
  }

  while (rows.models.length > order + 2 && rows.last().isEmpty() &&
    rows.models[rows.models.length - 2].isEmpty() && rows.models.length > 3) {
    rows.models[rows.models.length - 1].destroy();
  }
},
close: function() {
  this.model.set({
    location: this.location.val(),
    start_age: this.start_age.val(),
    end_age: this.end_age.val()
  });

  var start_age = this.start_age.val();
  var end_age = this.end_age.val();
  var order = this.model.get('order');
  var age = parse($("#age").val());
  if (order > 0) {
    if (age == 0 || start_age != '' || (age > 0 && rows.models[order -
1].get('end_age') < age))
      rows.models[order - 1].set({end_age: start_age});
  }
  if (order < rows.models.length - 1) {
    if (age == 0 || (age > 0 && end_age < age)) {
      rows.models[order + 1].set({start_age: end_age});
    } else if (age > 0 && end_age >= age) {
      rows.models[order + 1].set({start_age: ""});
    }
  }

  validateAll(false);
  updateTime();

  // TODO: purge rows
},
});

var RowList = Backbone.Collection.extend({
  model: Row,
});

rows = new RowList;

var LocationAppView = Backbone.View.extend({
  el: $("#locationapp"),
  initialize: function() {
    this.listenTo(rows, 'add', this.addOne);
  }
});

```

```

        rows.add([
            {order: 0, location: "", start_age: 0},
            {order: 1, location: ""},
            {order: 2, location: ""}
        ]);
    },
    render: function() {
    },
    addOne: function(row) {
        var view = new RowView({model: row});
        this.$("#location-list").append(view.render().el);
    },
});

var locationApp = new LocationAppView;

// -----
// QUESTIONS & QUESTION VALIDATION -----
// -----

function updateQuestions() {

    if ($("#input[name=family_skin_cancer]:checked").val() == 'yes') {
        $('input[name=family_skin_cancer_type]').removeAttr('disabled');
        $('#family_skin_cancer_specific').removeClass('specific-disabled');
    } else {
        $('input[name=family_skin_cancer_type]').attr('disabled', 'disabled');
        $('#family_skin_cancer_specific').addClass('specific-disabled');

        $('input[name=family_skin_cancer_type]').prop('checked', false);
    }

    if ($("#input[name=skin_cancer]:checked").val() == 'yes') {
        $('input[name=skin_cancer_age]').removeAttr('disabled');
        $('input[name=skin_cancer_type]').removeAttr('disabled');
        $('#skin_cancer_specific').removeClass('specific-disabled');
        $('#skin_cancer_specific2').removeClass('specific-hidden');
        //$('#feedback_number').text('14');
        // FIXME: Don't repeat yourself
        $('#question_tanning_bed').html('8. <u>Before</u> you were diagnosed with skin cancer, how many times did you use a tanning bed?');
        $('#question_sunburn').html('9. <u>Before</u> you were diagnosed with skin cancer, how many blistering sunburns did you have?');
        $('#question_sunscreen').html('10. <u>Before</u> you were diagnosed with skin cancer, how often did you use sunscreen when you were outside?');
        $('#question9').html('11. <u>Before</u> you were diagnosed with skin cancer, how often did you do the following when you were outside during the summer on a warm sunny day?</p>');
        $('.v_do').text('did');
        $('.v_cover').text('covered');
    } else {
        $('input[name=skin_cancer_age]').attr('disabled', 'disabled');
        $('input[name=skin_cancer_type]').attr('disabled', 'disabled');
        $('#skin_cancer_specific').addClass('specific-disabled');
        $('#skin_cancer_specific2').addClass('specific-hidden');
        //$('#feedback_number').text('10');
        // FIXME: Don't repeat yourself
        $('#question_tanning_bed').html('8. How many times have you used a tanning bed in your life?');
    }
}

```



```

    skin_type: [2, ''],
    family_skin_cancer: [3, ''],
    family_skin_cancer_types: [4, 'family_skin_cancer'],
    skin_cancer: [5, ''],
    skin_cancer_age: [6, 'skin_cancer'],
    skin_cancer_type: [7, 'skin_cancer'],
    tanning_bed: [8, ''],
    sunburn: [9, ''],
    sunscreen: [10, ''],
    q_shirt: [11, ''],
    q_hat: [11, ''],
    q_shade: [11, ''],
    af_tanning_bed: [12, 'skin_cancer'],
    af_sunburn: [13, 'skin_cancer'],
    af_sunscreen: [14, 'skin_cancer'],
    af_q_shirt: [15, 'skin_cancer'],
    af_q_hat: [15, 'skin_cancer'],
    af_q_shade: [15, 'skin_cancer']
  }
  var problems = [];
  for (var k in rules) {
    var unacceptableAnswer = (k == 'age' || k == 'skin_cancer_age') ? '' : undefined;
    if (questionData[k] === unacceptableAnswer) {
      var dependent = rules[k][1];
      if (dependent != '' && questionData[dependent] != 'yes')
        continue;
      problems.push(rules[k][0]);
    }
  }
  if (problems.length > 0) {
    problems = _.uniq(problems);
    messages.push('Please answer all the questions in the survey. ' +
      "(You haven't answered or completed question" + ((problems.length == 1) ? '' :
's') +
      ' #' + problems.join(', ') + ".)");
    success = false;
  }
}

// validate age
var age = parseFloat($("#input[name=age]").val());
var skin_cancer_age = parseFloat($("#input[name=skin_cancer_age]").val());
if (!isNaN(age) && !isNaN(skin_cancer_age) && age < skin_cancer_age) {
  $('#skin_cancer_age_error').text('This cannot be greater than your age.');
```

messages.push('The age you were diagnosed with skin cancer cannot be greater than your age.');

```

  success = false;
} else {
  $('#skin_cancer_age_error').text('');
}

// validate location
var locationErrorMessage = "Please fix the error in the Exposure Location Data section.";
for (var i = 0; i < rows.models.length; ++i) {
  model = rows.models[i];
  if (show) model.set({start_age_error: ''});
  if (show) model.set({end_age_error: ''});

  if (model.isEmpty()) continue;

```

```

var x = parseFloat(model.get('start_age'));
var y = parseFloat(model.get('end_age'));

if (thorough) {
  if (model.get('location').length == 0) {
    if (show) model.set({location_error: "Can't be blank"});
    messages.push(locationErrorMessage);
    success = false;
  }
  if (model.get('start_age').length == 0) {
    if (show) model.set({start_age_error: "Can't be blank"});
    messages.push(locationErrorMessage);
    success = false;
  }
  if (model.get('end_age').length == 0) {
    if (show) model.set({end_age_error: "Can't be blank"});
    messages.push(locationErrorMessage);
    success = false;
  }
}

if (!isNaN(x) && !isNaN(y) && x >= y) {
  if (show) model.set({start_age_error: ' '});
  if (show) model.set({end_age_error: 'Must be greater than ' + x});
  messages.push(locationErrorMessage);
  success = false;
}

if (prevModel) {
  var y0 = parseFloat(prevModel.get('end_age'));
  if (!isNaN(x) && !isNaN(y0) && x < y0) {
    if (show) model.set({start_age_error: 'Must be at least ' + y0});
    messages.push(locationErrorMessage);
    success = false;
  }
}
prevModel = model;
}

// validate time
var timeErrorMessage = "Please fix the error in the Estimated Outdoor Exposure Time
section.";
for (var i = 0; i < timeRows.models.length; ++i) {
  model = timeRows.models[i];
  if (show) model.set({d_work_error: ''});
  if (show) model.set({e_work_error: ''});

  if (thorough) {
    if (model.get('d_work').length == 0) {
      if (show) model.set({d_work_error: "Can't be blank"});
      messages.push(timeErrorMessage);
      success = false;
    }
    if (model.get('e_work').length == 0) {
      if (show) model.set({e_work_error: "Can't be blank"});
      messages.push(timeErrorMessage);
      success = false;
    }
  }
}

var d_work = parseFloat(model.get('d_work'));

```

```

    var e_work = parseFloat(model.get('e_work'));

    if (!isNaN(d_work) && d_work > 6) {
        if (show) model.set({d_work_error: 'Must not be greater than 6.<br>Please fill the
hours between<br>10 AM - 4 PM.'});
        messages.push(timeErrorMessage);
        success = false;
    }
    if (!isNaN(e_work) && e_work > 6) {
        if (show) model.set({e_work_error: 'Must not be greater than 6.<br>Please fill the
hours between<br>10 AM - 4 PM.'});
        messages.push(timeErrorMessage);
        success = false;
    }
}

messages = _.uniq(messages);
return {ok: success, messages: messages};
}

function okToUpdateTime() {
    var success = true;
    var model, prevModel = null;

    // validate location
    for (var i = 0; i < rows.models.length; ++i) {
        model = rows.models[i];
        if (model.isEmpty()) continue;
        var x = parseFloat(model.get('start_age'));
        var y = parseFloat(model.get('end_age'));
        if (!isNaN(x) && !isNaN(y) && x >= y) {
            success = false;
        }
        if (prevModel) {
            var y0 = parseFloat(prevModel.get('end_age'));
            if (!isNaN(x) && !isNaN(y0) && x < y0) {
                success = false;
            }
        }
        prevModel = model;
    }

    return success;
}

// TODO: can't copy & paste in Firefox
function isNumber(evt) {
    evt = evt ? evt : window.event;
    var target = $(evt.target);
    target.tooltip({
        container: 'body',
        html: 'true',
        title: '<span style="font-size: 14px;">Must be a number</span>',
        trigger: 'manual',
    });
}

var charCode = (evt.which) ? evt.which : evt.keyCode; // 46 = dot
if (charCode > 31 &&
    (
        ((charCode < 48 || charCode > 57) && charCode != 46) ||
        (charCode == 46 && target.val().indexOf('.') != -1)
    )

```

```

    )
    ) {
    target.tooltip('show');
    setTimeout(function() {
        target.tooltip('hide');
    }, 1500);
    return false;
}
return true;
}

// -----
// DYNAMIC -----
// -----

$('#age').change(function() {
    updateTime();
    validateAll(false);
});

$('#skin_cancer_age').blur(function() {
    updateTime();
    validateAll(false);
});

function clone(o) {
    return JSON.parse(JSON.stringify(o)); // TODO: hacky
}

// create a JSON object for calculation
function getTimeData() {
    var timeData = [], model, o;
    for (var i = 0; i < timeRows.models.length; ++i) {
        model = timeRows.models[i];
        o = model.toJSON();
        o.d_work = parse(o.d_work);
        o.e_work = parse(o.e_work);
        timeData.push(o);
    }
    // for (var i = 0; i < timeData.length - 1; ++i) {
    //     o = timeData[i];
    //     p = timeData[i + 1];
    //     if (o.end_age < p.start_age)
    //         o.end_age++;
    // }
    return timeData;
}

// create a JSON object for calculation
function getLocationData() {
    var locationData = [], model, o, p;
    var age = parse($('#age').val());
    for (var i = 0; i < rows.models.length; ++i) {
        model = rows.models[i];
        if (model.isEmpty()) continue;
        o = model.toJSON();
        if (o.start_age.length == 0) continue;
        if (o.end_age.length == 0) continue;
        o.start_age = parse(o.start_age);

```

```

    o.end_age = parse(o.end_age);
    locationData.push(o);
    if (age > 0 && o.end_age > age) {
        o.end_age = age;
        break;
    }
}

// fill the gap
for (var i = 0; i < locationData.length - 1; ++i) {
    o = locationData[i];
    p = locationData[i + 1];
    //if (o.end_age + 1 == p.start_age) {
    //    o.end_age++;
    //} else if (o.end_age + 1 < p.start_age) { // TODO: shouldn't have to check this!
    if (o.end_age < p.start_age) {
        locationData.splice(i+1, 0, {
            location: '',
            coordinates: [0, 0],
            uvi: 0,
            start_age: o.end_age,
            end_age: p.start_age,
        });
        i++;
    }
}
return locationData;
}

function updateTime() {
    if (!okToUpdateTime())
        return;

    var model, o, p, i, j;

    //var timeData = getTimeData();
    var timeData = [];
    for (var i = 0; i < timeRows.models.length; ++i) {
        model = timeRows.models[i];
        o = model.toJSON();
        //o.d_work = parse(o.d_work);
        //o.e_work = parse(o.e_work);
        timeData.push(o);
    }
    var locationData = getLocationData();

    var newTimeData = [
        {start_age: 0, end_age: 13},
        {start_age: 13, end_age: 20},
        {start_age: 20, end_age: 40},
        {start_age: 40, end_age: 65},
        {start_age: 65, end_age: 80},
        {start_age: 80, end_age: 1000}
    ];

    var age = parse($("#age").val());
    if (age > 0) {
        if (age > 200) age = 200; // TODO: ???
        while (newTimeData[newTimeData.length-1].start_age >= age) {
            newTimeData.pop();
        }
    }
}

```

```

    newTimeData[newTimeData.length-1].end_age = age;
}

// gather breakpoints
var bps = [];
for (var i = 0; i < locationData.length; ++i) {
    o = locationData[i];
    bps.push(o.start_age);
    bps.push(o.end_age);
}
var skin_cancer_age = parse($("#input[name=skin_cancer_age]").val());
if (skin_cancer_age > 0)
    bps.push(skin_cancer_age);
bps = _.uniq(bps);
bps.sort(function(a, b) { return a - b; });

// splits newTimeData along breakpoints;
i = 0;
j = 0;
while (i < newTimeData.length && j < bps.length) {
    o = newTimeData[i];
    if (o.end_age <= bps[j]) {
        i++;
    } else if (o.start_age >= bps[j]) {
        j++;
    } else {
        p = clone(o);
        p.end_age = bps[j];
        o.start_age = p.end_age;
        newTimeData.splice(i, 0, p);
    }
}

// restore data from timeData
var ds = new Array(200), es = new Array(200);
for (i = 0; i < 200; ++i) {
    ds[i] = 0;
    es[i] = 0;
}
for (i = 0; i < timeData.length; ++i) {
    var start_age = timeData[i].start_age;
    var end_age = Math.min(timeData[i].end_age, 199);
    var d_work = timeData[i].d_work;
    var e_work = timeData[i].e_work;
    for (j = start_age; j < end_age; ++j) {
        ds[j] = d_work;
        es[j] = e_work;
    }
}
for (i = 0; i < newTimeData.length; ++i) {
    var start_age = newTimeData[i].start_age;
    var end_age = Math.min(newTimeData[i].end_age, 199);
    var d_work = ds[start_age];
    var same = true;
    for (j = start_age + 1; j < end_age; ++j) {
        if (ds[j] != d_work) {
            same = false;
            break;
        }
    }
}

```

```

    if (same)
        newTimeData[i].d_work = d_work;
}
for (i = 0; i < newTimeData.length; ++i) {
    var start_age = newTimeData[i].start_age;
    var end_age = Math.min(newTimeData[i].end_age, 199);
    var e_work = es[start_age];
    var same = true;
    for (j = start_age + 1; j < end_age; ++j) {
        if (es[j] != e_work) {
            same = false;
            break;
        }
    }
    if (same)
        newTimeData[i].e_work = e_work;
}

// assign locations
j = 0;
for (i = 0; i < locationData.length; ++i) {
    o = locationData[i];
    while (j < newTimeData.length &&
        (p = newTimeData[j], (p.start_age < o.end_age))) {
        if (o.start_age < p.end_age)
            newTimeData[j].location = o.location;
        j++;
    }
}

// clean up location fields
for (i = newTimeData.length - 1; i > 0; --i) {
    o = newTimeData[i];
    p = newTimeData[i - 1];
    if (o.location == p.location) {
        o.location = "";
    } else {
        o.showsSeparator = true;
    }
}

// set new time data
while (timeRows.models.length) {
    timeRows.models[timeRows.models.length-1].destroy();
}
timeRows.add(newTimeData);

// redraw time line
drawTimeline();
}

var timeline = SVG('timeline');
if (timeline)
    timeline.size(620, 24);

function drawTimeline() {
    if (!timeline)
        return;
    var timeData = getTimeData();
    timeline.clear();

```

```

var age = parse($("#age").val());
if (age == 0)
    age = 85;

//var rect = timeline.rect(100, 100).attr({ fill: '#f06' })
var o, i, x;
var x0 = 10;
var xScale = (timeline.width() - x0 - 10) / age;
var y = 8;

    timeline.line(x0, y, x0 + age * xScale, y).attr({stroke: '#ccc', 'shape-rendering':
'crispEdges'});
    var drawTick = function (age) {
        var x = x0 + age * xScale;
        timeline.text(age + "").attr({
            x: x,
            y: y-1
        }).font({
            anchor: 'middle',
            fill: '#333',
            size: '13px'
        });
        timeline.line(x, y-5, x, y+5).attr({stroke: '#aaa', 'shape-rendering':
'crispEdges'});
    }
    for (i = 0; i < timeData.length; ++i) {
        o = timeData[i];
        if (i == 0)
            drawTick(o.start_age);
            drawTick(o.end_age);

            if (o.start_age == selectedStartAge) {
                timeline.line(x0 + o.start_age * xScale, y, x0 + o.end_age * xScale,
y).attr({stroke: '#5bc0de', 'stroke-width': 6});
            }
        }
    }

// -----
// -----
// -----

var markers = [];

function calculateCLUE() {
    var validateResult = validateAll(true);
    if (!validateResult.ok) {
        alert(validateResult.messages.join('\n\n'));
        return;
    }

    $('#calculate').addClass('disabled');

    var nRequests = 0;
    var nUnsuccessfulRequests = 0;
    var requests = [];

    var model, o, p;

```

```

var unresolvedModels = [];
for (var i = 0; i < rows.models.length; ++i) {
    model = rows.models[i];
    if (model.isEmpty()) continue;
    if (!model.get('resolved')) {
        nRequests++;
        unresolvedModels.push(model);
    }
}

var done = function () {

    if (nUnsuccessfulRequests) {
        alert("Can't find all the locations.");
        $('#calculate').removeClass('disabled');
        return;
    }

    var parse = function(s) {
        var tmp = parseFloat(s);
        return isNaN(tmp) ? 0 : tmp;
    }

    var timeData = getTimeData();
    var locationData = getLocationData();

    for (var i = 0; i < timeData.length; ++i) {
        o = timeData[i];
        o.uve = 52 * (5 * o.d_work + 2 * o.e_work);
    }

    var cleanAndCalculate = function(locationData, timeData) {
        // This function calculates C and NC scores and
        // also splits locationData along timeData's boundaries.
        var i = 0, j = 0;
        var o, p;
        while (i < locationData.length) {
            o = locationData[i];
            // FIXME: error if j >= timeData.length
            if (o.end_age <= timeData[j].end_age) {
                o.nc = (o.uvi == 0) ? 0 : (timeData[j].uve * (o.end_age - o.start_age));
                o.c = o.nc * o.uvi;
                i++;
            } else {
                if (o.start_age < timeData[j].end_age) {
                    p = clone(o);
                    p.end_age = timeData[j].end_age;
                    o.start_age = p.end_age;
                    locationData.splice(i, 0, p);
                } else {
                    j++;
                }
            }
        }
    }

    // calculate
    var originalLocationData = clone(locationData);
    cleanAndCalculate(locationData, timeData);

    // calculate C and NC for people who had skin cancer

```

```

var totalCSkinCancer = 0, totalNCSkinCancer = 0;
if ($("#input[name=skin_cancer]:checked").val() == 'yes' && locationData.length) {
    var skin_cancer_age = parse($("#input[name=skin_cancer_age]").val());
    if (skin_cancer_age > 0) {
        var locationDataSkinCancer = clone(originalLocationData);
        for (var i = locationDataSkinCancer.length - 1; i >= 0; --i) {
            o = locationDataSkinCancer[i];
            if (o.start_age > skin_cancer_age) {
                locationDataSkinCancer.pop();
            } else {
                o.end_age = Math.min(skin_cancer_age, o.end_age);
                break;
            }
        }
        cleanAndCalculate(locationDataSkinCancer, timeData);
        for (var i = 0; i < locationDataSkinCancer.length; ++i) {
            o = locationDataSkinCancer[i];
            totalCSkinCancer += o.c;
            totalNCSkinCancer += o.nc;
        }
    }
}

var totalC = 0, totalNC = 0;
for (var i = 0; i < locationData.length; ++i) {
    o = locationData[i];
    totalC += o.c;
    totalNC += o.nc;
}
//$('#total-c').text(addCommaToNumber(Math.round(totalC)));
//$('#total-nc').text(addCommaToNumber(totalNC));

// for saving purpose
j = 0;
for (var i = 0; i < timeData.length; ++i) {
    o = timeData[i];
    o.nc = 0;
    o.c = 0;
    while (j < locationData.length && locationData[j].end_age <= o.end_age) {
        o.nc += locationData[j].nc;
        o.c += locationData[j].c;
        j++;
    }
}

// TODO: use getQuestionData() and $.extend()
var data = {
    age: $("#age").val(),
    skin_type: $("#input[name=skin_type]:checked").val(),
    family_skin_cancer: $("#input[name=family_skin_cancer]:checked").val(),
    family_skin_cancer_types:
    $("#input[name=family_skin_cancer_type]:checked").map(function(_, el) { return
    $(el).val(); }).get(),
    skin_cancer: $("#input[name=skin_cancer]:checked").val(),
    skin_cancer_age: $("#input[name=skin_cancer_age]").val(),
    skin_cancer_type: $("#input[name=skin_cancer_type]:checked").val(),
    tanning_bed: $("#input[name=tanning_bed]:checked").val(),
    sunburn: $("#input[name=sunburn]:checked").val(),
    sunscreen: $("#input[name=sunscreen]:checked").val(),
    q_shirt: $("#input[name=q_shirt]:checked").val(),

```

```

q_hat: $("input[name=q_hat]:checked").val(),
q_shade: $("input[name=q_shade]:checked").val(),
af_tanning_bed: $("input[name=af_tanning_bed]:checked").val(),
af_sunburn: $("input[name=af_sunburn]:checked").val(),
af_sunscreen: $("input[name=af_sunscreen]:checked").val(),
af_q_shirt: $("input[name=af_q_shirt]:checked").val(),
af_q_hat: $("input[name=af_q_hat]:checked").val(),
af_q_shade: $("input[name=af_q_shade]:checked").val(),
time_data: timeData,
location_data: originalLocationData,
debug_location_data: locationData,
total_c: totalC,
total_nc: totalNC,
total_c_skin_cancer: totalCSkinCancer,
total_nc_skin_cancer: totalNCSkinCancer,
feedback: $("#feedback").val(),
};

alert('Your CUES is ' + Math.round(totalC/1000)*1000);
$('.resultsCUES').html('<b>Your CUES is: ' + Math.round(totalC/1000)*1000 + '.');

/*
$.ajax({
  url: "/home/save_data",
  method: 'post',
  data: {data: JSON.stringify(data)},
  success: function (o) {
    window.location.href = '/home/thanks';
  },
  error: function (jqXHR, textStatus, errorThrown) {
    alert(errorThrown);
    $('#calculate').removeClass('disabled');
  }
});
*/
};

if (nRequests == 0) {
  done();
} else {
  $.each(unresolvedModels, function(i, model) {
    geocode(model.get('location'), function(success, uvi, coordinates) {
      nRequests--;
      if (success) {
        model.set({resolved: true, uvi: uvi, coordinates: coordinates});
      } else {
        model.set({location_error: "Can't find \"" + model.get('location') + "\"});
        nUnsuccessfulRequests++;
      }
      if (nRequests == 0) {
        done();
      }
    });
  });
}
}

var geocoder = new google.maps.Geocoder();

function geocode(address, callback) {
  geocoder.geocode( {'address': address}, function(results, status) {

```

```

    if (status == google.maps.GeocoderStatus.OK) {
        var result = results[0];
        var location = result.geometry.location;
        var uvi = 0;

        var address_component;
        for (var i = 0; i < result.address_components.length; ++i) {
            address_component = result.address_components[i];
            if ($.inArray('country', address_component.types) != -1 &&
address_component.short_name == 'US') {
                var closestIndex = findClosestAnchor(location);
                uvi = anchors[closestIndex].uvi;
                break;
            }
        }
        callback(true, uvi, [location.lat(), location.lng()]);
    } else {
        console.log('Geocode was not successful for the following reason: ' + status);
        callback(false);
    }
});
}

function toggleDetail() {
    $('#detail').toggle();
    $('#toggle-detail').text($('#detail').is(":visible") ? 'Hide calculation details' :
'Show calculation details');
}

function addCommaToNumber(val){
    while (/(\d+)(\d{3})/.test(val.toString())) {
        val = val.toString().replace(/(\d+)(\d{3})/, '$1'+','+'$2');
    }
    return val;
}

// -----
-----

var anchors = [];

function anchor (name, place, uvi, uvisd){
    this.name = name;
    this.place = place;
    this.uvi = uvi;
    this.uvisd = uvisd;
}

function initialize() {
    if (typeof(Number.prototype.toRad) == "undefined") {
        Number.prototype.toRad = function() {
            return this * Math.PI / 180;
        }
    }
    anchors[0] = new anchor("Anchorage, AK", new google.maps.LatLng(61.2180556, -
149.90027780000003), 1.8771353446377, 0.0941944688374116);
    anchors[1] = new anchor("Mobile, AL", new google.maps.LatLng(30.6943566, -88.0430541),
6.99169760633009, 0.361432294330915);
    anchors[2] = new anchor("Little Rock, AR", new google.maps.LatLng(34.7464809, -
92.28959479999997), 5.68693868312647, 0.355749024951511);

```

```

anchors[3] = new anchor("Phoenix, AZ", new google.maps.LatLng(33.4483771, -
112.07403729999999), 6.93812541385763, 0.344929966758139);
anchors[4] = new anchor("Los Angeles, CA", new google.maps.LatLng(34.0522342, -
118.2436849), 6.9393429783746, 0.243801093659775);
anchors[5] = new anchor("San Francisco, CA", new google.maps.LatLng(37.7749295, -
122.41941550000001), 5.69740176712299, 0.262269072636097);
anchors[6] = new anchor("Denver, CO", new google.maps.LatLng(39.737567, -
104.98471790000002), 5.89226340757978, 0.256272517460823);
anchors[7] = new anchor("Hartford, CT", new google.maps.LatLng(41.76371109999999, -
72.68509319999998), 4.22672813838915, 0.203738763364689);
anchors[8] = new anchor("Washington, DC", new google.maps.LatLng(38.90723089999999, -
77.03646409999999), 4.71070047932166, 0.249665467913306);
anchors[9] = new anchor("Dover, DE", new google.maps.LatLng(39.158168, -
75.52436820000003), 4.5991097619854, 0.311636931010691);
anchors[10] = new anchor("Jacksonville, FL", new google.maps.LatLng(30.3321838, -
81.65565099999998), 7.09401420015119, 0.214101831034288);
anchors[11] = new anchor("Miami, FL", new google.maps.LatLng(25.7889689, -
80.22643929999998), 8.49667371888722, 0.166440998707845);
anchors[12] = new anchor("Tampa, FL", new google.maps.LatLng(27.950575, -
82.45717760000002), 7.76572074393997, 0.229518020912197);
anchors[13] = new anchor("Atlanta, GA", new google.maps.LatLng(33.7489954, -
84.3879824), 5.98641447813521, 0.425791266524952);
anchors[14] = new anchor("Honolulu, HI", new google.maps.LatLng(21.3069444, -
157.85833330000003), 9.37775280498836, 0.129399577036528);
anchors[15] = new anchor("Des Moines, IA", new google.maps.LatLng(41.6005448, -
93.60910639999997), 4.40568787948717, 0.147267152479771);
anchors[16] = new anchor("Boise, ID", new google.maps.LatLng(43.6187102, -
116.21460680000001), 4.78647280238093, 0.258540033772576);
anchors[17] = new anchor("Chicago, IL", new google.maps.LatLng(41.8781136, -
87.62979819999998), 4.23987074547039, 0.281434514296989);
anchors[18] = new anchor("Indianapolis, IN", new google.maps.LatLng(39.768403, -
86.15806800000001), 4.6062219376049, 0.306086245149201);
anchors[19] = new anchor("Wichita, KS", new google.maps.LatLng(37.68888889999999, -
97.33611109999998), 5.21848755746084, 0.222250439900723);
anchors[20] = new anchor("Louisville, KY", new google.maps.LatLng(38.2526647, -
85.75845570000001), 4.83281578196086, 0.302247966323691);
anchors[21] = new anchor("New Orleans, LA", new google.maps.LatLng(29.95106579999999, -
90.0715323), 7.34517383984514, 0.192206003452818);
anchors[22] = new anchor("Boston, MA", new google.maps.LatLng(42.3584308, -71.0597732),
4.21412873959406, 0.212939946089521);
anchors[23] = new anchor("Baltimore, MD", new google.maps.LatLng(39.2903848, -
76.61218930000001), 4.62674081014582, 0.25041388660887);
anchors[24] = new anchor("Portland, ME", new google.maps.LatLng(43.66147100000001, -
70.2553259), 3.83323922076223, 0.167681547205218);
anchors[25] = new anchor("Detroit, MI", new google.maps.LatLng(42.331427, -83.0457538),
4.04100135289314, 0.253487084710969);
anchors[26] = new anchor("Minneapolis, MN", new google.maps.LatLng(44.983334, -
93.26666999999998), 3.85650039932282, 0.107660787787273);
anchors[27] = new anchor("St. Louis, MO", new google.maps.LatLng(38.6270025, -
90.1994042), 4.91900270365961, 0.268721261055546);
anchors[28] = new anchor("Jackson, MS", new google.maps.LatLng(32.2987573, -
90.18481029999998), 6.46150532422559, 0.290171399185667);
anchors[29] = new anchor("Billings, MT", new google.maps.LatLng(45.7832856, -
108.5006904), 4.39514553786162, 0.186618610616946);
anchors[30] = new anchor("Raleigh, NC", new google.maps.LatLng(35.7795897, -
78.63817870000003), 5.54510015374996, 0.27886413330851);
anchors[31] = new anchor("Bismarck, ND", new google.maps.LatLng(46.8083268, -
100.78373920000001), 3.9111812684323, 0.199698339508765);
anchors[32] = new anchor("Omaha, NE", new google.maps.LatLng(41.2523634, -
95.99798829999997), 4.53869076728424, 0.190962100567503);

```

```

    anchors[33] = new anchor("Concord, NH", new google.maps.LatLng(43.2081366, -
71.537571800000002), 3.97383669658251, 0.194861593555015);
    anchors[34] = new anchor("Atlantic City, NJ", new google.maps.LatLng(39.3642834, -
74.422926599999998), 4.63895384418092, 0.319177873857818);
    anchors[35] = new anchor("Albuquerque, NM", new google.maps.LatLng(35.110703, -
106.609990999999998), 7.06754535400047, 0.406758951302654);
    anchors[36] = new anchor("Las Vegas, NV", new google.maps.LatLng(36.114646, -
115.172816000000001), 6.2806703849543, 0.232346040418651);
    anchors[37] = new anchor("Buffalo, NY", new google.maps.LatLng(42.886446799999999, -
78.8783689), 4.01281893094337, 0.242336109545002);
    anchors[38] = new anchor("New York, NY", new google.maps.LatLng(40.7143528, -
74.0059731), 4.4304686479075, 0.254456871969045);
    anchors[39] = new anchor("Cleveland, OH", new google.maps.LatLng(41.4994954, -
81.6954088), 4.2406486791056, 0.287714008691697);
    anchors[40] = new anchor("Oklahoma City, OK", new google.maps.LatLng(35.4675602, -
97.516427599999999), 5.64239354734025, 0.245035990121384);
    anchors[41] = new anchor("Portland, OR", new google.maps.LatLng(45.5234515, -
122.6762071), 3.55015852020372, 0.242493040092435);
    anchors[42] = new anchor("Philadelphia, PA", new google.maps.LatLng(39.952335, -
75.163789000000001), 4.41173721008709, 0.257418967294819);
    anchors[43] = new anchor("Pittsburgh, PA", new google.maps.LatLng(40.440624799999999, -
79.995886400000002), 4.25916065609887, 0.291240072931624);
    anchors[44] = new anchor("San Juan, PR", new google.maps.LatLng(18.4663338, -
66.1057217), 10.2888297136866, 0.129747253692345);
    anchors[45] = new anchor("Providence, RI", new google.maps.LatLng(41.8239891, -
71.412834299999999), 4.25545103210342, 0.221735674527966);
    anchors[46] = new anchor("Charleston, SC", new google.maps.LatLng(32.7765656, -
79.930921599999998), 6.35264708202032, 0.309244037662816);
    anchors[47] = new anchor("Sioux Falls, SD", new google.maps.LatLng(43.5499749, -
96.700327000000002), 4.17895327721649, 0.17862394140522);
    anchors[48] = new anchor("Memphis, TN", new google.maps.LatLng(35.1495343, -
90.0489801), 5.60720237401306, 0.272849975711269);
    anchors[49] = new anchor("Dallas, TX", new google.maps.LatLng(32.7801399, -
96.800451099999998), 6.02434279538013, 0.216509390235739);
    anchors[50] = new anchor("Houston, TX", new google.maps.LatLng(29.7601927, -
95.369389599999998), 6.90539348846443, 0.201026025806136);
    anchors[51] = new anchor("Salt Lake City, UT", new google.maps.LatLng(40.7607793, -
111.891047399999999), 5.72239076420379, 0.27183043134687);
    anchors[52] = new anchor("Norfolk, VA", new google.maps.LatLng(36.8507689, -
76.2858726), 5.37551979739632, 0.299473361294144);
    anchors[53] = new anchor("Burlington, VT", new google.maps.LatLng(44.4758825, -
73.212071999999998), 3.6274031441389, 0.315969938687122);
    anchors[54] = new anchor("Seattle, WA", new google.maps.LatLng(47.6062095, -
122.3320708), 3.37310198562014, 0.203350293515585);
    anchors[55] = new anchor("Milwaukee, WI", new google.maps.LatLng(43.0389025, -
87.906473600000003), 4.08636668364931, 0.245208533061033);
    anchors[56] = new anchor("Charleston, WV", new google.maps.LatLng(38.3498195, -
81.6326234), 4.68838477691702, 0.266749990771736);
    anchors[57] = new anchor("Cheyenne, WY", new google.maps.LatLng(41.1399814, -
104.820246199999999), 5.50418998673153, 0.328838871660807);

    drawTimeline();
}

initialize();

function findClosestAnchor(location) {
    var closestIndex = -1;
    var distance;
    var closestDistance = Infinity;

```

```

    for (var i = 0; i < anchors.length; i++){
        distance = calcHaversine(anchors[i].place, location);
        if (distance <= closestDistance) {
            closestIndex = i;
            closestDistance = distance;
        }
    }

    return closestIndex;
}

function calcHaversine(latlng1, latlng2){
    //http://www.movable-type.co.uk/scripts/latlong.html

    var lat1 = latlng1.lat();
    var lon1 = latlng1.lng();
    var lat2 = latlng2.lat();
    var lon2 = latlng2.lng();
    var R = 6371; // km
    var dLat = (lat2-lat1).toRad();
    var dLon = (lon2-lon1).toRad();
    var lat1 = lat1.toRad();
    var lat2 = lat2.toRad();
    var a = Math.sin(dLat/2) * Math.sin(dLat/2) + Math.sin(dLon/2) * Math.sin(dLon/2) *
Math.cos(lat1) * Math.cos(lat2);
    var c = 2 * Math.atan2(Math.sqrt(a), Math.sqrt(1-a));
    var d = R * c;
    return d;
}
</script>

</div>

<script src="js/bootstrap.min.js"></script>

</body>
</html>

```

jquery-1.11.3.min.js

jQuery is available for public use under the MIT license and can be found at <https://www.jquery.com>.

underscore-min.js

underscore.js is available for public use under the MIT license and can be found at <http://underscorejs.org/>.

backbone-min.js

backbone.js is available for public use under the MIT license and can be found at <http://backbonejs.org/>.

typeahead.jquery.js

typeahead.js is available for public use under the MIT license and can be found at <https://twitter.github.io/typeahead.js/>.

```

var cities = ["Akhiok, AK", "Akiachak, AK", "Akiak, AK", "Akutan, AK",
  "Alakanuk, AK", "Aleknagik, AK", "Allakaket, AK", "Ambler, AK",
  "Anaktuvuk Pass, AK", "Anchorage, AK", "Anderson, AK", "Angoon, AK",
  "Anvik, AK", "Arctic Village, AK", "Atmautluak, AK", "Barrow, AK",
  "Beaver, AK", "Bettles Field, AK", "Border, AK", "Brevig Mission, AK",
  "Buckland, AK", "Butte, AK", "Cantwell, AK", "Central, AK",
  "Chalkyitsik, AK", "Chefornak, AK", "Chenega Bay, AK", "Chevak, AK",
  "Chignik, AK", "Chignik Lagoon, AK", "Chuathbaluk, AK", "Chugiak, AK",
  "Circle, AK", "Clam Gulch, AK", "Clarks Point, AK", "Clear, AK",
  "Coldfoot, AK", "Cooper Landing, AK", "Copper Center, AK", "Craig, AK",
  "Crooked Creek, AK", "Deering, AK", "Denali National, AK",
  "Dot Lake, AK", "Douglas, AK", "Dutch Harbor, AK", "Eagle River, AK",
  "Eek, AK", "Egegik, AK", "Eielson Afb, AK", "Ekwok, AK", "Elim, AK",
  "Elmendorf Afb, AK", "Emmonak, AK", "Fairbanks, AK", "False Pass, AK",
  "Fort Richardson, AK", "Fort Wainwright, AK", "Fort Yukon, AK",
  "Galena, AK", "Gambell, AK", "Glennallen, AK", "Golovin, AK",
  "Goodnews Bay, AK", "Grayling, AK", "Gustavus, AK", "Haines, AK",
  "Healy, AK", "Holy Cross, AK", "Hoonah, AK", "Hooper Bay, AK",
  "Hughes, AK", "Huslia, AK", "Hydaburg, AK", "Hyder, AK", "Igiugig, AK",
  "Juneau, AK", "Kaktovik, AK", "Kalskag, AK", "Kaltag, AK",
  "Kasilof, AK", "Kenai, AK", "Ketchikan, AK", "Kiana, AK",
  "King Cove, AK", "Kipnuk, AK", "Kivalina, AK", "Klawock, AK",
  "Kobuk, AK", "Kokhanok, AK", "Koliganek, AK", "Kotlik, AK",
  "Kotzebue, AK", "Koyuk, AK", "Kwethluk, AK", "Kwigillingok, AK",
  "Lake Minchumina, AK", "Levelock, AK", "Lower Kalskag, AK",
  "Manley Hot Sprin, AK", "Manokotak, AK", "Marshall, AK", "Mc Grath, AK",
  "Mekoryuk, AK", "Metlakatla, AK", "Minto, AK", "Moose Pass, AK",
  "Mountain Village, AK", "Naknek, AK", "Napakiak, AK",
  "Nelson Lagoon, AK", "Nenana, AK", "New Stuyahok, AK", "Nikolaevsk, AK",
  "Nikolai, AK", "Nikolski, AK", "Ninilchik, AK", "Noatak, AK",
  "Nondalton, AK", "Noorvik, AK", "North Pole, AK", "Nuiqsut, AK",
  "Nulato, AK", "Pedro Bay, AK", "Perryville, AK", "Petersburg, AK",
  "Pilot Point, AK", "Pilot Station, AK", "Platinum, AK",
  "Point Baker, AK", "Point Hope, AK", "Point Lay, AK",
  "Port Alsworth, AK", "Port Graham, AK", "Port Heiden, AK",
  "Prudhoe Bay, AK", "Quinhagak, AK", "Rampart, AK", "Red Devil, AK",
  "Ruby, AK", "Russian Mission, AK", "Saint George Isl, AK",
  "Saint Marys, AK", "Saint Michael, AK", "Saint Paul Islan, AK",
  "Salcha, AK", "Sand Point, AK", "Savoonga, AK", "Scammon Bay, AK",
  "Selawik, AK", "Seward, AK", "Shageluk, AK", "Shaktoolik, AK",
  "Shishmaref, AK", "Shungnak, AK", "Sitka, AK", "Skagway, AK",
  "Slana, AK", "Sleetmute, AK", "Soldotna, AK", "South Naknek, AK",
  "Stebbins, AK", "Sterling, AK", "Stevens Village, AK", "Talkeetna, AK",
  "Tanana, AK", "Teller, AK", "Thorne Bay, AK", "Trapper Creek, AK",
  "Tuluksak, AK", "Tununak, AK", "Tyonek, AK", "Unalakleet, AK",
  "Unalaska, AK", "Valdez, AK", "Venetie, AK", "Wainwright, AK",
  "Wales, AK", "Wasilla, AK", "White Mountain, AK", "Willow, AK",
  "Wrangell, AK", "Yakutat, AK", "Abbeville, AL", "Acmar, AL",
  "Adamsville, AL", "Addison, AL", "Adger, AL", "Alberta, AL",
  "Albertville, AL", "Alden, AL", "Alexandria, AL", "Aliceville, AL",
  "Allen, AL", "Alpine, AL", "Andalusia, AL", "Anderson, AL",
  "Anniston, AL", "Arab, AL", "Ardmore, AL", "Ariton, AL", "Arley, AL",

```

"Arlington, AL", "Ashford, AL", "Ashland, AL", "Ashville, AL",
"Athens, AL", "Atmore, AL", "Attalla, AL", "Auburn, AL",
"Autaugaville, AL", "Axis, AL", "Baileyton, AL", "Banks, AL",
"Bankston, AL", "Bay Minette, AL", "Bayou La Batre, AL",
"Bear Creek, AL", "Beatrice, AL", "Beaverton, AL", "Benton, AL",
"Berry, AL", "Bessemer, AL", "Big Cove, AL", "Bigbee, AL",
"Billingsley, AL", "Birmingham, AL", "Black, AL", "Blountsville, AL",
"Bluff Park, AL", "Boaz, AL", "Boligee, AL", "Bon Secour, AL",
"Boys Ranch, AL", "Brantley, AL", "Bremen, AL", "Brent, AL",
"Bridgeport, AL", "Brierfield, AL", "Brilliant, AL",
"Brookley Field, AL", "Brookwood, AL", "Brownsboro, AL",
"Brundidge, AL", "Bryant, AL", "Buhl, AL", "Burkville, AL",
"Butler, AL", "Cahaba Heights, AL", "Calera, AL", "Camden, AL",
"Camp Hill, AL", "Campbell, AL", "Carbon Hill, AL", "Carlton, AL",
"Carrollton, AL", "Castleberry, AL", "Catherine, AL", "Cecil, AL",
"Cedar Bluff, AL", "Center Point, AL", "Centre, AL", "Centreville, AL",
"Chancellor, AL", "Chatom, AL", "Chelsea, AL", "Cherokee, AL",
"Chickasaw, AL", "Chunchula, AL", "Citronelle, AL", "Clanton, AL",
"Clayton, AL", "Cleveland, AL", "Clio, AL", "Clopton, AL",
"Coalburg, AL", "Coatopa, AL", "Codens, AL", "Coffee Springs, AL",
"Coffeeville, AL", "Coker, AL", "Collinsville, AL", "Columbia, AL",
"Columbiana, AL", "Coosa Pines, AL", "Cordova, AL", "Cottondale, AL",
"Cottonwood, AL", "Courtland, AL", "Coy, AL", "Cragford, AL",
"Crane Hill, AL", "Creola, AL", "Crestline Height, AL", "Cropwell, AL",
"Crossville, AL", "Cuba, AL", "Cullman, AL", "Cusseta, AL",
"Cypress, AL", "Dadeville, AL", "Daleville, AL", "Danville, AL",
"Daphne, AL", "Dauphin Island, AL", "Daviston, AL", "Dawson, AL",
"Deatsville, AL", "Decatur, AL", "Deer Park, AL", "Delta, AL",
"Demopolis, AL", "Detroit, AL", "Dickinson, AL", "Dixiana, AL",
"Dixons Mills, AL", "Dolomite, AL", "Dora, AL", "Double Springs, AL",
"Dozier, AL", "Duncanville, AL", "Dutton, AL", "East Brewton, AL",
"Eastaboga, AL", "Echola, AL", "Eclectic, AL", "Eight Mile, AL",
"Elba, AL", "Elberta, AL", "Eldridge, AL", "Elkmont, AL", "Elmore, AL",
"Elrod, AL", "Emelle, AL", "Empire, AL", "Enterprise, AL", "Epes, AL",
"Equality, AL", "Estillfork, AL", "Ethelsville, AL", "Eufaula, AL",
"Eutaw, AL", "Eva, AL", "Evergreen, AL", "Fackler, AL", "Fairfield, AL",
"Fairhope, AL", "Falkville, AL", "Faunsdale, AL", "Fayette, AL",
"Fitzpatrick, AL", "Five Points, AL", "Flat Rock, AL", "Flomaton, AL",
"Floral, AL", "Florence, AL", "Foley, AL", "Forest Home, AL",
"Forkland, AL", "Fort Davis, AL", "Fort Deposit, AL",
"Fort Mc Clellan, AL", "Fort Morgan, AL", "Fort Payne, AL",
"Fort Rucker, AL", "Fosters, AL", "Franklin, AL", "Frankville, AL",
"Frisco City, AL", "Fruitdale, AL", "Fruithurst, AL", "Fulton, AL",
"Fyffe, AL", "Gadsden, AL", "Gainestown, AL", "Gainesville, AL",
"Gallant, AL", "Gallion, AL", "Gantt, AL", "Gardendale, AL",
"Gaylesville, AL", "Geneva, AL", "Georgiana, AL", "Geraldine, AL",
"Gilbertown, AL", "Glencoe, AL", "Glenwood, AL", "Goodwater, AL",
"Gordo, AL", "Gordon, AL", "Goshen, AL", "Grady, AL", "Graham, AL",
"Grand Bay, AL", "Grant, AL", "Greensboro, AL", "Greenville, AL",
"Grove Hill, AL", "Groveoak, AL", "Guin, AL", "Gunter Afs, AL",
"Guntersville, AL", "Gurley, AL", "Hackleburg, AL", "Haleyville, AL",
"Hamilton, AL", "Hanceville, AL", "Hardaway, AL", "Harpersville, AL",
"Hartford, AL", "Hartselle, AL", "Harvest, AL", "Hatchechubbee, AL",
"Hayden, AL", "Hayneville, AL", "Hazel Green, AL", "Headland, AL",
"Heflin, AL", "Helena, AL", "Henagar, AL", "Higdon, AL",

"Highland Home, AL", "Hillsboro, AL", "Hodges, AL", "Hokes Bluff, AL",
"Holly Pond, AL", "Hollytree, AL", "Hollywood, AL", "Holt, AL",
"Homewood, AL", "Honoraville, AL", "Hoover, AL", "Hope Hull, AL",
"Horton, AL", "Houston, AL", "Hueytown, AL", "Huntsville, AL",
"Hurtsboro, AL", "Hytow, AL", "Ider, AL", "Indian Springs, AL",
"Irondale, AL", "Irvington, AL", "Jachin, AL", "Jack, AL",
"Jackson, AL", "Jacksons Gap, AL", "Jacksonville, AL", "Jasper, AL",
"Jemison, AL", "Jones, AL", "Joppa, AL", "Kellyton, AL", "Kennedy, AL",
"Keystone, AL", "Killen, AL", "Kimberly, AL", "Kinston, AL",
"Knoxville, AL", "Laceys Spring, AL", "Lafayette, AL", "Lanett, AL",
"Langston, AL", "Lapine, AL", "Lawley, AL", "Leeds, AL", "Leesburg, AL",
"Leighton, AL", "Lenox, AL", "Leroy, AL", "Lester, AL",
"Letohatchee, AL", "Lexington, AL", "Lillian, AL", "Lincoln, AL",
"Linden, AL", "Lineville, AL", "Lisman, AL", "Little River, AL",
"Logan, AL", "Louisville, AL", "Lower Peach Tree, AL", "Loxley, AL",
"Luverne, AL", "Lynn, AL", "Magnolia, AL", "Magnolia Springs, AL",
"Malvern, AL", "Maplesville, AL", "Marbury, AL", "Marion, AL",
"Marion Junction, AL", "Mathews, AL", "Maxwell A F B, AL",
"Maylene, AL", "Mc Calla, AL", "Mc Intosh, AL", "Mc Kenzie, AL",
"Mentone, AL", "Meridianville, AL", "Midfield, AL", "Midland City, AL",
"Midway, AL", "Millbrook, AL", "Millerville, AL", "Millport, AL",
"Millry, AL", "Mobile, AL", "Monroeville, AL", "Montevallo, AL",
"Montgomery, AL", "Morris, AL", "Morvin, AL", "Moulton, AL",
"Mount Hope, AL", "Mount Olive, AL", "Mount Vernon, AL",
"Mountain Brook, AL", "Munford, AL", "Muscadine, AL",
"Muscle Shoals, AL", "Napier Field, AL", "Nauvoo, AL", "Needham, AL",
"New Brockton, AL", "New Hope, AL", "New Market, AL", "New Site, AL",
"Newbern, AL", "Newell, AL", "Newton, AL", "Newville, AL",
"Northport, AL", "Notasulga, AL", "Oakman, AL", "Odenville, AL",
"Ohatchee, AL", "Oneonta, AL", "Opelika, AL", "Opp, AL",
"Orange Beach, AL", "Orrville, AL", "Oxford, AL", "Ozark, AL",
"Paint Rock, AL", "Pansey, AL", "Parrish, AL", "Pell City, AL",
"Pennington, AL", "Perdido, AL", "Perote, AL", "Peterman, AL",
"Phenix City, AL", "Phil Campbell, AL", "Piedmont, AL", "Pike Road, AL",
"Pine Apple, AL", "Pine Hill, AL", "Pisgah, AL", "Pittsview, AL",
"Plantersville, AL", "Pleasant Grove, AL", "Prairie, AL",
"Prattville, AL", "Prichard, AL", "Princeton, AL", "Quinton, AL",
"Ragland, AL", "Rainsville, AL", "Ralph, AL", "Ramer, AL",
"Ranburne, AL", "Randolph, AL", "Range, AL", "Red Bay, AL",
"Red Level, AL", "Reform, AL", "Remlap, AL", "Repton, AL",
"Riverside, AL", "Robertsdale, AL", "Rock Mills, AL", "Rockford, AL",
"Rogersville, AL", "Russellville, AL", "Rutledge, AL", "Safford, AL",
"Saint Stephens, AL", "Salem, AL", "Salitpa, AL", "Samson, AL",
"Saraland, AL", "Sardis, AL", "Satsuma, AL", "Sawyer ville, AL",
"Seale, AL", "Section, AL", "Selma, AL", "Seminole, AL", "Semmes, AL",
"Sheffield, AL", "Shelby, AL", "Shoal Creek, AL", "Shorter, AL",
"Shorterville, AL", "Silas, AL", "Silverhill, AL", "Skipperville, AL",
"Slocomb, AL", "Smiths, AL", "Snead, AL", "Somerville, AL",
"Southside, AL", "Spanish Fort, AL", "Springville, AL", "Sprott, AL",
"Spruce Pine, AL", "Stanton, AL", "Steele, AL", "Sterrett, AL",
"Stevenson, AL", "Stewart, AL", "Stockton, AL", "Sulligent, AL",
"Sumiton, AL", "Summerdale, AL", "Sweet Water, AL", "Sylacauga, AL",
"Sylvan Springs, AL", "Sylvania, AL", "Talladega, AL", "Tallassee, AL",
"Tanner, AL", "Taylor, AL", "Theodore, AL", "Thomaston, AL",
"Thomasville, AL", "Thorsby, AL", "Tibbie, AL", "Titus, AL",

"Toney, AL", "Town Creek, AL", "Townley, AL", "Toxey, AL",
 "Trafford, AL", "Trenton, AL", "Triana, AL", "Trinity, AL", "Troy, AL",
 "Trussville, AL", "Tuscaloosa, AL", "Tuscumbia, AL", "Tuskegee, AL",
 "Tuskegee Institu, AL", "Union Grove, AL", "Union Springs, AL",
 "Uniontown, AL", "Uriah, AL", "Valhermoso Sprin, AL", "Valley, AL",
 "Valley Head, AL", "Vance, AL", "Vandiver, AL", "Verbena, AL",
 "Vernon, AL", "Vestavia Hills, AL", "Vina, AL", "Vincent, AL",
 "Vinegar Bend, AL", "Vinemont, AL", "Vredenburgh, AL", "Wadley, AL",
 "Wagarville, AL", "Walker Springs, AL", "Ward, AL", "Warrior, AL",
 "Waterloo, AL", "Waverly, AL", "Weaver, AL", "Webb, AL", "Wedowee, AL",
 "Wellington, AL", "Weogufka, AL", "West Blocton, AL", "Wetumpka, AL",
 "Whatley, AL", "Wilmer, AL", "Wilsonville, AL", "Winfield, AL",
 "Wing, AL", "Woodland, AL", "Woodstock, AL", "Woodville, AL",
 "York, AL", "Adona, AR", "Agnos, AR", "Alco, AR", "Alexander, AR",
 "Alicia, AR", "Alix, AR", "Alma, AR", "Almyra, AR", "Alpena, AR",
 "Alzheimer, AR", "Altus, AR", "Amagon, AR", "Amity, AR", "Antoine, AR",
 "Arkadelphia, AR", "Arkansas City, AR", "Armored, AR", "Ashdown, AR",
 "Atkins, AR", "Aubrey, AR", "Augusta, AR", "Austin, AR",
 "Bald Knob, AR", "Banks, AR", "Barling, AR", "Bass, AR", "Bassett, AR",
 "Bates, AR", "Batesville, AR", "Bauxite, AR", "Bay, AR", "Bearden, AR",
 "Bee Branch, AR", "Beebe, AR", "Beech Grove, AR", "Beedeville, AR",
 "Bella Vista, AR", "Belleville, AR", "Benton, AR", "Bentonville, AR",
 "Berryville, AR", "Bethel Heights, AR", "Bexar, AR", "Big Flat, AR",
 "Bigelow, AR", "Biggers, AR", "Birdeye, AR", "Biscoe, AR",
 "Bismarck, AR", "Black Oak, AR", "Black Rock, AR", "Blakely, AR",
 "Blevins, AR", "Blue Mountain, AR", "Bluff City, AR", "Bluffton, AR",
 "Blytheville A F, AR", "Boles, AR", "Bonnerdale, AR", "Bono, AR",
 "Booneville, AR", "Boswell, AR", "Bradford, AR", "Bradley, AR",
 "Branch, AR", "Brickeys, AR", "Briggsville, AR", "Brinkley, AR",
 "Brockwell, AR", "Brookland, AR", "Bruno, AR", "Bryant, AR",
 "Buckner, AR", "Buckville, AR", "Bull Shoals, AR", "Burdette, AR",
 "Byron, AR", "Cabot, AR", "Caddo Gap, AR", "Calamine, AR", "Cale, AR",
 "Camp, AR", "Camp Joseph T Ro, AR", "Canehill, AR", "Caraway, AR",
 "Carlisle, AR", "Carthage, AR", "Casa, AR", "Cash, AR", "Casscoe, AR",
 "Cave City, AR", "Cave Springs, AR", "Cecil, AR", "Cedarville, AR",
 "Center Ridge, AR", "Centerton, AR", "Central City, AR",
 "Charleston, AR", "Charlotte, AR", "Cherokee Village, AR",
 "Cherry Valley, AR", "Chester, AR", "Choctaw, AR", "Clarendon, AR",
 "Clarkridge, AR", "Clarksville, AR", "Cleveland, AR", "Clinton, AR",
 "Coal Hill, AR", "College City, AR", "Colt, AR", "Columbus, AR",
 "Combs, AR", "Compton, AR", "Concord, AR", "Conway, AR", "Cord, AR",
 "Corning, AR", "Cotter, AR", "Cotton Plant, AR", "Cove, AR",
 "Crawfordsville, AR", "Crocketts Bluff, AR", "Crumrod, AR",
 "Cushman, AR", "Danville, AR", "Dardanelle, AR", "Datto, AR",
 "De Queen, AR", "De Valls Bluff, AR", "De Witt, AR", "Decatur, AR",
 "Deer, AR", "Delaplaine, AR", "Delaware, AR", "Delight, AR", "Dell, AR",
 "Dennard, AR", "Dermott, AR", "Des Arc, AR", "Desha, AR", "Dierks, AR",
 "Doddridge, AR", "Dogpatch, AR", "Dollarway, AR", "Dolph, AR",
 "Donaldson, AR", "Dover, AR", "Drasco, AR", "Driver, AR", "Dumas, AR",
 "Dyess, AR", "Earle, AR", "East Camden, AR", "Edgemont, AR",
 "Edmondson, AR", "El Dorado, AR", "El Paso, AR", "Elaine, AR",
 "Elizabeth, AR", "Elkins, AR", "Emerson, AR", "Emmet, AR",
 "England, AR", "Enola, AR", "Ethel, AR", "Etowah, AR", "Eudora, AR",
 "Eureka Springs, AR", "Evansville, AR", "Evening Shade, AR",
 "Everton, AR", "Fair Oaks, AR", "Farmington, AR", "Fayetteville, AR",

"Ferndale, AR", "Fifty Six, AR", "Fisher, AR", "Flippin, AR",
 "Floral, AR", "Fordyce, AR", "Foreman, AR", "Forrest City, AR",
 "Fort Chaffee, AR", "Fort Smith, AR", "Fouke, AR", "Fountain Hill, AR",
 "Fox, AR", "Franklin, AR", "Frenchmans Bayou, AR", "Friendship, AR",
 "Fulton, AR", "Gamaliel, AR", "Garfield, AR", "Garland City, AR",
 "Garner, AR", "Gassville, AR", "Gateway, AR", "Gentry, AR",
 "Georgetown, AR", "Gepp, AR", "Gillett, AR", "Gillham, AR",
 "Gilmore, AR", "Glencoe, AR", "Glenwood, AR", "Goodwin, AR",
 "Goshen, AR", "Gould, AR", "Grannis, AR", "Grapevine, AR",
 "Gravel Ridge, AR", "Gravelly, AR", "Gravette, AR", "Green Forest, AR",
 "Greenbrier, AR", "Greenway, AR", "Greenwood, AR", "Greers Ferry, AR",
 "Griffithville, AR", "Guion, AR", "Gurdon, AR", "Guy, AR",
 "Hackett, AR", "Hagarville, AR", "Hamburg, AR", "Hampton, AR",
 "Hanover, AR", "Hardy, AR", "Harrell, AR", "Harriet, AR",
 "Harrisburg, AR", "Harrison, AR", "Hartford, AR", "Hartman, AR",
 "Harvey, AR", "Hasty, AR", "Hatfield, AR", "Hattievile, AR",
 "Haynes, AR", "Hazen, AR", "Heber Springs, AR", "Hector, AR",
 "Helena, AR", "Henderson, AR", "Hensley, AR", "Heth, AR",
 "Hickory Plains, AR", "Hickory Ridge, AR", "Higginson, AR",
 "Hindsville, AR", "Hiwassee, AR", "Holly Grove, AR", "Horatio, AR",
 "Horseshoe Bend, AR", "Hot Springs Vill, AR", "Houston, AR",
 "Hoxie, AR", "Hughes, AR", "Humnoke, AR", "Humphrey, AR",
 "Huntington, AR", "Huntsville, AR", "Huttig, AR", "Ida, AR",
 "Imboden, AR", "Ingalls, AR", "Ivan, AR", "Jasper, AR", "Jefferson, AR",
 "Jersey, AR", "Jerusalem, AR", "Jessieville, AR", "Joiner, AR",
 "Jones Mills, AR", "Jonesboro, AR", "Jordan, AR", "Judsonia, AR",
 "Junction City, AR", "Keiser, AR", "Kensett, AR", "Keo, AR",
 "Kingsland, AR", "Kingston, AR", "Kirby, AR", "Knobel, AR",
 "Knoxville, AR", "Lafe, AR", "Lake Catherine, AR", "Lake City, AR",
 "Lake Hamilton, AR", "Lake Village, AR", "Lakeview, AR", "Lamar, AR",
 "Langley, AR", "Leachville, AR", "Lead Hill, AR", "Leola, AR",
 "Lepanto, AR", "Leslie, AR", "Lewisville, AR", "Lexa, AR",
 "Lincoln, AR", "Little Rock, AR", "Lockesburg, AR", "Locust Grove, AR",
 "London, AR", "Lonoke, AR", "Lonsdale, AR", "Louann, AR", "Lowell, AR",
 "Luxora, AR", "Lynn, AR", "Magazine, AR", "Magness, AR", "Magnolia, AR",
 "Malvern, AR", "Mammoth Spring, AR", "Manning, AR", "Mansfield, AR",
 "Marcella, AR", "Marianna, AR", "Marion, AR", "Marked Tree, AR",
 "Marmaduke, AR", "Marshall, AR", "Marvell, AR", "Mauwelle, AR",
 "Mayflower, AR", "Maynard, AR", "Maysville, AR", "Mc Caskill, AR",
 "Mc Crory, AR", "Mc Dougal, AR", "Mc Gehee, AR", "Mc Neil, AR",
 "Mc Rae, AR", "Mellwood, AR", "Mena, AR", "Midway, AR",
 "Mineral Springs, AR", "Minturn, AR", "Moko, AR", "Monette, AR",
 "Monticello, AR", "Montrose, AR", "Moro, AR", "Morrilton, AR",
 "Morrow, AR", "Mount Holly, AR", "Mount Ida, AR", "Mount Judea, AR",
 "Mount Pleasant, AR", "Mount Vernon, AR", "Mountain Home, AR",
 "Mountainburg, AR", "Mulberry, AR", "Murfreessboro, AR", "Nashville, AR",
 "Natural Dam, AR", "New Blaine, AR", "New Edinburg, AR", "Newark, AR",
 "Newhope, AR", "Newnata, AR", "Newport, AR", "Norfork, AR",
 "Norman, AR", "Norphlet, AR", "North, AR", "North Cedar, AR",
 "North Little Roc, AR", "O Kean, AR", "Oak Grove, AR", "Oakland, AR",
 "Oark, AR", "Oden, AR", "Ogden, AR", "Oil Trough, AR", "Okolona, AR",
 "Ola, AR", "Omaha, AR", "Oneida, AR", "Onia, AR", "Osceola, AR",
 "Oxford, AR", "Ozan, AR", "Ozark, AR", "Ozone, AR", "Palestine, AR",
 "Pangburn, AR", "Paragould, AR", "Paris, AR", "Parkdale, AR",
 "Parkin, AR", "Parks, AR", "Paron, AR", "Parthenon, AR", "Payneway, AR",

"Pea Ridge, AR", "Peach Orchard, AR", "Pearcy, AR", "Peel, AR",
 "Pelsor, AR", "Pencil Bluff, AR", "Perry, AR", "Perrytown, AR",
 "Perryville, AR", "Pettigrew, AR", "Pickens, AR", "Piggott, AR",
 "Pindall, AR", "Pine Bluff, AR", "Pineville, AR", "Plainview, AR",
 "Pleasant Grove, AR", "Pleasant Plains, AR", "Plumerville, AR",
 "Pocahontas, AR", "Pollard, AR", "Ponca, AR", "Poplar Grove, AR",
 "Portia, AR", "Portland, AR", "Pottsville, AR", "Poughkeepsie, AR",
 "Powhatan, AR", "Poyen, AR", "Prairie Grove, AR", "Prattsville, AR",
 "Prescott, AR", "Prim, AR", "Proctor, AR", "Quitman, AR",
 "Ratcliff, AR", "Ravenden, AR", "Ravenden Springs, AR", "Reader, AR",
 "Rector, AR", "Redfield, AR", "Reed, AR", "Reydell, AR", "Rison, AR",
 "Roe, AR", "Rogers, AR", "Rohwer, AR", "Roland, AR", "Romance, AR",
 "Rose Bud, AR", "Roseland, AR", "Rosie, AR", "Rosston, AR", "Rover, AR",
 "Royal, AR", "Rudy, AR", "Russellville, AR", "Saffell, AR", "Sage, AR",
 "Saint Charles, AR", "Saint Francis, AR", "Saint Joe, AR",
 "Saint Paul, AR", "Salado, AR", "Saratoga, AR", "Scotland, AR",
 "Scott, AR", "Scranton, AR", "Sedgwick, AR", "Shannon Hills, AR",
 "Sheridan, AR", "Sherrill, AR", "Sherwood, AR", "Shirley, AR",
 "Sidney, AR", "Siloam Springs, AR", "Sims, AR", "Smackover, AR",
 "Smithville, AR", "Snow Lake, AR", "Solgochachia, AR", "Springdale, AR",
 "Springfield, AR", "Stamps, AR", "Star City, AR",
 "State University, AR", "Stephens, AR", "Story, AR", "Strong, AR",
 "Sturkie, AR", "Stuttgart, AR", "Subiaco, AR", "Success, AR",
 "Sulphur Rock, AR", "Sulphur Springs, AR", "Summers, AR", "Swifton, AR",
 "Tamo, AR", "Taylor, AR", "Texarkana, AR", "Thida, AR", "Thornton, AR",
 "Tichnor, AR", "Tilly, AR", "Timbo, AR", "Tinsman, AR", "Tomato, AR",
 "Traskwood, AR", "Tucker, AR", "Tuckerman, AR", "Tumbling Shoals, AR",
 "Turrell, AR", "Twin Groves, AR", "Tyronza, AR", "Ulm, AR",
 "Umpire, AR", "Uniontown, AR", "Valley Springs, AR", "Van Buren, AR",
 "Vandervoort, AR", "Vendor, AR", "Village, AR", "Vilonia, AR",
 "Viola, AR", "Violet Hill, AR", "Wabbaseka, AR", "Wal-Mart Inc, AR",
 "Waldo, AR", "Waldron, AR", "Ward, AR", "Warm Springs, AR",
 "Warren, AR", "Washington, AR", "Watson, AR", "Waveland, AR",
 "Weiner, AR", "Wesley, AR", "West Fork, AR", "West Helena, AR",
 "West Memphis, AR", "Western Grove, AR", "Wheatley, AR", "Wickes, AR",
 "Wideman, AR", "Widener, AR", "Wilburn, AR", "Williford, AR",
 "Willisville, AR", "Wilmar, AR", "Wilmot, AR", "Wilson, AR",
 "Wilton, AR", "Winchester, AR", "Winslow, AR", "Winthrop, AR",
 "Wiseman, AR", "Witter, AR", "Witts Springs, AR", "Wooster, AR",
 "Wright, AR", "Wynne, AR", "Yellville, AR", "Yorktown, AR", "Zion, AR",
 "Alpine, AZ", "Amado, AZ", "Apache Junction, AZ",
 "Arizona Boys Ran, AZ", "Arlington, AZ", "Ash Fork, AZ", "Avondale, AZ",
 "Bagdad, AZ", "Benson, AZ", "Bisbee, AZ", "Blue, AZ", "Buckeye, AZ",
 "Bullhead City, AZ", "Bylas, AZ", "Camp Verde, AZ", "Casas Adobes, AZ",
 "Cave Creek, AZ", "Chambers, AZ", "Chandler, AZ", "Chinle, AZ",
 "Chino Valley, AZ", "Cibola, AZ", "Clarkdale, AZ", "Clifton, AZ",
 "Cochise, AZ", "Colorado City, AZ", "Concho, AZ", "Congress, AZ",
 "Coolidge, AZ", "Cornville, AZ", "Cottonwood, AZ", "Crown King, AZ",
 "Dateland, AZ", "Dennehotso, AZ", "Desert Hills, AZ", "Dewey, AZ",
 "Dolan Springs, AZ", "Douglas, AZ", "Eagar, AZ", "Eden, AZ",
 "El Mirage, AZ", "Eleven Mile Corn, AZ", "Elfrida, AZ", "Elgin, AZ",
 "Eloy, AZ", "Empire Landing, AZ", "Flagstaff, AZ", "Florence, AZ",
 "Fort Huachuca, AZ", "Fort McDowell, AZ", "Fountain Hills, AZ",
 "Franklin, AZ", "Fredonia, AZ", "Ganado, AZ", "Gila Bend, AZ",
 "Gilbert, AZ", "Glendale, AZ", "Globe, AZ", "Gold Canyon, AZ",

"Goodyear, AZ", "Green Valley, AZ", "Greenehaven, AZ",
 "Groom Creek, AZ", "Heber, AZ", "Hereford, AZ", "Higley, AZ",
 "Holbrook, AZ", "Hotevilla, AZ", "Huachuca City, AZ", "Hualapai, AZ",
 "Kaibito, AZ", "Kayenta, AZ", "Keams Canyon, AZ", "Kearny, AZ",
 "Kingman, AZ", "Kirkland, AZ", "Kykotsmovi Villa, AZ", "Lakeside, AZ",
 "Laveen, AZ", "Leupp, AZ", "Litchfield Park, AZ", "Littlefield, AZ",
 "Lukachukai, AZ", "Luke Afb, AZ", "Mammoth, AZ", "Many Farms, AZ",
 "Marana, AZ", "Marble Canyon, AZ", "Mayer, AZ", "Mc Neal, AZ",
 "Meadview, AZ", "Mesa, AZ", "Miami, AZ", "Mobile, AZ",
 "Mohave Valley, AZ", "Morenci, AZ", "Mormon Lake, AZ", "Morristown, AZ",
 "Navajo, AZ", "New River Stage, AZ", "Nogales, AZ", "Oracle, AZ",
 "Oro Valley, AZ", "Palo Verde, AZ", "Paradise Valley, AZ",
 "Patagonia, AZ", "Paulden, AZ", "Payson, AZ", "Peach Springs, AZ",
 "Pearce, AZ", "Peoria, AZ", "Peridot, AZ", "Phoenix, AZ", "Pima, AZ",
 "Pinetop, AZ", "Pinon, AZ", "Pisinemo, AZ", "Polacca, AZ", "Portal, AZ",
 "Prescott, AZ", "Prescott Valley, AZ", "Rimrock, AZ",
 "Rock Springs, AZ", "Roll, AZ", "Roosevelt, AZ", "Sacaton, AZ",
 "Safford, AZ", "Sahuarita, AZ", "Saint David, AZ", "Saint Johns, AZ",
 "Salome, AZ", "San Carlos, AZ", "San Manuel, AZ", "Scottsdale, AZ",
 "Second Mesa, AZ", "Sedona, AZ", "Seligman, AZ", "Shonto, AZ",
 "Show Low, AZ", "Sierra Vista, AZ", "Snowflake, AZ", "Somerton, AZ",
 "Sonoita, AZ", "Springerville, AZ", "Stanfield, AZ", "Strawberry, AZ",
 "Sun City, AZ", "Sun City West, AZ", "Sun Lakes, AZ", "Supai, AZ",
 "Superior, AZ", "Surprise, AZ", "Teec Nos Pos, AZ", "Tempe, AZ",
 "Thatcher, AZ", "Tolleson, AZ", "Tombstone, AZ", "Tonalea, AZ",
 "Tonopah, AZ", "Topock, AZ", "Tsaile, AZ", "Tuba City, AZ",
 "Tucson, AZ", "Vail, AZ", "Waddell, AZ", "Wellton, AZ", "Why, AZ",
 "Wickenburg, AZ", "Willcox, AZ", "Williams, AZ", "Williams Afb, AZ",
 "Winkelman, AZ", "Winslow, AZ", "Wittmann, AZ", "Yarnell, AZ",
 "Youngtown, AZ", "Yuma, AZ", "Yuma Proving Gro, AZ", "Acampo, CA",
 "Acton, CA", "Adelaide, CA", "Adelanto, CA", "Adin, CA",
 "Agua Dulce, CA", "Aguanga, CA", "Ahwahnee, CA", "Alamo, CA",
 "Albany, CA", "Albion, CA", "Alhambra, CA", "Aliso Viejo, CA",
 "Alleghany, CA", "Alpine, CA", "Alta, CA", "Alta Loma, CA",
 "Altadena, CA", "Alturas, CA", "Alviso, CA", "Amboy, CA",
 "American Canyon, CA", "Anaheim, CA", "Anderson, CA", "Angels Camp, CA",
 "Angelus Oaks, CA", "Angwin, CA", "Annapolis, CA", "Antioch, CA",
 "Anza, CA", "Apple Valley, CA", "Applegate, CA", "Aptos, CA",
 "Arbuckle, CA", "Arcadia, CA", "Argus, CA", "Arleta, CA", "Armona, CA",
 "Aromas, CA", "Arvin, CA", "Atascadero, CA", "Atherton, CA",
 "Atwater, CA", "Auberry, CA", "Auburn, CA", "Avalon, CA", "Avenal, CA",
 "Azusa, CA", "Baker, CA", "Bakersfield, CA", "Ballard, CA",
 "Ballico, CA", "Bangor, CA", "Banning, CA", "Bardsdale, CA",
 "Barstow, CA", "Bass Lake, CA", "Bassett, CA", "Bayside, CA",
 "Bear Valley, CA", "Bear Valley Spri, CA", "Beaumont, CA", "Belden, CA",
 "Bell Gardens, CA", "Bella Vista, CA", "Bellflower, CA", "Belmont, CA",
 "Belvedere, CA", "Ben Lomond, CA", "Benicia, CA", "Benton, CA",
 "Berkeley, CA", "Berry Creek, CA", "Beverly Hills, CA", "Big Bar, CA",
 "Big Bear City, CA", "Big Pine, CA", "Big River, CA", "Big Sur, CA",
 "Biggs, CA", "Birds Landing, CA", "Bloomington, CA", "Blue Lake, CA",
 "Blythe, CA", "Bodega, CA", "Bodega Bay, CA", "Bodfish, CA",
 "Bolin, CA", "Bonita, CA", "Bonsall, CA", "Boonville, CA",
 "Boron, CA", "Borrego Springs, CA", "Boulder Creek, CA",
 "Boulevard, CA", "Bradbury, CA", "Bradley, CA", "Branscomb, CA",
 "Brawley, CA", "Brea, CA", "Brentwood, CA", "Bridgeport, CA",

"Brisbane, CA", "Brooks, CA", "Browns Valley, CA", "Brownsville, CA",
 "Bryte, CA", "Buellton, CA", "Buena Park, CA", "Burbank, CA",
 "Burney, CA", "Burnt Ranch, CA", "Butte City, CA", "Butte Meadows, CA",
 "Buttonwillow, CA", "Byron, CA", "Cabazon, CA", "Calabasas, CA",
 "Calexico, CA", "California City, CA", "California Hot S, CA",
 "California State, CA", "California Valle, CA", "Calimesa, CA",
 "Calipatria, CA", "Calistoga, CA", "Callahan, CA", "Calpine, CA",
 "Calwa, CA", "Camarillo, CA", "Cambria, CA", "Cameron Park, CA",
 "Camino, CA", "Campbell, CA", "Campo, CA", "Camptonville, CA",
 "Canby, CA", "Canoga Park, CA", "Cantil, CA", "Cantua Creek, CA",
 "Canyon Country, CA", "Canyon Lake, CA", "Canyondam, CA", "Capay, CA",
 "Capistrano Beach, CA", "Capitola, CA", "Cardiff By The S, CA",
 "Carlotta, CA", "Carlsbad, CA", "Carmel, CA", "Carmel Valley, CA",
 "Carmichael, CA", "Carnelian Bay, CA", "Carpinteria, CA", "Carson, CA",
 "Caruthers, CA", "Caspar, CA", "Cassel, CA", "Castaic, CA",
 "Castro Valley, CA", "Castroville, CA", "Cathedral City, CA",
 "Catheys Valley, CA", "Cayucos, CA", "Cazadero, CA", "Cedarville, CA",
 "Ceres, CA", "Cerritos, CA", "Chatsworth, CA", "Chester, CA",
 "Chico, CA", "Chilcoot, CA", "China Lake Nwc, CA", "Chinese Camp, CA",
 "Chino, CA", "Chino Hills, CA", "Chiriaco Summit, CA", "Cholame, CA",
 "Chowchilla, CA", "Chualar, CA", "Chula Vista, CA",
 "Citrus Heights, CA", "City Of Commerce, CA", "Civic Center, CA",
 "Claremont, CA", "Clarksburg, CA", "Clayton, CA", "Clearlake, CA",
 "Clearlake Oaks, CA", "Clio, CA", "Cloverdale, CA", "Clovis, CA",
 "Coachella, CA", "Coalinga, CA", "Coarsegold, CA",
 "Coast Guard Isla, CA", "Cohasset, CA", "Cold Springs, CA", "Cole, CA",
 "Coleville, CA", "College Heights, CA", "Colma, CA", "Columbia, CA",
 "Colusa, CA", "Comptche, CA", "Concord, CA", "Cool, CA",
 "Copperopolis, CA", "Corcoran, CA", "Corning, CA", "Corona, CA",
 "Corona Del Mar, CA", "Coronado, CA", "Corte Madera, CA",
 "Costa Mesa, CA", "Cotati, CA", "Coto De Caza, CA", "Cottonwood, CA",
 "Coulterville, CA", "Courtland, CA", "Covelo, CA", "Covina, CA",
 "Cowan Heights, CA", "Coyote, CA", "Crescent City, CA",
 "Crescent Mills, CA", "Creston, CA", "Crockett, CA", "Cromberg, CA",
 "Crowley Lake, CA", "Crows Landing, CA", "Crystalaire, CA",
 "Culver City, CA", "Cutler, CA", "Cuyama, CA", "Cypress, CA",
 "Daggett, CA", "Daly City, CA", "Danville, CA", "Davenport, CA",
 "Davis, CA", "Davis Creek, CA", "Death Valley, CA", "Del Mar, CA",
 "Del Rey, CA", "Del Rey Oaks, CA", "Delano, CA", "Delhi, CA",
 "Denair, CA", "Descanso, CA", "Desert Hot Sprin, CA", "Di Giorgio, CA",
 "Diablo, CA", "Diamond Bar, CA", "Diamond Springs, CA", "Dinuba, CA",
 "Dobbins, CA", "Dos Palos, CA", "Dos Rios, CA", "Douglas City, CA",
 "Downey, CA", "Downieville, CA", "Doyle, CA", "Dublin, CA",
 "Dulzura, CA", "Dunlap, CA", "Dunnigan, CA", "Dunsmuir, CA",
 "Durham, CA", "Dutch Flat, CA", "Eagle Mountain, CA", "Earlimart, CA",
 "East Highland, CA", "East Los Angeles, CA", "East Palo Alto, CA",
 "East Rancho Domi, CA", "Easton, CA", "Echo Lake, CA", "Edgewood, CA",
 "El Cajon, CA", "El Centro, CA", "El Cerrito, CA",
 "El Dorado Hills, CA", "El Macero, CA", "El Monte, CA", "El Nido, CA",
 "El Segundo, CA", "El Sobrante, CA", "El Toro Marine C, CA",
 "Elizabeth Lake, CA", "Elk, CA", "Elk Creek, CA", "Elk Grove, CA",
 "Elverta, CA", "Emeryville, CA", "Emigrant Gap, CA", "Encinitas, CA",
 "Encino, CA", "Escalon, CA", "Escondido, CA", "Esparto, CA",
 "Essex, CA", "Etiwanda, CA", "Eureka, CA", "Exeter, CA",
 "Fair Oaks, CA", "Fairfax, CA", "Fairfield, CA", "Fall River Mills, CA",

"Fallbrook, CA", "Farmersville, CA", "Farmington, CA", "Felicity, CA",
"Fellows, CA", "Felton, CA", "Ferndale, CA", "Fiddletown, CA",
"Fig Garden Villa, CA", "Firebaugh, CA", "Fish Camp, CA",
"Flintridge, CA", "Floriston, CA", "Fontana, CA", "Foothill Ranch, CA",
"Forbestown, CA", "Forest Falls, CA", "Forest Knolls, CA",
"Foresthill, CA", "Forestville, CA", "Forks Of Salmon, CA",
"Fort Bidwell, CA", "Fort Bragg, CA", "Fort Irwin, CA",
"Fort Jones, CA", "Fort Ord, CA", "Fortuna, CA", "Foster City, CA",
"Fountain Valley, CA", "Fowler, CA", "Frazier Park, CA", "Freedom, CA",
"Freestone, CA", "Fremont, CA", "French Camp, CA", "French Gulch, CA",
"Fresno, CA", "Friant, CA", "Fullerton, CA", "Fulton, CA", "Galt, CA",
"Garden Grove, CA", "Garden Valley, CA", "Gardena, CA", "Gasquet, CA",
"Gazelle, CA", "George Afb, CA", "Georgetown, CA", "Gerber, CA",
"Geyserville, CA", "Giant Forest, CA", "Gilman Hot Sprin, CA",
"Gilroy, CA", "Glen Ellen, CA", "Glencoe, CA", "Glendale, CA",
"Glendora, CA", "Glenhaven, CA", "Glenn, CA", "Glennville, CA",
"Gold River, CA", "Gold Run, CA", "Goleta, CA", "Gonzales, CA",
"Goodyears Bar, CA", "Gorman, CA", "Granada Hills, CA",
"Grand Terrace, CA", "Grass Valley, CA", "Graton, CA", "Greenacres, CA",
"Greenfield, CA", "Greenville, CA", "Greenwood, CA", "Grenada, CA",
"Gridley, CA", "Grizzly Flats, CA", "Groveland, CA", "Grover Beach, CA",
"Guadalupe, CA", "Gualala, CA", "Guerneville, CA", "Guinda, CA",
"Gustine, CA", "Hacienda Heights, CA", "Halcyon, CA",
"Half Moon Bay, CA", "Hanford, CA", "Happy Camp, CA", "Harbor City, CA",
"Hat Creek, CA", "Havilah, CA", "Hawaiian Gardens, CA", "Hayfork, CA",
"Hayward, CA", "Hazard, CA", "Healdsburg, CA", "Heber, CA",
"Helena, CA", "Helendale, CA", "Helm, CA", "Hemet, CA", "Herald, CA",
"Hercules, CA", "Herlong, CA", "Hermosa Beach, CA", "Hesperia, CA",
"Hi Vista, CA", "Hickman, CA", "Hillsborough, CA", "Hilmar, CA",
"Hinkley, CA", "Hollister, CA", "Holly Park, CA", "Holtville, CA",
"Homeland, CA", "Homewood, CA", "Hoopa, CA", "Hope Valley, CA",
"Hopland, CA", "Hornbrook, CA", "Hornitos, CA", "Horse Creek, CA",
"Hughson, CA", "Huntington Beach, CA", "Huntington Park, CA",
"Huron, CA", "Hydesville, CA", "Idyllwild, CA", "Igo, CA",
"Imperial, CA", "Imperial Beach, CA", "Independence, CA",
"Indian Wells, CA", "Industry, CA", "Inglewood, CA", "Inverness, CA",
"Ione, CA", "Iowa Hill, CA", "Irvine, CA", "Irwindale, CA",
"Isleton, CA", "Ivanhoe, CA", "Jackson, CA", "Jacumba, CA",
"Jamestown, CA", "Jamul, CA", "Janesville, CA", "Jenner, CA",
"Johannesburg, CA", "Joshua Tree, CA", "Julian, CA", "June Lake, CA",
"Juniper Hills, CA", "Keene, CA", "Kelsey, CA", "Kelseyville, CA",
"Kelso, CA", "Kensington, CA", "Kentfield, CA", "Kenwood, CA",
"Kerman, CA", "Kernville, CA", "Kettleman City, CA", "King City, CA",
"Kings Beach, CA", "Kings Canyon Nat, CA", "Kingsburg, CA",
"Klamath, CA", "Klamath River, CA", "Kneeland, CA", "Knights Ferry, CA",
"Knights Landing, CA", "Knightsen, CA", "Korbel, CA", "Kyburz, CA",
"La Crescenta, CA", "La Grange, CA", "La Habra Heights, CA",
"La Honda, CA", "La Jolla, CA", "La Mesa, CA", "La Mirada, CA",
"La Porte, CA", "La Quinta, CA", "La Selva Beach, CA", "La Verne, CA",
"Ladera, CA", "Lafayette, CA", "Laguna Hills, CA", "Laguna Niguel, CA",
"Lagunitas, CA", "Lake City, CA", "Lake Elsinore, CA",
"Lake Forest, CA", "Lake Los Angeles, CA", "Lakehead, CA",
"Lakeport, CA", "Lakeside, CA", "Lakeview, CA", "Lakewood, CA",
"Lamont, CA", "Lancaster, CA", "Larkspur, CA", "Lathrop, CA",
"Laton, CA", "Lawndale, CA", "Laytonville, CA", "Le Grand, CA",

"Lee Vining, CA", "Lemon Grove, CA", "Lemoncove, CA",
 "Lemoore Naval Ai, CA", "Lennox, CA", "Leona Valley, CA",
 "Lewiston, CA", "Liberty Farms, CA", "Lincoln, CA", "Linden, CA",
 "Lindsay, CA", "Litchfield, CA", "Littleriver, CA", "Live Oak, CA",
 "Livermore, CA", "Livingston, CA", "Lockeford, CA", "Lockwood, CA",
 "Lodi, CA", "Loleta, CA", "Loma Linda, CA", "Loma Mar, CA",
 "Lompoc, CA", "Lone Pine, CA", "Long Beach, CA", "Loomis, CA",
 "Los Altos, CA", "Los Angeles, CA", "Los Banos, CA", "Los Gatos, CA",
 "Los Molinos, CA", "Los Nietos, CA", "Los Osos, CA", "Lost Hills, CA",
 "Lost Lake, CA", "Lotus, CA", "Lower Lake, CA", "Loyalton, CA",
 "Lucerne, CA", "Lucerne Valley, CA", "Ludlow, CA", "Lynwood, CA",
 "Lytle Creek, CA", "Macdoel, CA", "Mad River, CA", "Madera, CA",
 "Madison, CA", "Magalia, CA", "Malibu, CA", "Manchester, CA",
 "Manhattan Beach, CA", "Manteca, CA", "Manton, CA", "Mare Island, CA",
 "Maricopa, CA", "Marina, CA", "Marina Del Rey, CA",
 "Marine Corp Base, CA", "Mariposa, CA", "Marshall, CA",
 "Marysville, CA", "Mather Afb, CA", "Maxwell, CA", "Maywood, CA",
 "Mc Farland, CA", "Mc Kinleyville, CA", "Mc Kittrick, CA",
 "Mcarthur, CA", "Mcclellan Afb, CA", "Mccloud, CA", "Mead Valley, CA",
 "Meadow Valley, CA", "Meadow Vista, CA", "Mendocino, CA", "Mendota, CA",
 "Menifee, CA", "Mentone, CA", "Merced, CA", "Meridian, CA",
 "Mi Wuk Village, CA", "Middletown, CA", "Midpines, CA",
 "Midway City, CA", "Milford, CA", "Mill Valley, CA", "Millbrae, CA",
 "Millville, CA", "Milpitas, CA", "Mineral, CA", "Mira Loma, CA",
 "Miramonte, CA", "Mission Hills, CA", "Mission Viejo, CA",
 "Modesto, CA", "Moffett Field, CA", "Mojave, CA", "Mokelumne Hill, CA",
 "Monarch Bay, CA", "Monrovia, CA", "Montague, CA", "Montclair, CA",
 "Monte Sereno, CA", "Monte Vista, CA", "Montebello, CA",
 "Montecito, CA", "Monterey Park, CA", "Montgomery Creek, CA",
 "Montrose, CA", "Moorpark, CA", "Moraga, CA", "Moreno Valley, CA",
 "Morgan Hill, CA", "Morongo Valley, CA", "Morro Bay, CA",
 "Moss Beach, CA", "Mount Hamilton, CA", "Mount Shasta, CA",
 "Mountain Center, CA", "Mountain Mesa, CA", "Mountain Ranch, CA",
 "Mountain View, CA", "Mt Baldy, CA", "Murphys, CA", "Murrieta, CA",
 "Muscovy, CA", "Myers Flat, CA", "Napa, CA", "National City, CA",
 "Needles, CA", "Nevada City, CA", "New Cuyama, CA", "Newark, CA",
 "Newberry Springs, CA", "Newbury Park, CA", "Newcastle, CA",
 "Newhall, CA", "Newman, CA", "Newport Beach, CA", "Nicasio, CA",
 "Nice, CA", "Niland, CA", "Nipomo, CA", "Nipton, CA", "Norco, CA",
 "Norden, CA", "North Edwards, CA", "North Fork, CA",
 "North Highlands, CA", "North Hills, CA", "North Hollywood, CA",
 "North San Juan, CA", "Northridge, CA", "Norwalk, CA", "Novato, CA",
 "O Neals, CA", "Oak Park, CA", "Oak Run, CA", "Oak View, CA",
 "Oakhurst, CA", "Oakland, CA", "Oakley, CA", "Occidental, CA",
 "Oceano, CA", "Oceanside, CA", "Ojai, CA", "Old Station, CA",
 "Olivehurst, CA", "Ontario, CA", "Onyx, CA", "Orange, CA",
 "Orange Cove, CA", "Orangevale, CA", "Orcutt, CA", "Oregon House, CA",
 "Orick, CA", "Orinda, CA", "Orland, CA", "Orleans, CA",
 "Oro Grande, CA", "Orosi, CA", "Oroville, CA", "Oxnard, CA",
 "Pacheco, CA", "Pacific Grove, CA", "Pacific House, CA",
 "Pacific Palisade, CA", "Pacifica, CA", "Paicines, CA", "Pala, CA",
 "Palermo, CA", "Palm City, CA", "Palm Springs, CA", "Palo Alto, CA",
 "Palo Cedro, CA", "Palos Verdes Est, CA", "Panorama City, CA",
 "Paradise, CA", "Paramount, CA", "Parker Dam, CA", "Parkfield, CA",
 "Parlier, CA", "Pasadena, CA", "Patterson, CA", "Pauma Valley, CA",

"Paynes Creek, CA", "Pearsonville, CA", "Pebble Beach, CA",
"Peninsula Villag, CA", "Penn Valley, CA", "Penngrove, CA",
"Penryn, CA", "Perris, CA", "Pescadero, CA", "Petaluma, CA",
"Petrolia, CA", "Phelan, CA", "Phillips Ranch, CA", "Philo, CA",
"Pico Rivera, CA", "Piedmont, CA", "Pilot Hill, CA", "Pine Grove, CA",
"Pine Valley, CA", "Pinecrest, CA", "Pinedale, CA", "Pinole, CA",
"Pinon Hills, CA", "Pioneer, CA", "Pixley, CA", "Placentia, CA",
"Placerville, CA", "Playa Del Rey, CA", "Pleasant Grove, CA",
"Pleasant Hill, CA", "Pleasanton, CA", "Plymouth, CA",
"Point Arena, CA", "Point Mugu Nawc, CA", "Point Reyes Stat, CA",
"Pomona, CA", "Pond, CA", "Pope Valley, CA", "Port Costa, CA",
"Port Hueneme, CA", "Port Hueneme Cbc, CA", "Porter Ranch, CA",
"Porterville, CA", "Portola, CA", "Posey, CA", "Potrero, CA",
"Potter Valley, CA", "Poway, CA", "Prather, CA", "Princeton, CA",
"Prunedale, CA", "Pulga, CA", "Quartz Hill, CA", "Quincy, CA",
"Rackerby, CA", "Raisin, CA", "Ramona, CA", "Ranchita, CA",
"Rancho Cordova, CA", "Rancho Cucamonga, CA", "Rancho Dominguez, CA",
"Rancho Mirage, CA", "Rancho Murieta, CA", "Rancho Palos Ver, CA",
"Rancho Santa Mar, CA", "Randsburg, CA", "Ravendale, CA", "Raymond, CA",
"Red Bluff, CA", "Red Top, CA", "Redcrest, CA", "Redding, CA",
"Redlands, CA", "Redondo Beach, CA", "Redway, CA", "Redwood City, CA",
"Redwood Valley, CA", "Reedley, CA", "Rescue, CA", "Reseda, CA",
"Rialto, CA", "Richmond, CA", "Rio Dell, CA", "Rio Linda, CA",
"Rio Oso, CA", "Rio Vista, CA", "Ripon, CA", "Riverbank, CA",
"Riverdale, CA", "Riverside, CA", "Rocklin, CA", "Rodeo, CA",
"Rohnert Park, CA", "Romoland, CA", "Rosemead, CA", "Roseville, CA",
"Rosewood, CA", "Rossmoor, CA", "Rough And Ready, CA",
"Rowland Heights, CA", "Rubidoux, CA", "Rumsey, CA",
"Russian River, CA", "Russian River Md, CA", "Ruth, CA",
"Sacramento, CA", "Saint Helena, CA", "Salida, CA", "Salinas, CA",
"Salton City, CA", "Salyer, CA", "Samoa, CA", "San Andreas, CA",
"San Anselmo, CA", "San Ardo, CA", "San Bernardino, CA",
"San Bruno, CA", "San Buenaventura, CA", "San Carlos, CA",
"San Clemente, CA", "San Diego, CA", "San Dimas, CA",
"San Fernando, CA", "San Francisco, CA", "San Gabriel, CA",
"San Geronimo, CA", "San Gregorio, CA", "San Jacinto, CA",
"San Joaquin, CA", "San Jose, CA", "San Juan Bautist, CA",
"San Leandro, CA", "San Lorenzo, CA", "San Luis Obispo, CA",
"San Luis Rey, CA", "San Marcos, CA", "San Marino, CA",
"San Martin, CA", "San Mateo, CA", "San Pablo, CA", "San Pedro, CA",
"San Rafael, CA", "San Ramon, CA", "San Simeon, CA", "San Ysidro, CA",
"Sand City, CA", "Sanger, CA", "Santa Ana, CA", "Santa Ana Height, CA",
"Santa Barbara, CA", "Santa Clara, CA", "Santa Cruz, CA",
"Santa Fe Springs, CA", "Santa Maria, CA", "Santa Monica, CA",
"Santa Paula, CA", "Santa Rosa, CA", "Santa Susana, CA",
"Santa Ynez, CA", "Santa Ysabel, CA", "Santee, CA", "Saratoga, CA",
"Sausalito, CA", "Sawyers Bar, CA", "Scotia, CA", "Scott Bar, CA",
"Scotts Valley, CA", "Seal Beach, CA", "Seiad Valley, CA", "Selma, CA",
"Shadow Hills, CA", "Shafter, CA", "Shandon, CA", "Shasta, CA",
"Shasta Lake, CA", "Shaver Lake, CA", "Shell Beach, CA", "Sheridan, CA",
"Sherman Oaks, CA", "Shingletown, CA", "Shore Acres, CA",
"Shoshone, CA", "Sierra City, CA", "Sierra Madre, CA",
"Sierraville, CA", "Signal Hill, CA", "Simi Valley, CA",
"Smartville, CA", "Smith River, CA", "Snelling, CA", "Soda Springs, CA",
"Solana Beach, CA", "Soledad, CA", "Somerset, CA", "Somes Bar, CA",

"Somis, CA", "Sonoma, CA", "Sonora, CA", "Soquel, CA",
 "Soulsbyville, CA", "South El Monte, CA", "South Gate, CA",
 "South Lake Tahoe, CA", "South Pasadena, CA", "South San Franci, CA",
 "Spanish Flat, CA", "Spring Valley, CA", "Spring Valley La, CA",
 "Springville, CA", "Squaw Valley, CA", "Standish, CA", "Stanford, CA",
 "Stanton, CA", "Stevinson, CA", "Stinson Beach, CA", "Stockton, CA",
 "Stonyford, CA", "Stratford, CA", "Strathmore, CA", "Studio City, CA",
 "Suisun City, CA", "Summerland, CA", "Sun City, CA", "Sun Valley, CA",
 "Sunnyvale, CA", "Sunol, CA", "Susanville, CA", "Sutter, CA",
 "Sutter Creek, CA", "Sylmar, CA", "Taft, CA", "Tahoe City, CA",
 "Tahoe Vista, CA", "Tahoma, CA", "Tarzana, CA", "Taylorsville, CA",
 "Tecate, CA", "Tecopa, CA", "Temecula, CA", "Temple City, CA",
 "Templeton, CA", "Termo, CA", "Terra Bella, CA", "The Sea Ranch, CA",
 "Thousand Oaks, CA", "Thousand Palms, CA", "Three Rivers, CA",
 "Tipton, CA", "Tollhouse, CA", "Toluca Lake, CA", "Toms Place, CA",
 "Topanga, CA", "Topaz, CA", "Torrance, CA", "Tracy, CA",
 "Tranquillity, CA", "Travis Afb, CA", "Trinity Center, CA",
 "Trowbridge, CA", "Truckee, CA", "Tujunga, CA", "Tulare, CA",
 "Tulelake, CA", "Tuolumne, CA", "Tupman, CA", "Turlock, CA",
 "Tustin, CA", "Twain, CA", "Twain Harte, CA", "Twentynine Palms, CA",
 "Twin Bridges, CA", "Ukiah, CA", "Union City, CA",
 "Univ Of The Paci, CA", "Upland, CA", "Upper Lake, CA", "Vacaville, CA",
 "Valencia, CA", "Vallecito, CA", "Vallejo, CA", "Valley Center, CA",
 "Valley Ford, CA", "Valley Springs, CA", "Valley Village, CA",
 "Valyermo, CA", "Van Nuys, CA", "Venice, CA", "Vernon, CA", "Vidal, CA",
 "Villa Park, CA", "Vinton, CA", "Visalia, CA", "Vista, CA",
 "Volcano, CA", "Walnut Creek, CA", "Walnut Grove, CA",
 "Warner Springs, CA", "Waterford, CA", "Weaverville, CA", "Weldon, CA",
 "Wendel, CA", "West Covina, CA", "West Hills, CA", "West Hollywood, CA",
 "West Menlo Park, CA", "West Point, CA", "West Sacramento, CA",
 "Westhaven, CA", "Westlake Village, CA", "Westminster, CA",
 "Westmorland, CA", "Westport, CA", "Wheatland, CA", "White Water, CA",
 "Whitmore, CA", "Whittier, CA", "Wildomar, CA", "Wildwood, CA",
 "Williams, CA", "Willits, CA", "Willow Creek, CA", "Willow Springs, CA",
 "Willows, CA", "Wilmington, CA", "Wilseyville, CA", "Wilton, CA",
 "Winchester, CA", "Windsor, CA", "Winnetka, CA", "Winters, CA",
 "Winton, CA", "Wishon, CA", "Witter Springs, CA", "Wofford Heights, CA",
 "Woodacre, CA", "Woodbridge, CA", "Woodlake, CA", "Woodland, CA",
 "Woodland Hills, CA", "Woodside, CA", "Woody, CA", "Wrightwood, CA",
 "Yorba Linda, CA", "Yorkville, CA", "Yountville, CA", "Yreka, CA",
 "Yuba City, CA", "Yucaipa, CA", "Yucca Valley, CA", "Zamora, CA",
 "Agate, CO", "Aguilar, CO", "Akron, CO", "Alamosa, CO",
 "Allenspark, CO", "Almont, CO", "Amherst, CO", "Anton, CO",
 "Antonito, CO", "Arapahoe, CO", "Arboles, CO", "Arlington, CO",
 "Arriba, CO", "Arvada, CO", "Aspen, CO", "Atwood, CO", "Ault, CO",
 "Aurora, CO", "Austin, CO", "Bailey, CO", "Basalt, CO",
 "Battlement Mesa, CO", "Bayfield, CO", "Bedrock, CO", "Bellvue, CO",
 "Bennett, CO", "Berthoud, CO", "Bethune, CO", "Beulah, CO",
 "Black Hawk, CO", "Blanca, CO", "Bond, CO", "Boone, CO", "Boulder, CO",
 "Bow Mar, CO", "Brandon, CO", "Branson, CO", "Briggsdale, CO",
 "Bristol, CO", "Broomfield, CO", "Brush, CO", "Buena Vista, CO",
 "Burlington, CO", "Byers, CO", "Caddoa, CO", "Cahone, CO", "Calhan, CO",
 "Campo, CO", "Canon City, CO", "Carr, CO", "Castle Rock, CO",
 "Cedaredge, CO", "Center, CO", "Cherry Hills Vil, CO",
 "Cheyenne Mtn Afb, CO", "Cheyenne Wells, CO", "Chivington, CO",

"Cimarron, CO", "Clark, CO", "Clifton, CO", "Climax, CO",
"Coalmont, CO", "Collbran, CO", "Colorado Springs, CO",
"Commerce City, CO", "Conifer, CO", "Cope, CO", "Cortez, CO",
"Craig, CO", "Crawford, CO", "Creede, CO", "Crested Butte, CO",
"Crook, CO", "Cuchara, CO", "Dacono, CO", "De Beque, CO", "Deckers, CO",
"Deer Trail, CO", "Delhi, CO", "Delta, CO", "Denver, CO", "Deora, CO",
"Dinosaur, CO", "Divide, CO", "Dolores, CO", "Dove Creek, CO",
"Drake, CO", "Durango, CO", "Eagle, CO", "Eaton, CO", "Eckert, CO",
"Eckley, CO", "Edgewater, CO", "Egnar, CO", "Elbert, CO",
"Elizabeth, CO", "Elk Springs, CO", "Englewood, CO", "Erie, CO",
"Estes Park, CO", "Evans, CO", "Evergreen, CO", "Fairplay, CO",
"Farisita, CO", "Farista, CO", "Federal Heights, CO", "Flagler, CO",
"Fleming, CO", "Florence, CO", "Florissant, CO", "Fort Carson, CO",
"Fort Collins, CO", "Fort Garland, CO", "Fort Morgan, CO",
"Fountain, CO", "Fowler, CO", "Foxton, CO", "Franktown, CO",
"Fruita, CO", "Fruitvale, CO", "Galeton, CO", "Garden City, CO",
"Gateway, CO", "Genoa, CO", "Gill, CO", "Glendale, CO",
"Glenwood Springs, CO", "Golden, CO", "Granada, CO", "Granby, CO",
"Grand Junction, CO", "Grand Lake, CO", "Granite, CO", "Greeley, CO",
"Greenwood Villag, CO", "Grover, CO", "Guffey, CO", "Gunnison, CO",
"Gypsum, CO", "Hale, CO", "Hamilton, CO", "Hartman, CO", "Haswell, CO",
"Haxtun, CO", "Hayden, CO", "Henderson, CO", "Hesperus, CO",
"Highlands Ranch, CO", "Hillrose, CO", "Holly, CO", "Holyoke, CO",
"Hooper, CO", "Hotchkiss, CO", "Howard, CO", "Hoyt, CO", "Hudson, CO",
"Hugo, CO", "Idaho Springs, CO", "Ignacio, CO", "Iliff, CO",
"Jamestown, CO", "Jansen, CO", "Jefferson, CO", "Joes, CO",
"Johnstown, CO", "Julesburg, CO", "Karval, CO", "Keenesburg, CO",
"Kersey, CO", "Keystone, CO", "Kiowa, CO", "Kirk, CO", "Kit Carson, CO",
"Kremmling, CO", "La Garita, CO", "La Jara, CO", "La Salle, CO",
"Lafayette, CO", "Laird, CO", "Lake City, CO", "Lake George, CO",
"Lakewood, CO", "Lamar, CO", "Laporte, CO", "Larkspur, CO",
"Last Chance, CO", "Leadville, CO", "Lewis, CO", "Limon, CO",
"Lindon, CO", "Littleton, CO", "Lochbui, CO", "Loma, CO",
"Longmont, CO", "Louisville, CO", "Loveland, CO", "Lycan, CO",
"Lyons, CO", "Mack, CO", "Mancos, CO", "Manitou Springs, CO",
"Manzanola, CO", "Marble, CO", "Matheson, CO", "Maybell, CO",
"Mc Clave, CO", "Mc Coy, CO", "Meeker, CO", "Meredith, CO", "Mesa, CO",
"Mesita, CO", "Milliken, CO", "Moffat, CO", "Monte Vista, CO",
"Montrose, CO", "Monument, CO", "Morrison, CO", "Mosca, CO",
"Mountain View, CO", "Nathrop, CO", "Naturita, CO", "Nederland, CO",
"New Castle, CO", "New Raymer, CO", "North Avondale, CO",
"North Pole, CO", "Northglenn, CO", "Norwood, CO", "Nucla, CO",
"Nunn, CO", "Oak Creek, CO", "Olathe, CO", "Olney Springs, CO",
"Ophir, CO", "Orchard, CO", "Ordway, CO", "Otis, CO", "Ouray, CO",
"Ovid, CO", "Padroni, CO", "Pagosa Springs, CO", "Palisade, CO",
"Palmer Lake, CO", "Paonia, CO", "Parker, CO", "Parlin, CO",
"Parshall, CO", "Peetz, CO", "Penrose, CO", "Peyton, CO", "Pierce, CO",
"Pine, CO", "Pinecliffe, CO", "Pitkin, CO", "Placerville, CO",
"Platteville, CO", "Pleasant View, CO", "Powderhorn, CO", "Pueblo, CO",
"Pueblo West, CO", "Ramah, CO", "Rangely, CO", "Red Feather Lake, CO",
"Redvale, CO", "Ridgway, CO", "Rifle, CO", "Rocky Ford, CO",
"Roggen, CO", "Rollinsville, CO", "Rush, CO", "Rye, CO", "Saguache, CO",
"Salida, CO", "San Acacio, CO", "San Pablo, CO", "Sanford, CO",
"Sedgwick, CO", "Seibert, CO", "Silt, CO", "Silverton, CO", "Simla, CO",
"Slater, CO", "Snowmass, CO", "Snyder, CO", "Somerset, CO",

"South Fork, CO", "Springfield, CO", "Steamboat Spring, CO",
 "Sterling, CO", "Stoneham, CO", "Strasburg, CO", "Stratton, CO",
 "Sugar City, CO", "Telluride, CO", "Thornton, CO", "Timpas, CO",
 "Toponas, CO", "Towaoc, CO", "Towner, CO", "Trinchera, CO",
 "Twin Lakes, CO", "United States Ai, CO", "Utleyville, CO", "Vail, CO",
 "Vernon, CO", "Vilas, CO", "Villa Grove, CO", "Villegreen, CO",
 "Virginia Dale, CO", "Vona, CO", "Walden, CO", "Walsh, CO", "Ward, CO",
 "Watkins, CO", "Wattenburg, CO", "Weldona, CO", "Wellington, CO",
 "Westcliffe, CO", "Westminster, CO", "Weston, CO", "Wetmore, CO",
 "Wheat Ridge, CO", "Whitewater, CO", "Wiley, CO", "Willard, CO",
 "Windsor, CO", "Woodland Park, CO", "Yellow Jacket, CO", "Yoder, CO",
 "Yuma, CO", "Amston, CT", "Andover, CT", "Ansonia, CT", "Avon, CT",
 "Baltic, CT", "Bantam, CT", "Beacon Falls, CT", "Berlin, CT",
 "Bethany, CT", "Bethel, CT", "Bethlehem, CT", "Bloomfield, CT",
 "Bolton, CT", "Bozrah, CT", "Branford, CT", "Bridgeport, CT",
 "Bridgewater, CT", "Bristol, CT", "Brookfield, CT", "Brooklyn, CT",
 "Burlington, CT", "Byram, CT", "Canaan, CT", "Canterbury, CT",
 "Canton, CT", "Canton Center, CT", "Centerbrook, CT", "Chaplin, CT",
 "Cheshire, CT", "Chester, CT", "Clinton, CT", "Colchester, CT",
 "Colebrook, CT", "Collinsville, CT", "Columbia, CT", "Cos Cob, CT",
 "Coventry, CT", "Cromwell, CT", "Danbury, CT", "Danielson, CT",
 "Darien, CT", "Dayville, CT", "Deep River, CT", "Derby, CT",
 "Durham, CT", "East Berlin, CT", "East Canaan, CT", "East Granby, CT",
 "East Haddam, CT", "East Hampton, CT", "East Hartford, CT",
 "East Hartland, CT", "East Haven, CT", "East Killingly, CT",
 "East Lyme, CT", "East Windsor, CT", "Eastford, CT", "Easton, CT",
 "Ellington, CT", "Enfield, CT", "Essex, CT", "Fairfield, CT",
 "Falls Village, CT", "Farmington, CT", "Gales Ferry, CT",
 "Gaylordsville, CT", "Gilman, CT", "Glastonbury, CT", "Goshen, CT",
 "Granby, CT", "Greenwich, CT", "Groton, CT", "Guilford, CT",
 "Haddam, CT", "Hamden, CT", "Hampton, CT", "Hartford, CT",
 "Harwinton, CT", "Hebron, CT", "Higganum, CT", "Ivoryton, CT",
 "Jewett City, CT", "Kent, CT", "Killingworth, CT", "Lakeside, CT",
 "Lakeville, CT", "Lebanon, CT", "Ledyard, CT", "Litchfield, CT",
 "Madison, CT", "Manchester, CT", "Mansfield Center, CT",
 "Maple Hill, CT", "Marlborough, CT", "Meriden, CT", "Middlebury, CT",
 "Middlefield, CT", "Middletown, CT", "Milford, CT", "Monroe, CT",
 "Montville, CT", "Moodus, CT", "Moosup, CT", "Morris, CT", "Mystic, CT",
 "Naugatuck, CT", "New Britain, CT", "New Canaan, CT",
 "New Fairfield, CT", "New Hartford, CT", "New Haven, CT",
 "New London, CT", "New Milford, CT", "New Preston Marb, CT",
 "Newtown, CT", "Niantic, CT", "Norfolk, CT", "North Branford, CT",
 "North Canton, CT", "North Franklin, CT", "North Granby, CT",
 "North Grosvenord, CT", "North Haven, CT", "North Stonington, CT",
 "North Windham, CT", "Northfield, CT", "Northford, CT", "Norwalk, CT",
 "Norwich, CT", "Oakdale, CT", "Oakville, CT", "Old Greenwich, CT",
 "Old Lyme, CT", "Old Saybrook, CT", "Orange, CT", "Oxford, CT",
 "Pawcatuck, CT", "Plainfield, CT", "Plainville, CT", "Plantsville, CT",
 "Pleasant Valley, CT", "Plymouth, CT", "Pomfret Center, CT",
 "Portland, CT", "Preston, CT", "Prospect, CT", "Putnam, CT",
 "Quaker Hill, CT", "Quinebaug, CT", "Ridgefield, CT", "Ridgeway, CT",
 "Riverside, CT", "Riverton, CT", "Rockfall, CT", "Rocky Hill, CT",
 "Roxbury, CT", "Salem, CT", "Salisbury, CT", "Sandy Hook, CT",
 "Scotland, CT", "Seymour, CT", "Sharon, CT", "Shelton, CT",
 "Sherman, CT", "Simsbury, CT", "Somers, CT", "South Glastonbur, CT",

"South Kent, CT", "South Windham, CT", "South Windsor, CT",
 "Southbury, CT", "Southington, CT", "Southport, CT",
 "Stafford Springs, CT", "Stamford, CT", "Sterling, CT",
 "Stonington, CT", "Storrs Mansfield, CT", "Stratford, CT",
 "Suffield, CT", "Taftville, CT", "Tariffville, CT", "Terryville, CT",
 "Thomaston, CT", "Thompson, CT", "Tolland, CT", "Torrington, CT",
 "Trumbull, CT", "Uncasville, CT", "Unionville, CT",
 "Vernon Rockville, CT", "Voluntown, CT", "W Hartford, CT",
 "Wallingford, CT", "Warren, CT", "Warrenville, CT",
 "Washington Depot, CT", "Waterbury, CT", "Waterford, CT",
 "Watertown, CT", "Weatogue, CT", "West Cornwall, CT", "West Granby, CT",
 "West Haven, CT", "West Redding, CT", "West Simsbury, CT",
 "West Suffield, CT", "West Willington, CT", "Westbrook, CT",
 "Weston, CT", "Westport, CT", "Wethersfield, CT", "Willimantic, CT",
 "Wilton, CT", "Windham, CT", "Windsor, CT", "Windsor Locks, CT",
 "Windsorville, CT", "Winsted, CT", "Wolcott, CT", "Woodbridge, CT",
 "Woodbury, CT", "Woodstock, CT", "Woodstock Valley, CT", "Pentagon, DC",
 "Washington, DC", "Bear, DE", "Bethany Beach, DE", "Bethel, DE",
 "Bridgeville, DE", "Camden Wyoming, DE", "Claymont, DE", "Clayton, DE",
 "Dagsboro, DE", "Delmar, DE", "Dewey Beach, DE", "Dover, DE",
 "Dover Afb, DE", "Edgemoor, DE", "Ellendale, DE", "Felton, DE",
 "Frankford, DE", "Frederica, DE", "Georgetown, DE", "Greenville, DE",
 "Greenwood, DE", "Harbeson, DE", "Harrington, DE", "Hartly, DE",
 "Hockessin, DE", "Houston, DE", "Laurel, DE", "Lewes, DE",
 "Lincoln, DE", "Long Neck, DE", "Magnolia, DE", "Manor, DE",
 "Marshallton, DE", "Marydel, DE", "Middletown, DE", "Milford, DE",
 "Millville, DE", "Milton, DE", "Newark, DE", "Newport, DE",
 "Seaford, DE", "Selbyville, DE", "Smyrna, DE", "Talleyville, DE",
 "Townsend, DE", "Viola, DE", "Wilmington, DE", "Airport, FL",
 "Alford, FL", "Aloma, FL", "Altamonte Spring, FL", "Altha, FL",
 "Altoona, FL", "Alva, FL", "Amelia Island, FL", "Anthony, FL",
 "Apalachicola, FL", "Apollo Beach, FL", "Apopka, FL", "Arcadia, FL",
 "Archer, FL", "Astatula, FL", "Astor, FL", "Atlantic Beach, FL",
 "Auburndale, FL", "Avon Park, FL", "Azalea Park, FL", "Babson Park, FL",
 "Baker, FL", "Bal Harbour, FL", "Baldwin, FL", "Barefoot Bay, FL",
 "Bartow, FL", "Bascom, FL", "Basinger, FL", "Bell, FL",
 "Belle Glade, FL", "Belleair Beach, FL", "Belleair Bluffs, FL",
 "Big Pine Key, FL", "Blountstown, FL", "Boca Raton, FL", "Bokeelia, FL",
 "Bonifay, FL", "Bonita Springs, FL", "Boynton Beach, FL",
 "Boys Ranch, FL", "Braden River, FL", "Bradenton, FL",
 "Bradenton Beach, FL", "Brandon, FL", "Branford, FL",
 "Briny Breezes, FL", "Bristol, FL", "Bronson, FL", "Brooker, FL",
 "Brooksville, FL", "Bryceville, FL", "Buena Ventura La, FL",
 "Bunnell, FL", "Bushnell, FL", "Callahan, FL", "Campbellton, FL",
 "Canal Point, FL", "Cantonment, FL", "Cape Canaveral, FL",
 "Cape Coral Centr, FL", "Captiva, FL", "Carl Fisher, FL",
 "Carol City, FL", "Carrabelle, FL", "Carrollwood, FL", "Caryville, FL",
 "Casselberry, FL", "Cecil Field Nas, FL", "Cedar Key, FL",
 "Center Hill, FL", "Century, FL", "Chattahoochee, FL", "Chiefland, FL",
 "Chipley, FL", "Christmas, FL", "Chuluota, FL", "Citra, FL",
 "City Of Sunrise, FL", "Clarksville, FL", "Clearwater, FL",
 "Clermont, FL", "Clewiston, FL", "Cocoa, FL", "Cocoa Beach, FL",
 "College Parkway, FL", "College Plaza, FL", "Coral Gables, FL",
 "Coral Springs, FL", "Cortez, FL", "Cottondale, FL",
 "Crawfordville, FL", "Crescent Beach, FL", "Crescent City, FL",

"Crestview, FL", "Cypress Gardens, FL", "Dania, FL", "Davenport, FL",
 "Davie, FL", "Day, FL", "Daytona Beach, FL", "De Funiak Spring, FL",
 "De Leon Springs, FL", "Debary, FL", "Deerfield Beach, FL",
 "Deland, FL", "Delray Beach, FL", "Deltona, FL", "Dona Vista, FL",
 "Dover, FL", "Duette, FL", "Dundee, FL", "Dunedin, FL", "Dunlawton, FL",
 "Eagle Lake, FL", "Earleton, FL", "East Palatka, FL", "Eatonville, FL",
 "Eau Gallie, FL", "Ebro, FL", "Edgewater, FL", "Eglin A F B, FL",
 "El Jobean, FL", "Elkton, FL", "Ellenton, FL", "Eloise, FL",
 "Englewood, FL", "Estero, FL", "Eustis, FL", "Fairvilla, FL",
 "Fellsmere, FL", "Fern Park, FL", "Flagler Beach, FL", "Florahome, FL",
 "Florida City, FL", "Forest City, FL", "Forest Lakes, FL",
 "Fort Lauderdale, FL", "Fort Meade, FL", "Fort Myers, FL",
 "Fort Myers Beach, FL", "Fort Pierce, FL", "Fort Walton Beac, FL",
 "Fort White, FL", "Fountain, FL", "Freeport, FL", "Frostproof, FL",
 "Fruitland Park, FL", "Gainesville, FL", "Geneva, FL", "Georgetown, FL",
 "Gibsonston, FL", "Glen Ridge, FL", "Glen Saint Mary, FL",
 "Graceville, FL", "Grand Island, FL", "Grand Ridge, FL",
 "Green Cove Sprin, FL", "Greenacres, FL", "Greenville, FL",
 "Greenwood, FL", "Grenelefe, FL", "Grove City, FL", "Groveland, FL",
 "Gulf Breeze, FL", "Haines Creek, FL", "Hallandale, FL", "Hampton, FL",
 "Hastings, FL", "Havana, FL", "Haverhill, FL", "Hawthorne, FL",
 "Heathrow, FL", "Hialeah, FL", "High Springs, FL", "Highland Beach, FL",
 "Hilliard, FL", "Hobe Sound, FL", "Holiday, FL", "Holly Hill, FL",
 "Hollywood, FL", "Holt, FL", "Homestead, FL", "Homestead Air Fo, FL",
 "Horseshoe Beach, FL", "Hosford, FL", "Howey In The Hil, FL",
 "Hudson, FL", "Hunt Club, FL", "Immokalee, FL", "Indialantic, FL",
 "Indian Harbor Be, FL", "Indian River Sho, FL", "Indiantown, FL",
 "Interlachen, FL", "Islamorada, FL", "Jacksonville, FL",
 "Jacksonville Bea, FL", "Jacksonville N A, FL", "Jasper, FL", "Jay, FL",
 "Jennings, FL", "Jensen Beach, FL", "Jupiter, FL", "Kathleen, FL",
 "Kenansville, FL", "Kendall, FL", "Kennedy Space Ce, FL",
 "Kenneth City, FL", "Key Biscayne, FL", "Keystone Heights, FL",
 "Kinard, FL", "Kissimmee, FL", "Labelle, FL", "Lady Lake, FL",
 "Lake Alfred, FL", "Lake Buena Vista, FL", "Lake Butler, FL",
 "Lake City, FL", "Lake Helen, FL", "Lake Panasoffkee, FL",
 "Lake Park, FL", "Lake Placid, FL", "Lake Tarpon, FL", "Lake Wales, FL",
 "Lake Worth, FL", "Lakeland, FL", "Lamont, FL", "Land O Lakes, FL",
 "Lantana, FL", "Largo, FL", "Laurel Hill, FL", "Lawtey, FL", "Lee, FL",
 "Leesburg, FL", "Lehigh Acres, FL", "Lighthouse Point, FL",
 "Lithia, FL", "Lockhart, FL", "Longwood, FL", "Lorida, FL",
 "Loxahatchee, FL", "Lulu, FL", "Lutz, FL", "Lynn Haven, FL",
 "Macclenny, FL", "Madeira Beach, FL", "Madison, FL", "Malone, FL",
 "Marathon, FL", "Marco Island, FL", "Margate, FL", "Marianna, FL",
 "Mary Esther, FL", "Mayo, FL", "Mc Alpin, FL", "Meadows Village, FL",
 "Melbourne, FL", "Melbourne Beach, FL", "Melbourne Villag, FL",
 "Melrose, FL", "Merritt Island, FL", "Miami, FL", "Miami Shores, FL",
 "Miami Springs, FL", "Micanopy, FL", "Mid Venice, FL", "Middleburg, FL",
 "Milton, FL", "Mims, FL", "Miramar, FL", "Monticello, FL",
 "Montverde, FL", "Moore Haven, FL", "Morrison, FL", "Mount Dora, FL",
 "Mulberry, FL", "Myakka City, FL", "Naples, FL", "Naval Air Statio, FL",
 "Naval Training C, FL", "Navarre, FL", "Neptune Beach, FL",
 "New Port Richey, FL", "New Smyrna Beach, FL", "Newberry, FL",
 "Niceville, FL", "Nokomis, FL", "North Bay Villag, FL",
 "North Coral Spri, FL", "North Miami, FL", "North Miami Beac, FL",
 "North Palm Beach, FL", "North Port, FL", "O Brien, FL", "Oak Hill, FL",

"Oakland Park, FL", "Ocean Reef, FL", "Ochopee, FL", "Ocklawaha, FL",
"Ocoee, FL", "Odessa, FL", "Ojus, FL", "Okahumpka, FL",
"Okeechobee, FL", "Old Town, FL", "Oldsmar, FL", "Olympia Heights, FL",
"Ona, FL", "Opa Locka, FL", "Orange City, FL", "Orange Park, FL",
"Orlando, FL", "Orlo Vista, FL", "Ormond Beach, FL", "Osprey, FL",
"Osteen, FL", "Oviedo, FL", "Pace, FL", "Pahokee, FL", "Paisley, FL",
"Palatka, FL", "Palm Bay, FL", "Palm Beach, FL", "Palm Beach Garde, FL",
"Palm City, FL", "Palm Coast, FL", "Palm Harbor, FL", "Palma Sola, FL",
"Palmetto, FL", "Panacea, FL", "Panama City, FL",
"Panama City Beac, FL", "Parrish, FL", "Patrick A F B, FL",
"Pembroke Pines, FL", "Pensacola, FL", "Perrine, FL", "Perry, FL",
"Pierson, FL", "Pine Castle, FL", "Pine Hills, FL", "Pinellas Park, FL",
"Pinetta, FL", "Placida, FL", "Plant City, FL", "Plantation, FL",
"Poinciana, FL", "Polk City, FL", "Pomona Park, FL",
"Pompano Beach, FL", "Ponce De Leon, FL", "Ponte Vedra Beac, FL",
"Port Charlotte, FL", "Port Orange, FL", "Port Richey, FL",
"Port Saint Joe, FL", "Port Saint John, FL", "Port Saint Lucie, FL",
"Princeton, FL", "Punta Gorda, FL", "Quail Heights, FL", "Quincy, FL",
"Raiford, FL", "Reddick, FL", "Ridge Manor, FL", "Ridge Manor Esta, FL",
"Ridge Manor West, FL", "Riverview, FL", "Riviera Beach, FL",
"Rockledge, FL", "Royal Palm Beach, FL", "Ruskin, FL",
"Safety Harbor, FL", "Saint Augustine, FL", "Saint Cloud, FL",
"Saint George Isl, FL", "Saint James City, FL", "Saint Petersburg, FL",
"Salem, FL", "Salt Springs, FL", "San Antonio, FL", "San Mateo, FL",
"Sand Lake, FL", "Sanderson, FL", "Sandestin, FL", "Sanford, FL",
"Sanibel, FL", "Santa Fe, FL", "Santa Rosa Beach, FL", "Sarasota, FL",
"Sarasota Square, FL", "Satsuma, FL", "Sebastian, FL", "Sebring, FL",
"Seffner, FL", "Seminole, FL", "Seville, FL", "Shady Hills, FL",
"Shalimar, FL", "Sneads, FL", "Sopchoppy, FL", "Sorrento, FL",
"South Bay, FL", "South Miami, FL", "South Trail, FL",
"South Venice, FL", "Southport, FL", "Southside, FL", "Spring Hill, FL",
"Springs Plaza, FL", "Starke, FL", "Steinhatchee, FL", "Stuart, FL",
"Summerland Key, FL", "Sun City Center, FL", "Sunrise, FL",
"Tallahassee, FL", "Tamarac, FL", "Tampa, FL", "Tarpon Springs, FL",
"Tavares, FL", "Tavernier, FL", "Tequesta, FL", "Thonotosassa, FL",
"Tice, FL", "Tierra Verde, FL", "Titusville, FL", "Trenton, FL",
"Union Park, FL", "Valparaiso, FL", "Valrico, FL", "Venice, FL",
"Ventura, FL", "Venus, FL", "Vernon, FL", "Vero Beach, FL",
"Village Of Golf, FL", "Waldo, FL", "Walnut Hill, FL", "Wauchula, FL",
"Weirsdale, FL", "Wellborn, FL", "Wesley Chapel, FL",
"West Palm Beach, FL", "Westgate, FL", "Westville, FL",
"Wewahitchka, FL", "White Springs, FL", "Whitney Beach, FL",
"Wildwood, FL", "Williston, FL", "Wimauma, FL", "Windermere, FL",
"Winter Garden, FL", "Winter Haven, FL", "Winter Park, FL",
"Winter Springs, FL", "Yalaha, FL", "Youngstown, FL", "Yulee, FL",
"Zellwood, FL", "Zephyrhills, FL", "Zolfo Springs, FL", "Abac, GA",
"Abbeville, GA", "Acworth, GA", "Adairsville, GA", "Adel, GA",
"Adrian, GA", "Ailey, GA", "Alamo, GA", "Alapaha, GA", "Albany, GA",
"Allenhurst, GA", "Allentown, GA", "Alma, GA", "Alpharetta, GA",
"Alto, GA", "Alvaton, GA", "Ambrose, GA", "Andersonville, GA",
"Appling, GA", "Arabi, GA", "Aragon, GA", "Arlington, GA",
"Armuchee, GA", "Arnoldsville, GA", "Ashburn, GA", "Athens, GA",
"Atlanta, GA", "Attapulcus, GA", "Auburn, GA", "Augusta, GA",
"Austell, GA", "Avera, GA", "Avondale Estates, GA", "Axson, GA",
"Baconton, GA", "Bainbridge, GA", "Baldwin, GA", "Ball Ground, GA",

"Barnesville, GA", "Barney, GA", "Bartow, GA", "Baxley, GA",
"Bemiss, GA", "Bethlehem, GA", "Bishop, GA", "Blackshear, GA",
"Blairsville, GA", "Blakely, GA", "Bloomington, GA", "Blue Ridge, GA",
"Bluffton, GA", "Blythe, GA", "Bogart, GA", "Bonaire, GA", "Boston, GA",
"Bowdon, GA", "Bowersville, GA", "Bowman, GA", "Braselton, GA",
"Bremen, GA", "Bridgeboro, GA", "Brinson, GA", "Bristol, GA",
"Bronwood, GA", "Brooklet, GA", "Brooks, GA", "Broxton, GA",
"Brunswick, GA", "Buchanan, GA", "Buckhead, GA", "Buford, GA",
"Butler, GA", "Byromville, GA", "Cadwell, GA", "Cairo, GA",
"Calhoun, GA", "Calvary, GA", "Camilla, GA", "Canon, GA", "Canton, GA",
"Carlton, GA", "Carnesville, GA", "Carrollton, GA", "Cartersville, GA",
"Cataula, GA", "Cave Spring, GA", "Cedartown, GA", "Centerville, GA",
"Centerville Gwin, GA", "Chamblee, GA", "Chatsworth, GA",
"Chauncey, GA", "Cherrylog, GA", "Chester, GA", "Chickamauga, GA",
"Chula, GA", "Cisco, GA", "Clarkesville, GA", "Clarkston, GA",
"Claxton, GA", "Clayton, GA", "Clermont, GA", "Cleveland, GA",
"Climax, GA", "Cloudland, GA", "Clyattville, GA", "Clyo, GA",
"Cobb, GA", "Cobbtown, GA", "Cochran, GA", "Cogdell, GA", "Cohutta, GA",
"Colbert, GA", "Coleman, GA", "College Park, GA", "Collins, GA",
"Colquitt, GA", "Columbus, GA", "Comer, GA", "Commerce, GA",
"Concord, GA", "Conley, GA", "Conyers, GA", "Coolidge, GA",
"Cordele, GA", "Cornelia, GA", "Crandall, GA", "Crawford, GA",
"Crawfordville, GA", "Crescent, GA", "Culloden, GA", "Cumming, GA",
"Cusseta, GA", "Custer Terrace, GA", "Cuthbert, GA", "Dacula, GA",
"Dahlonega, GA", "Dallas, GA", "Dalton, GA", "Damascus, GA",
"Danburg, GA", "Danielsville, GA", "Danville, GA", "Darien, GA",
"Davisboro, GA", "De Soto, GA", "Dearing, GA", "Decatur, GA",
"Deepstep, GA", "Demorest, GA", "Denton, GA", "Devereux, GA",
"Dewy Rose, GA", "Dexter, GA", "Dixie, GA", "Doerun, GA",
"Donalsonville, GA", "Doraville, GA", "Douglas, GA", "Douglasville, GA",
"Dry Branch, GA", "Du Pont, GA", "Dudley, GA", "Duluth, GA",
"Dunwoody, GA", "East Dublin, GA", "East Ellijay, GA", "East Point, GA",
"Eastanollee, GA", "Eastman, GA", "Eatonton, GA", "Edison, GA",
"Elberton, GA", "Elko, GA", "Ellabell, GA", "Ellaville, GA",
"Ellenwood, GA", "Ellerslie, GA", "Ellijay, GA", "Emerson, GA",
"Enigma, GA", "Epworth, GA", "Evans, GA", "Fairburn, GA",
"Fairmount, GA", "Fargo, GA", "Felton, GA", "Fitzgerald, GA",
"Fleming, GA", "Flintstone, GA", "Flovilla, GA", "Flowery Branch, GA",
"Folkston, GA", "Forest Hills, GA", "Forest Park, GA", "Forsyth, GA",
"Fort Gaines, GA", "Fort Gordon, GA", "Fort Oglethorpe, GA",
"Fort Stewart, GA", "Fort Valley, GA", "Fortson, GA", "Gainesville, GA",
"Garden City, GA", "Garfield, GA", "Georgetown, GA",
"Georgia Southwes, GA", "Gibson, GA", "Gillsville, GA", "Girard, GA",
"Glenn, GA", "Glennville, GA", "Glenwood, GA", "Glynco, GA",
"Good Hope, GA", "Grantville, GA", "Graves, GA", "Gray, GA",
"Grayson, GA", "Greensboro, GA", "Griffin, GA", "Grovetown, GA",
"Guyton, GA", "Haddock, GA", "Hahira, GA", "Hamilton, GA",
"Hampton, GA", "Hapeville, GA", "Harlem, GA", "Harrison, GA",
"Hartsfield, GA", "Hartwell, GA", "Hawkinsville, GA", "Hazlehurst, GA",
"Helen, GA", "Helena, GA", "Hephzibah, GA", "Hiawassee, GA",
"Hill City, GA", "Hiltonia, GA", "Hinesville, GA", "Hiram, GA",
"Hoboken, GA", "Hogansville, GA", "Homer, GA", "Hortense, GA",
"Hoschton, GA", "Huber, GA", "Hull, GA", "Ideal, GA", "Iron City, GA",
"Irwinton, GA", "Irwinville, GA", "Jackson, GA", "Jacksonville, GA",
"Jakin, GA", "Jasper, GA", "Jefferson, GA", "Jeffersonville, GA",

"Jekyll Island, GA", "Jenkinsburg, GA", "Jesup, GA", "Jewell, GA",
"Jonesboro, GA", "Juliette, GA", "Junction City, GA", "Juniper, GA",
"Juno, GA", "Kathleen, GA", "Kennesaw, GA", "Keysville, GA",
"Kingsland, GA", "Kingston, GA", "Kite, GA", "Knoxville, GA",
"La Fayette, GA", "La Grange, GA", "Lake Park, GA", "Lakeland, GA",
"Lakemont, GA", "Lavonia, GA", "Lawrenceville, GA", "Leary, GA",
"Leesburg, GA", "Lenox, GA", "Leslie, GA", "Lexington, GA",
"Lilburn, GA", "Lincolnton, GA", "Lindale, GA", "Lithia Springs, GA",
"Lithonia, GA", "Lizella, GA", "Locust Grove, GA", "Loganville, GA",
"Lookout Mountain, GA", "Louisville, GA", "Ludowici, GA", "Lula, GA",
"Lumber City, GA", "Lumpkin, GA", "Luthersville, GA", "Lyerly, GA",
"Lyons, GA", "M M, GA", "Mableton, GA", "Macon, GA", "Madison, GA",
"Manassas, GA", "Manchester, GA", "Manor, GA", "Mansfield, GA",
"Marble Hill, GA", "Marietta, GA", "Marine Corps Log, GA",
"Marshallville, GA", "Martin, GA", "Martinez, GA", "Matthews, GA",
"Mauk, GA", "Maysville, GA", "Mc Caysville, GA", "Mc Donough, GA",
"Mc Intyre, GA", "Mc Rae, GA", "Meansville, GA", "Meigs, GA",
"Meridian, GA", "Mershon, GA", "Metter, GA", "Midland, GA",
"Midville, GA", "Midway, GA", "Milan, GA", "Milledgeville, GA",
"Millen, GA", "Millwood, GA", "Milner, GA", "Mineral Bluff, GA",
"Mitchell, GA", "Molena, GA", "Monroe, GA", "Montezuma, GA",
"Monticello, GA", "Montrose, GA", "Moreland, GA", "Morgan, GA",
"Morganton, GA", "Morrow, GA", "Morven, GA", "Moultrie, GA",
"Mount Airy, GA", "Mount Vernon, GA", "Murrayville, GA", "Musella, GA",
"Nahunta, GA", "Nashville, GA", "Naylor, GA", "Newborn, GA",
"Newington, GA", "Newnan, GA", "Newton, GA", "Nicholls, GA",
"Nicholson, GA", "Norcross, GA", "Norman Park, GA", "Norwood, GA",
"Oak Park, GA", "Oakfield, GA", "Oakwood, GA", "Ochlocknee, GA",
"Ocilla, GA", "Odum, GA", "Oglethorpe, GA", "Okefenokee, GA",
"Omaha, GA", "Omega, GA", "Oxford, GA", "Palmetto, GA", "Parrott, GA",
"Patterson, GA", "Pavo, GA", "Peach Orchard, GA", "Peachtree City, GA",
"Pearson, GA", "Pelham, GA", "Pembroke, GA", "Pendergrass, GA",
"Perkins, GA", "Perry, GA", "Philomath, GA", "Pine Log, GA",
"Pine Mountain, GA", "Pine Mountain Va, GA", "Pinehurst, GA",
"Pinetta, GA", "Pineview, GA", "Pitts, GA", "Plains, GA",
"Plainville, GA", "Pooler, GA", "Port Wentworth, GA", "Portal, GA",
"Poulan, GA", "Powder Springs, GA", "Powersville, GA", "Preston, GA",
"Quitman, GA", "Rabun Gap, GA", "Ranger, GA", "Ray City, GA",
"Raymond, GA", "Rebecca, GA", "Register, GA", "Reidsville, GA",
"Rentz, GA", "Rex, GA", "Reynolds, GA", "Rhine, GA", "Riceboro, GA",
"Richland, GA", "Richmond Hill, GA", "Rincon, GA", "Ringgold, GA",
"Rising Fawn, GA", "Riverdale, GA", "Roberta, GA", "Robins A F B, GA",
"Rochelle, GA", "Rock Spring, GA", "Rockledge, GA", "Rockmart, GA",
"Rocky Face, GA", "Rocky Ford, GA", "Rome, GA", "Roopville, GA",
"Rossville, GA", "Roswell, GA", "Round Oak, GA", "Royston, GA",
"Rupert, GA", "Rutledge, GA", "Saint George, GA", "Saint Marys, GA",
"Saint Simons Isl, GA", "Sale City, GA", "Sandy Springs, GA",
"Sapelo Island, GA", "Sardis, GA", "Sautee Nacoochee, GA",
"Savannah, GA", "Scottsdale, GA", "Screven, GA", "Senoia, GA",
"Shady Dale, GA", "Sharpsburg, GA", "Shellman, GA", "Shiloh, GA",
"Silver Creek, GA", "Sky Valley, GA", "Smithville, GA", "Smyrna, GA",
"Snellville, GA", "Social Circle, GA", "Soperton, GA", "Sparks, GA",
"Springvale, GA", "Stapleton, GA", "Starrsville, GA",
"State College, GA", "Statenville, GA", "Statesboro, GA", "Statham, GA",
"Stephens, GA", "Stevens Pottery, GA", "Stillwell, GA",

"Stockbridge, GA", "Stockton, GA", "Stone Mountain, GA", "Stovall, GA",
 "Suches, GA", "Sugar Valley, GA", "Summerville, GA", "Sumner, GA",
 "Surrency, GA", "Suwanee, GA", "Sycamore, GA", "Sylvester, GA",
 "Talbotton, GA", "Talking Rock, GA", "Tallapoosa, GA", "Talmo, GA",
 "Tarrytown, GA", "Tate, GA", "Taylorsville, GA", "Tazewell, GA",
 "Temple, GA", "Tennille, GA", "The Rock, GA", "Thomaston, GA",
 "Thomasville, GA", "Thomson, GA", "Tiger, GA", "Toccoa, GA",
 "Toombsboro, GA", "Townsend, GA", "Trenton, GA", "Trion, GA",
 "Tucker, GA", "Tunnel Hill, GA", "Twin City, GA", "Ty Ty, GA",
 "Tybee Island, GA", "Tyrone, GA", "Unadilla, GA", "Union City, GA",
 "Union Point, GA", "Upatoi, GA", "Uvalda, GA", "Vidalia, GA",
 "Vienna, GA", "Villa Rica, GA", "Waco, GA", "Wadley, GA", "Waleska, GA",
 "Warm Springs, GA", "Warner Robins, GA", "Warrenton, GA", "Warthen, GA",
 "Warwick, GA", "Washington, GA", "Watkinsville, GA", "Waverly, GA",
 "Waverly Hall, GA", "Waynesboro, GA", "Waynesville, GA",
 "West Green, GA", "West Point, GA", "Weston, GA", "Whigham, GA",
 "White, GA", "White Oak, GA", "White Plains, GA", "Whitesburg, GA",
 "Wildwood, GA", "Willacoochee, GA", "Williamson, GA",
 "Wilson Airport, GA", "Winder, GA", "Winston, GA", "Winterville, GA",
 "Woodbine, GA", "Woodbury, GA", "Woodland, GA", "Woodstock, GA",
 "Woolsey, GA", "Wray, GA", "Wrens, GA", "Wrightsville, GA",
 "Yatesville, GA", "Young Harris, GA", "Zebulon, GA", "Aiea, HI",
 "Captain Cook, HI", "Eleele, HI", "Ewa Beach, HI", "Haiku, HI",
 "Hakalau, HI", "Haleiwa, HI", "Hana, HI", "Hanapepe, HI", "Hauula, HI",
 "Hawaii National, HI", "Hawi, HI", "Hilo, HI", "Holualoa, HI",
 "Honaunau, HI", "Honokaa, HI", "Honolulu, HI", "Honomu, HI",
 "Hoolehua, HI", "Kaaawa, HI", "Kahului, HI", "Kailua, HI",
 "Kailua Kona, HI", "Kalaupapa, HI", "Kamuela, HI", "Kaneohe, HI",
 "Kapaa, HI", "Kapaau, HI", "Kapolei, HI", "Kaunakani, HI",
 "Kaunakakai, HI", "Keaau, HI", "Kealahou, HI", "Kekaha, HI",
 "Kihei, HI", "Koloa, HI", "Kualapuu, HI", "Kula, HI", "Kurtistown, HI",
 "Lahaina, HI", "Laie, HI", "Lanai City, HI", "Laupahoehoe, HI",
 "Lihue, HI", "Makawao, HI", "Makaweli, HI", "Maunaloa, HI",
 "Mililani, HI", "Mountain View, HI", "Naalehu, HI", "Ninole, HI",
 "Ookala, HI", "Paauhau, HI", "Paauilo, HI", "Pahala, HI", "Pahoa, HI",
 "Paia, HI", "Papaaloa, HI", "Papaikou, HI", "Pearl City, HI",
 "Pepeekeo, HI", "Princeville, HI", "Volcano, HI", "Wahiawa, HI",
 "Waialua, HI", "Waianae, HI", "Wailuku, HI", "Waimanalo, HI",
 "Waimea, HI", "Waipahu, HI", "Ackley, IA", "Ackworth, IA", "Adair, IA",
 "Adel, IA", "Afton, IA", "Agency, IA", "Ainsworth, IA", "Akron, IA",
 "Albert City, IA", "Albia, IA", "Albion, IA", "Alburnett, IA",
 "Alden, IA", "Alexander, IA", "Algona, IA", "Alleman, IA",
 "Allerton, IA", "Allison, IA", "Alta, IA", "Alta Vista, IA",
 "Alton, IA", "Altoona, IA", "Alvord, IA", "Amana, IA", "Ames, IA",
 "Anamosa, IA", "Andover, IA", "Andrew, IA", "Anita, IA", "Ankeny, IA",
 "Anthon, IA", "Aplington, IA", "Arcadia, IA", "Archer, IA",
 "Aredale, IA", "Argyle, IA", "Arion, IA", "Arlington, IA",
 "Armstrong, IA", "Arnolds Park, IA", "Arthur, IA", "Ashton, IA",
 "Aspinwall, IA", "Atalissa, IA", "Atkins, IA", "Atlantic, IA",
 "Audubon, IA", "Aurelia, IA", "Aurora, IA", "Austinville, IA",
 "Avoca, IA", "Ayrshire, IA", "Badger, IA", "Bagley, IA", "Baldwin, IA",
 "Bancroft, IA", "Barnes City, IA", "Barnum, IA", "Batavia, IA",
 "Battle Creek, IA", "Baxter, IA", "Bayard, IA", "Beacon, IA",
 "Beaconsfield, IA", "Beaman, IA", "Beaver, IA", "Bedford, IA",
 "Belle Plaine, IA", "Bellevue, IA", "Belmond, IA", "Bennett, IA",

"Benton, IA", "Bernard, IA", "Bettendorf, IA", "Bevington, IA",
"Big Rock, IA", "Birmingham, IA", "Blairsburg, IA", "Blairstown, IA",
"Blakesburg, IA", "Blanchard, IA", "Blencoe, IA", "Blockton, IA",
"Bloomfield, IA", "Blue Grass, IA", "Bode, IA", "Bonaparte, IA",
"Bondurant, IA", "Boone, IA", "Booneville, IA", "Bouton, IA",
"Boxholm, IA", "Boyden, IA", "Braddyville, IA", "Bradford, IA",
"Bradgate, IA", "Brandon, IA", "Brayton, IA", "Breda, IA", "Bremer, IA",
"Bridgewater, IA", "Brighton, IA", "Bristow, IA", "Britt, IA",
"Bronson, IA", "Brooklyn, IA", "Bryant, IA", "Buckingham, IA",
"Buffalo Center, IA", "Burlington, IA", "Burnside, IA", "Burr Oak, IA",
"Burt, IA", "Bussey, IA", "Calamus, IA", "Callender, IA", "Calmar, IA",
"Calumet, IA", "Camanche, IA", "Cambridge, IA", "Cantril, IA",
"Carlisle, IA", "Carroll, IA", "Carson, IA", "Carter Lake, IA",
"Cascade, IA", "Casey, IA", "Castalia, IA", "Castana, IA", "Cedar, IA",
"Cedar Falls, IA", "Cedar Rapids, IA", "Center Junction, IA",
"Center Point, IA", "Centerville, IA", "Central City, IA",
"Chariton, IA", "Charles City, IA", "Charlotte, IA", "Charter Oak, IA",
"Chelsea, IA", "Cherokee, IA", "Chester, IA", "Chillicothe, IA",
"Churdan, IA", "Cincinnati, IA", "Clare, IA", "Clarence, IA",
"Clarinda, IA", "Clarion, IA", "Clarksville, IA", "Clear Lake, IA",
"Clearfield, IA", "Cleghorn, IA", "Clemons, IA", "Clermont, IA",
"Clinton, IA", "Clio, IA", "Clive, IA", "Clutier, IA", "Coggon, IA",
"Coin, IA", "Colesburg, IA", "Colfax, IA", "College Springs, IA",
"Collins, IA", "Colo, IA", "Columbia, IA", "Columbus Junction, IA",
"Conesville, IA", "Conrad, IA", "Conroy, IA", "Coon Rapids, IA",
"Cooper, IA", "Coralville, IA", "Corning, IA", "Correctionville, IA",
"Corwith, IA", "Council Bluffs, IA", "Crawfordsville, IA",
"Crescent, IA", "Cresco, IA", "Crystal Lake, IA", "Cumberland, IA",
"Cumming, IA", "Curlew, IA", "Cushing, IA", "Cylinder, IA",
"Dallas, IA", "Dallas Center, IA", "Dana, IA", "Danbury, IA",
"Danville, IA", "Davenport, IA", "Dawson, IA", "Dayton, IA",
"De Soto, IA", "De Witt, IA", "Decatur, IA", "Decorah, IA",
"Dedham, IA", "Deep River, IA", "Delaware, IA", "Delhi, IA",
"Delmar, IA", "Deloit, IA", "Delphos, IA", "Delta, IA", "Denison, IA",
"Denmark, IA", "Denver, IA", "Derby, IA", "Des Moines, IA",
"Dexter, IA", "Diagonal, IA", "Dickens, IA", "Dike, IA", "Dolliver, IA",
"Donahue, IA", "Donnellson, IA", "Doon, IA", "Dorchester, IA",
"Douds, IA", "Dougherty, IA", "Dow City, IA", "Dows, IA",
"Drakesville, IA", "Dubuque, IA", "Dumont, IA", "Duncombe, IA",
"Dundee, IA", "Dunkerton, IA", "Durango, IA", "Durant, IA",
"Dyersville, IA", "Dysart, IA", "Eagle Grove, IA", "Earlham, IA",
"Earling, IA", "Earlville, IA", "Early, IA", "Eddyville, IA",
"Edgewood, IA", "Elberon, IA", "Eldon, IA", "Eldora, IA",
"Eldorado, IA", "Eldridge, IA", "Elgin, IA", "Elk Horn, IA",
"Elkader, IA", "Elkhart, IA", "Elkport, IA", "Elliott, IA",
"Ellston, IA", "Ellsworth, IA", "Elma, IA", "Elwood, IA", "Ely, IA",
"Emerson, IA", "Emmetsburg, IA", "Epworth, IA", "Essex, IA",
"Estherville, IA", "Evansdale, IA", "Everly, IA", "Exira, IA",
"Exline, IA", "Fairbank, IA", "Fairfax, IA", "Fairfield, IA",
"Farley, IA", "Farmersburg, IA", "Farmington, IA", "Farnhamville, IA",
"Farragut, IA", "Fayette, IA", "Fenton, IA", "Fertile, IA",
"Floris, IA", "Floyd, IA", "Fonda, IA", "Fontanelle, IA",
"Forest City, IA", "Fort Atkinson, IA", "Fort Dodge, IA",
"Fort Madison, IA", "Fostoria, IA", "Fredericksburg, IA", "Fremont, IA",
"Galt, IA", "Galva, IA", "Garber, IA", "Garden City, IA",

"Garden Grove, IA", "Garnavillo, IA", "Garner, IA", "Garrison, IA",
 "Garwin, IA", "Geneva, IA", "George, IA", "Gibson, IA", "Gilman, IA",
 "Gilmore City, IA", "Gladbrook, IA", "Glenwood, IA", "Glidden, IA",
 "Goldfield, IA", "Goodell, IA", "Goose Lake, IA", "Gowrie, IA",
 "Graettinger, IA", "Grafton, IA", "Grand Junction, IA",
 "Grand Mound, IA", "Grand River, IA", "Granger, IA", "Grant, IA",
 "Granville, IA", "Gravity, IA", "Gray, IA", "Greeley, IA", "Greene, IA",
 "Greenfield, IA", "Greenville, IA", "Grimes, IA", "Grinnell, IA",
 "Griswold, IA", "Grundy Center, IA", "Guernsey, IA",
 "Guthrie Center, IA", "Guttenberg, IA", "Halbur, IA", "Hamburg, IA",
 "Hamilton, IA", "Hamlin, IA", "Hampton, IA", "Hancock, IA",
 "Hanlontown, IA", "Hansell, IA", "Harcourt, IA", "Hardy, IA",
 "Harlan, IA", "Harper, IA", "Harpers Ferry, IA", "Harris, IA",
 "Hartford, IA", "Hartley, IA", "Hartwick, IA", "Harvey, IA",
 "Hastings, IA", "Havelock, IA", "Haverhill, IA", "Hawarden, IA",
 "Hawkeye, IA", "Hazleton, IA", "Hedrick, IA", "Henderson, IA",
 "Hiawatha, IA", "Highland Center, IA", "Hillsboro, IA", "Hinton, IA",
 "Holland, IA", "Holstein, IA", "Holy Cross, IA", "Homestead, IA",
 "Honey Creek, IA", "Hopkinton, IA", "Hornick, IA", "Hospers, IA",
 "Houghton, IA", "Hubbard, IA", "Hudson, IA", "Hull, IA", "Humboldt, IA",
 "Humeston, IA", "Huxley, IA", "Ida Grove, IA", "Imogene, IA",
 "Independence, IA", "Inwood, IA", "Ionia, IA", "Iowa City, IA",
 "Iowa Falls, IA", "Ira, IA", "Ireton, IA", "Irwin, IA",
 "Jackson Junction, IA", "Jamaica, IA", "Janesville, IA",
 "Jefferson, IA", "Jesup, IA", "Jewell, IA", "Johnston, IA", "Joice, IA",
 "Jolley, IA", "Kalona, IA", "Kamrar, IA", "Kanawha, IA",
 "Kellerton, IA", "Kelley, IA", "Kellogg, IA", "Kensett, IA", "Kent, IA",
 "Keokuk, IA", "Keosauqua, IA", "Keota, IA", "Kesley, IA", "Keswick, IA",
 "Keystone, IA", "Kimballton, IA", "Kingsley, IA", "Kinross, IA",
 "Kirkman, IA", "Kirkville, IA", "Kiron, IA", "Klemme, IA",
 "Knierim, IA", "Knoke, IA", "Knoxville, IA", "La Motte, IA",
 "La Porte City, IA", "Lacona, IA", "Ladora, IA", "Lake City, IA",
 "Lake Mills, IA", "Lake Park, IA", "Lake View, IA", "Lakota, IA",
 "Lamoni, IA", "Lamont, IA", "Lanesboro, IA", "Lansing, IA",
 "Larchwood, IA", "Larrabee, IA", "Latimer, IA", "Laurel, IA",
 "Laurens, IA", "Lawler, IA", "Lawton, IA", "Le Claire, IA",
 "Le Mars, IA", "Ledyard, IA", "Lehigh, IA", "Leighton, IA",
 "Leland, IA", "Lenox, IA", "Leon, IA", "Letts, IA", "Lewis, IA",
 "Liberty Center, IA", "Libertyville, IA", "Lidderdale, IA",
 "Lime Springs, IA", "Lincoln, IA", "Linden, IA", "Lineville, IA",
 "Linn Grove, IA", "Lisbon, IA", "Liscomb, IA", "Little Cedar, IA",
 "Little Rock, IA", "Little Sioux, IA", "Livermore, IA", "Lockridge, IA",
 "Logan, IA", "Lohrville, IA", "Lone Rock, IA", "Lone Tree, IA",
 "Long Grove, IA", "Lorimor, IA", "Lost Nation, IA", "Lovilia, IA",
 "Lowden, IA", "Lu Verne, IA", "Luana, IA", "Lucas, IA", "Luther, IA",
 "Luzerne, IA", "Lynnville, IA", "Lytton, IA", "Macedonia, IA",
 "Macksburg, IA", "Madrid, IA", "Magnolia, IA", "Malcom, IA",
 "Mallard, IA", "Maloy, IA", "Malvern, IA", "Manawa, IA",
 "Manchester, IA", "Manilla, IA", "Manly, IA", "Manning, IA",
 "Manson, IA", "Mapleton, IA", "Maquoketa, IA", "Marathon, IA",
 "Marble Rock, IA", "Marcus, IA", "Marengo, IA", "Marion, IA",
 "Marne, IA", "Marquette, IA", "Marshalltown, IA", "Martelle, IA",
 "Mason City, IA", "Masonville, IA", "Massena, IA", "Maurice, IA",
 "Maxwell, IA", "Maynard, IA", "Mc Callsburg, IA", "Mc Clelland, IA",
 "Mc Gregor, IA", "Mc Intire, IA", "Mechanicsville, IA",

"Mediapolis, IA", "Melbourne, IA", "Melcher-Dallas, IA", "Melrose, IA",
"Melvin, IA", "Menlo, IA", "Meriden, IA", "Merrill, IA", "Meservey, IA",
"Middle Amana, IA", "Middletown, IA", "Miles, IA", "Milford, IA",
"Millersburg, IA", "Millerton, IA", "Milo, IA", "Milton, IA",
"Minburn, IA", "Minden, IA", "Mineola, IA", "Mingo, IA",
"Missouri Valley, IA", "Mitchellville, IA", "Modale, IA",
"Mondamin, IA", "Monmouth, IA", "Monona, IA", "Monroe, IA",
"Montezuma, IA", "Monticello, IA", "Montour, IA", "Montrose, IA",
"Moorhead, IA", "Moorland, IA", "Moravia, IA", "Morning Sun, IA",
"Moscow, IA", "Moulton, IA", "Mount Auburn, IA", "Mount Ayr, IA",
"Mount Pleasant, IA", "Mount Sterling, IA", "Mount Union, IA",
"Mount Vernon, IA", "Moville, IA", "Murray, IA", "Muscatine, IA",
"Mystic, IA", "Nashua, IA", "Nemaha, IA", "Neola, IA", "Nevada, IA",
"Nevinville, IA", "New Albin, IA", "New Hampton, IA",
"New Hartford, IA", "New Liberty, IA", "New London, IA",
"New Market, IA", "New Providence, IA", "New Sharon, IA",
"New Vienna, IA", "New Virginia, IA", "Newell, IA", "Newhall, IA",
"Newton, IA", "Nichols, IA", "Nodaway, IA", "Nora Springs, IA",
"North Buena Vist, IA", "North English, IA", "North Liberty, IA",
"Northboro, IA", "Northwood, IA", "Norwalk, IA", "Norway, IA",
"Numa, IA", "Oakland, IA", "Oakville, IA", "Ocheyedan, IA",
"Odebolt, IA", "Oelwein, IA", "Ogden, IA", "Okobojo, IA", "Olds, IA",
"Olin, IA", "Ollie, IA", "Onawa, IA", "Onslow, IA", "Orange City, IA",
"Orchard, IA", "Orient, IA", "Osage, IA", "Osceola, IA",
"Oskaloosa, IA", "Ossian, IA", "Otho, IA", "Otley, IA", "Oto, IA",
"Ottosen, IA", "Oxford, IA", "Oxford Junction, IA",
"Pacific Junction, IA", "Packwood, IA", "Palmer, IA", "Palo, IA",
"Panama, IA", "Panora, IA", "Parkersburg, IA", "Parnell, IA",
"Paton, IA", "Patterson, IA", "Paullina, IA", "Pella, IA", "Peosta, IA",
"Percival, IA", "Perry, IA", "Persia, IA", "Peru, IA", "Peterson, IA",
"Pierson, IA", "Pilot Mound, IA", "Pisgah, IA", "Plainfield, IA",
"Plano, IA", "Pleasant Hill, IA", "Pleasanton, IA", "Pleasantville, IA",
"Plover, IA", "Plymouth, IA", "Pocahontas, IA", "Polk City, IA",
"Pomeroy, IA", "Popejoy, IA", "Portsmouth, IA", "Postville, IA",
"Prairie City, IA", "Prescott, IA", "Preston, IA", "Primghar, IA",
"Princeton, IA", "Prole, IA", "Promise City, IA", "Pulaski, IA",
"Quasqueton, IA", "Quimby, IA", "Radcliffe, IA", "Rake, IA",
"Ralston, IA", "Randalia, IA", "Randall, IA", "Randolph, IA",
"Raymond, IA", "Readlyn, IA", "Reasnor, IA", "Red Oak, IA",
"Redding, IA", "Redfield, IA", "Reinbeck, IA", "Rembrandt, IA",
"Remsen, IA", "Renwick, IA", "Rhodes, IA", "Riceville, IA",
"Richland, IA", "Ricketts, IA", "Ridgeway, IA", "Ringsted, IA",
"Rippey, IA", "Riverside, IA", "Riverton, IA", "Robins, IA",
"Rock Falls, IA", "Rock Rapids, IA", "Rock Valley, IA", "Rockford, IA",
"Rockwell, IA", "Rockwell City, IA", "Rodman, IA", "Rodney, IA",
"Roland, IA", "Rolfe, IA", "Rose Hill, IA", "Rowan, IA", "Rowley, IA",
"Royal, IA", "Rudd, IA", "Runnells, IA", "Russell, IA", "Ruthven, IA",
"Rutland, IA", "Ryan, IA", "Sabula, IA", "Sac City, IA",
"Saint Ansgar, IA", "Saint Anthony, IA", "Saint Charles, IA",
"Saint Donatus, IA", "Saint Lucas, IA", "Saint Marys, IA",
"Saint Olaf, IA", "Saint Paul, IA", "Salem, IA", "Salix, IA",
"Sanborn, IA", "Scarville, IA", "Schaller, IA", "Schleswig, IA",
"Scotch Grove, IA", "Scranton, IA", "Searsboro, IA", "Selma, IA",
"Sergeant Bluff, IA", "Sewal, IA", "Seymour, IA", "Shambaugh, IA",
"Shannon City, IA", "Sharpsburg, IA", "Sheffield, IA", "Shelby, IA",

"Sheldon, IA", "Shell Rock, IA", "Shellsburg, IA", "Shenandoah, IA",
 "Sherrill, IA", "Sibley, IA", "Sidney, IA", "Sigourney, IA",
 "Silver City, IA", "Sioux Center, IA", "Sioux City, IA",
 "Sioux Rapids, IA", "Slater, IA", "Sloan, IA", "Smithland, IA",
 "Soldier, IA", "Solon, IA", "Somers, IA", "South Amana, IA",
 "South English, IA", "Spencer, IA", "Sperry, IA", "Spirit Lake, IA",
 "Spragueville, IA", "Spring Hill, IA", "Springbrook, IA",
 "Springville, IA", "Stacyville, IA", "Stanhope, IA", "Stanley, IA",
 "Stanton, IA", "Stanwood, IA", "State Center, IA", "Steamboat Rock, IA",
 "Stockport, IA", "Stockton, IA", "Storm Lake, IA", "Story City, IA",
 "Stratford, IA", "Strawberry Point, IA", "Stuart, IA", "Sully, IA",
 "Sumner, IA", "Superior, IA", "Sutherland, IA", "Swaledale, IA",
 "Swan, IA", "Swea City, IA", "Swisher, IA", "Tabor, IA", "Tama, IA",
 "Templeton, IA", "Terril, IA", "Thayer, IA", "Thompson, IA", "Thor, IA",
 "Thornton, IA", "Thurman, IA", "Tingley, IA", "Tipton, IA",
 "Titonka, IA", "Toddville, IA", "Toledo, IA", "Toronto, IA",
 "Tracy, IA", "Traer, IA", "Treynor, IA", "Tripoli, IA", "Truro, IA",
 "Turin, IA", "Udell, IA", "Underwood, IA", "Union, IA",
 "Unionville, IA", "Urbandale, IA", "Ute, IA", "Vail, IA",
 "Van Horne, IA", "Van Meter, IA", "Van Wert, IA", "Ventura, IA",
 "Victor, IA", "Villisca, IA", "Vincent, IA", "Vining, IA", "Vinton, IA",
 "Volga, IA", "Wadena, IA", "Walcott, IA", "Walker, IA", "Wall Lake, IA",
 "Wallingford, IA", "Walnut, IA", "Wapello, IA", "Washburn, IA",
 "Washington, IA", "Washta, IA", "Waterloo, IA", "Waterville, IA",
 "Watkins, IA", "Waucoma, IA", "Waukee, IA", "Waukon, IA", "Wayland, IA",
 "Webb, IA", "Webster, IA", "Webster City, IA", "Weldon, IA",
 "Wellman, IA", "Wellsburg, IA", "Welton, IA", "Wesley, IA",
 "West Amana, IA", "West Bend, IA", "West Branch, IA",
 "West Burlington, IA", "West Chester, IA", "West Des Moines, IA",
 "West Grove, IA", "West Liberty, IA", "West Point, IA", "Westfield, IA",
 "Westgate, IA", "Westphalia, IA", "Westside, IA", "Wever, IA",
 "What Cheer, IA", "Wheatland, IA", "Whiting, IA", "Whittemore, IA",
 "Williams, IA", "Williamsburg, IA", "Williamson, IA", "Wilton, IA",
 "Windsor Heights, IA", "Winfield, IA", "Winterset, IA", "Winthrop, IA",
 "Wiota, IA", "Woden, IA", "Woodbine, IA", "Woodburn, IA",
 "Woodward, IA", "Woolstock, IA", "Worthington, IA", "Wyoming, IA",
 "Yale, IA", "Yarmouth, IA", "Yetter, IA", "Yorktown, IA", "Zearing, IA",
 "Zwingle, IA", "Acequia, ID", "Ahsahka, ID", "American Falls, ID",
 "Ammon, ID", "Arbon, ID", "Arco, ID", "Arimo, ID", "Ashton, ID",
 "Athol, ID", "Atlanta, ID", "Avery, ID", "Bancroft, ID", "Banks, ID",
 "Bayview, ID", "Bellevue, ID", "Bern, ID", "Blackfoot, ID",
 "Blanchard, ID", "Bliss, ID", "Boise, ID", "Bonners Ferry, ID",
 "Buhl, ID", "Burley, ID", "Calder, ID", "Caldwell, ID", "Cambridge, ID",
 "Carey, ID", "Careywood, ID", "Carmen, ID", "Castleford, ID",
 "Cataldo, ID", "Challis, ID", "Chubbuck, ID", "Clark Fork, ID",
 "Clarkia, ID", "Clayton, ID", "Clearwater, ID", "Clifton, ID",
 "Cocolalla, ID", "Coeur D Alene, ID", "Conda, ID", "Coolin, ID",
 "Corral, ID", "Cottonwood, ID", "Council, ID", "Craigmont, ID",
 "Culdesac, ID", "Darlington, ID", "Dayton, ID", "Deary, ID",
 "Declo, ID", "Desmet, ID", "Dietrich, ID", "Dixie, ID", "Donnelly, ID",
 "Downey, ID", "Driggs, ID", "Dubois, ID", "Eagle, ID", "Eden, ID",
 "Elba, ID", "Elk River, ID", "Ellis, ID", "Fairfield, ID", "Felt, ID",
 "Ferdinand, ID", "Fernwood, ID", "Filer, ID", "Firth, ID",
 "Fish Haven, ID", "Fort Hall, ID", "Franklin, ID", "Fruitland, ID",
 "Garden City, ID", "Garden Valley, ID", "Genesee, ID", "Geneva, ID",

"Gibbonsville, ID", "Glenns Ferry, ID", "Gooding, ID", "Grace, ID",
 "Grand View, ID", "Grangeville, ID", "Grasmere, ID", "Greencreek, ID",
 "Hagerman, ID", "Hailey, ID", "Hamer, ID", "Hammett, ID", "Hansen, ID",
 "Harrison, ID", "Harvard, ID", "Hayden Lake, ID", "Hazelton, ID",
 "Heyburn, ID", "Holbrook, ID", "Homedale, ID", "Hope, ID",
 "Horseshoe Bend, ID", "Idaho City, ID", "Idaho Falls, ID",
 "Indian Valley, ID", "Inkom, ID", "Iona, ID", "Island Park, ID",
 "Jerome, ID", "Juliaetta, ID", "Kamiah, ID", "Kellogg, ID",
 "Kendrick, ID", "Keuterville, ID", "Kimberly, ID", "King Hill, ID",
 "Kingston, ID", "Kuna, ID", "Lapwai, ID", "Lava Hot Springs, ID",
 "Leadore, ID", "Lenore, ID", "Letha, ID", "Lewisville, ID",
 "Lowman, ID", "Lucile, ID", "Mackay, ID", "Malad City, ID",
 "Marsing, ID", "Mc Call, ID", "Mc Cammon, ID", "Medimont, ID",
 "Melba, ID", "Menan, ID", "Meridian, ID", "Mesa, ID", "Middleton, ID",
 "Midvale, ID", "Minidoka, ID", "Montevue, ID", "Montour, ID",
 "Montpelier, ID", "Moore, ID", "Moscow, ID", "Mountain Home, ID",
 "Mountain Home A, ID", "Moyie Springs, ID", "Mullan, ID",
 "Murtaugh, ID", "Naf, ID", "Nampa, ID", "Naples, ID", "New Meadows, ID",
 "New Plymouth, ID", "Newdale, ID", "Nezperce, ID", "Nordman, ID",
 "North Fork, ID", "Oakley, ID", "Obsidian, ID", "Ola, ID",
 "Old Town, ID", "Oreana, ID", "Orofino, ID", "Ovid, ID", "Paris, ID",
 "Parma, ID", "Patterson, ID", "Paul, ID", "Payette, ID", "Peck, ID",
 "Picabo, ID", "Pierce, ID", "Pinehurst, ID", "Pingree, ID",
 "Plummer, ID", "Pocatello, ID", "Pollock, ID", "Porthill, ID",
 "Post Falls, ID", "Potlatch, ID", "Preston, ID", "Priest River, ID",
 "Princeton, ID", "Rathdrum, ID", "Reubens, ID", "Rexburg, ID",
 "Richfield, ID", "Rigby, ID", "Riggins, ID", "Ririe, ID", "Roberts, ID",
 "Rockland, ID", "Rogerson, ID", "Sagle, ID", "Saint Anthony, ID",
 "Saint Charles, ID", "Saint Maries, ID", "Salmon, ID", "Sandpoint, ID",
 "Shelley, ID", "Shoshone, ID", "Shoup, ID", "Smelterville, ID",
 "Soda Springs, ID", "South Gate Plaza, ID", "Spencer, ID",
 "Spirit Lake, ID", "Stanley, ID", "Star, ID", "Sterling, ID",
 "Stone, ID", "Sugar City, ID", "Swan Valley, ID", "Sweet, ID",
 "Tensed, ID", "Terreton, ID", "Teton, ID", "Tetonia, ID",
 "Thatcher, ID", "Troy, ID", "Twin Falls, ID", "Victor, ID", "Viola, ID",
 "Wallace, ID", "Wayan, ID", "Weippe, ID", "Weiser, ID", "Wendell, ID",
 "West Mountain, ID", "Weston, ID", "White Bird, ID", "Wilder, ID",
 "Winchester, ID", "Worley, ID", "Yellow Pine, ID", "Abbott Park, IL",
 "Abingdon, IL", "Adair, IL", "Addison, IL", "Albany, IL", "Albers, IL",
 "Albion, IL", "Aledo, IL", "Alexis, IL", "Alhambra, IL", "Allen, IL",
 "Allendale, IL", "Allerton, IL", "Alma, IL", "Alorton, IL", "Alpha, IL",
 "Alsip, IL", "Altamont, IL", "Alto Pass, IL", "Alton, IL", "Altona, IL",
 "Alvin, IL", "Amboy, IL", "Amf Ohare, IL", "Anchor, IL", "Ancona, IL",
 "Andalusia, IL", "Andrew, IL", "Anna, IL", "Annapolis, IL",
 "Annawan, IL", "Antioch, IL", "Apple River, IL", "Arcadia, IL",
 "Arcola, IL", "Arenzville, IL", "Argo, IL", "Argonne, IL",
 "Arlington, IL", "Arlington Height, IL", "Armington, IL",
 "Armstrong, IL", "Aroma Park, IL", "Arrowsmith, IL", "Arthur, IL",
 "Ashkum, IL", "Ashley, IL", "Ashmore, IL", "Ashton, IL",
 "Assumption, IL", "Astoria, IL", "Atkinson, IL", "Atlanta, IL",
 "Atterbury, IL", "Atwater, IL", "Atwood, IL", "Auburn, IL",
 "Augusta, IL", "Aurora, IL", "Ava, IL", "Avon, IL", "Bader, IL",
 "Baileyville, IL", "Baldwin, IL", "Barclay, IL", "Bardolph, IL",
 "Barnhill, IL", "Barr, IL", "Barrington, IL", "Barry, IL",
 "Bartelso, IL", "Bartonville, IL", "Basco, IL", "Batavia, IL",

"Batchtown, IL", "Bates, IL", "Baylis, IL", "Beardstown, IL",
"Bearsdale, IL", "Beason, IL", "Beaverville, IL", "Bedford Park, IL",
"Beecher, IL", "Beecher City, IL", "Belknap, IL", "Belle Rive, IL",
"Belleville, IL", "Bellflower, IL", "Bellwood, IL", "Belvidere, IL",
"Bement, IL", "Benld, IL", "Bensenville, IL", "Benson, IL",
"Benton, IL", "Berry, IL", "Berwick, IL", "Bethalto, IL", "Bethany, IL",
"Bible Grove, IL", "Big Rock, IL", "Biggs, IL", "Biggsville, IL",
"Bingham, IL", "Birds, IL", "Bismarck, IL", "Blackstone, IL",
"Blandinsville, IL", "Bloomington, IL", "Bloomington, IL",
"Blue Island, IL", "Blue Mound, IL", "Bluford, IL", "Bolingbrook, IL",
"Bolivia, IL", "Bone Gap, IL", "Bonfield, IL", "Bonnie, IL",
"Boody, IL", "Bourbonnais, IL", "Bowen, IL", "Braceville, IL",
"Bradford, IL", "Bradley, IL", "Braidwood, IL", "Breese, IL",
"Bridgeport, IL", "Bridgeview, IL", "Brighton, IL", "Brimfield, IL",
"Bristol, IL", "Broadlands, IL", "Broadview, IL", "Broadwell, IL",
"Brocton, IL", "Brookfield, IL", "Broughton, IL", "Brownfield, IL",
"Browns, IL", "Brownstown, IL", "Brunswick, IL", "Bryant, IL",
"Buckingham, IL", "Buckley, IL", "Buckner, IL", "Buda, IL",
"Buffalo Grove, IL", "Buffalo Hart, IL", "Buncombe, IL",
"Bunker Hill, IL", "Burbank, IL", "Burnham, IL", "Burnside, IL",
"Burnt Prairie, IL", "Bushnell, IL", "Butler, IL", "Byron, IL",
"Cabery, IL", "Cache, IL", "Cahokia, IL", "Cairo, IL", "Caledonia, IL",
"Calhoun, IL", "Calumet City, IL", "Calumet Park, IL", "Camargo, IL",
"Cambridge, IL", "Camden, IL", "Cameron, IL", "Camp Point, IL",
"Campbell Hill, IL", "Canton, IL", "Cantrall, IL", "Capron, IL",
"Carbondale, IL", "Carlock, IL", "Carlyle, IL", "Carman, IL",
"Carmi, IL", "Carol Stream, IL", "Carpentersville, IL",
"Carrier Mills, IL", "Carrollton, IL", "Carterville, IL",
"Carthage, IL", "Cary, IL", "Casey, IL", "Caseyville, IL", "Casner, IL",
"Catlin, IL", "Cave In Rock, IL", "Cazenovia, IL", "Centralia, IL",
"Cerro Gordo, IL", "Chadwick, IL", "Chambersburg, IL", "Champaign, IL",
"Chana, IL", "Channahon, IL", "Chapin, IL", "Charleston, IL",
"Chatham, IL", "Chatsworth, IL", "Chebanse, IL", "Chenoa, IL",
"Cherry Valley, IL", "Chester, IL", "Chestnut, IL", "Chicago, IL",
"Chicago Ridge, IL", "Chillicothe, IL", "Chrisman, IL",
"Christopher, IL", "Cicero, IL", "Cimic, IL", "Cisco, IL", "Cisne, IL",
"Cissna Park, IL", "Clare, IL", "Claremont, IL", "Clarendon Hills, IL",
"Clarksburg, IL", "Clarksdale, IL", "Clay City, IL", "Clayton, IL",
"Clements, IL", "Clifton, IL", "Clinton, IL", "Coal City, IL",
"Coal Valley, IL", "Coatsburg, IL", "Cobden, IL", "Coffeen, IL",
"Colchester, IL", "Colfax, IL", "Collinsville, IL", "Collison, IL",
"Colmar, IL", "Columbia, IL", "Comer, IL", "Compton, IL", "Concord, IL",
"Congerville, IL", "Cooksville, IL", "Cordova, IL", "Cottage Hills, IL",
"Country Club Hil, IL", "Cowden, IL", "Creal Springs, IL",
"Crescent City, IL", "Crestwood, IL", "Crete, IL", "Cropsey, IL",
"Crossville, IL", "Crystal Lake, IL", "Cuba, IL", "Cullom, IL",
"Custer Park, IL", "Cutler, IL", "Cypress, IL", "Dahinda, IL",
"Dahlgren, IL", "Dakota, IL", "Dale, IL", "Dalton City, IL",
"Dalzell, IL", "Dana, IL", "Danforth, IL", "Danvers, IL",
"Danville, IL", "Davis, IL", "Davis Junction, IL", "Dawson, IL",
"De Kalb, IL", "De Land, IL", "De Soto, IL", "Decatur, IL",
"Deer Creek, IL", "Deer Grove, IL", "Deerfield, IL", "Delavan, IL",
"Dennison, IL", "Des Plaines, IL", "Detroit, IL", "Dewey, IL",
"Dewitt, IL", "Dieterich, IL", "Dix, IL", "Dixon, IL", "Dollville, IL",
"Dolton, IL", "Dongola, IL", "Donnellson, IL", "Donovan, IL",

"Dorchester, IL", "Dorsey, IL", "Dow, IL", "Downers Grove, IL",
 "Du Bois, IL", "Du Quoin, IL", "Dundas, IL", "Dundee, IL",
 "Dunfermline, IL", "Dunkel, IL", "Dunlap, IL", "Dupo, IL", "Durand, IL",
 "Dwight, IL", "Earlville, IL", "East Alton, IL", "East Carondelet, IL",
 "East Dubuque, IL", "East Moline, IL", "East Peoria, IL",
 "East Saint Louis, IL", "Eckard, IL", "Eddyville, IL", "Edelstein, IL",
 "Edinburg, IL", "Edwards, IL", "Edwardsville, IL", "Effingham, IL",
 "Egan, IL", "El Paso, IL", "Elburn, IL", "Eldorado, IL", "Eldred, IL",
 "Elgin, IL", "Elizabeth, IL", "Elizabethtown, IL",
 "Elk Grove Villag, IL", "Elkville, IL", "Ellery, IL", "Ellis Grove, IL",
 "Ellisville, IL", "Ellsworth, IL", "Elmhurst, IL", "Elmwood, IL",
 "Elmwood Park, IL", "Elsah, IL", "Elvaston, IL", "Elwood, IL",
 "Emden, IL", "Emington, IL", "Enfield, IL", "Equality, IL", "Erie, IL",
 "Esmond, IL", "Essex, IL", "Eureka, IL", "Evanston, IL",
 "Evansville, IL", "Evergreen Park, IL", "Ewing, IL", "Exeter, IL",
 "Fairbury, IL", "Fairfield, IL", "Fairmount, IL", "Fairview, IL",
 "Fairview Heights, IL", "Fancy Prairie, IL", "Farina, IL",
 "Farmer City, IL", "Farmingdale, IL", "Fenton, IL", "Fiatt, IL",
 "Fidelity, IL", "Fieldon, IL", "Fillmore, IL", "Fisher, IL",
 "Fithian, IL", "Flanagan, IL", "Flat Rock, IL", "Flora, IL",
 "Flossmoor, IL", "Foosland, IL", "Forest City, IL", "Forest Park, IL",
 "Forrest, IL", "Forreston, IL", "Fort Sheridan, IL", "Fowler, IL",
 "Fox Lake, IL", "Fox River Grove, IL", "Frankfort, IL",
 "Frankfort Height, IL", "Franklin Grove, IL", "Franklin Park, IL",
 "Frederick, IL", "Freeburg, IL", "Freeport, IL", "Fulton, IL",
 "Fults, IL", "Gages Lake, IL", "Galatia, IL", "Gale, IL", "Galena, IL",
 "Galesburg, IL", "Galva, IL", "Garden Prairie, IL", "Gardner, IL",
 "Gays, IL", "Geff, IL", "Geneseo, IL", "Geneva, IL", "Genoa, IL",
 "Georgetown, IL", "Gerlaw, IL", "German Valley, IL", "Germantown, IL",
 "Gibson City, IL", "Gifford, IL", "Gilberts, IL", "Gilman, IL",
 "Gilson, IL", "Gladstone, IL", "Glasford, IL", "Glasgow, IL",
 "Glen Carbon, IL", "Glen Ellyn, IL", "Glenarm, IL", "Glencoe, IL",
 "Glendale Heights, IL", "Glenview, IL", "Glenview Nas, IL",
 "Glenwood, IL", "Godfrey, IL", "Golden, IL", "Golden Eagle, IL",
 "Golden Gate, IL", "Good Hope, IL", "Goreville, IL", "Gorham, IL",
 "Grafton, IL", "Grand Chain, IL", "Grand Ridge, IL", "Grand Tower, IL",
 "Grandview, IL", "Grant Park, IL", "Grantsburg, IL", "Granville, IL",
 "Graymont, IL", "Grayville, IL", "Great Lakes, IL", "Green Rock, IL",
 "Green Valley, IL", "Greenfield, IL", "Greenville, IL", "Gridley, IL",
 "Griggsville, IL", "Gurnee, IL", "Hagaman, IL", "Hamburg, IL",
 "Hamel, IL", "Hamilton, IL", "Hammond, IL", "Hampshire, IL",
 "Hampton, IL", "Hanna City, IL", "Hanover, IL", "Hanover Park, IL",
 "Hardin, IL", "Harmon, IL", "Harrisburg, IL", "Hartford, IL",
 "Hartsburg, IL", "Harvard, IL", "Harvel, IL", "Harwood Heights, IL",
 "Hazel Crest, IL", "Hazel Dell, IL", "Hebron, IL", "Hecker, IL",
 "Heman, IL", "Hennepin, IL", "Henry, IL", "Herald, IL", "Herod, IL",
 "Herrick, IL", "Herrin, IL", "Herscher, IL", "Hervey City, IL",
 "Hettick, IL", "Hewittsville, IL", "Heyworth, IL", "Hickory Hills, IL",
 "Hidalgo, IL", "Highland, IL", "Highland Park, IL", "Highwood, IL",
 "Hillsboro, IL", "Hillsdale, IL", "Hillside, IL", "Hillview, IL",
 "Hinckley, IL", "Hindsboro, IL", "Hines, IL", "Hodgkins, IL",
 "Hoffman Estates, IL", "Holder, IL", "Homer, IL", "Hometown, IL",
 "Homewood, IL", "Hoopeston, IL", "Hopedale, IL", "Hoyleton, IL",
 "Hubly, IL", "Hudson, IL", "Hull, IL", "Humboldt, IL", "Hume, IL",
 "Huntley, IL", "Huntsville, IL", "Hutsonville, IL", "Illinois City, IL",

"Illiopopolis, IL", "Ina, IL", "Indianola, IL", "Industry, IL",
 "Ingleside, IL", "Ingraham, IL", "Ipava, IL", "Irving, IL",
 "Island Lake, IL", "Itasca, IL", "Iuka, IL", "Ivesdale, IL",
 "Jacob, IL", "Jerome, IL", "Jerseyville, IL", "Jewett, IL",
 "Johnsonville, IL", "Johnston City, IL", "Joliet, IL", "Jonesboro, IL",
 "Joppa, IL", "Joy, IL", "Junction, IL", "Justice, IL", "Kampsville, IL",
 "Kane, IL", "Kankakee, IL", "Kansas, IL", "Karbers Ridge, IL",
 "Karnak, IL", "Keenes, IL", "Keithsburg, IL", "Kell, IL", "Kempton, IL",
 "Kenilworth, IL", "Kenney, IL", "Kent, IL", "Kewanee, IL",
 "Keyesport, IL", "Kilbourne, IL", "Kinderhook, IL", "Kings, IL",
 "Kingston, IL", "Kingston Mines, IL", "Kinmundy, IL", "Kinsman, IL",
 "Kirkland, IL", "Kirkwood, IL", "Knoxville, IL", "La Fayette, IL",
 "La Harpe, IL", "La Moille, IL", "La Prairie, IL", "La Salle, IL",
 "Laclede, IL", "Lacon, IL", "Lake Bluff, IL", "Lake Forest, IL",
 "Lake In The Hill, IL", "Lakewood, IL", "Lanark, IL", "Lancaster, IL",
 "Lansing, IL", "Latham, IL", "Laura, IL", "Lawrenceville, IL",
 "Layton, IL", "Le Roy, IL", "Lebanon, IL", "Lee, IL", "Leland, IL",
 "Lena, IL", "Lenzburg, IL", "Leonore, IL", "Lerna, IL", "Lewistown, IL",
 "Lexington, IL", "Liberty, IL", "Libertyville, IL", "Lima, IL",
 "Lincoln, IL", "Lincolnwood, IL", "Lindenhurst, IL", "Lindenwood, IL",
 "Lisle, IL", "Litchfield, IL", "Little Indian, IL", "Little York, IL",
 "Littleton, IL", "Liverpool, IL", "Loami, IL", "Loda, IL", "Lomax, IL",
 "Lombard, IL", "London Mills, IL", "Long Grove, IL", "Long Point, IL",
 "Longview, IL", "Lorraine, IL", "Lostant, IL", "Loves Park, IL",
 "Lovington, IL", "Ludlow, IL", "Luther, IL", "Lynchburg, IL",
 "Lyndon, IL", "Lynn Center, IL", "Lyons, IL", "Macedonia, IL",
 "Mackinaw, IL", "Macomb, IL", "Macon, IL", "Madison, IL",
 "Maeystown, IL", "Magnolia, IL", "Mahomet, IL", "Makanda, IL",
 "Malden, IL", "Malta, IL", "Manhattan, IL", "Manito, IL",
 "Mansfield, IL", "Manteno, IL", "Manville, IL", "Maple Park, IL",
 "Mapleton, IL", "Maquon, IL", "Marengo, IL", "Marietta, IL",
 "Marine, IL", "Marion, IL", "Marissa, IL", "Markham, IL", "Maroa, IL",
 "Marseilles, IL", "Marshall, IL", "Martinsville, IL", "Martinton, IL",
 "Mascoutah, IL", "Mason, IL", "Matherville, IL", "Matteson, IL",
 "Mattoon, IL", "Mazon, IL", "Mc Clure, IL", "Mc Connell, IL",
 "Mc Gaw Park, IL", "Mc Henry, IL", "Mc Lean, IL", "Mc Leansboro, IL",
 "Mc Nabb, IL", "Mcvey, IL", "Media, IL", "Medinah, IL", "Medora, IL",
 "Melrose Park, IL", "Melvin, IL", "Mendon, IL", "Mendota, IL",
 "Meppen, IL", "Merrionette Park, IL", "Metamora, IL", "Metcalf, IL",
 "Metropolis, IL", "Michael, IL", "Middlegrove, IL", "Middletown, IL",
 "Milan, IL", "Milford, IL", "Mill Shoals, IL", "Millcreek, IL",
 "Milledgeville, IL", "Miller City, IL", "Millstadt, IL", "Milmine, IL",
 "Milton, IL", "Mineral, IL", "Minier, IL", "Minonk, IL", "Minooka, IL",
 "Mitchell, IL", "Modesto, IL", "Modoc, IL", "Mokena, IL", "Moline, IL",
 "Momence, IL", "Monee, IL", "Monmouth, IL", "Monroe Center, IL",
 "Montgomery, IL", "Monticello, IL", "Montrose, IL", "Mooseheart, IL",
 "Moro, IL", "Morris, IL", "Morrison, IL", "Morrisonville, IL",
 "Morton, IL", "Morton Grove, IL", "Mound City, IL", "Mounds, IL",
 "Mount Auburn, IL", "Mount Carmel, IL", "Mount Carroll, IL",
 "Mount Erie, IL", "Mount Morris, IL", "Mount Olive, IL",
 "Mount Prospect, IL", "Mount Pulaski, IL", "Mount Sterling, IL",
 "Mount Vernon, IL", "Mozier, IL", "Mulberry Grove, IL",
 "Mulkeytown, IL", "Mundelein, IL", "Murphysboro, IL", "Naperville, IL",
 "Naples, IL", "Nashville, IL", "Nason, IL", "Nebo, IL", "Neoga, IL",
 "Neponset, IL", "New Athens, IL", "New Baden, IL", "New Bedford, IL",

"New Boston, IL", "New Burnside, IL", "New Canton, IL",
"New Douglas, IL", "New Haven, IL", "New Holland, IL", "New Lenox, IL",
"New Liberty, IL", "New Salem, IL", "New Windsor, IL", "Newark, IL",
"Newburg, IL", "Newman, IL", "Newmansville, IL", "Newton, IL",
"Niantic, IL", "Nilwood, IL", "Niota, IL", "Noble, IL", "Nokomis, IL",
"Normal, IL", "Normandy, IL", "Norridge, IL", "Norris City, IL",
"North Aurora, IL", "North Henderson, IL", "North Riverside, IL",
"Northbrook, IL", "Northfield, IL", "Northlake, IL", "Nortonville, IL",
"Oak Brook, IL", "Oak Forest, IL", "Oak Hill, IL", "Oak Lawn, IL",
"Oak Park, IL", "Oakdale, IL", "Oakford, IL", "Oakland, IL",
"Oakwood, IL", "Oblong, IL", "Oconee, IL", "Odell, IL", "Odin, IL",
"Ogden, IL", "Oglesby, IL", "Ohio, IL", "Okawville, IL", "Olmsted, IL",
"Olney, IL", "Olympia Fields, IL", "Omaha, IL", "Onarga, IL",
"Oneida, IL", "Opdyke, IL", "Oquawka, IL", "Orangeville, IL",
"Oreana, IL", "Oregon, IL", "Orion, IL", "Orland Park, IL",
"Orleans, IL", "Osco, IL", "Oswego, IL", "Ottawa, IL", "Owaneco, IL",
"Ozark, IL", "Palatine, IL", "Palestine, IL", "Paloma, IL",
"Palos Heights, IL", "Palos Hills, IL", "Palos Park, IL",
"Panther Creek, IL", "Paris, IL", "Park Ridge, IL", "Parkersburg, IL",
"Patoka, IL", "Paw Paw, IL", "Paxton, IL", "Payson, IL", "Pearl, IL",
"Pearl City, IL", "Pecatonica, IL", "Pekin, IL", "Penfield, IL",
"Peoria, IL", "Peoria Heights, IL", "Peotone, IL", "Percy, IL",
"Perry, IL", "Peru, IL", "Pesotum, IL", "Philo, IL", "Piasa, IL",
"Pinckneyville, IL", "Piper City, IL", "Pittsburg, IL",
"Pittsfield, IL", "Plainfield, IL", "Plainview, IL", "Plainville, IL",
"Plano, IL", "Pleasant Hill, IL", "Pocahontas, IL", "Polo, IL",
"Pomona, IL", "Pontiac, IL", "Pontoosuc, IL", "Poplar Grove, IL",
"Port Byron, IL", "Posen, IL", "Potomac, IL", "Prairie City, IL",
"Prairie Du Roche, IL", "Prairie View, IL", "Princeton, IL",
"Princeville, IL", "Prophetstown, IL", "Prospect Heights, IL",
"Pulaski, IL", "Putnam, IL", "Quincy, IL", "Radford, IL", "Raleigh, IL",
"Ramsey, IL", "Rankin, IL", "Ransom, IL", "Rantoul, IL", "Raritan, IL",
"Raymond, IL", "Red Bud, IL", "Reddick, IL", "Renault, IL",
"Reynolds, IL", "Richmond, IL", "Richton Park, IL", "Richview, IL",
"Ridge Farm, IL", "Ridgway, IL", "Ridott, IL", "Rinard, IL",
"Ringwood, IL", "Rio, IL", "River Forest, IL", "River Grove, IL",
"Riverdale, IL", "Roanoke, IL", "Robbins, IL", "Robbs, IL",
"Roberts, IL", "Robinson, IL", "Rochelle, IL", "Rock City, IL",
"Rock Falls, IL", "Rock Island, IL", "Rockbridge, IL", "Rockdale, IL",
"Rockford, IL", "Rockport, IL", "Rockton, IL", "Rockwood, IL",
"Rolling Meadows, IL", "Romeoville, IL", "Roodhouse, IL",
"Rosamond, IL", "Roscoe, IL", "Roselle, IL", "Rosemont, IL",
"Roseville, IL", "Rosiclar, IL", "Rossville, IL", "Round Lake, IL",
"Roxana, IL", "Royal Lakes, IL", "Royalton, IL", "Rutland, IL",
"Sadorus, IL", "Saint Anne, IL", "Saint Augustine, IL",
"Saint Charles, IL", "Saint David, IL", "Saint Elmo, IL",
"Saint Francisvil, IL", "Saint Jacob, IL", "Saint Joseph, IL",
"Saint Mary, IL", "Saint Peter, IL", "Salem, IL", "Sandoval, IL",
"Sandwich, IL", "Sauget, IL", "Sauk Village, IL", "Saunemin, IL",
"Savanna, IL", "Savoy, IL", "Saybrook, IL", "Scales Mound, IL",
"Schaumburg, IL", "Scheller, IL", "Schiller Park, IL", "Sciota, IL",
"Scott A F B, IL", "Scottville, IL", "Seaton, IL", "Secor, IL",
"Seneca, IL", "Serena, IL", "Sesser, IL", "Seymour, IL", "Shabbona, IL",
"Shannon, IL", "Shattuc, IL", "Shawneetown, IL", "Sheffield, IL",
"Sheldon, IL", "Sheridan, IL", "Sherrard, IL", "Shiloh, IL",

"Shirley, IL", "Shobonier, IL", "Shorewood, IL", "Shumway, IL",
 "Sibley, IL", "Sicily, IL", "Sidell, IL", "Sidney, IL", "Sigel, IL",
 "Silvis, IL", "Sims, IL", "Skokie, IL", "Smithboro, IL",
 "Smithfield, IL", "Smithshire, IL", "Smithton, IL", "Somonauk, IL",
 "Sorento, IL", "South Beloit, IL", "South Elgin, IL",
 "South Holland, IL", "Southern View, IL", "Sparland, IL", "Sparta, IL",
 "Spaulding, IL", "Speer, IL", "Spring Grove, IL", "Spring Valley, IL",
 "Springerton, IL", "Springfield, IL", "Stanford, IL", "Staunton, IL",
 "Steeleville, IL", "Steger, IL", "Sterling, IL", "Steward, IL",
 "Stewardson, IL", "Stickney, IL", "Stillman Valley, IL", "Stockton, IL",
 "Stone Park, IL", "Stonefort, IL", "Stonington, IL", "Strasburg, IL",
 "Strawn, IL", "Streamwood, IL", "Streator, IL", "Stronghurst, IL",
 "Sublette, IL", "Sugar Grove, IL", "Sullivan, IL", "Sumner, IL",
 "Sunnyland, IL", "Sutter, IL", "Swanwick, IL", "Sycamore, IL",
 "Table Grove, IL", "Tallula, IL", "Tamaroa, IL", "Tamms, IL",
 "Tampico, IL", "Taylor Ridge, IL", "Techny, IL", "Tennessee, IL",
 "Teutopolis, IL", "Texico, IL", "Thawville, IL", "Thomasboro, IL",
 "Thomasville, IL", "Thompsonville, IL", "Thomson, IL", "Thornton, IL",
 "Tilton, IL", "Timewell, IL", "Tinley Park, IL", "Tiskilwa, IL",
 "Toledo, IL", "Tolono, IL", "Toluca, IL", "Tonica, IL", "Topeka, IL",
 "Toulon, IL", "Towanda, IL", "Tremont, IL", "Trenton, IL", "Trilla, IL",
 "Trivoli, IL", "Troy, IL", "Tunnel Hill, IL", "Tuscola, IL",
 "Ullin, IL", "Union, IL", "University Park, IL", "Urbana, IL",
 "Ursa, IL", "Utica, IL", "Valmeyer, IL", "Vandalia, IL", "Varna, IL",
 "Venedy, IL", "Venice, IL", "Vergennes, IL", "Vermont, IL",
 "Vernon, IL", "Vernon Hills, IL", "Verona, IL", "Versailles, IL",
 "Victoria, IL", "Vienna, IL", "Villa Grove, IL", "Villa Park, IL",
 "Villa Ridge, IL", "Viola, IL", "Virden, IL", "Wadsworth, IL",
 "Waggoner, IL", "Walnut Hill, IL", "Walsh, IL", "Walshville, IL",
 "Waltonville, IL", "Wapella, IL", "Warren, IL", "Warrenville, IL",
 "Warsaw, IL", "Washburn, IL", "Washington Park, IL", "Wataga, IL",
 "Waterloo, IL", "Waterman, IL", "Watseka, IL", "Watson, IL",
 "Wauconda, IL", "Waukegan, IL", "Waverly, IL", "Wayne City, IL",
 "Waynesville, IL", "Weldon, IL", "Wellington, IL", "Wenona, IL",
 "West Brooklyn, IL", "West Chicago, IL", "West Frankfort, IL",
 "West Liberty, IL", "West Point, IL", "West Salem, IL",
 "West Union, IL", "West York, IL", "Westchester, IL",
 "Western Springs, IL", "Westfield, IL", "Westmont, IL", "Westville, IL",
 "Wheaton, IL", "Wheeler, IL", "Wheeling, IL", "White Hall, IL",
 "White Heath, IL", "Whittington, IL", "Williamsfield, IL",
 "Williamsville, IL", "Willisville, IL", "Willow Hill, IL",
 "Willow Springs, IL", "Wilmette, IL", "Windsor, IL", "Winfield, IL",
 "Winnebago, IL", "Winslow, IL", "Winthrop Harbor, IL", "Witt, IL",
 "Wolf Lake, IL", "Wonder Lake, IL", "Wood Dale, IL", "Wood River, IL",
 "Woodhull, IL", "Woodlawn, IL", "Woodridge, IL", "Woodstock, IL",
 "Worden, IL", "Worth, IL", "Wyandot, IL", "Wyoming, IL", "Xenia, IL",
 "Yale, IL", "Yates City, IL", "Yorkville, IL", "Zeigler, IL",
 "Zion, IL", "Acton, IN", "Adams, IN", "Akron, IN", "Albany, IN",
 "Albion, IN", "Alexandria, IN", "Ambia, IN", "Amboy, IN",
 "Anderson, IN", "Andrews, IN", "Angola, IN", "Arcadia, IN", "Argos, IN",
 "Arlington, IN", "Ashley, IN", "Atlanta, IN", "Attica, IN",
 "Auburn, IN", "Aurora, IN", "Austin, IN", "Avilla, IN", "Avon, IN",
 "Bainbridge, IN", "Ball State Unive, IN", "Bargersville, IN",
 "Batesville, IN", "Bath, IN", "Battle Ground, IN", "Bedford, IN",
 "Beech Grove, IN", "Bennington, IN", "Bicknell, IN", "Birdseye, IN",

"Bloomfield, IN", "Bloomington, IN", "Bluffton, IN",
"Boggstown, IN", "Boonville, IN", "Borden, IN", "Boswell, IN",
"Bourbon, IN", "Bowling Green, IN", "Branchville, IN", "Brazil, IN",
"Bremen, IN", "Bridgeport, IN", "Bridgeton, IN", "Bringinghurst, IN",
"Bristol, IN", "Brook, IN", "Brookston, IN", "Brookville, IN",
"Brownsburg, IN", "Brownstown, IN", "Brownsville, IN", "Bruceville, IN",
"Bryant, IN", "Buckskin, IN", "Buffaloville, IN", "Bunker Hill, IN",
"Burnettsville, IN", "Butler, IN", "Butlerville, IN",
"Cambridge City, IN", "Camby, IN", "Camden, IN", "Campbellsburg, IN",
"Canaan, IN", "Cannelburg, IN", "Carbon, IN", "Carlisle, IN",
"Carmel, IN", "Carthage, IN", "Castleton, IN", "Cates, IN",
"Cayuga, IN", "Cedar Grove, IN", "Celestine, IN", "Centerpoint, IN",
"Centerton, IN", "Centerville, IN", "Central, IN", "Chalmers, IN",
"Chandler, IN", "Charlestown, IN", "Charlottesville, IN",
"Chesterfield, IN", "Chili, IN", "Chrisney, IN", "Churubusco, IN",
"Cicero, IN", "Clarks Hill, IN", "Clarksville, IN", "Clay City, IN",
"Claypool, IN", "Clayton, IN", "Clermont, IN", "Clinton, IN",
"Cloverdale, IN", "Coal City, IN", "Coatesville, IN", "Colfax, IN",
"Collegeville, IN", "Columbia City, IN", "Columbus, IN",
"Commiskey, IN", "Connersville, IN", "Converse, IN", "Cortland, IN",
"Corunna, IN", "Cory, IN", "Corydon, IN", "Covington, IN",
"Craigville, IN", "Crandall, IN", "Crane Naval Depo, IN",
"Crawfordsville, IN", "Cromwell, IN", "Cross Plains, IN",
"Crothersville, IN", "Crown Point, IN", "Culver Military, IN",
"Cumberland, IN", "Cutler, IN", "Cynthiana, IN", "Dale, IN",
"Daleville, IN", "Dana, IN", "Danville, IN", "Darlington, IN",
"Decatur, IN", "Decker, IN", "Delphi, IN", "Demotte, IN", "Depauw, IN",
"Deputy, IN", "Derby, IN", "Dillsboro, IN", "Dubois, IN", "Dugger, IN",
"Dunkirk, IN", "Dupont, IN", "Dyer, IN", "Eagle Creek, IN",
"Earl Park, IN", "East Cedar Lake, IN", "East Chicago, IN", "Eaton, IN",
"Eckerty, IN", "Economy, IN", "Edinburgh, IN", "Edwardsport, IN",
"Elberfeld, IN", "Elizabeth, IN", "Elizabethtown, IN", "Elkhart, IN",
"Ellettsville, IN", "Elnora, IN", "Elwood, IN", "English, IN",
"Etna Green, IN", "Evanston, IN", "Evansville, IN", "Fair Oaks, IN",
"Fairbanks, IN", "Fairland, IN", "Fairmount, IN", "Falmouth, IN",
"Farmersburg, IN", "Farmland, IN", "Ferdinand, IN", "Fillmore, IN",
"Fishers, IN", "Flat Rock, IN", "Flora, IN", "Florence, IN",
"Floyds Knobs, IN", "Foraker, IN", "Forest, IN", "Fort Benjamin Ha, IN",
"Fort Branch, IN", "Fort Wayne, IN", "Fortville, IN",
"Fountain City, IN", "Fountaintown, IN", "Fowler, IN",
"Francesville, IN", "Francisco, IN", "Franklin, IN", "Frankton, IN",
"Fredericksburg, IN", "Freedom, IN", "Freetown, IN", "Fremont, IN",
"French Lick, IN", "Friendship, IN", "Galveston, IN", "Garrett, IN",
"Gary, IN", "Gas City, IN", "Gaston, IN", "Geneva, IN",
"Gentryville, IN", "Georgetown, IN", "Glenwood, IN", "Goodland, IN",
"Gosport, IN", "Grabill, IN", "Grammer, IN", "Grandview, IN",
"Granger, IN", "Grantsburg, IN", "Greencastle, IN", "Greenfield, IN",
"Greens Fork, IN", "Greentown, IN", "Greenville, IN", "Greenwood, IN",
"Griffin, IN", "Griffith, IN", "Grissom Air Forc, IN", "Grovertown, IN",
"Guilford, IN", "Hagerstown, IN", "Hamilton, IN", "Hamlet, IN",
"Hammond, IN", "Hanna, IN", "Hanover, IN", "Hardinsburg, IN",
"Harlan, IN", "Hartford City, IN", "Hartsville, IN", "Haubstadt, IN",
"Haysville, IN", "Hazleton, IN", "Hebron, IN", "Helmer, IN",
"Heltonville, IN", "Henryville, IN", "Highland, IN", "Hillisburg, IN",
"Hillsboro, IN", "Hillsdale, IN", "Hoagland, IN", "Hobart, IN",

"Holland, IN", "Holton, IN", "Hope, IN", "Howe, IN", "Huntertown, IN",
"Huntingburg, IN", "Huntington, IN", "Idaville, IN", "Indianapolis, IN",
"Ingalls, IN", "Inwood, IN", "Jamestown, IN", "Jasonville, IN",
"Jeffersonville, IN", "Jonesboro, IN", "Kempton, IN",
"Kendallville, IN", "Kentland, IN", "Kewanna, IN", "Keystone, IN",
"Kimmell, IN", "Kirklin, IN", "Knightstown, IN", "Kokomo, IN",
"Kouts, IN", "Kyana, IN", "La Crosse, IN", "La Fontaine, IN",
"La Porte, IN", "Laconia, IN", "Ladoga, IN", "Lafayette, IN",
"Lagrange, IN", "Lagro, IN", "Lake Station, IN", "Lake Village, IN",
"Lakeville, IN", "Landess, IN", "Lanesville, IN", "Laotto, IN",
"Lapel, IN", "Larwill, IN", "Laurel, IN", "Lawrence, IN",
"Lawrenceburg, IN", "Leavenworth, IN", "Lebanon, IN", "Leesburg, IN",
"Leo, IN", "Leopold, IN", "Lewis, IN", "Lewisville, IN",
"Lexington, IN", "Liberty, IN", "Liberty Center, IN", "Ligonier, IN",
"Lincoln City, IN", "Linden, IN", "Linn Grove, IN", "Linton, IN",
"Lizton, IN", "Logansport, IN", "Loogootee, IN", "Losantville, IN",
"Lowell, IN", "Lucerne, IN", "Lynn, IN", "Lynnville, IN", "Lyons, IN",
"Macy, IN", "Madison, IN", "Magnet, IN", "Manilla, IN", "Marengo, IN",
"Mariah Hill, IN", "Marion, IN", "Markle, IN", "Markleville, IN",
"Marshall, IN", "Marshfield, IN", "Marysville, IN", "Mauckport, IN",
"Mc Cordsville, IN", "Medaryville, IN", "Medora, IN", "Memphis, IN",
"Mentone, IN", "Merom, IN", "Merrillville, IN", "Metamora, IN",
"Michigan City, IN", "Michigantown, IN", "Middlebury, IN",
"Middletown, IN", "Milan, IN", "Milford, IN", "Mill Creek, IN",
"Millersburg, IN", "Milltown, IN", "Milroy, IN", "Milton, IN",
"Mishawaka, IN", "Mitchell, IN", "Modoc, IN", "Monon, IN", "Monroe, IN",
"Monroe City, IN", "Monroeville, IN", "Monrovia, IN", "Monterey, IN",
"Montezuma, IN", "Montgomery, IN", "Monticello, IN", "Montpelier, IN",
"Mooreland, IN", "Moores Hill, IN", "Mooresville, IN", "Morgantown, IN",
"Morocco, IN", "Morristown, IN", "Mount Pleasant, IN",
"Mount Vernon, IN", "Mulberry, IN", "Muncie, IN", "Munster, IN",
"Nabb, IN", "Nappanee, IN", "Nashville, IN", "Needham, IN",
"New Albany, IN", "New Augusta, IN", "New Carlisle, IN",
"New Castle, IN", "New Harmony, IN", "New Haven, IN",
"New Middletown, IN", "New Palestine, IN", "New Paris, IN",
"New Richmond, IN", "New Ross, IN", "New Salisbury, IN",
"New Washington, IN", "New Whiteland, IN", "Newberry, IN",
"Newburgh, IN", "Nineveh, IN", "Noblesville, IN", "Nora, IN",
"Norman, IN", "North Judson, IN", "North Liberty, IN",
"North Manchester, IN", "North Salem, IN", "North Terre Haut, IN",
"North Vernon, IN", "North Webster, IN", "Oakland City, IN",
"Oaklondon, IN", "Oaktown, IN", "Ober, IN", "Odon, IN", "Oldenburg, IN",
"Oolitic, IN", "Orland, IN", "Orleans, IN", "Osceola, IN", "Osgood, IN",
"Ossian, IN", "Otisco, IN", "Otterbein, IN", "Otwell, IN",
"Owensburg, IN", "Owensville, IN", "Oxford, IN", "Palmyra, IN",
"Paoli, IN", "Paragon, IN", "Paris Crossing, IN", "Park Fletcher, IN",
"Parker City, IN", "Patoka, IN", "Patriot, IN", "Pekin, IN",
"Pendleton, IN", "Pennville, IN", "Perrysville, IN", "Peru, IN",
"Petersburg, IN", "Pierceton, IN", "Pimento, IN", "Pine Village, IN",
"Pittsboro, IN", "Plainville, IN", "Pleasant Lake, IN", "Poland, IN",
"Poneto, IN", "Portage, IN", "Porter, IN", "Portland, IN",
"Poseyville, IN", "Princeton, IN", "Quincy, IN", "Ramsey, IN",
"Redkey, IN", "Reelsville, IN", "Remington, IN", "Reynolds, IN",
"Richland, IN", "Richmond, IN", "Ridgeville, IN", "Rising Sun, IN",
"Roachdale, IN", "Roann, IN", "Roanoke, IN", "Rochester, IN",

"Rockport, IN", "Rockville, IN", "Rolling Prairie, IN", "Rome, IN",
 "Rome City, IN", "Romney, IN", "Rosedale, IN", "Rossville, IN",
 "Royal Center, IN", "Rushville, IN", "Russellville, IN",
 "Russiaville, IN", "Saint Croix, IN", "Saint Joe, IN", "Saint John, IN",
 "Saint Marys, IN", "Saint Meinrad, IN", "Saint Paul, IN",
 "Salamonia, IN", "Salem, IN", "San Pierre, IN", "Sandborn, IN",
 "Sandford, IN", "Santa Claus, IN", "Saratoga, IN", "Schererville, IN",
 "Schnellville, IN", "Scipio, IN", "Scottsburg, IN", "Selma, IN",
 "Seymour, IN", "Sharpsville, IN", "Shelburn, IN", "Shelbyville, IN",
 "Sheridan, IN", "Shipshewana, IN", "Shirley, IN", "Shoals, IN",
 "Siberia, IN", "Silver Lake, IN", "Solsberry, IN", "South Bend, IN",
 "South Whitley, IN", "Southport, IN", "Speed, IN", "Speedway, IN",
 "Spencer, IN", "Spencerville, IN", "Spiceland, IN", "Springport, IN",
 "Springville, IN", "Star City, IN", "Stendal, IN", "Stilesville, IN",
 "Straughn, IN", "Sullivan, IN", "Sulphur, IN", "Sulphur Springs, IN",
 "Summitville, IN", "Sunman, IN", "Swayzee, IN", "Switz City, IN",
 "Syracuse, IN", "Tangier, IN", "Taswell, IN", "Tell City, IN",
 "Tennyson, IN", "Terre Haute, IN", "Thorntown, IN", "Tippecanoe, IN",
 "Tipton, IN", "Tobinsport, IN", "Topeka, IN", "Trafalgar, IN",
 "Troy, IN", "Twelve Mile, IN", "Underwood, IN", "Union City, IN",
 "Union Mills, IN", "Uniondale, IN", "Unionville, IN", "Upland, IN",
 "Urbana, IN", "Vallonia, IN", "Valparaiso, IN", "Veedersburg, IN",
 "Velpen, IN", "Vernon, IN", "Versailles, IN", "Vevay, IN",
 "Vincennes, IN", "W Harrison, IN", "Wabash, IN", "Wadesville, IN",
 "Wakarusa, IN", "Waldron, IN", "Walkerton, IN", "Walton, IN",
 "Wanamaker, IN", "Wanatah, IN", "Warren, IN", "Warsaw, IN",
 "Washington, IN", "Waterloo, IN", "Waveland, IN", "Wawaka, IN",
 "Waynetown, IN", "Webster, IN", "West Baden Sprin, IN",
 "West Lafayette, IN", "West Lebanon, IN", "Westfield, IN",
 "Westpoint, IN", "Westport, IN", "Westville, IN", "Wheatfield, IN",
 "Wheatland, IN", "Whitestown, IN", "Whiting, IN", "Wilkinson, IN",
 "Williams, IN", "Williamsburg, IN", "Winamac, IN", "Winchester, IN",
 "Windfall, IN", "Wingate, IN", "Winona Lake, IN", "Winslow, IN",
 "Wolcott, IN", "Wolcottville, IN", "Woodbridge, IN", "Woodburn, IN",
 "Worthington, IN", "Yoder, IN", "Yorktown, IN", "Zionsville, IN",
 "Abbyville, KS", "Abilene, KS", "Ada, KS", "Admire, KS", "Agenda, KS",
 "Agra, KS", "Alamota, KS", "Albert, KS", "Alden, KS", "Alexander, KS",
 "Alma, KS", "Almena, KS", "Alta Vista, KS", "Altamont, KS", "Alton, KS",
 "Altoona, KS", "Americus, KS", "Ames, KS", "Andale, KS", "Andover, KS",
 "Antelope, KS", "Anthony, KS", "Antonino, KS", "Arcadia, KS",
 "Argonia, KS", "Arkansas City, KS", "Arlington, KS", "Arnold, KS",
 "Ashland, KS", "Assaria, KS", "Atchison, KS", "Athol, KS",
 "Atlanta, KS", "Attica, KS", "Atwood, KS", "Auburn, KS", "Augusta, KS",
 "Aurora, KS", "Axtell, KS", "Baileyville, KS", "Baldwin City, KS",
 "Barnard, KS", "Barnes, KS", "Bartlett, KS", "Basehor, KS",
 "Bavaria, KS", "Baxter Springs, KS", "Bazine, KS", "Beattie, KS",
 "Beaumont, KS", "Beaver, KS", "Beeler, KS", "Bel Aire, KS",
 "Bellaire, KS", "Belle Plaine, KS", "Belleville, KS", "Belmont, KS",
 "Belpre, KS", "Belvue, KS", "Bendena, KS", "Benedict, KS",
 "Bennington, KS", "Bentley, KS", "Benton, KS", "Bern, KS",
 "Berryton, KS", "Beverly, KS", "Bird City, KS", "Bison, KS",
 "Blaine, KS", "Bloom, KS", "Blue Mound, KS", "Blue Rapids, KS",
 "Bluff City, KS", "Bogue, KS", "Bremen, KS", "Brewster, KS",
 "Bronson, KS", "Brookville, KS", "Brownell, KS", "Bucklin, KS",
 "Bucyrus, KS", "Buffalo, KS", "Buhler, KS", "Bunker Hill, KS",

"Burden, KS", "Burdett, KS", "Burdick, KS", "Burlingame, KS",
 "Burns, KS", "Burr Oak, KS", "Burrton, KS", "Bushong, KS",
 "Bushton, KS", "Byers, KS", "Caldwell, KS", "Cambridge, KS",
 "Caney, KS", "Canton, KS", "Carbondale, KS", "Carlton, KS",
 "Carlyle, KS", "Carona, KS", "Cassoday, KS", "Catharine, KS",
 "Cawker City, KS", "Cedar, KS", "Cedar Vale, KS", "Centerville, KS",
 "Centralia, KS", "Chanute, KS", "Chapman, KS", "Chase, KS",
 "Cheney, KS", "Cherokee, KS", "Cherryvale, KS", "Chetopa, KS",
 "Cimarron, KS", "Circleville, KS", "Claflin, KS", "Clay Center, KS",
 "Clayton, KS", "Clearwater, KS", "Clements, KS", "Clifton, KS",
 "Climax, KS", "Clyde, KS", "Coats, KS", "Codell, KS", "Coffeyville, KS",
 "Colby, KS", "Coldwater, KS", "Collyer, KS", "Colony, KS",
 "Colwich, KS", "Conway, KS", "Conway Springs, KS", "Copeland, KS",
 "Corbin, KS", "Corning, KS", "Cottonwood Falls, KS", "Countryside, KS",
 "Courtland, KS", "Coyville, KS", "Crestline, KS", "Cuba, KS",
 "Culver, KS", "Cummings, KS", "Damar, KS", "Danville, KS",
 "De Soto, KS", "Deerfield, KS", "Delavan, KS", "Delia, KS",
 "Delphos, KS", "Denison, KS", "Dennis, KS", "Densmore, KS",
 "Denton, KS", "Derby, KS", "Dexter, KS", "Dodge City, KS",
 "Dorrance, KS", "Douglass, KS", "Dover, KS", "Downs, KS", "Dresden, KS",
 "Duluth, KS", "Dunlap, KS", "Durham, KS", "Dwight, KS",
 "Eastborough, KS", "Easton, KS", "Edgerton, KS", "Edmond, KS",
 "Edna, KS", "Edson, KS", "Effingham, KS", "El Dorado, KS", "Elbing, KS",
 "Elk City, KS", "Elk Falls, KS", "Elkhart, KS", "Ellinwood, KS",
 "Ellis, KS", "Ellsworth, KS", "Elmdale, KS", "Elsmore, KS",
 "Emmett, KS", "Emporia, KS", "Englewood, KS", "Ensign, KS",
 "Enterprise, KS", "Erie, KS", "Esbon, KS", "Eskridge, KS", "Eudora, KS",
 "Eureka, KS", "Everest, KS", "Fairview, KS", "Fall River, KS",
 "Falun, KS", "Farlington, KS", "Florence, KS", "Fontana, KS",
 "Ford, KS", "Formoso, KS", "Fort Dodge, KS", "Fort Leavenworth, KS",
 "Fort Riley, KS", "Fowler, KS", "Freeport, KS", "Friend, KS",
 "Fulton, KS", "Galatia, KS", "Galena, KS", "Galesburg, KS", "Galva, KS",
 "Garden City, KS", "Garden Plain, KS", "Gardner, KS", "Garfield, KS",
 "Garland, KS", "Garnett, KS", "Gaylord, KS", "Gem, KS", "Geneseo, KS",
 "Geuda Springs, KS", "Girard, KS", "Glade, KS", "Glasco, KS",
 "Glen Elder, KS", "Goddard, KS", "Goessel, KS", "Goff, KS",
 "Goodland, KS", "Gorham, KS", "Gove, KS", "Grainfield, KS",
 "Grantville, KS", "Greeley, KS", "Green, KS", "Greenleaf, KS",
 "Greensburg, KS", "Grenola, KS", "Gridley, KS", "Grinnell, KS",
 "Gypsum, KS", "Haddam, KS", "Hallowell, KS", "Halstead, KS",
 "Hamilton, KS", "Hanover, KS", "Hanston, KS", "Hardtner, KS",
 "Harlan, KS", "Harper, KS", "Hartford, KS", "Harveyville, KS",
 "Havana, KS", "Haven, KS", "Havensville, KS", "Haviland, KS",
 "Haysville, KS", "Hazelton, KS", "Healy, KS", "Heizer, KS",
 "Hepler, KS", "Herkimer, KS", "Herndon, KS", "Hesston, KS",
 "Hiattville, KS", "Highland, KS", "Hill City, KS", "Hillsboro, KS",
 "Hillsdale, KS", "Holcomb, KS", "Hollenberg, KS", "Holton, KS",
 "Holyrood, KS", "Home, KS", "Hope, KS", "Horton, KS", "Howard, KS",
 "Hoxie, KS", "Hoyt, KS", "Hudson, KS", "Hugoton, KS", "Humboldt, KS",
 "Hunter, KS", "Huron, KS", "Hutchinson, KS", "Independence, KS",
 "Industrial Airpo, KS", "Ingalls, KS", "Inman, KS", "Ionia, KS",
 "Isabel, KS", "Iuka, KS", "Jamestown, KS", "Jennings, KS",
 "Jetmore, KS", "Johnson, KS", "Junction City, KS", "Kalvesta, KS",
 "Kanopolis, KS", "Kanorado, KS", "Kansas City, KS", "Kelly, KS",
 "Kendall, KS", "Kensington, KS", "Kingsdown, KS", "Kinsley, KS",

"Kiowa, KS", "Kirwin, KS", "Kismet, KS", "La Crosse, KS",
 "La Cygne, KS", "La Harpe, KS", "Lafontaine, KS", "Lake City, KS",
 "Lake Of The Fore, KS", "Lake Quivira, KS", "Lakin, KS", "Lane, KS",
 "Langdon, KS", "Lansing, KS", "Latham, KS", "Lawrence, KS",
 "Lawton, KS", "Le Roy, KS", "Leavenworth, KS", "Leawood, KS",
 "Lebo, KS", "Lecompton, KS", "Lehigh, KS", "Lenexa, KS", "Leon, KS",
 "Leona, KS", "Leonardville, KS", "Leoti, KS", "Levant, KS", "Lewis, KS",
 "Liberal, KS", "Liberty, KS", "Liebenthal, KS", "Lindsborg, KS",
 "Linn, KS", "Linwood, KS", "Little River, KS", "Logan, KS",
 "Long Island, KS", "Longford, KS", "Longton, KS", "Lorraine, KS",
 "Lost Springs, KS", "Louisburg, KS", "Louisville, KS", "Lucas, KS",
 "Ludell, KS", "Luray, KS", "Lyndon, KS", "Lyons, KS", "Macksville, KS",
 "Madison, KS", "Mahaska, KS", "Maize, KS", "Manchester, KS",
 "Manhattan, KS", "Mankato, KS", "Manter, KS", "Maple City, KS",
 "Maple Hill, KS", "Mapleton, KS", "Marion, KS", "Marquette, KS",
 "Marysville, KS", "Matfield Green, KS", "Mayetta, KS", "Mayfield, KS",
 "Mc Connell A F B, KS", "Mc Cracken, KS", "Mc Cune, KS",
 "Mc Donald, KS", "Mc Louth, KS", "Meade, KS", "Medicine Lodge, KS",
 "Medora, KS", "Melvorn, KS", "Menlo, KS", "Mentor, KS", "Meriden, KS",
 "Milan, KS", "Mildred, KS", "Milford, KS", "Milton, KS",
 "Miltonvale, KS", "Minneapolis, KS", "Mission, KS", "Modoc, KS",
 "Moline, KS", "Montezuma, KS", "Monument, KS", "Moran, KS",
 "Morganville, KS", "Morland, KS", "Morrill, KS", "Morrowville, KS",
 "Moscow, KS", "Mound City, KS", "Mound Valley, KS", "Moundridge, KS",
 "Mount Hope, KS", "Mulberry, KS", "Mullinville, KS", "Mulvane, KS",
 "Munden, KS", "Murdock, KS", "Muscotah, KS", "Narka, KS",
 "Nashville, KS", "Natoma, KS", "Navarre, KS", "Nekoma, KS",
 "Neodesha, KS", "Neosho Falls, KS", "Neosho Rapids, KS",
 "Ness City, KS", "Netawaka, KS", "New Albany, KS", "New Almelo, KS",
 "New Cambria, KS", "Newton, KS", "Nickerson, KS", "Niotaze, KS",
 "Norcatur, KS", "North Newton, KS", "Norton, KS", "Nortonville, KS",
 "Norway, KS", "Norwich, KS", "Oakhill, KS", "Oakley, KS", "Oberlin, KS",
 "Odin, KS", "Offerle, KS", "Ogallah, KS", "Ogden, KS", "Oketo, KS",
 "Olathe, KS", "Olpe, KS", "Olsburg, KS", "Onaga, KS", "Oneida, KS",
 "Osage City, KS", "Osawatomie, KS", "Osborne, KS", "Oskaloosa, KS",
 "Oswego, KS", "Ottawa, KS", "Overbrook, KS", "Overland Park, KS",
 "Oxford, KS", "Ozawkie, KS", "Palco, KS", "Palmer, KS", "Paola, KS",
 "Paradise, KS", "Park, KS", "Park City, KS", "Parker, KS",
 "Parsons, KS", "Partridge, KS", "Pauline, KS", "Pawnee Rock, KS",
 "Paxico, KS", "Peabody, KS", "Peck, KS", "Penalosa, KS", "Penokee, KS",
 "Perry, KS", "Peru, KS", "Pfeifer, KS", "Phillipsburg, KS",
 "Piedmont, KS", "Pierceville, KS", "Piqua, KS", "Plains, KS",
 "Plainville, KS", "Pleasanton, KS", "Plevna, KS", "Pomona, KS",
 "Portis, KS", "Potwin, KS", "Powhattan, KS", "Prairie View, KS",
 "Prairie Village, KS", "Pratt, KS", "Prescott, KS",
 "Pretty Prairie, KS", "Princeton, KS", "Protection, KS", "Quenemo, KS",
 "Quinter, KS", "Radium, KS", "Radley, KS", "Rago, KS", "Ramona, KS",
 "Randall, KS", "Randolph, KS", "Ransom, KS", "Rantoul, KS",
 "Raymond, KS", "Reading, KS", "Redfield, KS", "Republic, KS",
 "Reserve, KS", "Rice, KS", "Richfield, KS", "Richmond, KS", "Riley, KS",
 "Riverton, KS", "Rock, KS", "Rolla, KS", "Rosalia, KS", "Rose Hill, KS",
 "Rosedale, KS", "Rossville, KS", "Roxbury, KS", "Rozel, KS",
 "Rush Center, KS", "Russell, KS", "Russell Springs, KS", "Sabetha, KS",
 "Saint George, KS", "Saint John, KS", "Saint Marys, KS",
 "Saint Paul, KS", "Satanta, KS", "Savonburg, KS", "Sawyer, KS",

"Scandia, KS", "Schoenchen, KS", "Scottsville, KS", "Scranton, KS",
 "Sedan, KS", "Sedgwick, KS", "Selden, KS", "Severance, KS",
 "Seward, KS", "Sharon, KS", "Sharon Springs, KS", "Shawnee, KS",
 "Shawnee Mission, KS", "Shields, KS", "Silver Lake, KS", "Simpson, KS",
 "Smith Center, KS", "Smolan, KS", "Soldier, KS", "Solomon, KS",
 "South Haven, KS", "South Hutchinson, KS", "Spearville, KS",
 "Spivey, KS", "Spring Hill, KS", "Stafford, KS", "Stanley, KS",
 "Stark, KS", "Sterling, KS", "Stilwell, KS", "Stockton, KS",
 "Strawn, KS", "Strong City, KS", "Studley, KS", "Sublette, KS",
 "Summerfield, KS", "Sun City, KS", "Susank, KS", "Sylvan Grove, KS",
 "Sylvia, KS", "Syracuse, KS", "Talmage, KS", "Tampa, KS",
 "Tecumseh, KS", "Thayer, KS", "Timken, KS", "Tipton, KS",
 "Tonganoxie, KS", "Topeka, KS", "Toronto, KS", "Towanda, KS",
 "Treece, KS", "Tribune, KS", "Udall, KS", "Ulysses, KS",
 "Uniontown, KS", "Utica, KS", "Valley Center, KS", "Valley Falls, KS",
 "Vassar, KS", "Victoria, KS", "Viola, KS", "Virgil, KS", "Vliets, KS",
 "Wa Keeney, KS", "Wakarusa, KS", "Wakefield, KS", "Waldo, KS",
 "Waldron, KS", "Wallace, KS", "Walnut, KS", "Walton, KS", "Wamego, KS",
 "Washington, KS", "Waterville, KS", "Wathena, KS", "Waverly, KS",
 "Webber, KS", "Welda, KS", "Wellington, KS", "Wells, KS",
 "Wellsville, KS", "Weskan, KS", "Westfall, KS", "Westphalia, KS",
 "Wetmore, KS", "Wheeler, KS", "White City, KS", "White Cloud, KS",
 "Whitewater, KS", "Whiting, KS", "Wichita, KS", "Williamsburg, KS",
 "Wilmore, KS", "Wilsey, KS", "Wilson, KS", "Winchester, KS",
 "Windom, KS", "Winfield, KS", "Winifred, KS", "Winona, KS",
 "Woodbine, KS", "Woodston, KS", "Wright, KS", "Yates Center, KS",
 "Zenda, KS", "Zurich, KS", "Aaron, KY", "Adairville, KY", "Adams, KY",
 "Adolphus, KY", "Aflex, KY", "Ages Brookside, KY", "Albany, KY",
 "Alcalde, KY", "Alexandria, KY", "Allen, KY", "Allensville, KY",
 "Almo, KY", "Alpha, KY", "Altro, KY", "Alvaton, KY", "Amburgey, KY",
 "Anchorage, KY", "Anco, KY", "Argillite, KY", "Argo, KY", "Arjay, KY",
 "Arlington, KY", "Artemus, KY", "Ary, KY", "Ashcamp, KY", "Ashland, KY",
 "Auburn, KY", "Augusta, KY", "Austin, KY", "Auxier, KY", "Bagdad, KY",
 "Bailey Switch, KY", "Bakerton, KY", "Banner, KY", "Bardstown, KY",
 "Bardwell, KY", "Barlow, KY", "Barnetts Creek, KY", "Battletown, KY",
 "Baxter, KY", "Bear Branch, KY", "Bearville, KY", "Beaumont, KY",
 "Beaver Dam, KY", "Bedford, KY", "Bee Spring, KY", "Beech Creek, KY",
 "Beechmont, KY", "Belcher, KY", "Belfry, KY", "Bellevue, KY",
 "Belton, KY", "Benham, KY", "Benton, KY", "Berea, KY", "Berry, KY",
 "Bethanna, KY", "Bethelridge, KY", "Bethlehem, KY", "Betsy Layne, KY",
 "Beverly, KY", "Bevinsville, KY", "Big Clifty, KY", "Big Creek, KY",
 "Big Laurel, KY", "Big Rock, KY", "Big Spring, KY", "Biggs, KY",
 "Bimble, KY", "Blaine, KY", "Blairs Mill, KY", "Bloomfield, KY",
 "Blue Diamond, KY", "Blue River, KY", "Boaz, KY", "Bondville, KY",
 "Bonnieville, KY", "Boons Camp, KY", "Boston, KY", "Bow, KY",
 "Bowling Green, KY", "Bradfordsville, KY", "Brandenburg, KY",
 "Breeding, KY", "Bremen, KY", "Bright Shade, KY", "Brinkley, KY",
 "Broad Bottom, KY", "Brodhead, KY", "Bronston, KY", "Brooks, KY",
 "Brooksville, KY", "Browder, KY", "Browning, KY", "Bryants Store, KY",
 "Buckhorn, KY", "Buckingham, KY", "Buckner, KY", "Buechel, KY",
 "Buffalo, KY", "Burke, KY", "Burkesville, KY", "Burkhart, KY",
 "Burna, KY", "Bush, KY", "Busy, KY", "Butler, KY", "Bybee, KY",
 "Calhoun, KY", "California, KY", "Callaway, KY", "Calvert City, KY",
 "Calvin, KY", "Camp Dix, KY", "Campbellsburg, KY", "Campbellsville, KY",
 "Canada, KY", "Caney, KY", "Caneyville, KY", "Canmer, KY", "Cannon, KY",

"Canoe, KY", "Carcassonne, KY", "Carlisle, KY", "Carrie, KY",
"Carrollton, KY", "Carrsville, KY", "Carver, KY", "Casey Creek, KY",
"Catlettsburg, KY", "Cave City, KY", "Center, KY", "Centertown, KY",
"Central City, KY", "Cerulean, KY", "Chaplin, KY", "Chavies, KY",
"Chevrolet, KY", "Cinda, KY", "Clay, KY", "Clayhole, KY",
"Clearfield, KY", "Clifford, KY", "Climax, KY", "Clinton, KY",
"Closplint, KY", "Clover Bottom, KY", "Cloverport, KY", "Coalgood, KY",
"Cobhill, KY", "Coldiron, KY", "Columbus, KY", "Combs, KY",
"Confluence, KY", "Constantine, KY", "Conway, KY", "Coopersville, KY",
"Corbin, KY", "Corinth, KY", "Cornishville, KY", "Corydon, KY",
"Cottle, KY", "Covington, KY", "Crab Orchard, KY", "Cranks, KY",
"Craynor, KY", "Crestwood, KY", "Crittenden, KY", "Crofton, KY",
"Cromwell, KY", "Cropper, KY", "Crown, KY", "Crummies, KY",
"Crutchfield, KY", "Cub Run, KY", "Cumberland, KY", "Cundiff, KY",
"Cunningham, KY", "Custer, KY", "Cutshin, KY", "Cynthiana, KY",
"Daisy, KY", "Dana, KY", "Danville, KY", "Darfork, KY", "Davella, KY",
"David, KY", "Davisport, KY", "Dawson Springs, KY", "Day Rural, KY",
"Dayhoit, KY", "Daysville, KY", "Dayton, KY", "Deane, KY",
"Deatsville, KY", "Decoy, KY", "Delphia, KY", "Delta, KY", "Dema, KY",
"Demossville, KY", "Denniston, KY", "Denton, KY", "Denver, KY",
"Dewitt, KY", "Dexter, KY", "Dice, KY", "Dixie, KY", "Dixon, KY",
"Dizney, KY", "Dover, KY", "Drakesboro, KY", "Dreyfus, KY",
"Dry Creek, KY", "Dry Ridge, KY", "Dryhill, KY", "Dubre, KY",
"Dundee, KY", "Dunmor, KY", "Dunnville, KY", "E Town, KY", "E View, KY",
"Earlington, KY", "East Mc Dowell, KY", "East Point, KY", "Eastern, KY",
"Eddyville, KY", "Edna, KY", "Eighty Eight, KY", "Ekron, KY",
"Elias, KY", "Elk Horn, KY", "Elkfork, KY", "Elkton, KY", "Elmrock, KY",
"Elna, KY", "Elsie, KY", "Eminence, KY", "Emma, KY", "Endicott, KY",
"Eolia, KY", "Erlanger, KY", "Ermine, KY", "Estill, KY", "Etoile, KY",
"Etty, KY", "Ewing, KY", "Ezel, KY", "Fairdale, KY", "Fairplay, KY",
"Falmouth, KY", "Fancy Farm, KY", "Farler, KY", "Farmington, KY",
"Fern Creek, KY", "Finchville, KY", "Finley, KY", "Firebrick, KY",
"Fisherville, KY", "Fishtrap, KY", "Fisty, KY", "Flat, KY",
"Flatwoods, KY", "Fleming Neon, KY", "Flemingsburg, KY", "Florence, KY",
"Fonde, KY", "Fordsville, KY", "Forest Hills, KY", "Fort Campbell, KY",
"Fort Knox, KY", "Fort Thomas, KY", "Foster, KY", "Fountain Run, KY",
"Franklin, KY", "Fredonia, KY", "Freeburn, KY", "Frew, KY", "Fuget, KY",
"Fultz, KY", "Galveston, KY", "Gamaliel, KY", "Garfield, KY",
"Garrett, KY", "Garrison, KY", "Gays Creek, KY", "Georgetown, KY",
"Germantown, KY", "Ghent, KY", "Gilbertsville, KY", "Gillmore, KY",
"Gilly, KY", "Girdler, KY", "Glasgow, KY", "Glen Dean, KY",
"Glencoe, KY", "Glendale, KY", "Glens Fork, KY", "Golden Pond, KY",
"Goshen, KY", "Gracey, KY", "Gradyville, KY", "Graham, KY",
"Grassy Creek, KY", "Gratz, KY", "Gravel Switch, KY", "Gray, KY",
"Grays Knob, KY", "Green Hall, KY", "Green Road, KY", "Greensburg, KY",
"Greenville, KY", "Grethel, KY", "Gulston, KY", "Guston, KY",
"Haddix, KY", "Hagerhill, KY", "Halo, KY", "Hampton, KY", "Hanson, KY",
"Happy, KY", "Hardin, KY", "Hardshell, KY", "Hardy, KY",
"Hardyville, KY", "Harold, KY", "Hartford, KY", "Hatton, KY",
"Hawesville, KY", "Hazel, KY", "Head Of Grassy, KY", "Hebron, KY",
"Heidrick, KY", "Henderson, KY", "Hendricks, KY", "Henshaw, KY",
"Herndon, KY", "Hestand, KY", "Hickman, KY", "Hickory, KY",
"High Bridge, KY", "Hillsboro, KY", "Hindman, KY", "Hinkle, KY",
"Hitchins, KY", "Hite, KY", "Hode, KY", "Hodgenville, KY",
"Holland, KY", "Hollybush, KY", "Hollyhill, KY", "Holmes Mill, KY",

"Honaker, KY", "Hopkinsville, KY", "Horse Branch, KY", "Horse Cave, KY",
"Howardstown, KY", "Huddy, KY", "Hudson, KY", "Huff, KY", "Hulen, KY",
"Hustonville, KY", "Independence, KY", "Irvington, KY", "Island, KY",
"Island City, KY", "Isom, KY", "Isonville, KY", "Iuka, KY", "Ivel, KY",
"Jabez, KY", "Jackhorn, KY", "Jamboree, KY", "Jamestown, KY",
"Jeffersontown, KY", "Jeffersonville, KY", "Jeremiah, KY", "Jetson, KY",
"Jinks, KY", "Job, KY", "Jonesville, KY", "Junction City, KY",
"Keaton, KY", "Keavy, KY", "Keene, KY", "Keith, KY", "Kenvir, KY",
"Kettle, KY", "Kettle Island, KY", "Kevil, KY", "Kimper, KY",
"Kings Mountain, KY", "Kirksey, KY", "Knifley, KY", "Knob Lick, KY",
"Kona, KY", "Korea, KY", "Kuttawa, KY", "Kyrock, KY", "La Center, KY",
"La Fayette, KY", "La Grange, KY", "Lackey, KY", "Lamb, KY",
"Lambric, KY", "Lamero, KY", "Lancaster, KY", "Langley, KY",
"Larkslane, KY", "Latonia, KY", "Laura, KY", "Lawrenceburg, KY",
"Lawton, KY", "Leander, KY", "Leatherwood, KY", "Lebanon, KY",
"Lebanon Junction, KY", "Ledbetter, KY", "Lee City, KY", "Leeco, KY",
"Lejunior, KY", "Lenox, KY", "Letcher, KY", "Lewis Creek, KY",
"Lewisburg, KY", "Lewisport, KY", "Lexington, KY", "Liberty, KY",
"Lick Creek, KY", "Ligon, KY", "Lily, KY", "Limestone Sq, KY",
"Lindseyville, KY", "Linefork, KY", "Littcarr, KY", "Little, KY",
"Livermore, KY", "Livingston, KY", "Lockport, KY", "Locust Hill, KY",
"Logansport, KY", "Loretto, KY", "Louellen, KY", "Louisville, KY",
"Lovely, KY", "Lucas, KY", "Ludlow, KY", "Lynch, KY", "Lyndon, KY",
"Lynn, KY", "Maceo, KY", "Mackville, KY", "Madisonville, KY",
"Magnolia, KY", "Mallie, KY", "Maloneton, KY", "Mammoth Cave Nat, KY",
"Manila, KY", "Manitou, KY", "Mariba, KY", "Marion, KY", "Martha, KY",
"Mary Alice, KY", "Maud, KY", "Mayfield, KY", "Mays Lick, KY",
"Mc Andrews, KY", "Mc Carr, KY", "Mc Combs, KY", "Mc Daniels, KY",
"Mc Veigh, KY", "Meally, KY", "Means, KY", "Melber, KY",
"Melbourne, KY", "Middleburg, KY", "Middlesboro, KY", "Middletown, KY",
"Midway, KY", "Mills, KY", "Millstone, KY", "Milltown, KY",
"Millwood, KY", "Milton, KY", "Mistletoe, KY", "Montpelier, KY",
"Mooleyville, KY", "Moorefield, KY", "Moores Creek, KY", "Morehead, KY",
"Morning View, KY", "Morris Fork, KY", "Mount Eden, KY",
"Mount Herman, KY", "Mount Olivet, KY", "Mount Sherman, KY",
"Mount Sterling, KY", "Mount Washington, KY", "Mousie, KY",
"Mouthcard, KY", "Mozelle, KY", "Munfordville, KY", "Murray, KY",
"Napfor, KY", "Narrows, KY", "Nebo, KY", "Nevisdale, KY",
"New Castle, KY", "New Concord, KY", "New Liberty, KY", "Newport, KY",
"Nicholasville, KY", "Nippa, KY", "Noctor, KY", "Nortonville, KY",
"Oak Grove, KY", "Oakland, KY", "Offutt, KY", "Okolona, KY",
"Olaton, KY", "Old Landing, KY", "Oldtown, KY", "Olmstead, KY",
"Olympia, KY", "Omaha, KY", "Oneida, KY", "Orlando, KY",
"Oven Fork, KY", "Owensboro, KY", "Owenton, KY", "Owingsville, KY",
"Paducah, KY", "Paint Lick, KY", "Paris, KY", "Park City, KY",
"Parkers Lake, KY", "Parksville, KY", "Partridge, KY", "Pathfork, KY",
"Patsey, KY", "Paw Paw, KY", "Payne Gap, KY", "Payneville, KY",
"Pembroke, KY", "Pendleton, KY", "Penrod, KY", "Perryville, KY",
"Petersburg, KY", "Pewee Valley, KY", "Peytonsburg, KY", "Phelps, KY",
"Philpot, KY", "Phyllis, KY", "Pine Ridge, KY", "Pinsonfork, KY",
"Pleasant View, KY", "Pleasure Ridge P, KY", "Plum Springs, KY",
"Pointer, KY", "Pomeroyton, KY", "Premium, KY", "Princeton, KY",
"Printer, KY", "Prospect, KY", "Providence, KY", "Pryse, KY",
"Pueblo, KY", "Pulaski, KY", "Puncheon, KY", "Putney, KY", "Pyramid, KY",
"Quality, KY", "Quicksand, KY", "Quincy, KY", "Rabbit Hash, KY",

"Raceland, KY", "Radcliff, KY", "Raven, KY", "Ravenna, KY",
 "Raywick, KY", "Redfox, KY", "Reed, KY", "Reedyville, KY", "Regina, KY",
 "Revelo, KY", "Reynolds Station, KY", "Rhodelia, KY", "Ricetown, KY",
 "Riceville, KY", "Richmond, KY", "Rineyville, KY", "River, KY",
 "Roark, KY", "Robards, KY", "Robinson Creek, KY", "Rochester, KY",
 "Rockholds, KY", "Rockport, KY", "Rocky Hill, KY", "Rockybranch, KY",
 "Rogers, KY", "Roundhill, KY", "Rouse, KY", "Rousseau, KY", "Rowdy, KY",
 "Roxana, KY", "Royalton, KY", "Rumsey, KY", "Rush, KY",
 "Sacramento, KY", "Sadieville, KY", "Sadler, KY", "Saint Catharine, KY",
 "Saint Charles, KY", "Saint Francis, KY", "Saint Matthews, KY",
 "Saint Paul, KY", "Saldee, KY", "Salem, KY", "Salt Gum, KY",
 "Salt Lick, KY", "Sanders, KY", "Sandgap, KY", "Sasser, KY",
 "Sawyer, KY", "Scalf, KY", "Science Hill, KY", "Scottsville, KY",
 "Scranton, KY", "Scuddy, KY", "Se Ree, KY", "Sebree, KY", "Seco, KY",
 "Sedalia, KY", "Seitz, KY", "Senterville, KY", "Sextons Creek, KY",
 "Sharon Grove, KY", "Sharpsburg, KY", "Shelbiana, KY", "Shelby Gap, KY",
 "Shelbyville, KY", "Shepherdsville, KY", "Shively, KY", "Shopville, KY",
 "Sidney, KY", "Siler, KY", "Silver Grove, KY", "Simpsonville, KY",
 "Sitka, KY", "Skyline, KY", "Slade, KY", "Slaughters, KY", "Slemp, KY",
 "Sloans Valley, KY", "Smilax, KY", "Smith, KY", "Smithfield, KY",
 "Smiths Grove, KY", "Soft Shell, KY", "Sonora, KY",
 "South Portsmouth, KY", "South Williamson, KY", "Southgate, KY",
 "Sparta, KY", "Speight, KY", "Spottsville, KY", "Stambaugh, KY",
 "Stamping Ground, KY", "Stanford, KY", "Stanville, KY", "Stearns, KY",
 "Steele, KY", "Stephens, KY", "Stephensburg, KY", "Stephensport, KY",
 "Stinnett, KY", "Stone, KY", "Stoney Fork, KY", "Strunk, KY",
 "Sturgis, KY", "Subtle, KY", "Sulphur, KY", "Summer Shade, KY",
 "Summersville, KY", "Sunfish, KY", "Symbol, KY", "Symsonia, KY",
 "T Ville, KY", "Talbert, KY", "Talcum, KY", "Tateville, KY",
 "Taylorsville, KY", "Teaberry, KY", "Thelma, KY", "Thornton, KY",
 "Tiline, KY", "Tiny Town, KY", "Tollesboro, KY", "Tottz, KY",
 "Trappist, KY", "Trenton, KY", "Tribbey, KY", "Trinity, KY",
 "Trosper, KY", "Turkey Creek, KY", "Turners Station, KY",
 "Tutor Key, KY", "Ulvah, KY", "Union, KY", "Union Star, KY",
 "Uniontown, KY", "Upton, KY", "Utica, KY", "Vada, KY",
 "Valley Station, KY", "Van Lear, KY", "Vancleve, KY", "Varney, KY",
 "Verona, KY", "Versailles, KY", "Vest, KY", "Vicco, KY", "Vincent, KY",
 "Vine Grove, KY", "Waddy, KY", "Waldo, KY", "Walker, KY",
 "Wallingford, KY", "Wallins Creek, KY", "Walton, KY", "Warsaw, KY",
 "Water Valley, KY", "Waverly, KY", "Wax, KY", "Wayland, KY",
 "Waynesburg, KY", "Webbs Cross Road, KY", "Webbville, KY",
 "Webster, KY", "Welchs Creek, KY", "Wendover, KY", "West Paducah, KY",
 "West Point, KY", "Westbend, KY", "Westport, KY", "Westview, KY",
 "Westwood, KY", "Whick, KY", "White Mills, KY", "White Oak, KY",
 "White Plains, KY", "Whitehouse, KY", "Whitesville, KY", "Wiborg, KY",
 "Wickliffe, KY", "Williamsport, KY", "Williamstown, KY",
 "Willisburg, KY", "Willow Shade, KY", "Winchester, KY", "Windy, KY",
 "Wingo, KY", "Wittensville, KY", "Woodbine, KY", "Woodburn, KY",
 "Woollum, KY", "Worthington, KY", "Worthville, KY", "Yosemite, KY",
 "Zachariah, KY", "Zoe, KY", "Abita Springs, LA", "Acme, LA",
 "Addis, LA", "Aimwell, LA", "Albany, LA", "Alexandria, LA", "Alto, LA",
 "Ama, LA", "Amelia, LA", "Amite, LA", "Anacoco, LA", "Angie, LA",
 "Angola, LA", "Arabi, LA", "Arcadia, LA", "Arnaudville, LA",
 "Athens, LA", "Atlanta, LA", "Bains, LA", "Baker, LA", "Baldwin, LA",
 "Barataria, LA", "Barksdale A F B, LA", "Basile, LA", "Baskin, LA",

"Bastrop, LA", "Batchelor, LA", "Baton Rouge, LA", "Belcher, LA",
 "Bell City, LA", "Belle Chasse, LA", "Belle Rose, LA", "Belmont, LA",
 "Bentley, LA", "Benton, LA", "Bernice, LA", "Berwick, LA",
 "Bethany, LA", "Bienville, LA", "Big Bend, LA", "Blanks, LA",
 "Bogalusa, LA", "Bonita, LA", "Bossier City, LA", "Bourg, LA",
 "Boutte, LA", "Boyce, LA", "Braithwaite, LA", "Branch, LA",
 "Bridge City, LA", "Broussard, LA", "Brusly, LA", "Buckeye, LA",
 "Bueche, LA", "Buras, LA", "Bush, LA", "Calcasieu, LA", "Calhoun, LA",
 "Cameron, LA", "Campti, LA", "Cankton, LA", "Carencro, LA",
 "Caspiana, LA", "Castor, LA", "Center Point, LA", "Chalmette, LA",
 "Chatham, LA", "Chauvin, LA", "Cheneyville, LA", "Chestnut, LA",
 "Chopin, LA", "Choudrant, LA", "Church Point, LA", "Clayton, LA",
 "Clifton, LA", "Clinton, LA", "Colfax, LA", "Collinston, LA",
 "Convent, LA", "Cotton Valley, LA", "Cottonport, LA", "Covington, LA",
 "Creole, LA", "Crowley, LA", "Cut Off, LA", "Darrow, LA",
 "Delcambre, LA", "Dequincy, LA", "Deridder, LA", "Derry, LA",
 "Des Allemands, LA", "Dixie, LA", "Dodson, LA", "Donaldsonville, LA",
 "Downsville, LA", "Doyline, LA", "Dry Creek, LA", "Dry Prong, LA",
 "Dubach, LA", "Dubberly, LA", "Dulac, LA", "Duson, LA", "Edgard, LA",
 "Egan, LA", "Elm Grove, LA", "Elmer, LA", "Elton, LA", "Enterprise, LA",
 "Eola, LA", "Epps, LA", "Erath, LA", "Eros, LA", "Erwinville, LA",
 "Ethel, LA", "Eunice, LA", "Evangeline, LA", "Evans, LA",
 "Extension, LA", "Farmerville, LA", "Fields, LA", "Fisher, LA",
 "Flatwoods, LA", "Florien, LA", "Fluker, LA", "Folsom, LA",
 "Forbing, LA", "Fordoche, LA", "Forest Hill, LA", "Forked Island, LA",
 "Fort Necessity, LA", "Fort Polk, LA", "Franklin, LA",
 "Franklinton, LA", "French Settlement, LA", "Frierson, LA",
 "Frogmore, LA", "Galliano, LA", "Galvez, LA", "Garyville, LA",
 "Geismar, LA", "Georgetown, LA", "Gheens, LA", "Gibbsland, LA",
 "Gibson, LA", "Gilbert, LA", "Gilliam, LA", "Gloster, LA", "Glynn, LA",
 "Golden Meadow, LA", "Goldonna, LA", "Gonzales, LA", "Goudeau, LA",
 "Grambling, LA", "Gramercy, LA", "Grand Cane, LA", "Grand Chenier, LA",
 "Grand Isle, LA", "Gray, LA", "Grayson, LA", "Greensburg, LA",
 "Greenwell Spring, LA", "Greenwood, LA", "Gretna, LA",
 "Grosse Tete, LA", "Gueydan, LA", "Hackberry, LA", "Hahnville, LA",
 "Hall Summit, LA", "Hamburg, LA", "Hammond, LA", "Hanna, LA",
 "Harahan, LA", "Harrisonburg, LA", "Harvey, LA", "Haughton, LA",
 "Haynesville, LA", "Hebert, LA", "Heflin, LA", "Henderson, LA",
 "Hessmer, LA", "Hicks, LA", "Holden, LA", "Homer, LA", "Hornbeck, LA",
 "Hosston, LA", "Houma, LA", "Iberville, LA", "Ida, LA",
 "Independence, LA", "Iota, LA", "Iowa, LA", "Jamestown, LA",
 "Jarreau, LA", "Jeanerette, LA", "Jefferson, LA", "Jena, LA",
 "Jennings, LA", "Jones, LA", "Jonesboro, LA", "Kaplan, LA",
 "Keatchie, LA", "Keithville, LA", "Kelly, LA", "Kenner, LA",
 "Kentwood, LA", "Kinder, LA", "Kolin, LA", "Krotz Springs, LA",
 "La Place, LA", "Labadieville, LA", "Lacamp, LA", "Lacassine, LA",
 "Lacombe, LA", "Lafayette, LA", "Lafitte, LA", "Lake Arthur, LA",
 "Lake Charles, LA", "Lake Providence, LA", "Lakeland, LA", "Larto, LA",
 "Le Moyne, LA", "Leander, LA", "Lecompte, LA", "Lettsworth, LA",
 "Lillie, LA", "Linville, LA", "Lisbon, LA", "Livingston, LA",
 "Livonia, LA", "Lockport, LA", "Logansport, LA", "Longville, LA",
 "Loranger, LA", "Loreauville, LA", "Lottie, LA", "Luling, LA",
 "Lutcher, LA", "Madisonville, LA", "Mamou, LA", "Mandeville, LA",
 "Mangham, LA", "Mansfield, LA", "Mansura, LA", "Many, LA",
 "Marksville, LA", "Marrero, LA", "Marthaville, LA", "Mathews, LA",

"Maurepas, LA", "Maurice, LA", "Melder, LA", "Melville, LA",
 "Mer Rouge, LA", "Meraux, LA", "Metairie, LA", "Midland, LA",
 "Minden, LA", "Mira, LA", "Mitchell, LA", "Mittie, LA", "Monroe, LA",
 "Montegut, LA", "Monterey, LA", "Montgomery, LA", "Mooringsport, LA",
 "Moreauville, LA", "Morgan City, LA", "Morganza, LA", "Mound, LA",
 "Mount Hermon, LA", "Napoleonville, LA", "Natchez, LA",
 "Natchitoches, LA", "New Iberia, LA", "New Orleans, LA",
 "New Roads, LA", "New Sarpy, LA", "Newellton, LA", "Newllano, LA",
 "Noble, LA", "Norco, LA", "Norwood, LA", "Oak Ridge, LA", "Oakdale, LA",
 "Oberlin, LA", "Oil City, LA", "Olla, LA", "Opelousas, LA", "Oscar, LA",
 "Otis, LA", "Palmetto, LA", "Paradis, LA", "Patterson, LA",
 "Paulina, LA", "Pearl River, LA", "Pelican, LA", "Pierre Part, LA",
 "Pine Grove, LA", "Pioneer, LA", "Pitkin, LA", "Plain Dealing, LA",
 "Plaquemine, LA", "Plaucheville, LA", "Pleasant Hill, LA",
 "Point Clair, LA", "Pollock, LA", "Ponchatoula, LA", "Port Allen, LA",
 "Port Barre, LA", "Port Sulphur, LA", "Port Vincent, LA", "Pride, LA",
 "Princeton, LA", "Provencal, LA", "Quitman, LA", "Raceland, LA",
 "Ragley, LA", "Ramah, LA", "Rayne, LA", "Reeves, LA", "Reserve, LA",
 "Richwood, LA", "Ringgold, LA", "Roanoke, LA", "Robeline, LA",
 "Robert, LA", "Rodessa, LA", "Rosedale, LA", "Roseland, LA",
 "Rougon, LA", "Ruston, LA", "Saint Amant, LA", "Saint Bernard, LA",
 "Saint James, LA", "Saint Joseph, LA", "Saint Landry, LA",
 "Saint Martinvill, LA", "Saint Rose, LA", "Sarepta, LA",
 "Schriever, LA", "Scotlandville, LA", "Scott, LA", "Shongaloo, LA",
 "Shreveport, LA", "Sibley, LA", "Sicily Island, LA", "Sieper, LA",
 "Sikes, LA", "Simmesport, LA", "Simsboro, LA", "Singer, LA",
 "Slaughter, LA", "Slidell, LA", "Sondheimer, LA", "Sorrento, LA",
 "Spearsville, LA", "Spencer, LA", "Springfield, LA", "Springhill, LA",
 "Starks, LA", "Stonewall, LA", "Sugartown, LA", "Sulphur, LA",
 "Summerfield, LA", "Sunshine, LA", "Terry, LA", "Terrytown, LA",
 "The Bluffs, LA", "Theriot, LA", "Thibodaux, LA", "Tickfaw, LA",
 "Tioga, LA", "Torbert, LA", "Transylvania, LA", "Trees, LA",
 "Trout, LA", "Tullos, LA", "Uncle Sam, LA", "Vacherie, LA",
 "Varnado, LA", "Venice, LA", "Ventress, LA", "Vick, LA", "Vidalia, LA",
 "Ville Platte, LA", "Vinton, LA", "Violet, LA", "Walker, LA",
 "Warden, LA", "Washington, LA", "Waterproof, LA", "Welsh, LA",
 "West Monroe, LA", "Westlake, LA", "White Castle, LA", "Wilson, LA",
 "Winnfield, LA", "Winnsboro, LA", "Wisner, LA", "Woodworth, LA",
 "Youngsville, LA", "Zachary, LA", "Zwolle, LA", "Abington, MA",
 "Acton, MA", "Acushnet, MA", "Adams, MA", "Agawam, MA", "Allston, MA",
 "Amesbury, MA", "Andover, MA", "Arlington, MA", "Ashburnham, MA",
 "Ashby, MA", "Ashfield, MA", "Ashland, MA", "Ashley Falls, MA",
 "Assonet, MA", "Attleboro, MA", "Auburn, MA", "Auburndale, MA",
 "Avon, MA", "Ayer, MA", "Baldwinville, MA", "Barnstable, MA",
 "Barre, MA", "Bass River, MA", "Becket, MA", "Bedford, MA",
 "Belchertown, MA", "Bellingham, MA", "Belmont, MA", "Berkley, MA",
 "Berlin, MA", "Beverly, MA", "Billerica, MA", "Blackstone, MA",
 "Blandford, MA", "Bolton, MA", "Boston, MA", "Boston College, MA",
 "Boxboro, MA", "Boxford, MA", "Boylston, MA", "Bradford, MA",
 "Braintree, MA", "Brewster, MA", "Bridgewater, MA", "Brighton, MA",
 "Brimfield, MA", "Brockton, MA", "Brookfield, MA", "Brookline, MA",
 "Buckland, MA", "Burlington, MA", "Byfield, MA", "Cambridge, MA",
 "Canton, MA", "Carlisle, MA", "Carver, MA", "Centerville, MA",
 "Charlestown, MA", "Charlton, MA", "Chelsea, MA", "Cherry Valley, MA",
 "Cheshire, MA", "Chester, MA", "Chesterfield, MA", "Chicopee, MA",

"Chilmark, MA", "Clarksburg, MA", "Clinton, MA", "Cohasset, MA",
"Colrain, MA", "Concord, MA", "Conway, MA", "Cotuit, MA",
"Cummington, MA", "Cushman, MA", "Cuttyhunk, MA", "Dalton, MA",
"Danvers, MA", "Dedham, MA", "Deerfield, MA", "Dennis, MA",
"Dennis Port, MA", "Dighton, MA", "Dorchester, MA", "Dover, MA",
"Dracut, MA", "Dudley, MA", "Dudley Hill, MA", "Dunstable, MA",
"Duxbury, MA", "East Boston, MA", "East Bridgewater, MA",
"East Brookfield, MA", "East Cambridge, MA", "East Douglas, MA",
"East Freetown, MA", "East Longmeadow, MA", "East Lynn, MA",
"East Sandwich, MA", "East Taunton, MA", "East Walpole, MA",
"East Wareham, MA", "East Watertown, MA", "Eastham, MA",
"Edgartown, MA", "Erving, MA", "Essex, MA", "Everett, MA",
"Fairhaven, MA", "Fall River, MA", "Falmouth, MA", "Feeding Hills, MA",
"Fiskdale, MA", "Fitchburg, MA", "Florence, MA", "Forestdale, MA",
"Foxboro, MA", "Framingham, MA", "Franklin, MA", "Ft Devens, MA",
"Gardner, MA", "Georgetown, MA", "Gilbertville, MA", "Gloucester, MA",
"Goshen, MA", "Grafton, MA", "Granby, MA", "Graniteville, MA",
"Great Barrington, MA", "Groton, MA", "Groveland, MA", "Hadley, MA",
"Halifax, MA", "Hampden, MA", "Hancock, MA", "Hanover, MA",
"Hanson, MA", "Harvard, MA", "Harwich, MA", "Harwich Port, MA",
"Hatfield, MA", "Haverhill, MA", "Hawley, MA", "Haydenville, MA",
"Heath, MA", "Hingham, MA", "Holbrook, MA", "Holden, MA", "Holland, MA",
"Holliston, MA", "Holyoke, MA", "Hopedale, MA", "Hopkinton, MA",
"Housatonic, MA", "Hubbardston, MA", "Hudson, MA", "Hull, MA",
"Huntington, MA", "Hyde Park, MA", "Indian Orchard, MA", "Ipswich, MA",
"Jamaica Plain, MA", "Jefferson, MA", "Kingston, MA", "Lakeville, MA",
"Lancaster, MA", "Lawrence, MA", "Lee, MA", "Leeds, MA",
"Leicester, MA", "Lenox, MA", "Leominster, MA", "Leverett, MA",
"Lexington, MA", "Leyden, MA", "Lincoln, MA", "Littleton, MA",
"Longmeadow, MA", "Lowell, MA", "Ludlow, MA", "Lunenburg, MA",
"Lynn, MA", "Lynnfield, MA", "Malden, MA", "Manchester, MA",
"Mansfield, MA", "Marblehead, MA", "Marion, MA", "Marlborough, MA",
"Marshfield, MA", "Marstons Mills, MA", "Mashpee, MA", "Mattapan, MA",
"Mattapoisett, MA", "Maynard, MA", "Medfield, MA", "Medford, MA",
"Medway, MA", "Melrose, MA", "Mendon, MA", "Merrimac, MA",
"Methuen, MA", "Middleboro, MA", "Middlefield, MA", "Middleton, MA",
"Milford, MA", "Millbury, MA", "Millers Falls, MA", "Millis, MA",
"Millville, MA", "Milton, MA", "Monroe, MA", "Monson, MA",
"Montague, MA", "Montgomery, MA", "Mount Tom, MA", "Nahant, MA",
"Nantucket, MA", "Natick, MA", "Needham, MA", "New Bedford, MA",
"New Braintree, MA", "New Salem, MA", "Newbury, MA", "Newburyport, MA",
"Newton Center, MA", "Newton Highlands, MA", "Newton Upper Fal, MA",
"Newtonville, MA", "Norfolk, MA", "North Andover, MA",
"North Attleboro, MA", "North Billerica, MA", "North Brookfield, MA",
"North Cambridge, MA", "North Chatham, MA", "North Chelmsford, MA",
"North Dartmouth, MA", "North Dighton, MA", "North Easton, MA",
"North Falmouth, MA", "North Grafton, MA", "North Oxford, MA",
"North Reading, MA", "North Truro, MA", "North Waltham, MA",
"Northborough, MA", "Northbridge, MA", "Northfield, MA", "Norton, MA",
"Norwell, MA", "Norwood, MA", "Oakham, MA", "Onset, MA", "Orleans, MA",
"Osterville, MA", "Otis, MA", "Otis A F B, MA", "Oxford, MA",
"Padanaram Villag, MA", "Palmer, MA", "Paxton, MA", "Peabody, MA",
"Pembroke, MA", "Pepperell, MA", "Peru, MA", "Petersham, MA",
"Pittsfield, MA", "Plainfield, MA", "Plainville, MA", "Plymouth, MA",
"Plympton, MA", "Pocasset, MA", "Princeton, MA", "Provincetown, MA",

"Quincy, MA", "Randolph, MA", "Raynham, MA", "Reading, MA",
 "Rehoboth, MA", "Revere, MA", "Richmond, MA", "Rochdale, MA",
 "Rochester, MA", "Rockland, MA", "Rockport, MA", "Roslindale, MA",
 "Rowe, MA", "Rowley, MA", "Roxbury, MA", "Russell, MA", "Rutland, MA",
 "Salem, MA", "Salisbury, MA", "Sandisfield, MA", "Sandwich, MA",
 "Saugus, MA", "Savoy, MA", "Scituate, MA", "Seekonk, MA", "Sharon, MA",
 "Sheffield, MA", "Shelburne Falls, MA", "Sherborn, MA",
 "Shirley Center, MA", "Shrewsbury, MA", "Shutesbury, MA",
 "Somerset, MA", "Somerville, MA", "South Boston, MA",
 "South Chatham, MA", "South Chelmsford, MA", "South Deerfield, MA",
 "South Dennis, MA", "South Easton, MA", "South Egremont, MA",
 "South Grafton, MA", "South Hadley, MA", "South Hamilton, MA",
 "South Walpole, MA", "Southampton, MA", "Southborough, MA",
 "Southbridge, MA", "Southfield, MA", "Southwick, MA", "Spencer, MA",
 "Springfield, MA", "Sterling, MA", "Stockbridge, MA", "Stoneham, MA",
 "Stoughton, MA", "Stow, MA", "Sturbridge, MA", "Sudbury, MA",
 "Sunderland, MA", "Swampscott, MA", "Swansea, MA", "Taunton, MA",
 "Teaticket, MA", "Templeton, MA", "Tewksbury, MA", "Three Rivers, MA",
 "Tolland, MA", "Topsfield, MA", "Townsend, MA", "Truro, MA",
 "Turners Falls, MA", "Tyngsboro, MA", "Uxbridge, MA",
 "Village Of Nagog, MA", "Vineyard Haven, MA", "W Townsend, MA",
 "Waban, MA", "Wakefield, MA", "Wales, MA", "Walpole, MA", "Ware, MA",
 "Wareham, MA", "Wayland, MA", "Wellesley, MA", "Wellfleet, MA",
 "Wendell, MA", "Wenham, MA", "West Barnstable, MA", "West Boylston, MA",
 "West Bridgewater, MA", "West Brookfield, MA", "West Dennis, MA",
 "West Harwich, MA", "West Lynn, MA", "West Newbury, MA",
 "West Otis, MA", "West Roxbury, MA", "West Springfield, MA",
 "West Stockbridge, MA", "West Tisbury, MA", "West Upton, MA",
 "West Wareham, MA", "West Warren, MA", "West Yarmouth, MA",
 "Westborough, MA", "Westminster, MA", "Weston, MA", "Westover Afb, MA",
 "Westport, MA", "Westwood, MA", "Weymouth, MA", "Whitinsville, MA",
 "Whitman, MA", "Wilbraham, MA", "Wilkinsonville, MA",
 "Williamsburg, MA", "Williamstown, MA", "Wilmington, MA",
 "Winchendon, MA", "Winchester, MA", "Windsor, MA", "Winthrop, MA",
 "Woburn, MA", "Woods Hole, MA", "Worcester, MA", "Worthington, MA",
 "Wrentham, MA", "Yarmouth Port, MA", "Abell, MD", "Aberdeen, MD",
 "Aberdeen Proving, MD", "Abingdon, MD", "Accident, MD", "Accokeek, MD",
 "Adelphi, MD", "Andrews Afb, MD", "Annapolis, MD",
 "Annapolis Juncti, MD", "Aquasco, MD", "Arnold, MD", "Ashton, MD",
 "Aspen Hill, MD", "Avenue, MD", "Bainbridge, MD", "Baldwin, MD",
 "Baltimore, MD", "Barclay, MD", "Barnesville, MD", "Barton, MD",
 "Beallsville, MD", "Bel Air, MD", "Bel Alton, MD", "Belcamp, MD",
 "Beltsville, MD", "Bentley Springs, MD", "Berlin, MD", "Bethesda, MD",
 "Betterton, MD", "Big Pool, MD", "Big Spring, MD", "Bishopville, MD",
 "Bittering, MD", "Bivalve, MD", "Bladensburg, MD", "Bloomington, MD",
 "Bowie, MD", "Boysds, MD", "Bozman, MD", "Bradshaw, MD",
 "Brandywine, MD", "Brentwood, MD", "Brinklow, MD", "Brookeville, MD",
 "Brooklyn Curtis, MD", "Broomes Island, MD", "Brunswick, MD",
 "Bryans Road, MD", "Bryantown, MD", "Burkittsville, MD",
 "Burtonsville, MD", "Bushwood, MD", "Cabin John, MD", "California, MD",
 "Callaway, MD", "Cambridge, MD", "Cape Saint Clair, MD",
 "Capital Heights, MD", "Carrollton, MD", "Carrolltowne, MD",
 "Catonsville, MD", "Cecilton, MD", "Centreville, MD", "Chance, MD",
 "Charlestown, MD", "Charlotte Hall, MD", "Cheltenham, MD",
 "Chesapeake Beach, MD", "Chesapeake City, MD", "Chester, MD",

"Chestertown, MD", "Chevy Chase, MD", "Church Creek, MD",
"Church Hill, MD", "Churchton, MD", "Churchville, MD", "Clarksburg, MD",
"Clarksville, MD", "Clements, MD", "Clinton, MD",
"Cockeysville Hun, MD", "Colesville, MD", "College Park, MD",
"Colora, MD", "Coltons Point, MD", "Columbia, MD", "Conowingo, MD",
"Cooksville, MD", "Cordova, MD", "Crapo, MD", "Cresaptown, MD",
"Crisfield, MD", "Crofton, MD", "Crownsville, MD", "Crumpton, MD",
"Damascus, MD", "Dameron, MD", "Dames Quarter, MD", "Daniels, MD",
"Darlington, MD", "Darnestown, MD", "Davidsonville, MD", "Dayton, MD",
"Deal Island, MD", "Deale, MD", "Deer Park, MD", "Delmar, MD",
"Denton, MD", "Derwood, MD", "Detour, MD", "Dickerson, MD",
"District Heights, MD", "Doubs, MD", "Drayden, MD",
"Dundalk Sparrows, MD", "Dunkirk, MD", "Earleville, MD",
"East New Market, MD", "Easton, MD", "Eden, MD", "Edgewater Beach, MD",
"Edgewood, MD", "Elkton, MD", "Ellicott City, MD", "Emmitsburg, MD",
"Essex, MD", "Eudowood, MD", "Ewell, MD", "Fahrney Keedy Me, MD",
"Fair Play, MD", "Fallston, MD", "Faulkner, MD", "Federalburg, MD",
"Fishing Creek, MD", "Flintstone, MD", "Forest Hill, MD", "Fork, MD",
"Fort Detrick, MD", "Fort George G Me, MD", "Fort Ritchie, MD",
"Fort Washington, MD", "Fowbelsburg, MD", "Freeland, MD",
"Friendship, MD", "Friendsville, MD", "Frostburg, MD", "Fruitland, MD",
"Fulton, MD", "Gaithersburg, MD", "Galena, MD", "Gambrills, MD",
"Germantown, MD", "Gibson Island, MD", "Girdletree, MD", "Glen Arm, MD",
"Glen Burnie, MD", "Glen Echo, MD", "Glencoe, MD", "Glenelg, MD",
"Glenn Dale, MD", "Glenwood, MD", "Glyndon, MD", "Goldsboro, MD",
"Golts, MD", "Graceham, MD", "Granite, MD", "Grasonville, MD",
"Great Mills, MD", "Greenbelt, MD", "Greenmount, MD", "Greensboro, MD",
"Gunpowder, MD", "Gwynn Oak, MD", "Hagerstown, MD", "Halethorpe, MD",
"Hancock, MD", "Hanover, MD", "Harwood, MD", "Havre De Grace, MD",
"Hebron, MD", "Henderson, MD", "Hereford, MD", "Highland, MD",
"Hollywood, MD", "Hughesville, MD", "Huntingtown, MD",
"Hyattsville, MD", "Hydes, MD", "Ijamsville, MD", "Ingleside, MD",
"Issue, MD", "Jacksonville, MD", "Jarrettsville, MD", "Jefferson, MD",
"Jennings, MD", "Jessup, MD", "Joppa, MD", "Keedysville, MD",
"Kennedyville, MD", "Kensington, MD", "Keymar, MD", "Kingsville, MD",
"Knoxville, MD", "La Plata, MD", "Landover, MD", "Landover Hills, MD",
"Lanham, MD", "Laurel, MD", "Laytonsville, MD", "Leonardtwn, MD",
"Lewistown, MD", "Lexington Park, MD", "Lineboro, MD", "Linkwood, MD",
"Linthicum Height, MD", "Linwood, MD", "Little Orleans, MD",
"Lonaconing, MD", "Lothian, MD", "Loveville, MD", "Luke, MD",
"Lusby, MD", "Lutherville, MD", "Maddox, MD", "Manchester, MD",
"Mardela Springs, MD", "Marion Station, MD", "Marriottsville, MD",
"Marydel, MD", "Massey, MD", "Maugansville, MD", "McCoole, MD",
"Mcdaniel, MD", "Mechanicsville, MD", "Middle River, MD",
"Middletown, MD", "Millers, MD", "Millersville, MD", "Millington, MD",
"Mitchellville, MD", "Monrovia, MD", "Montpelier, MD", "Mount Airy, MD",
"Mount Rainier, MD", "Mount Savage, MD", "Myersville, MD",
"Nanjemoy, MD", "Nanticoke, MD", "Naval Academy, MD", "New Windsor, MD",
"Newark, MD", "Newburg, MD", "North Beach, MD", "North East, MD",
"Nottingham, MD", "Ocean City, MD", "Odenton, MD", "Oldtown, MD",
"Olney, MD", "Owings, MD", "Owings Mills, MD", "Oxford, MD",
"Oxon Hill, MD", "Park Hall, MD", "Parkville, MD", "Parsonsborg, MD",
"Patapsco, MD", "Patuxent River, MD", "Perry Hall, MD",
"Perryville, MD", "Pikesville, MD", "Piney Point, MD", "Pisgah, MD",
"Pittsville, MD", "Pocomoke City, MD", "Point Of Rocks, MD",

"Pomfret, MD", "Poolesville, MD", "Port Republic, MD",
 "Port Tobacco, MD", "Potomac, MD", "Preston, MD",
 "Prince Frederick, MD", "Princess Anne, MD", "Pylesville, MD",
 "Quantico, MD", "Queen Anne, MD", "Queenstown, MD", "Randallstown, MD",
 "Rawlings, MD", "Reisterstown, MD", "Rhodesdale, MD", "Ridge, MD",
 "Ridgely, MD", "Rising Sun, MD", "Rison, MD", "Riva, MD",
 "Riverdale, MD", "Riviera Beach, MD", "Rock Hall, MD", "Rocks, MD",
 "Rockville, MD", "Rocky Ridge, MD", "Rohrersville, MD", "Rosedale, MD",
 "Royal Oak, MD", "Sabillasville, MD", "Saint Charles, MD",
 "Saint Inigoes, MD", "Saint Leonard, MD", "Saint Michaels, MD",
 "Salisbury, MD", "Sandy Spring, MD", "Sang Run, MD", "Savage, MD",
 "Scotland, MD", "Severn, MD", "Severna Park, MD", "Shady Side, MD",
 "Shallmar, MD", "Sharpsburg, MD", "Sherwood, MD", "Silver Spring, MD",
 "Smithsburg, MD", "Snow Hill, MD", "Solomons, MD", "Spencerville, MD",
 "Stevensville, MD", "Still Pond, MD", "Stockton, MD",
 "Sudlersville, MD", "Suitland, MD", "Sunderland, MD", "Swanton, MD",
 "Takoma Park, MD", "Tall Timbers, MD", "Taneytown, MD",
 "Taylors Island, MD", "Temple Hills, MD", "Tilghman, MD",
 "Toddville, MD", "Tracys Landing, MD", "Trappe, MD", "Tuscarora, MD",
 "Tyaskin, MD", "Tylerton, MD", "Uniontown, MD", "Unionville, MD",
 "Upper Falls, MD", "Upper Marlboro, MD", "Valley Lee, MD", "Vienna, MD",
 "Waldorf, MD", "Walkersville, MD", "Warwick, MD", "Welcome, MD",
 "Wenona, MD", "West Bethesda, MD", "West Friendship, MD",
 "West Hyattsville, MD", "West River, MD", "Westover, MD",
 "Whaleysville, MD", "Wheaton, MD", "White Hall, MD", "White Marsh, MD",
 "White Plains, MD", "Whiteford, MD", "Willards, MD", "Williamsburg, MD",
 "Williamsport, MD", "Wingate, MD", "Wittman, MD", "Woodbine, MD",
 "Woodsboro, MD", "Woolford, MD", "Worton, MD", "Wye Mills, MD",
 "Abbot Village, ME", "Acton, ME", "Addison, ME", "Albion, ME",
 "Alfred, ME", "Andover, ME", "Anson, ME", "Arundel, ME", "Ashland, ME",
 "Athens, ME", "Auburn, ME", "Augusta, ME", "Aurora, ME",
 "Bailey Island, ME", "Bangor, ME", "Bar Harbor, ME", "Bass Harbor, ME",
 "Bath, ME", "Beals, ME", "Belfast, ME", "Belgrade, ME",
 "Belgrade Lakes, ME", "Benedicta, ME", "Benton Station, ME",
 "Bernard, ME", "Berwick, ME", "Bethel, ME", "Biddeford Pool, ME",
 "Bingham, ME", "Birch Harbor, ME", "Birch Island, ME", "Blue Hill, ME",
 "Blue Hill Falls, ME", "Boothbay, ME", "Bowdoinham, ME", "Bradford, ME",
 "Bradley, ME", "Brewer, ME", "Bridgewater, ME", "Bridgton, ME",
 "Bristol, ME", "Brooklin, ME", "Brooks, ME", "Brooksville, ME",
 "Brookton, ME", "Brownfield, ME", "Brownville, ME", "Bryant Pond, ME",
 "Buckfield, ME", "Bucks Harbor, ME", "Bucksport, ME", "Burlington, ME",
 "Burnham, ME", "Bustins Island, ME", "Calais, ME", "Cambridge, ME",
 "Camden, ME", "Canaan, ME", "Canton, ME", "Cape Elizabeth, ME",
 "Cape Neddick, ME", "Capitol Island, ME", "Caratunk, ME",
 "Cardville, ME", "Caribou, ME", "Carmel, ME", "Casco, ME",
 "Center Lovell, ME", "Chamberlain, ME", "Charleston, ME",
 "Chebeague Island, ME", "Cherryfield, ME", "Clayton Lake, ME",
 "Cliff Island, ME", "Clinton, ME", "Columbia Falls, ME",
 "Coopers Mills, ME", "Corea, ME", "Corinna, ME", "Cornish, ME",
 "Costigan, ME", "Cumberland Cente, ME", "Cumberland Fores, ME",
 "Cushing, ME", "Cushing Island, ME", "Cutler, ME", "Damariscotta, ME",
 "Danforth, ME", "Danville, ME", "Deer Isle, ME", "Denmark, ME",
 "Dennysville, ME", "Derby, ME", "Detroit, ME", "Dexter, ME",
 "Dixfield, ME", "Dixmont, ME", "Dover Foxcroft, ME", "Dresden, ME",
 "Dryden, ME", "East Andover, ME", "East Baldwin, ME",

"East Boothbay, ME", "East Corinth, ME", "East Eddington, ME",
"East Holden, ME", "East Livermore, ME", "East Machias, ME",
"East Millinocket, ME", "East Orland, ME", "East Stoneham, ME",
"East Waterboro, ME", "Easton, ME", "Eastport, ME", "Edgecomb, ME",
"Eliot, ME", "Ellsworth, ME", "Enfield, ME", "Etna, ME", "Eustis, ME",
"Exeter, ME", "Falmouth, ME", "Farmingdale, ME", "Farmington, ME",
"Fort Fairfield, ME", "Fort Kent, ME", "Frankfort, ME", "Franklin, ME",
"Freedom, ME", "Freeport, ME", "Friendship, ME", "Frye, ME",
"Fryeburg, ME", "Gardiner, ME", "Gorham, ME", "Gouldsboro, ME",
"Grand Isle, ME", "Gray, ME", "Greene, ME", "Greenville, ME",
"Greenville Junct, ME", "Guilford, ME", "Hallowell, ME", "Hampden, ME",
"Hancock, ME", "Hanover, ME", "Harborside, ME", "Harrington, ME",
"Harrison, ME", "Hartland, ME", "Haynesville, ME", "Hebron, ME",
"Hiram, ME", "Hollis Center, ME", "Hope, ME", "Houlton, ME",
"Hudson, ME", "Island Falls, ME", "Isle Au Haut, ME", "Islesboro, ME",
"Jackman, ME", "Jay, ME", "Jefferson, ME", "Jonesboro, ME",
"Jonesport, ME", "Kenduskeag, ME", "Kennebunk, ME", "Kennebunkport, ME",
"Kents Hill, ME", "Kezar Falls, ME", "Kingfield, ME", "Kingman, ME",
"Kittery, ME", "Kittery Point, ME", "Lagrange, ME", "Lee, ME",
"Leeds, ME", "Levant, ME", "Lewiston, ME", "Liberty, ME", "Lille, ME",
"Limerick, ME", "Limestone, ME", "Limington, ME", "Lincoln, ME",
"Lincoln Center, ME", "Lincolnton, ME", "Lisbon, ME",
"Lisbon Falls, ME", "Litchfield, ME", "Little Deer Isle, ME",
"Livermore Falls, ME", "Long Island, ME", "Loring Afb, ME",
"Lovell, ME", "Lubec, ME", "Mac Mahan, ME", "Machias, ME",
"Machiasport, ME", "Madawaska, ME", "Madison, ME", "Manchester, ME",
"Manset, ME", "Mapleton, ME", "Maplewood, ME", "Mars Hill, ME",
"Mattawamkeag, ME", "Mechanic Falls, ME", "Meddybemps, ME",
"Medomak, ME", "Medway, ME", "Merepoint, ME", "Mexico, ME",
"Milbridge, ME", "Milford, ME", "Millinocket, ME", "Monhegan, ME",
"Monmouth, ME", "Monroe, ME", "Monson, ME", "Monticello, ME",
"Morrill, ME", "Mount Desert, ME", "Mount Vernon, ME", "Naples, ME",
"New Gloucester, ME", "New Harbor, ME", "New Portland, ME",
"New Sharon, ME", "New Sweden, ME", "New Vineyard, ME", "Newcastle, ME",
"Newport, ME", "Newry, ME", "Nobleboro, ME", "Norridgewock, ME",
"North Amity, ME", "North Anson, ME", "North Berwick, ME",
"North Brooklin, ME", "North Fryeburg, ME", "North Haven, ME",
"North Monmouth, ME", "North New Portla, ME", "North Sebago, ME",
"North Shapleigh, ME", "North Turner, ME", "North Vassalboro, ME",
"North Waterboro, ME", "North Waterford, ME", "North Whitefield, ME",
"Norway, ME", "Oakfield, ME", "Oakland, ME", "Ogunquit, ME",
"Old Orchard Beac, ME", "Old Town, ME", "Orland, ME", "Orono, ME",
"Orrington, ME", "Orrs Island, ME", "Owls Head, ME", "Oxbow, ME",
"Oxford, ME", "Palermo, ME", "Palmyra, ME", "Passadumkeag, ME",
"Patten, ME", "Peaks Island, ME", "Pejepscot, ME", "Pemaquid, ME",
"Pembroke, ME", "Penobscot, ME", "Perry, ME", "Peru, ME",
"Phillips, ME", "Phippsburg, ME", "Pittsfield, ME", "Plymouth, ME",
"Poland, ME", "Poland Spring, ME", "Portage, ME", "Porter, ME",
"Portland, ME", "Pownal, ME", "Presque Isle, ME", "Princeton, ME",
"Prospect Harbor, ME", "Randolph, ME", "Rangeley, ME", "Raymond, ME",
"Readfield, ME", "Richmond, ME", "Robbinston, ME", "Rockland, ME",
"Rockport, ME", "Rockwood, ME", "Round Pond, ME", "Roxbury, ME",
"Rumford, ME", "Rumford Center, ME", "Rumford Point, ME",
"Sabattus, ME", "Saco, ME", "Saint Agatha, ME", "Saint Albans, ME",
"Saint David, ME", "Saint Francis, ME", "Saint George, ME",

"Sanford, ME", "Sangerville, ME", "Sargentville, ME", "Scarborough, ME",
 "Searsmont, ME", "Searsport, ME", "Sebago Lake, ME",
 "Sebasco Estates, ME", "Seboeis, ME", "Sedgwick, ME", "Shapleigh, ME",
 "Sherman Mills, ME", "Sherman Station, ME", "Sinclair, ME",
 "Skowhegan, ME", "Small Point, ME", "Smithfield, ME",
 "Smyrna Mills, ME", "Soldier Pond, ME", "Solon, ME", "Sorrento, ME",
 "South Berwick, ME", "South Bristol, ME", "South Casco, ME",
 "South China, ME", "South Gouldsboro, ME", "South Harpswell, ME",
 "South Paris, ME", "South Portland, ME", "South Thomaston, ME",
 "South Waterford, ME", "Southwest Harbor, ME", "Springfield, ME",
 "Springvale, ME", "Spruce Head, ME", "Squirrel Island, ME",
 "Standish, ME", "Steep Falls, ME", "Stetson, ME", "Steuben, ME",
 "Stockholm, ME", "Stockton Springs, ME", "Stonington, ME",
 "Stratton, ME", "Strong, ME", "Sunset, ME", "Surry, ME", "Temple, ME",
 "Tenants Harbor, ME", "Thomaston, ME", "Thorndike, ME", "Topsfield, ME",
 "Trevett, ME", "Troy, ME", "Turner, ME", "Union, ME", "Unity, ME",
 "Van Buren, ME", "Vanceboro, ME", "Vassalboro, ME", "Vinalhaven, ME",
 "Waite, ME", "Waldoboro, ME", "Walpole, ME", "Warren, ME",
 "Washburn, ME", "Washington, ME", "Waterboro, ME", "Wayne, ME",
 "Weeks Mills, ME", "Weld, ME", "Wellington, ME", "Wells, ME",
 "West Baldwin, ME", "West Buxton, ME", "West Forks, ME",
 "West Lebanon, ME", "West Paris, ME", "West Poland, ME",
 "West Rockport, ME", "West Southport, ME", "West Sumner, ME",
 "West Tremont, ME", "Westbrook, ME", "Westfield, ME", "Wilton, ME",
 "Windham, ME", "Windsor, ME", "Winn, ME", "Winslow, ME",
 "Winter Harbor, ME", "Winterport, ME", "Winthrop, ME", "Wiscasset, ME",
 "Woodland, ME", "Woolwich, ME", "Wypitlock, ME", "Yarmouth, ME",
 "York, ME", "Ada, MI", "Addison, MI", "Addison Township, MI",
 "Adrian, MI", "Afton, MI", "Akron, MI", "Alanson, MI", "Albion, MI",
 "Alden, MI", "Alger, MI", "Allegan, MI", "Allen, MI", "Allen Park, MI",
 "Allendale, MI", "Alma, MI", "Almont, MI", "Alpena, MI", "Alto, MI",
 "Anchorville, MI", "Ann Arbor, MI", "Applegate, MI", "Arcadia, MI",
 "Armada, MI", "Ashley, MI", "Athens, MI", "Atlanta, MI",
 "Atlantic Mine, MI", "Attica, MI", "Au Gres, MI", "Au Train, MI",
 "Auburn, MI", "Auburn Hills, MI", "Augusta, MI", "Bad Axe, MI",
 "Bailey, MI", "Baldwin, MI", "Bancroft, MI", "Bangor, MI",
 "Bannister, MI", "Barbeau, MI", "Baroda, MI", "Barryton, MI",
 "Barton City, MI", "Bath, MI", "Battle Creek, MI", "Bay City, MI",
 "Bay Port, MI", "Bay View, MI", "Bear Lake, MI", "Beaver Grove, MI",
 "Beaverton, MI", "Belding, MI", "Bellaire, MI", "Belleville, MI",
 "Bellevue, MI", "Belmont, MI", "Bentley, MI", "Benton Harbor, MI",
 "Benzonia, MI", "Bergland, MI", "Berkley, MI", "Berlin, MI",
 "Berrien Center, MI", "Berrien Springs, MI", "Bessemer, MI",
 "Beulah, MI", "Big Rapids, MI", "Birch Run, MI", "Birmingham, MI",
 "Bitely, MI", "Black River, MI", "Blanchard, MI", "Blissfield, MI",
 "Bloomfield Towns, MI", "Bloomington, MI", "Boon, MI",
 "Boyne City, MI", "Boyne Falls, MI", "Brampton, MI", "Branch, MI",
 "Brant, MI", "Breckenridge, MI", "Brethren, MI", "Bridgeport, MI",
 "Bridgman, MI", "Brighton, MI", "Britton, MI", "Brockway, MI",
 "Bronson, MI", "Brooklyn, MI", "Brown City, MI", "Bruce, MI",
 "Bruce Crossing, MI", "Brutus, MI", "Buchanan, MI", "Buckley, MI",
 "Burlington, MI", "Burr Oak, MI", "Burt, MI", "Byron, MI",
 "Byron Center, MI", "Cadillac, MI", "Camden, MI", "Canton, MI",
 "Carland, MI", "Carleton, MI", "Carney, MI", "Caro, MI",
 "Carp Lake, MI", "Carson City, MI", "Carsonville, MI", "Caseville, MI",

"Casnovia, MI", "Cass City, MI", "Cassopolis, MI", "Cedar, MI",
"Cedar River, MI", "Cedar Springs, MI", "Cedarville, MI",
"Cement City, MI", "Center Line, MI", "Central Lake, MI",
"Centreville, MI", "Ceresco, MI", "Champion, MI", "Channing, MI",
"Charlevoix, MI", "Charlotte, MI", "Chase, MI", "Chassell, MI",
"Cheboygan, MI", "Chelsea, MI", "Chesaning, MI", "Chesterfield, MI",
"Christmas, MI", "Clare, MI", "Clarklake, MI", "Clarksville, MI",
"Clawson, MI", "Clayton, MI", "Clifford, MI", "Climax, MI",
"Clinton, MI", "Clio, MI", "Coldwater, MI", "Coleman, MI", "Coloma, MI",
"Colon, MI", "Columbiaville, MI", "Comins, MI", "Commerce Townshi, MI",
"Comstock Park, MI", "Concord, MI", "Conklin, MI", "Constantine, MI",
"Cooks, MI", "Coopersville, MI", "Copemish, MI", "Coral, MI",
"Cornell, MI", "Corunna, MI", "Cottrellville, MI", "Covert, MI",
"Covington, MI", "Croswell, MI", "Crystal, MI", "Crystal Falls, MI",
"Curran, MI", "Curtis, MI", "Custer, MI", "Dafter, MI", "Daggett, MI",
"Dansville, MI", "Davison, MI", "De Tour Village, MI", "Dearborn, MI",
"Dearborn Heights, MI", "Decatur, MI", "Decker, MI", "Deckerville, MI",
"Deerfield, MI", "Deerton, MI", "Deford, MI", "Delton, MI",
"Detroit, MI", "Detroit Beach, MI", "Dewitt, MI", "Dexter, MI",
"Dimondale, MI", "Dodgeville, MI", "Dorr, MI", "Dowagiac, MI",
"Dowling, MI", "Drummond Island, MI", "Dryden, MI", "Dundee, MI",
"Durand, MI", "Dutton, MI", "Eagle, MI", "Eagle Harbor, MI",
"East Jordan, MI", "East Lansing, MI", "East Leroy, MI",
"East Tawas, MI", "Eastpointe, MI", "Eaton Rapids, MI",
"Eau Claire, MI", "Eben Junction, MI", "Eckerman, MI", "Ecorse, MI",
"Edenville, MI", "Edmore, MI", "Edwardsburg, MI", "Elk Rapids, MI",
"Elkton, MI", "Ellsworth, MI", "Elmira, MI", "Elwell, MI", "Emmett, MI",
"Empire, MI", "Engadine, MI", "Erie, MI", "Escanaba, MI",
"Essexville, MI", "Evart, MI", "Ewen, MI", "Fairgrove, MI",
"Fairview, MI", "Falmouth, MI", "Farmington Hills, MI", "Farwell, MI",
"Fennville, MI", "Fenton, MI", "Fenwick, MI", "Ferndale, MI",
"Fibre, MI", "Fife Lake, MI", "Filion, MI", "Flat Rock, MI",
"Flint, MI", "Flushing, MI", "Foster City, MI", "Fostoria, MI",
"Fountain, MI", "Fowler, MI", "Fowlerville, MI", "Frankenmuth, MI",
"Frankfort, MI", "Franklin, MI", "Fraser, MI", "Frederic, MI",
"Free Soil, MI", "Freeland, MI", "Freeport, MI", "Fremont, MI",
"Fruitport, MI", "Fulton, MI", "Gaastra, MI", "Gagetown, MI",
"Gaines, MI", "Galesburg, MI", "Galien, MI", "Garden, MI",
"Garden City, MI", "Gay, MI", "Gaylord, MI", "Germfask, MI",
"Gibraltar, MI", "Gladwin, MI", "Glen Arbor, MI", "Glennie, MI",
"Gobles, MI", "Goetzville, MI", "Goodrich, MI", "Gould City, MI",
"Gowen, MI", "Grand Beach, MI", "Grand Blanc, MI", "Grand Haven, MI",
"Grand Junction, MI", "Grand Ledge, MI", "Grand Marais, MI",
"Grand Rapids, MI", "Grandville, MI", "Grant, MI", "Grant Township, MI",
"Grass Lake, MI", "Grawn, MI", "Grayling, MI", "Greenbush, MI",
"Greenville, MI", "Greenwood, MI", "Gregory, MI", "Grosse Ile, MI",
"Grosse Pointe, MI", "Gulliver, MI", "Hale, MI", "Hamilton, MI",
"Hamtramck, MI", "Hancock, MI", "Hanover, MI", "Harbor Beach, MI",
"Harbor Point, MI", "Hardwood, MI", "Harper Woods, MI", "Harrietta, MI",
"Harrison, MI", "Harrisville, MI", "Harsens Island, MI", "Hart, MI",
"Hartford, MI", "Hartland, MI", "Haslett, MI", "Hastings, MI",
"Hawks, MI", "Hazel Park, MI", "Hemlock, MI", "Henderson, MI",
"Hermansville, MI", "Herron, MI", "Hersey, MI", "Hesperia, MI",
"Hickory Corners, MI", "Highland, MI", "Highland Park, MI",
"Hillman, MI", "Hillsdale, MI", "Holland, MI", "Holly, MI", "Holt, MI",

"Holton, MI", "Homer, MI", "Honor, MI", "Hope, MI", "Hopkins, MI",
 "Horton, MI", "Houghton, MI", "Houghton Lake, MI", "Howard City, MI",
 "Howell, MI", "Hubbard Lake, MI", "Hubbardston, MI", "Hudson, MI",
 "Hudsonville, MI", "Huntington Woods, MI", "Ida, MI", "Idlewild, MI",
 "Imlay City, MI", "Independence, MI", "Indian River, MI", "Ingalls, MI",
 "Inkster, MI", "Interlochen, MI", "Ionia, MI", "Ira, MI",
 "Iron Mountain, MI", "Iron River, MI", "Irons, MI", "Ironwood, MI",
 "Ithaca, MI", "Jackson, MI", "Jasper, MI", "Jenison, MI", "Jerome, MI",
 "Johannesburg, MI", "Jones, MI", "Jonesville, MI",
 "K I Sawyer A F B, MI", "Kalamazoo, MI", "Kaleva, MI", "Kalkaska, MI",
 "Karlin, MI", "Kawkawlin, MI", "Kent City, MI", "Kenton, MI",
 "Kentwood, MI", "Kewadin, MI", "Keweenaw Bay, MI", "Kimball, MI",
 "Kincheloe, MI", "Kinde, MI", "Kingsley, MI", "Kingston, MI",
 "Kinross, MI", "La Salle, MI", "Lachine, MI", "Laingsburg, MI",
 "Lake, MI", "Lake Ann, MI", "Lake Leelanau, MI", "Lake Odessa, MI",
 "Lakeside, MI", "Lakeview, MI", "Lambertville, MI", "Lanse, MI",
 "Lansing, MI", "Lapeer, MI", "Lathrup Village, MI", "Laurium, MI",
 "Lawrence, MI", "Lawton, MI", "Leland, MI", "Lennon, MI", "Lenox, MI",
 "Leonidas, MI", "Leroy, MI", "Leslie, MI", "Levering, MI",
 "Lewiston, MI", "Lexington, MI", "Limestone, MI", "Lincoln, MI",
 "Lincoln Park, MI", "Linden, MI", "Linwood, MI", "Litchfield, MI",
 "Little Lake, MI", "Livonia, MI", "Long Lake, MI", "Lowell, MI",
 "Ludington, MI", "Luna Pier, MI", "Lupton, MI", "Luther, MI",
 "Luzerne, MI", "Lyons, MI", "Mackinac Island, MI", "Macomb, MI",
 "Madison Heights, MI", "Mancelona, MI", "Manchester, MI",
 "Manitou Beach, MI", "Manton, MI", "Maple City, MI", "Marcellus, MI",
 "Marenisco, MI", "Marion, MI", "Marlette, MI", "Marne, MI",
 "Marshall, MI", "Martin, MI", "Marysville, MI", "Mason, MI",
 "Mass City, MI", "Mattawan, MI", "Maybee, MI", "Mayville, MI",
 "Mc Bain, MI", "Mc Millan, MI", "Mears, MI", "Mecosta, MI",
 "Melvin, MI", "Melvindale, MI", "Mendon, MI", "Menominee, MI",
 "Merrill, MI", "Merritt, MI", "Mesick, MI", "Metamora, MI",
 "Michigamme, MI", "Michigan Center, MI", "Middleton, MI",
 "Middleville, MI", "Midland, MI", "Mikado, MI", "Milan, MI",
 "Milford, MI", "Millersburg, MI", "Millington, MI", "Minden City, MI",
 "Mio, MI", "Montague, MI", "Montgomery, MI", "Montrose, MI",
 "Moorestown, MI", "Moran, MI", "Morenci, MI", "Morley, MI",
 "Morrice, MI", "Mount Clemens, MI", "Mount Morris, MI",
 "Mount Pleasant, MI", "Muir, MI", "Mulliken, MI", "Munger, MI",
 "Munith, MI", "Muskegon, MI", "Muskegon Heights, MI", "Mussey, MI",
 "Nashville, MI", "National City, MI", "Naubinway, MI", "Negaunee, MI",
 "New Boston, MI", "New Era, MI", "New Hudson, MI", "New Lothrop, MI",
 "New Richmond, MI", "Newaygo, MI", "Newberry, MI", "Newport, MI",
 "Niles, MI", "Nisula, MI", "North Adams, MI", "North Branch, MI",
 "North Lake, MI", "North Muskegon, MI", "Northeast, MI",
 "Northport, MI", "Northville, MI", "Northwest, MI", "Norway, MI",
 "Novi, MI", "Nunica, MI", "Oak Park, MI", "Oakland, MI", "Oakley, MI",
 "Okemos, MI", "Olivet, MI", "Omer, MI", "Onaway, MI", "Onekama, MI",
 "Onondaga, MI", "Onsted, MI", "Ontonagon, MI", "Orchard Lake, MI",
 "Orion, MI", "Orleans, MI", "Ortonville, MI", "Oscoda, MI", "Osseo, MI",
 "Ossineke, MI", "Otisville, MI", "Otsego, MI", "Ottawa Lake, MI",
 "Otter Lake, MI", "Ovid, MI", "Owendale, MI", "Owosso, MI",
 "Oxford, MI", "Palms, MI", "Palmyra, MI", "Paradise, MI",
 "Parchment, MI", "Paris, MI", "Parma, MI", "Paw Paw, MI",
 "Pearl Beach, MI", "Peck, MI", "Pelkie, MI", "Pellston, MI",

"Pentwater, MI", "Perrinton, MI", "Perronville, MI", "Perry, MI",
"Petersburg, MI", "Pewamo, MI", "Pickford, MI", "Pierson, MI",
"Pigeon, MI", "Pinckney, MI", "Pinconning, MI", "Pittsford, MI",
"Plainwell, MI", "Pleasant Lake, MI", "Pleasant Ridge, MI",
"Plymouth, MI", "Pointe Aux Pins, MI", "Pontiac, MI", "Port Austin, MI",
"Port Hope, MI", "Port Huron, MI", "Port Sanilac, MI", "Portland, MI",
"Posen, MI", "Potterville, MI", "Powers, MI", "Prescott, MI",
"Princeton, MI", "Prudenville, MI", "Pullman, MI", "Quincy, MI",
"Quinnesec, MI", "Raco, MI", "Rapid City, MI", "Rapid River, MI",
"Ravenna, MI", "Reading, MI", "Redford, MI", "Reed City, MI",
"Reese, MI", "Remus, MI", "Republic, MI", "Rhodes, MI", "Richland, MI",
"Richmond, MI", "Ridgeway, MI", "Riga, MI", "Riley, MI",
"River Rouge, MI", "Riverdale, MI", "Riverview, MI",
"Rives Junction, MI", "Rochester Hills, MI", "Rock, MI", "Rockford, MI",
"Rodney, MI", "Rogers City, MI", "Romulus, MI", "Roscommon, MI",
"Rose City, MI", "Rosebush, MI", "Roseville, MI", "Rothbury, MI",
"Royal Oak, MI", "Ruby, MI", "Rumely, MI", "Ruth, MI", "Saginaw, MI",
"Sagola, MI", "Saint Charles, MI", "Saint Clair, MI",
"Saint Clair Shor, MI", "Saint Helen, MI", "Saint Ignace, MI",
"Saint James, MI", "Saint Johns, MI", "Saint Joseph, MI",
"Saint Louis, MI", "Saline, MI", "Sand Creek, MI", "Sand Lake, MI",
"Sandusky, MI", "Sanford, MI", "Saranac, MI", "Saugatuck, MI",
"Sault Sainte Mar, MI", "Sawyer, MI", "Schoolcraft, MI", "Scotts, MI",
"Scottville, MI", "Sears, MI", "Sebewaing, MI", "Selfridge A N G, MI",
"Seney, MI", "Shelby, MI", "Shelby Township, MI", "Shelbyville, MI",
"Shepherd, MI", "Sheridan, MI", "Sherwood, MI", "Shingleton, MI",
"Sidney, MI", "Silverwood, MI", "Six Lakes, MI", "Skandia, MI",
"Skanee, MI", "Snover, MI", "Sodus, MI", "Somerset, MI",
"South Boardman, MI", "South Branch, MI", "South Haven, MI",
"South Lyon, MI", "South Rockwood, MI", "Southeast, MI",
"Southfield, MI", "Southgate, MI", "Spalding, MI", "Sparta, MI",
"Spring Arbor, MI", "Spring Lake, MI", "Springfield, MI",
"Springport, MI", "Spruce, MI", "Stalwart, MI", "Standish, MI",
"Stanton, MI", "Stanwood, MI", "Stephenson, MI", "Sterling, MI",
"Sterling Heights, MI", "Stevensville, MI", "Stockbridge, MI",
"Stronach, MI", "Sturgis, MI", "Sumner, MI", "Sunfield, MI",
"Suttons Bay, MI", "Swartz Creek, MI", "Sylvan Lake, MI",
"Tawas City, MI", "Taylor, MI", "Tecumseh, MI", "Tekonsha, MI",
"Temperance, MI", "Thompson, MI", "Thompsonville, MI", "Three Oaks, MI",
"Three Rivers, MI", "Tipton, MI", "Toivola, MI", "Tower, MI",
"Traunik, MI", "Traverse City, MI", "Trenary, MI", "Trout Creek, MI",
"Troy, MI", "Trufant, MI", "Turner, MI", "Tustin, MI", "Twin Lake, MI",
"Twining, MI", "Ubly, MI", "Union, MI", "Union City, MI",
"Union Pier, MI", "Unionville, MI", "University Cente, MI",
"Vandalia, MI", "Vanderbilt, MI", "Vassar, MI", "Vermontville, MI",
"Vestaburg, MI", "Vicksburg, MI", "Vulcan, MI", "Wakefield, MI",
"Waldron, MI", "Wales, MI", "Walker, MI", "Walkerville, MI",
"Wallace, MI", "Warren, MI", "Washington, MI", "Waterford, MI",
"Watersmeet, MI", "Watervliet, MI", "Watton, MI", "Wayland, MI",
"Wayne, MI", "Webberville, MI", "Weidman, MI", "Wells, MI",
"Wellston, MI", "West Bloomfield, MI", "West Branch, MI",
"West Olive, MI", "Westland, MI", "Westphalia, MI", "Wetmore, MI",
"Wheeler, MI", "White Cloud, MI", "White Lake, MI", "White Pigeon, MI",
"Whitehall, MI", "Whitmore Lake, MI", "Whittemore, MI",
"Williamsburg, MI", "Williamston, MI", "Willis, MI", "Wilson, MI",

"Wixom, MI", "Wolverine, MI", "Wolverine Lake, MI", "Woodhaven, MI",
"Woodland, MI", "Wyoming, MI", "Ypsilanti, MI", "Zeeland, MI",
"Adams, MN", "Adrian, MN", "Afton, MN", "Aitkin, MN", "Akeley, MN",
"Albany, MN", "Albert Lea, MN", "Alberta, MN", "Albertville, MN",
"Alborn, MN", "Alden, MN", "Aldrich, MN", "Alexandria, MN", "Alpha, MN",
"Altura, MN", "Alvarado, MN", "Amboy, MN", "Amiret, MN",
"Angle Inlet, MN", "Angora, MN", "Angus, MN", "Annandale, MN",
"Apple Valley, MN", "Appleton, MN", "Arco, MN", "Argyle, MN",
"Arlington, MN", "Ashby, MN", "Askov, MN", "Atwater, MN", "Audubon, MN",
"Aurora, MN", "Austin, MN", "Avoca, MN", "Avon, MN", "Babbitt, MN",
"Backus, MN", "Badger, MN", "Bagley, MN", "Baker, MN", "Balaton, MN",
"Barnum, MN", "Barrett, MN", "Barry, MN", "Battle Lake, MN",
"Baudette, MN", "Bayport, MN", "Beardsley, MN", "Beaver Creek, MN",
"Becker, MN", "Bejou, MN", "Belgrade, MN", "Belle Plaine, MN",
"Bellingham, MN", "Beltrami, MN", "Belview, MN", "Bemidji, MN",
"Bena, MN", "Benson, MN", "Beroun, MN", "Bertha, MN", "Big Falls, MN",
"Big Lake, MN", "Bigelow, MN", "Bigfork, MN", "Bingham Lake, MN",
"Birchdale, MN", "Bird Island, MN", "Blackduck, MN", "Blaine, MN",
"Blooming Prairie, MN", "Bloomington, MN", "Blue Earth, MN",
"Bluffton, MN", "Bock, MN", "Borup, MN", "Bovey, MN", "Bowlus, MN",
"Boy River, MN", "Boyd, MN", "Braham, MN", "Brandon, MN",
"Breckenridge, MN", "Brewster, MN", "Bricelyn, MN", "Brimson, MN",
"Britt, MN", "Brooklyn Center, MN", "Brooklyn Park, MN", "Brooks, MN",
"Brookston, MN", "Brooten, MN", "Browerville, MN", "Browns Valley, MN",
"Brownsdale, MN", "Brownsville, MN", "Brownton, MN", "Bruno, MN",
"Buffalo, MN", "Buffalo Lake, MN", "Burnsville, MN", "Burtrum, MN",
"Butterfield, MN", "Buyck, MN", "Byron, MN", "Caledonia, MN",
"Callaway, MN", "Cambridge, MN", "Canby, MN", "Cannon Falls, MN",
"Canton, MN", "Canyon, MN", "Carlisle, MN", "Carlos, MN", "Carlton, MN",
"Carver, MN", "Cass Lake, MN", "Castle Rock, MN",
"Cedar East Bethe, MN", "Centerville, MN", "Ceylon, MN", "Champlin, MN",
"Chandler, MN", "Chanhassen, MN", "Chaska, MN", "Chatfield, MN",
"Chisago City, MN", "Chisholm, MN", "Chokio, MN", "Circle Pines, MN",
"Clara City, MN", "Claremont, MN", "Clarissa, MN", "Clarkfield, MN",
"Clarks Grove, MN", "Clear Lake, MN", "Clearbrook, MN",
"Clearwater, MN", "Clements, MN", "Cleveland, MN", "Clinton, MN",
"Clitherall, MN", "Clontarf, MN", "Cloquet, MN", "Cohasset, MN",
"Cokato, MN", "Cold Spring, MN", "Cologne, MN", "Columbia Heights, MN",
"Comfrey, MN", "Comstock, MN", "Conger, MN", "Cook, MN",
"Coon Rapids, MN", "Correll, MN", "Cosmos, MN", "Cottage Grove, MN",
"Cottonwood, MN", "Courtland, MN", "Crane Lake, MN", "Cromwell, MN",
"Crookston, MN", "Crosby, MN", "Crosslake, MN", "Crystal, MN",
"Culver, MN", "Currie, MN", "Cushing, MN", "Cyrus, MN", "Dakota, MN",
"Dalbo, MN", "Dalton, MN", "Danube, MN", "Danvers, MN", "Darfur, MN",
"Darwin, MN", "Dassel, MN", "Dawson, MN", "Dayton, MN", "De Graff, MN",
"Deer Creek, MN", "Deer River, MN", "Deerwood, MN", "Delano, MN",
"Delavan, MN", "Delft, MN", "Delhi, MN", "Dent, MN",
"Detroit Lakes, MN", "Dexter, MN", "Dilworth, MN", "Dodge Center, MN",
"Donaldson, MN", "Donnelly, MN", "Doran, MN", "Dover, MN", "Dovray, MN",
"Downer, MN", "Duluth, MN", "Dumont, MN", "Dundas, MN", "Dundee, MN",
"Dunnell, MN", "Eagan, MN", "Eagle Bend, MN", "Eagle Lake, MN",
"East Bethel, MN", "East Grand Forks, MN", "East Gull Lake, MN",
"Easton, MN", "Echo, MN", "Eden Prairie, MN", "Eden Valley, MN",
"Edgerton, MN", "Edina, MN", "Effie, MN", "Elbow Lake, MN",
"Eldred, MN", "Elgin, MN", "Elizabeth, MN", "Elk River, MN", "Elko, MN",

"Elkton, MN", "Ellendale, MN", "Ellsworth, MN", "Elmore, MN", "Ely, MN",
 "Elysian, MN", "Embarrass, MN", "Emily, MN", "Emmons, MN", "Erhard, MN",
 "Erskine, MN", "Esko, MN", "Essig, MN", "Euclid, MN", "Evan, MN",
 "Evansville, MN", "Eveleth, MN", "Excelsior, MN", "Fairfax, MN",
 "Fairmont, MN", "Faribault, MN", "Farmington, MN", "Farwell, MN",
 "Federal Dam, MN", "Felton, MN", "Fertile, MN", "Fifty Lakes, MN",
 "Finland, MN", "Finlayson, MN", "Fisher, MN", "Flensburg, MN",
 "Floodwood, MN", "Florence, MN", "Foley, MN", "Forest Lake, MN",
 "Foreston, MN", "Fort Ripley, MN", "Fort Snelling, MN", "Fosston, MN",
 "Fountain, MN", "Foxhome, MN", "Franklin, MN", "Frazee, MN",
 "Freeborn, MN", "Freeport, MN", "Fridley, MN", "Frontenac, MN",
 "Frost, MN", "Fulda, MN", "Garden City, MN", "Garfield, MN",
 "Garrison, MN", "Garvin, MN", "Gary, MN", "Gatzke, MN", "Gaylord, MN",
 "Geneva, MN", "Georgetown, MN", "Gheen, MN", "Ghent, MN", "Gibbon, MN",
 "Gilbert, MN", "Gilman, MN", "Glencoe, MN", "Glenville, MN",
 "Glenwood, MN", "Glyndon, MN", "Golden Valley, MN", "Gonvick, MN",
 "Good Thunder, MN", "Goodhue, MN", "Goodland, MN", "Goodridge, MN",
 "Goodview, MN", "Graceville, MN", "Granada, MN", "Grand Marais, MN",
 "Grand Meadow, MN", "Grand Portage, MN", "Granger, MN",
 "Granite Falls, MN", "Grasston, MN", "Green Isle, MN", "Greenbush, MN",
 "Grey Eagle, MN", "Grove City, MN", "Grygla, MN", "Gully, MN",
 "Hackensack, MN", "Hadley, MN", "Hallock, MN", "Halma, MN",
 "Halstad, MN", "Ham Lake, MN", "Hamburg, MN", "Hamel, MN",
 "Hammond, MN", "Hampton, MN", "Hancock, MN", "Hanley Falls, MN",
 "Hanska, MN", "Hardwick, MN", "Harmony, MN", "Harris, MN",
 "Hartland, MN", "Hatfield, MN", "Hawick, MN", "Hayfield, MN",
 "Hayward, MN", "Hazel Run, MN", "Hector, MN", "Henderson, MN",
 "Hendricks, MN", "Hendrum, MN", "Henning, MN", "Herman, MN",
 "Hermantown, MN", "Heron Lake, MN", "Hewitt, MN", "Hibbing, MN",
 "Hill City, MN", "Hillman, MN", "Hills, MN", "Hinckley, MN",
 "Hines, MN", "Hitterdal, MN", "Hoffman, MN", "Hokah, MN",
 "Holdingford, MN", "Holland, MN", "Hollandale, MN", "Holloway, MN",
 "Holmes City, MN", "Holyoke, MN", "Hope, MN", "Houston, MN",
 "Hovland, MN", "Howard Lake, MN", "Hoyt Lakes, MN", "Huntley, MN",
 "Hutchinson, MN", "International Fa, MN", "Inver Grove Heig, MN",
 "Iona, MN", "Iron, MN", "Ironton, MN", "Isabella, MN", "Isanti, MN",
 "Isle, MN", "Ivanhoe, MN", "Jackson, MN", "Jacobson, MN",
 "Janesville, MN", "Jasper, MN", "Jeffers, MN", "Jenkins, MN",
 "Johnson, MN", "Jordan, MN", "Kanaranzi, MN", "Kandiyohi, MN",
 "Karlstad, MN", "Kasota, MN", "Kasson, MN", "Kelliher, MN",
 "Kelsey, MN", "Kennedy, MN", "Kenneth, MN", "Kensington, MN",
 "Kent, MN", "Kenyon, MN", "Kerkhoven, MN", "Kerrick, MN",
 "Kettle River, MN", "Kiestler, MN", "Kilkenny, MN", "Kimball, MN",
 "La Crescent, MN", "La Prairie, MN", "Lafayette, MN", "Lake Benton, MN",
 "Lake Bronson, MN", "Lake City, MN", "Lake Crystal, MN",
 "Lake Elmo, MN", "Lake George, MN", "Lake Itasca, MN",
 "Lake Lillian, MN", "Lake Park, MN", "Lake Shore, MN",
 "Lake Wilson, MN", "Lakefield, MN", "Lakeland, MN", "Lakeville, MN",
 "Lamberton, MN", "Lanesboro, MN", "Laporte, MN", "Lauderdale, MN",
 "Le Center, MN", "Le Roy, MN", "Le Sueur, MN", "Leader, MN",
 "Lengby, MN", "Leonard, MN", "Leota, MN", "Lester Prairie, MN",
 "Lewiston, MN", "Lewisville, MN", "Lindstrom, MN", "Lismore, MN",
 "Litchfield, MN", "Little Canada, MN", "Little Falls, MN",
 "Little Marais, MN", "Little Sauk, MN", "Littlefork, MN",
 "Lockhart, MN", "Loman, MN", "London, MN", "Long Lake, MN",

"Longville, MN", "Loretto, MN", "Louisburg, MN", "Lowry, MN",
 "Lucan, MN", "Lutsen, MN", "Luverne, MN", "Lyle, MN", "Lynd, MN",
 "Mabel, MN", "Madelia, MN", "Madison, MN", "Madison Lake, MN",
 "Magnolia, MN", "Mahnomen, MN", "Mahtowa, MN", "Makinen, MN",
 "Manchester, MN", "Manhattan Beach, MN", "Mankato, MN",
 "Mantorville, MN", "Maple Grove, MN", "Maple Lake, MN",
 "Maple Plain, MN", "Mapleton, MN", "Maplewood, MN", "Marcell, MN",
 "Marietta, MN", "Marine On Saint, MN", "Markville, MN", "Marshall, MN",
 "Max, MN", "Mayer, MN", "Maynard, MN", "Mazeppa, MN", "Mc Grath, MN",
 "Mc Gregor, MN", "Mcintosh, MN", "Meadowlands, MN", "Medford, MN",
 "Melrose, MN", "Melrude, MN", "Menahga, MN", "Mendota, MN",
 "Mentor, MN", "Meriden, MN", "Merrifield, MN", "Middle River, MN",
 "Milaca, MN", "Milan, MN", "Millville, MN", "Milroy, MN", "Miltona, MN",
 "Minneapolis, MN", "Minneota, MN", "Minnesota City, MN",
 "Minnesota Lake, MN", "Minnetonka, MN", "Mizpah, MN", "Montevideo, MN",
 "Montgomery, MN", "Monticello, MN", "Montrose, MN", "Moorhead, MN",
 "Moose Lake, MN", "Mora, MN", "Morgan, MN", "Morris, MN",
 "Morristown, MN", "Morton, MN", "Mound, MN", "Mountain Iron, MN",
 "Mountain Lake, MN", "Murdock, MN", "Nashua, MN", "Nashwauk, MN",
 "Nassau, MN", "Naytahwaush, MN", "Nelson, MN", "Nerstrand, MN",
 "Nevis, MN", "New Auburn, MN", "New Brighton, MN", "New Germany, MN",
 "New London, MN", "New Prague, MN", "New Richland, MN", "New Ulm, MN",
 "New York Mills, MN", "Newfolden, MN", "Newport, MN", "Nicollet, MN",
 "Nielsville, MN", "Norcross, MN", "North Branch, MN",
 "North Mankato, MN", "North Saint Paul, MN", "Northfield, MN",
 "Northome, MN", "Northrop, MN", "Norwood, MN", "Noyes, MN",
 "Oak Island, MN", "Oak Park, MN", "Oak Park Heights, MN", "Oakdale, MN",
 "Oakland, MN", "Odessa, MN", "Odin, MN", "Ogema, MN", "Ogilvie, MN",
 "Okabena, MN", "Oklee, MN", "Olivia, MN", "Onamia, MN", "Orleans, MN",
 "Ormsby, MN", "Oronoco, MN", "Ortonville, MN", "Osage, MN",
 "Osakis, MN", "Oslo, MN", "Ostrander, MN", "Otisco, MN",
 "Ottertail, MN", "Outing, MN", "Owatonna, MN", "Palisade, MN",
 "Park Rapids, MN", "Parkers Prairie, MN", "Parkville, MN",
 "Paynesville, MN", "Pease, MN", "Pelican Rapids, MN", "Pemberton, MN",
 "Pencer, MN", "Pengilly, MN", "Pennington, MN", "Pennock, MN",
 "Pequot Lakes, MN", "Perham, MN", "Perley, MN", "Peterson, MN",
 "Pierz, MN", "Pillager, MN", "Pine Island, MN", "Pine River, MN",
 "Pitt, MN", "Plainview, MN", "Plato, MN", "Plummer, MN", "Plymouth, MN",
 "Ponemah, MN", "Ponsford, MN", "Porter, MN", "Preston, MN",
 "Princeton, MN", "Prinsburg, MN", "Prior Lake, MN", "Proctor, MN",
 "Puposky, MN", "Quamba, MN", "Racine, MN", "Radium, MN", "Ramsey, MN",
 "Randall, MN", "Randolph, MN", "Ranier, MN", "Ray, MN", "Raymond, MN",
 "Reading, MN", "Red Lake Falls, MN", "Red Wing, MN", "Redby, MN",
 "Redlake, MN", "Remer, MN", "Renville, MN", "Revere, MN", "Rice, MN",
 "Richfield, MN", "Richmond, MN", "Richville, MN", "Richwood, MN",
 "Robbinsdale, MN", "Rochert, MN", "Rochester, MN", "Rock Creek, MN",
 "Rockford, MN", "Rogers, MN", "Rollag, MN", "Rollingstone, MN",
 "Roosevelt, MN", "Rose Creek, MN", "Rosemount, MN", "Roseville, MN",
 "Rothsay, MN", "Round Lake, MN", "Royalton, MN", "Rush City, MN",
 "Rushford, MN", "Rushmore, MN", "Russell, MN", "Sabin, MN",
 "Sacred Heart, MN", "Saginaw, MN", "Saint Charles, MN",
 "Saint Clair, MN", "Saint Cloud, MN", "Saint Francis, MN",
 "Saint Hilaire, MN", "Saint James, MN", "Saint Joseph, MN",
 "Saint Leo, MN", "Saint Louis Park, MN", "Saint Michael, MN",
 "Saint Paul, MN", "Saint Paul Park, MN", "Saint Peter, MN",

"Saint Stephen, MN", "Saint Vincent, MN", "Salol, MN", "Sanborn, MN",
 "Sargeant, MN", "Sartell, MN", "Sauk Centre, MN", "Sauk Rapids, MN",
 "Saum, MN", "Savage, MN", "Sawyer, MN", "Scandia, MN", "Schroeder, MN",
 "Seaforth, MN", "Sebek, MN", "Sedan, MN", "Shafer, MN", "Shakopee, MN",
 "Shelly, MN", "Sherburn, MN", "Shevlin, MN", "Shoreview, MN",
 "Silver Creek, MN", "Silver Lake, MN", "Slayton, MN", "Sleepy Eye, MN",
 "Solway, MN", "South Haven, MN", "South Saint Paul, MN", "Spicer, MN",
 "Spring Grove, MN", "Spring Lake, MN", "Spring Park, MN",
 "Spring Valley, MN", "Springfield, MN", "Squaw Lake, MN", "Stacy, MN",
 "Stanchfield, MN", "Stanton, MN", "Staples, MN", "Starbuck, MN",
 "Steen, MN", "Stephen, MN", "Stewart, MN", "Stewartville, MN",
 "Storden, MN", "Strandquist, MN", "Strathcona, MN", "Sturgeon Lake, MN",
 "Sunburg, MN", "Svea, MN", "Swan River, MN", "Swanville, MN",
 "Swatara, MN", "Swift, MN", "Talmoon, MN", "Tamarack, MN", "Taopi, MN",
 "Taunton, MN", "Taylors Falls, MN", "Tenney, MN", "Tenstrike, MN",
 "Theilman, MN", "Thief River Fall, MN", "Tofte, MN", "Togo, MN",
 "Tower, MN", "Tracy, MN", "Trail, MN", "Trimont, MN", "Truman, MN",
 "Twin Lakes, MN", "Twin Valley, MN", "Two Harbors, MN", "Tyler, MN",
 "Ulen, MN", "Underwood, MN", "Upsala, MN", "Utica, MN",
 "Vadnais Heights, MN", "Verdi, MN", "Vergas, MN", "Verndale, MN",
 "Vernon Center, MN", "Veseli, MN", "Vesta, MN", "Victoria, MN",
 "Viking, MN", "Villard, MN", "Vining, MN", "Viola, MN", "Virginia, MN",
 "Wabasha, MN", "Wabasso, MN", "Waconia, MN", "Wadena, MN", "Wahkon, MN",
 "Waite Park, MN", "Waldorf, MN", "Walker, MN", "Walnut Grove, MN",
 "Walters, MN", "Waltham, MN", "Wanamingo, MN", "Wanda, MN",
 "Wannaska, MN", "Warba, MN", "Warroad, MN", "Warsaw, MN", "Waseca, MN",
 "Waskish, MN", "Watertown, MN", "Waterville, MN", "Watkins, MN",
 "Watson, MN", "Waubun, MN", "Waverly, MN", "Wayzata, MN", "Webster, MN",
 "Welch, MN", "Welcome, MN", "Wells, MN", "Wendell, MN",
 "West Concord, MN", "West Saint Paul, MN", "West Union, MN",
 "Westbrook, MN", "Whalan, MN", "Wheaton, MN", "Whipholt, MN",
 "White Bear Lake, MN", "Wilder, MN", "Williams, MN", "Willmar, MN",
 "Willow River, MN", "Wilmont, MN", "Wilton, MN", "Winger, MN",
 "Winnebago, MN", "Winsted, MN", "Winthrop, MN", "Wirt, MN",
 "Wolf Lake, MN", "Wolverton, MN", "Wood Lake, MN", "Woodbury, MN",
 "Woodstock, MN", "Worthington, MN", "Wrenshall, MN", "Wright, MN",
 "Wykoff, MN", "Young America, MN", "Zim, MN", "Zimmerman, MN",
 "Zumbrota, MN", "Adrian, MO", "Advance, MO", "Affton, MO", "Agency, MO",
 "Albany, MO", "Aldrich, MO", "Alexandria, MO", "Alma, MO",
 "Altamont, MO", "Amazonia, MO", "Amity, MO", "Amoret, MO",
 "Amsterdam, MO", "Anabel, MO", "Anderson, MO", "Annada, MO",
 "Annapolis, MO", "Antonia, MO", "Anutt, MO", "Appleton City, MO",
 "Arab, MO", "Arbela, MO", "Arbyrd, MO", "Arcadia, MO", "Archie, MO",
 "Arcola, MO", "Argyle, MO", "Armstrong, MO", "Arnold, MO", "Asbury, MO",
 "Ash Grove, MO", "Ashburn, MO", "Ashland, MO", "Atlanta, MO",
 "Augusta, MO", "Auxvasse, MO", "Ava, MO", "Avalon, MO", "Avilla, MO",
 "Bakersfield, MO", "Ballwin, MO", "Baring, MO", "Barnard, MO",
 "Barnett, MO", "Barnhart, MO", "Bates City, MO", "Bay, MO",
 "Beaufort, MO", "Belgrade, MO", "Bell City, MO", "Belle, MO",
 "Belleview, MO", "Bellflower, MO", "Belton, MO", "Bendavis, MO",
 "Benton, MO", "Benton City, MO", "Berger, MO", "Berkeley, MO",
 "Bernie, MO", "Berryman, MO", "Bertrand, MO", "Bethany, MO",
 "Bethel, MO", "Beulah, MO", "Bevier, MO", "Bigelow, MO", "Billings, MO",
 "Birch Tree, MO", "Bixby, MO", "Black, MO", "Blackburn, MO",
 "Blackwater, MO", "Blackwell, MO", "Blairstown, MO", "Bland, MO",

"Bloomfield, MO", "Bloomsdale, MO", "Blue Eye, MO", "Blue Springs, MO",
"Blythedale, MO", "Bogard, MO", "Bois D Arc, MO", "Bolckow, MO",
"Bolivar, MO", "Bonne Terre, MO", "Bonnots Mill, MO", "Boonville, MO",
"Boss, MO", "Bosworth, MO", "Bourbon, MO", "Bowling Green, MO",
"Bradleyville, MO", "Bragg City, MO", "Brandsville, MO",
"Branson West, MO", "Brashear, MO", "Braymer, MO", "Breckenridge, MO",
"Brentwood, MO", "Briar, MO", "Bridgeton, MO", "Brighton, MO",
"Brinktown, MO", "Brixey, MO", "Bronaugh, MO", "Brookfield, MO",
"Brookline Statio, MO", "Broseley, MO", "Browning, MO", "Brumley, MO",
"Bruner, MO", "Brunswick, MO", "Bucklin, MO", "Buckner, MO",
"Bucyrus, MO", "Buffalo, MO", "Bunceton, MO", "Bunker, MO",
"Burfordville, MO", "Burlington Junct, MO", "Butler, MO",
"Butterfield, MO", "Cabool, MO", "Cadet, MO", "Cainsville, MO",
"Cairo, MO", "Caledonia, MO", "Calhoun, MO", "California, MO",
"Callao, MO", "Camden, MO", "Camden Point, MO", "Camdenton, MO",
"Cameron, MO", "Campbell, MO", "Canton, MO", "Cape Fair, MO",
"Cape Girardeau, MO", "Cardwell, MO", "Carl Junction, MO",
"Carrollton, MO", "Carterville, MO", "Carthage, MO",
"Caruthersville, MO", "Cassville, MO", "Catawissa, MO", "Catron, MO",
"Caulfield, MO", "Cedar Hill, MO", "Cedarcreek, MO", "Center, MO",
"Centertown, MO", "Centerview, MO", "Centerville, MO", "Centralia, MO",
"Chadwick, MO", "Chaffee, MO", "Chamois, MO", "Charleston, MO",
"Cherryville, MO", "Chesterfield, MO", "Chestnutridge, MO",
"Chilhowee, MO", "Chillicothe, MO", "Chula, MO", "Clarence, MO",
"Clark, MO", "Clarksburg, MO", "Clarksdale, MO", "Clarksville, MO",
"Clarkton, MO", "Clayton, MO", "Clearmont, MO", "Cleveland, MO",
"Clever, MO", "Clifton Hill, MO", "Climax Springs, MO", "Clubb, MO",
"Clyde, MO", "Coatsville, MO", "Coffey, MO", "Cole Camp, MO",
"Collins, MO", "Columbia, MO", "Conception, MO", "Conception Junct, MO",
"Concordia, MO", "Conway, MO", "Cook Station, MO", "Corder, MO",
"Corning, MO", "Cosby, MO", "Couch, MO", "Cowgill, MO", "Crane, MO",
"Creighton, MO", "Crescent, MO", "Creve Coeur, MO", "Crocker, MO",
"Cross Timbers, MO", "Crystal City, MO", "Cuba, MO", "Curryville, MO",
"Dadeville, MO", "Daisy, MO", "Dalton, MO", "Darlington, MO",
"Davisville, MO", "Dawn, MO", "De Kalb, MO", "De Soto, MO",
"De Witt, MO", "Dearborn, MO", "Deepwater, MO", "Deerfield, MO",
"Defiance, MO", "Delta, MO", "Denver, MO", "Des Arc, MO",
"Des Peres, MO", "Desloge, MO", "Devils Elbow, MO", "Dexter, MO",
"Diamond, MO", "Dittmer, MO", "Dixon, MO", "Doe Run, MO", "Dora, MO",
"Dover, MO", "Downing, MO", "Drexel, MO", "Drury, MO", "Dudley, MO",
"Dugginsville, MO", "Duke, MO", "Dunnegan, MO", "Durham, MO",
"Eagle Rock, MO", "Eagleville, MO", "East Prairie, MO", "Easton, MO",
"Edgar Springs, MO", "Edgerton, MO", "Edina, MO", "Edwards, MO",
"El Dorado Spring, MO", "Eldon, MO", "Eldridge, MO", "Elk Creek, MO",
"Elkland, MO", "Ellington, MO", "Ellsinore, MO", "Elmer, MO",
"Elmo, MO", "Elsberry, MO", "Emden, MO", "Eminence, MO", "Eolia, MO",
"Essex, MO", "Ethel, MO", "Eugene, MO", "Eunice, MO", "Everton, MO",
"Ewing, MO", "Excello, MO", "Excelsior Spring, MO", "Exeter, MO",
"Fair Grove, MO", "Fair Play, MO", "Fairdealing, MO", "Fairfax, MO",
"Fairview, MO", "Falcon, MO", "Farber, MO", "Farmington, MO",
"Faucett, MO", "Fayette, MO", "Fenton, MO", "Ferguson, MO",
"Ferrelview, MO", "Festus, MO", "Fillmore, MO", "Fisk, MO",
"Flemington, MO", "Fletcher, MO", "Florence, MO", "Florissant, MO",
"Foley, MO", "Fordland, MO", "Forest City, MO", "Foristell, MO",
"Forsyth, MO", "Fort Leonard Woo, MO", "Fortescue, MO", "Fortuna, MO",

"Foster, MO", "Four Seasons, MO", "Frankford, MO", "Franklin, MO",
 "Freeburg, MO", "Freeman, MO", "Freistatt, MO", "Fremont, MO",
 "French Village, MO", "Friedheim, MO", "Frohna, MO", "Fulton, MO",
 "Gainesville, MO", "Galena, MO", "Gallatin, MO", "Galt, MO",
 "Garden City, MO", "Garrison, MO", "Gatewood, MO", "Gentry, MO",
 "Gerald, MO", "Gibbs, MO", "Gideon, MO", "Gilliam, MO",
 "Gilman City, MO", "Gipsy, MO", "Gladstone, MO", "Glasgow, MO",
 "Glenallen, MO", "Glencoe, MO", "Glenwood, MO", "Gobler, MO",
 "Golden, MO", "Golden City, MO", "Goodman, MO", "Goodson, MO",
 "Gorin, MO", "Gower, MO", "Graff, MO", "Graham, MO", "Grain Valley, MO",
 "Granby, MO", "Grand Pass, MO", "Grandin, MO", "Grandview, MO",
 "Grant City, MO", "Grassy, MO", "Gravois Mills, MO", "Gray Summit, MO",
 "Green Castle, MO", "Green City, MO", "Green Ridge, MO",
 "Greenfield, MO", "Greentop, MO", "Greenville, MO", "Grover, MO",
 "Grovespring, MO", "Guilford, MO", "Hale, MO", "Half Way, MO",
 "Hallsville, MO", "Hamilton, MO", "Hannibal, MO", "Hardenville, MO",
 "Hardin, MO", "Harris, MO", "Harrisburg, MO", "Harrisonville, MO",
 "Hartsburg, MO", "Hartshorn, MO", "Hartville, MO", "Harviell, MO",
 "Harwood, MO", "Hatfield, MO", "Hawk Point, MO", "Hayti Heights, MO",
 "Hazelwood, MO", "Helena, MO", "Hemple, MO", "Henley, MO",
 "Henrietta, MO", "Herculaneum, MO", "Hermitage, MO", "Higbee, MO",
 "Higginsville, MO", "High Hill, MO", "High Point, MO", "High Ridge, MO",
 "Highlandville, MO", "Hillsboro, MO", "Hiram, MO", "Holcomb, MO",
 "Holden, MO", "Holliday, MO", "Hollister, MO", "Holt, MO",
 "Holts Summit, MO", "Homestown, MO", "Hopkins, MO", "Hornersville, MO",
 "Horton, MO", "House Springs, MO", "Houston, MO", "Houstonia, MO",
 "Huggins, MO", "Hughesville, MO", "Humansville, MO", "Humphreys, MO",
 "Hunnewell, MO", "Huntsville, MO", "Hurdland, MO", "Hurley, MO",
 "Iantha, MO", "Iberia, MO", "Independence, MO", "Ionia, MO",
 "Iron Mountain, MO", "Irondale, MO", "Isabella, MO", "Jackson, MO",
 "Jacksonville, MO", "Jadwin, MO", "Jameson, MO", "Jamesport, MO",
 "Jamestown, MO", "Jane, MO", "Jasper, MO", "Jefferson City, MO",
 "Jenkins, MO", "Jennings, MO", "Jerico Springs, MO", "Jerome, MO",
 "Jonesburg, MO", "Joplin, MO", "Kahoka, MO", "Kaiser, MO",
 "Kansas City, MO", "Kearney, MO", "Kennett, MO", "Keytesville, MO",
 "Kidder, MO", "Kimberling City, MO", "King City, MO",
 "Kingdom City, MO", "Kingston, MO", "Kingsville, MO", "Kirbyville, MO",
 "Kirksville, MO", "Kirkwood, MO", "Kissee Mills, MO", "Knob Noster, MO",
 "Knox City, MO", "Koeltztown, MO", "Koshkonong, MO", "La Belle, MO",
 "La Grange, MO", "La Monte, MO", "La Plata, MO", "La Russell, MO",
 "Labadie, MO", "Laclede, MO", "Laddonia, MO", "Ladue, MO",
 "Lake Forest Esta, MO", "Lake Lotawana, MO", "Lake Saint Louis, MO",
 "Lake Sherwood, MO", "Lake Tapawingo, MO", "Lake Waukomis, MO",
 "Lake Winnebago, MO", "Lampe, MO", "Lanagan, MO", "Lancaster, MO",
 "Laquey, MO", "Laredo, MO", "Latham, MO", "Lathrop, MO", "Latour, MO",
 "Lawson, MO", "Leadwood, MO", "Leasburg, MO", "Lebanon, MO",
 "Lees Summit, MO", "Leeton, MO", "Lemay, MO", "Lenox, MO",
 "Lentner, MO", "Leonard, MO", "Leopold, MO", "Leslie, MO",
 "Lesterville, MO", "Lewistown, MO", "Lexington, MO", "Liberal, MO",
 "Licking, MO", "Lilbourn, MO", "Lincoln, MO", "Linn, MO",
 "Linn Creek, MO", "Linneus, MO", "Livonia, MO", "Lock Springs, MO",
 "Lockwood, MO", "Lodi, MO", "Lohman, MO", "Lone Jack, MO",
 "Lonedell, MO", "Long Lane, MO", "Loose Creek, MO", "Louisburg, MO",
 "Louisiana, MO", "Lowndes, MO", "Lowry City, MO", "Lucerne, MO",
 "Ludlow, MO", "Luebbering, MO", "Luray, MO", "Lynchburg, MO",

"Macks Creek, MO", "Macomb, MO", "Macon, MO", "Madison, MO",
"Maitland, MO", "Malden, MO", "Manchester, MO", "Mansfield, MO",
"Maplewood, MO", "Marceline, MO", "Marionville, MO", "Marquand, MO",
"Marshfield, MO", "Marston, MO", "Martin City, MO", "Martinsburg, MO",
"Martinsville, MO", "Marvel Cave Park, MO", "Maryland Heights, MO",
"Maryville, MO", "Matthews, MO", "Maysville, MO", "Mayview, MO",
"Maywood, MO", "Mc Clurg, MO", "Mc Fall, MO", "Mc Gee, MO",
"Meadville, MO", "Memphis, MO", "Mendon, MO", "Mercer, MO", "Meta, MO",
"Mexico, MO", "Miami, MO", "Middle Brook, MO", "Middletown, MO",
"Milan, MO", "Mill Spring, MO", "Millcreek, MO", "Miller, MO",
"Millersville, MO", "Milo, MO", "Mindenmines, MO", "Mineral Point, MO",
"Moberly, MO", "Mokane, MO", "Monett, MO", "Monroe City, MO",
"Montgomery City, MO", "Monticello, MO", "Montreal, MO", "Montrose, MO",
"Moody, MO", "Mooresville, MO", "Mora, MO", "Morehouse, MO",
"Morrison, MO", "Morrisville, MO", "Moscow Mills, MO", "Mound City, MO",
"Moundville, MO", "Mount Moriah, MO", "Mount Sterling, MO",
"Mount Vernon, MO", "Mountain Grove, MO", "Mountain View, MO",
"Myrtle, MO", "Napoleon, MO", "Napton, MO", "Naylor, MO",
"Neelyville, MO", "Nelson, MO", "Neosho, MO", "Nevada, MO",
"New Bloomfield, MO", "New Boston, MO", "New Cambria, MO",
"New Florence, MO", "New Franklin, MO", "New Hampton, MO",
"New Hartford, MO", "New Haven, MO", "New London, MO", "New Madrid, MO",
"New Wells, MO", "Newark, MO", "Newburg, MO", "Newtown, MO",
"Niangua, MO", "Nixa, MO", "Noble, MO", "Noel, MO", "Norborne, MO",
"Normandy, MO", "North County, MO", "North Kansas Cit, MO",
"Norwood, MO", "Nottingham, MO", "Novelty, MO", "Novinger, MO",
"Oak Grove, MO", "Oak Ridge, MO", "Odessa, MO", "Old Appleton, MO",
"Old Monroe, MO", "Oldfield, MO", "Olean, MO", "Olivette, MO",
"Olney, MO", "Oran, MO", "Oregon, MO", "Oronogo, MO", "Orrick, MO",
"Osage Beach, MO", "Osborn, MO", "Osceola, MO", "Otterville, MO",
"Overland, MO", "Owensville, MO", "Oxly, MO", "Ozark, MO",
"Pacific, MO", "Palmyra, MO", "Paris, MO", "Parkville, MO", "Parma, MO",
"Parnell, MO", "Patterson, MO", "Patton, MO", "Pattonsburg, MO",
"Paynesville, MO", "Peace Valley, MO", "Peculiar, MO", "Perry, MO",
"Perryville, MO", "Pevely, MO", "Philadelphia, MO", "Phillipsburg, MO",
"Pickering, MO", "Piedmont, MO", "Pierce City, MO", "Pilot Grove, MO",
"Pittsburg, MO", "Plato, MO", "Platte City, MO", "Plattsburg, MO",
"Pleasant Hill, MO", "Pleasant Hope, MO", "Pleasant Valley, MO",
"Plevna, MO", "Polk, MO", "Pollock, MO", "Polo, MO", "Pomona, MO",
"Ponce De Leon, MO", "Pontiac, MO", "Poplar Bluff, MO",
"Portage Des Siou, MO", "Portageville, MO", "Portland, MO",
"Potosi, MO", "Pottersville, MO", "Powell, MO", "Powersite, MO",
"Powersville, MO", "Poynor, MO", "Prairie Home, MO", "Preston, MO",
"Princeton, MO", "Protem, MO", "Purdin, MO", "Purdy, MO", "Puxico, MO",
"Queen City, MO", "Quincy, MO", "Quitman, MO", "Qulin, MO",
"Randolph, MO", "Ravenwood, MO", "Raymondville, MO", "Raymore, MO",
"Raytown, MO", "Rayville, MO", "Rea, MO", "Redford, MO", "Reeds, MO",
"Republic, MO", "Rhineland, MO", "Rich Hill, MO", "Richards, MO",
"Richland, MO", "Richmond, MO", "Richmond Heights, MO", "Richwoods, MO",
"Ridgedale, MO", "Ridgeway, MO", "Riverton, MO", "Roach, MO",
"Robertsville, MO", "Roby, MO", "Rocheport, MO", "Rock Port, MO",
"Rockaway Beach, MO", "Rockville, MO", "Rocky Comfort, MO",
"Rocky Mount, MO", "Rogersville, MO", "Rolla, MO", "Rosebud, MO",
"Rosendale, MO", "Rothville, MO", "Rueter, MO", "Rush Hill, MO",
"Rushville, MO", "Russellville, MO", "Rutledge, MO", "Saint Ann, MO",

"Saint Catharine, MO", "Saint Charles, MO", "Saint Clair, MO",
 "Saint Elizabeth, MO", "Saint James, MO", "Saint Joseph, MO",
 "Saint Louis, MO", "Saint Mary, MO", "Saint Patrick, MO",
 "Saint Paul, MO", "Saint Peters, MO", "Saint Robert, MO",
 "Saint Thomas, MO", "Salem, MO", "Salisbury, MO", "Santa Fe, MO",
 "Sappington, MO", "Sarcoxie, MO", "Savannah, MO", "Schell City, MO",
 "Scopus, MO", "Scott City, MO", "Sedalia, MO", "Sedgewickville, MO",
 "Seligman, MO", "Senath, MO", "Seneca, MO", "Seymour, MO",
 "Shelbina, MO", "Shelbyville, MO", "Sheldon, MO", "Shell Knob, MO",
 "Sheridan, MO", "Shook, MO", "Sibley, MO", "Sikeston, MO", "Silex, MO",
 "Silva, MO", "Skidmore, MO", "Slater, MO", "Smithton, MO",
 "Smithville, MO", "Solo, MO", "Souder, MO", "South County, MO",
 "South Fork, MO", "South Greenfield, MO", "South West City, MO",
 "Sparta, MO", "Spickard, MO", "Spokane, MO", "Springfield, MO",
 "Squires, MO", "Stanberry, MO", "Stark City, MO", "Steedman, MO",
 "Steele, MO", "Steffenville, MO", "Stella, MO", "Stockton, MO",
 "Stotesbury, MO", "Stotts City, MO", "Stoutland, MO", "Stoutsville, MO",
 "Stover, MO", "Strafford, MO", "Sturdivant, MO", "Sturgeon, MO",
 "Success, MO", "Sugar Creek, MO", "Sullivan, MO", "Summersville, MO",
 "Sumner, MO", "Sunrise Beach, MO", "Sweet Springs, MO", "Sycamore, MO",
 "Syracuse, MO", "Taneyville, MO", "Tarkio, MO", "Taylor, MO",
 "Tebbetts, MO", "Tecumseh, MO", "Teresita, MO", "Thayer, MO",
 "Thompson, MO", "Tiff City, MO", "Tightwad, MO", "Tina, MO",
 "Tipton, MO", "Town And Country, MO", "Trenton, MO", "Trimble, MO",
 "Triplett, MO", "Troy, MO", "Truxton, MO", "Tunas, MO", "Turney, MO",
 "Tuscumbia, MO", "Udall, MO", "Ulman, MO", "Union, MO",
 "Union Star, MO", "Uniontown, MO", "Unionville, MO",
 "University City, MO", "Urbana, MO", "Urich, MO", "Utica, MO",
 "Valley Park, MO", "Van Buren, MO", "Vandalia, MO", "Vanzant, MO",
 "Verona, MO", "Versailles, MO", "Viburnum, MO", "Vichy, MO",
 "Vienna, MO", "Villa Ridge, MO", "Vulcan, MO", "Walker, MO",
 "Walnut Grove, MO", "Walnut Shade, MO", "Wappapello, MO",
 "Warrensburg, MO", "Warrenton, MO", "Warsaw, MO", "Washburn, MO",
 "Washington, MO", "Watson, MO", "Waverly, MO", "Wayland, MO",
 "Weatherby, MO", "Weaubleau, MO", "Webb City, MO", "Webster Groves, MO",
 "Wellington, MO", "Wellsville, MO", "Wentworth, MO", "Wentzville, MO",
 "Wesco, MO", "West Alton, MO", "West County, MO", "West Plains, MO",
 "Westboro, MO", "Weston, MO", "Westphalia, MO", "Wheatland, MO",
 "Wheaton, MO", "Wheeling, MO", "Whiteman Afb, MO", "Whitewater, MO",
 "Willard, MO", "Williamsburg, MO", "Williamstown, MO",
 "Williamsville, MO", "Willow Springs, MO", "Windsor, MO",
 "Windyville, MO", "Winfield, MO", "Winigan, MO", "Winona, MO",
 "Winston, MO", "Wittenberg, MO", "Wooldridge, MO", "Worth, MO",
 "Worthington, MO", "Wright City, MO", "Wyaconda, MO", "Yukon, MO",
 "Zalma, MO", "Zanoni, MO", "Abbeville, MS", "Aberdeen, MS",
 "Ackerman, MS", "Agricola, MS", "Alligator, MS", "Amory, MS",
 "Anguilla, MS", "Askew, MS", "Avalon, MS", "Bailey, MS", "Baird, MS",
 "Baldwyn, MS", "Banner, MS", "Bassfield, MS", "Batesville, MS",
 "Bay Springs, MS", "Beaumont, MS", "Belden, MS", "Belmont, MS",
 "Belzoni, MS", "Benoit, MS", "Benton, MS", "Bentonina, MS", "Beulah, MS",
 "Big Creek, MS", "Biloxi, MS", "Blue Mountain, MS", "Blue Springs, MS",
 "Bogue Chitto, MS", "Bolton, MS", "Booneville, MS", "Boyle, MS",
 "Brandon, MS", "Braxton, MS", "Brookhaven, MS", "Brooklyn, MS",
 "Brooksville, MS", "Bruce, MS", "Buckatunna, MS", "Burnsville, MS",
 "Byhalia, MS", "Caledonia, MS", "Calhoun City, MS", "Camden, MS",

"Cannon, MS", "Canton, MS", "Carlisle, MS", "Carriere, MS",
 "Carrollton, MS", "Carson, MS", "Cascilla, MS", "Cedarbluff, MS",
 "Centreville, MS", "Charleston, MS", "Chunky, MS", "Church Hill, MS",
 "Cleveland, MS", "Clinton, MS", "Coahoma, MS", "Coffeeville, MS",
 "Coila, MS", "Coldwater, MS", "Collins, MS", "Collinsville, MS",
 "Columbia, MS", "Columbus, MS", "Como, MS", "Conehatta, MS",
 "Courtland, MS", "Crawford, MS", "Crosby, MS", "Cruger, MS",
 "Crystal Springs, MS", "Daleville, MS", "De Kalb, MS", "Decatur, MS",
 "Dennis, MS", "Diamondhead, MS", "Doddsville, MS", "Drew, MS",
 "Duck Hill, MS", "Dumas, MS", "Duncan, MS", "Dundee, MS", "Durant, MS",
 "Ecru, MS", "Edinburg, MS", "Edwards, MS", "Egypt, MS", "Elizabeth, MS",
 "Ellisville, MS", "Enid, MS", "Enterprise, MS", "Ethel, MS", "Etta, MS",
 "Falkner, MS", "Fayette, MS", "Flora, MS", "Florence, MS", "Forest, MS",
 "Foxworth, MS", "French Camp, MS", "Fulton, MS", "Gattman, MS",
 "Gautier, MS", "Georgetown, MS", "Glen, MS", "Glen Allan, MS",
 "Gloster, MS", "Golden, MS", "Goodman, MS", "Gore Springs, MS",
 "Greenville, MS", "Greenwood, MS", "Greenwood Spring, MS",
 "Grenada, MS", "Gulfport, MS", "Gunnison, MS", "Guntown, MS",
 "Hamilton, MS", "Harrisville, MS", "Hattiesburg, MS", "Hazlehurst, MS",
 "Heidelberg, MS", "Hermanville, MS", "Hernando, MS", "Hickory, MS",
 "Hickory Flat, MS", "Holcomb, MS", "Holly Bluff, MS",
 "Holly Springs, MS", "Horn Lake, MS", "Houlka, MS", "Houston, MS",
 "Inverness, MS", "Isola, MS", "Itta Bena, MS", "Iuka, MS",
 "Jackson, MS", "Jayess, MS", "Kilmichael, MS", "Kokomo, MS",
 "Kosciusko, MS", "Kossuth, MS", "Kreole, MS", "Lafayette, MS",
 "Lake, MS", "Lake Cormorant, MS", "Lamar, MS", "Lambert, MS",
 "Lauderdale, MS", "Laurel, MS", "Lawrence, MS", "Leaf, MS",
 "Leakesville, MS", "Learned, MS", "Lena, MS", "Lexington, MS",
 "Liberty, MS", "Little Rock, MS", "Long Beach, MS", "Lorman, MS",
 "Louin, MS", "Louise, MS", "Louisville, MS", "Lumberton, MS",
 "Lyon, MS", "Maben, MS", "Macon, MS", "Madden, MS", "Madison, MS",
 "Magee, MS", "Magnolia, MS", "Mantachie, MS", "Mantee, MS",
 "Marietta, MS", "Marks, MS", "Matherville, MS", "Mathiston, MS",
 "Mayersville, MS", "Mc Call Creek, MS", "Mc Carley, MS", "Mc Comb, MS",
 "Mc Cool, MS", "Mc Henry, MS", "Meadville, MS", "Mendenhall, MS",
 "Meridian, MS", "Merigold, MS", "Michigan City, MS", "Minter City, MS",
 "Mize, MS", "Monticello, MS", "Mooreville, MS", "Moorhead, MS",
 "Morton, MS", "Moselle, MS", "Mound Bayou, MS", "Mount Olive, MS",
 "Myrtle, MS", "Natchez, MS", "Neely, MS", "Nesbit, MS", "Nettleton, MS",
 "New Albany, MS", "New Augusta, MS", "New Site, MS", "Newhebron, MS",
 "Newton, MS", "North Bay, MS", "Noxapater, MS", "Oak Vale, MS",
 "Oakland, MS", "Ocean Springs, MS", "Olive Branch, MS", "Osyka, MS",
 "Ovett, MS", "Pachuta, MS", "Pascagoula, MS", "Pass Christian, MS",
 "Pattison, MS", "Paulding, MS", "Pearl, MS", "Pelahatchie, MS",
 "Percy, MS", "Perkinston, MS", "Petal, MS", "Pheba, MS",
 "Philadelphia, MS", "Philipp, MS", "Picayune, MS", "Pickens, MS",
 "Pinola, MS", "Pittsboro, MS", "Plantersville, MS",
 "Pleasant Grove, MS", "Pontotoc, MS", "Pope, MS", "Poplarville, MS",
 "Port Gibson, MS", "Porterville, MS", "Potts Camp, MS", "Prairie, MS",
 "Prentiss, MS", "Preston, MS", "Pulaski, MS", "Purvis, MS",
 "Quitman, MS", "Raleigh, MS", "Red Banks, MS", "Redwood, MS",
 "Richland, MS", "Richton, MS", "Ridgeland, MS", "Rienzi, MS",
 "Ripley, MS", "Robinsonville, MS", "Rolling Fork, MS", "Rose Hill, MS",
 "Rosedale, MS", "Roxie, MS", "Ruleville, MS", "Ruth, MS", "Sallis, MS",
 "Saltillo, MS", "Sandy Hook, MS", "Sardis, MS", "Sarepta, MS",

"Satartia, MS", "Saucier, MS", "Savage, MS", "Schlater, MS",
 "Scobey, MS", "Scooba, MS", "Seminary, MS", "Senatobia, MS",
 "Sessums, MS", "Shannon, MS", "Shaw, MS", "Shelby, MS", "Shuqualak, MS",
 "Sidon, MS", "Silver City, MS", "Silver Creek, MS", "Sledge, MS",
 "Smithdale, MS", "Smithville, MS", "Sontag, MS", "Soso, MS",
 "Southaven, MS", "State Line, MS", "Steens, MS", "Stewart, MS",
 "Stonewall, MS", "Stovall, MS", "Stringer, MS", "Sturgis, MS",
 "Summit, MS", "Sumrall, MS", "Sunflower, MS", "Taylor, MS",
 "Taylorsville, MS", "Tchula, MS", "Terry, MS", "Thaxton, MS",
 "Tillatoba, MS", "Tiplersville, MS", "Tishomingo, MS", "Tomnolen, MS",
 "Toomsuba, MS", "Tremont, MS", "Tunica, MS", "Tupelo, MS",
 "Tutwiler, MS", "Tylertown, MS", "Union, MS", "Union Church, MS",
 "University, MS", "Utica, MS", "Vaiden, MS", "Valley Park, MS",
 "Vance, MS", "Vardaman, MS", "Vicksburg, MS", "Vossburg, MS",
 "Walls, MS", "Walnut, MS", "Walnut Grove, MS", "Water Valley, MS",
 "Waterford, MS", "Waveland, MS", "Waynesboro, MS", "Wayside, MS",
 "Weir, MS", "Wesson, MS", "West, MS", "West Point, MS", "Wiggins, MS",
 "Winona, MS", "Woodland, MS", "Woodville, MS", "Yazoo City, MS",
 "Absarokee, MT", "Acton, MT", "Alberton, MT", "Alzada, MT",
 "Anaconda, MT", "Angela, MT", "Antelope, MT", "Arlee, MT",
 "Ashland, MT", "Augusta, MT", "Babb, MT", "Bainville, MT", "Baker, MT",
 "Ballantine, MT", "Bearcreek, MT", "Belfry, MT", "Belgrade, MT",
 "Belle Creek, MT", "Belt, MT", "Biddle, MT", "Big Arm, MT",
 "Big Sandy, MT", "Big Timber, MT", "Bighorn, MT", "Billings, MT",
 "Billings Heights, MT", "Birney, MT", "Black Eagle, MT",
 "Bloomfield, MT", "Bonner, MT", "Boulder, MT", "Box Elder, MT",
 "Boyes, MT", "Bozeman, MT", "Brady, MT", "Bridger, MT", "Broadview, MT",
 "Brockton, MT", "Brockway, MT", "Brusett, MT", "Buffalo, MT",
 "Busby, MT", "Butte, MT", "Bynum, MT", "Cameron, MT",
 "Canyon Creek, MT", "Capitol, MT", "Cardwell, MT", "Carter, MT",
 "Cascade, MT", "Cat Creek, MT", "Chester, MT", "Chinook, MT",
 "Choteau, MT", "Circle, MT", "Clinton, MT", "Coffee Creek, MT",
 "Cohagen, MT", "Columbia Falls, MT", "Columbus, MT", "Condon, MT",
 "Conner, MT", "Conrad, MT", "Corvallis, MT", "Crow Agency, MT",
 "Culbertson, MT", "Custer, MT", "Cut Bank, MT", "Dagmar, MT",
 "Darby, MT", "Dayton, MT", "Decker, MT", "Deer Lodge, MT", "Dell, MT",
 "Denton, MT", "Dillon, MT", "Divide, MT", "Dixon, MT", "Dodson, MT",
 "Drummond, MT", "Dutton, MT", "East Glacier Par, MT", "East Helena, MT",
 "Ekalaka, MT", "Elmo, MT", "Emigrant, MT", "Ennis, MT", "Essex, MT",
 "Eureka, MT", "Evergreen, MT", "Fairfield, MT", "Fairview, MT",
 "Fallon, MT", "Fishtail, MT", "Flaxville, MT", "Florence, MT",
 "Floweree, MT", "Forestgrove, MT", "Forsyth, MT", "Fort Benton, MT",
 "Fort Peck, MT", "Fort Shaw, MT", "Frenchtown, MT", "Froid, MT",
 "Fromberg, MT", "Galata, MT", "Gallatin Gateway, MT", "Gardiner, MT",
 "Garrison, MT", "Garryowen, MT", "Geraldine, MT", "Geyser, MT",
 "Gildford, MT", "Glasgow, MT", "Glendive, MT", "Gold Creek, MT",
 "Grass Range, MT", "Great Falls, MT", "Greenough, MT", "Greycliff, MT",
 "Hall, MT", "Hamilton, MT", "Hammond, MT", "Hardin, MT", "Harlem, MT",
 "Harrison, MT", "Havre, MT", "Hays, MT", "Heart Butte, MT",
 "Helena, MT", "Helmville, MT", "Heron, MT", "Highwood, MT",
 "Hilger, MT", "Hingham, MT", "Hinsdale, MT", "Hogeland, MT",
 "Homestead, MT", "Hot Springs, MT", "Huntley, MT", "Huson, MT",
 "Hysham, MT", "Ingomar, MT", "Inverness, MT", "Ismay, MT",
 "Jackson, MT", "Joplin, MT", "Jordan, MT", "Judith Gap, MT",
 "Kevin, MT", "Kila, MT", "Kinsey, MT", "Kremlin, MT", "Lakeside, MT",

"Lambert, MT", "Lame Deer, MT", "Larslan, MT", "Laurel, MT",
"Lavina, MT", "Ledger, MT", "Lewistown, MT", "Libby, MT", "Lima, MT",
"Lincoln, MT", "Lindsay, MT", "Livingston, MT", "Lloyd, MT",
"Lodge Grass, MT", "Lolo, MT", "Loma, MT", "Lonepine, MT", "Loring, MT",
"Lustre, MT", "Luther, MT", "Malta, MT", "Manhattan, MT", "Marion, MT",
"Martinsdale, MT", "Mc Leod, MT", "Medicine Lake, MT", "Melville, MT",
"Mildred, MT", "Miles City, MT", "Missoula, MT", "Moccasin, MT",
"Moiese, MT", "Molt, MT", "Monarch, MT", "Montana City, MT",
"Moore, MT", "Mosby, MT", "Musselshell, MT", "Nashua, MT",
"Neihart, MT", "Niarada, MT", "Norris, MT", "Noxon, MT", "Nye, MT",
"Olive, MT", "Opheim, MT", "Otter, MT", "Outlook, MT", "Ovando, MT",
"Park City, MT", "Peerless, MT", "Pendroy, MT", "Philipsburg, MT",
"Plains, MT", "Plentywood, MT", "Plevna, MT", "Polebridge, MT",
"Polson, MT", "Pompeys Pillar, MT", "Pony, MT", "Poplar, MT",
"Powderville, MT", "Power, MT", "Pray, MT", "Proctor, MT",
"Radersburg, MT", "Ramsay, MT", "Rapelje, MT", "Raymond, MT",
"Raynesford, MT", "Red Lodge, MT", "Redstone, MT", "Reedpoint, MT",
"Reserve, MT", "Rexford, MT", "Richey, MT", "Richland, MT",
"Ringling, MT", "Roberts, MT", "Rollins, MT", "Ronan, MT", "Roscoe, MT",
"Rosebud, MT", "Roundup, MT", "Roy, MT", "Rudyard, MT", "Ryegate, MT",
"Saco, MT", "Saint Ignatius, MT", "Saint Mary, MT", "Saint Regis, MT",
"Saint Xavier, MT", "Sand Coulee, MT", "Sand Springs, MT", "Savage, MT",
"Scobey, MT", "Seeley Lake, MT", "Shawmut, MT", "Shelby, MT",
"Shepherd, MT", "Sheridan, MT", "Sidney, MT", "Silesia, MT",
"Silver Star, MT", "Somers, MT", "Stanford, MT", "Stevensville, MT",
"Stockett, MT", "Sula, MT", "Sun River, MT", "Sunburst, MT",
"Superior, MT", "Swan Lake, MT", "Sweetgrass, MT", "Terry, MT",
"Thompson Falls, MT", "Three Forks, MT", "Toston, MT", "Townsend, MT",
"Trout Creek, MT", "Troy, MT", "Turner, MT", "Twin Bridges, MT",
"Twodot, MT", "Utica, MT", "Valier, MT", "Vaughn, MT", "Victor, MT",
"Vida, MT", "Virginia City, MT", "Volborg, MT", "Walkerville, MT",
"Warm Springs, MT", "West Yellowstone, MT", "Westby, MT",
"White Sulphur Sp, MT", "Whitefish, MT", "Whitehall, MT",
"Whitetail, MT", "Whitewater, MT", "Whitlash, MT", "Wibaux, MT",
"Willard, MT", "Wilsall, MT", "Winifred, MT", "Winnett, MT",
"Winston, MT", "Wisdom, MT", "Wise River, MT", "Wolf Creek, MT",
"Wolf Point, MT", "Worden, MT", "Wyola, MT", "Zortman, MT",
"Aberdeen, NC", "Advance, NC", "Ahoskie, NC", "Albemarle, NC",
"Albertson, NC", "Alexander, NC", "Alexander Mills, NC", "Alexis, NC",
"Almond, NC", "Andrews, NC", "Angier, NC", "Apex, NC", "Aquone, NC",
"Arapahoe, NC", "Ararat, NC", "Archdale, NC", "Arden, NC", "Ash, NC",
"Asheville, NC", "Atkinson, NC", "Atlantic, NC", "Aulander, NC",
"Aurora, NC", "Autryville, NC", "Ayden, NC", "Aydlett, NC",
"Bahama, NC", "Bailey, NC", "Bakersville, NC", "Balsam Grove, NC",
"Banner Elk, NC", "Barco, NC", "Barnardsville, NC", "Bath, NC",
"Battleboro, NC", "Bayboro, NC", "Bear Creek, NC", "Beaufort, NC",
"Belews Creek, NC", "Belhaven, NC", "Belmont, NC", "Belvidere, NC",
"Bennett, NC", "Benson, NC", "Bessemer City, NC", "Bethel, NC",
"Beulaville, NC", "Biscoe, NC", "Black Mountain S, NC",
"Bladenboro, NC", "Blanch, NC", "Blounts Creek, NC", "Blowing Rock, NC",
"Boger City, NC", "Boiling Spring L, NC", "Bolivia, NC", "Bolton, NC",
"Bonnie Doone, NC", "Boomer, NC", "Boone, NC", "Boonville, NC",
"Bostic, NC", "Bowdens, NC", "Brasstown, NC", "Brevard, NC",
"Broadway, NC", "Browns Summit, NC", "Bryson City, NC", "Bullock, NC",
"Bunnlevel, NC", "Burgaw, NC", "Burlington, NC", "Burnsville, NC",

"Butner, NC", "Calabash, NC", "Camden, NC", "Camp Lejeune, NC",
"Candler, NC", "Candor, NC", "Canton, NC", "Cape Fear, NC",
"Carolina Beach, NC", "Carrboro, NC", "Carthage, NC", "Cary, NC",
"Casar, NC", "Cashiers, NC", "Castalia, NC", "Castle Hayne, NC",
"Catawba, NC", "Cedar Grove, NC", "Cedar Island, NC", "Cerro Gordo, NC",
"Chadbourn, NC", "Chapel Hill, NC", "Charlotte, NC", "Cherokee, NC",
"Cherryville, NC", "China Grove, NC", "Chinquapin, NC",
"Chocowinity, NC", "Claremont, NC", "Clarendon, NC", "Clarkton, NC",
"Clayton, NC", "Clemmons, NC", "Cleveland, NC", "Climax, NC",
"Clinton, NC", "Clyde, NC", "Coats, NC", "Cofield, NC", "Coinjock, NC",
"Colerain, NC", "Coleridge, NC", "Colfax, NC", "Collettsville, NC",
"Colon, NC", "Columbia, NC", "Columbus, NC", "Como, NC", "Concord, NC",
"Connellys Spring, NC", "Conover, NC", "Conway, NC", "Corapeake, NC",
"Cornelius, NC", "Corolla, NC", "Council, NC", "Cove City, NC",
"Cramerton, NC", "Creedmoor, NC", "Creston, NC", "Creswell, NC",
"Crisp, NC", "Crouse, NC", "Crumpler, NC", "Cullowhee, NC",
"Currie, NC", "Currituck, NC", "Dallas, NC", "Danbury, NC",
"Deep Gap, NC", "Deep Run, NC", "Delco, NC", "Denton, NC", "Denver, NC",
"Dobson, NC", "Dover, NC", "Dudley, NC", "Dunn, NC", "Durants Neck, NC",
"Durham, NC", "Eagle Springs, NC", "East Bend, NC",
"East Fayettevill, NC", "East Flat Rock, NC", "East Lake, NC",
"Eden, NC", "Edenton, NC", "Edward, NC", "Efland, NC",
"Elizabeth City, NC", "Elizabethtown, NC", "Elk Park, NC", "Elkin, NC",
"Ellenboro, NC", "Ellerbe, NC", "Elm City, NC", "Elon College, NC",
"Emerald Isle, NC", "Enfield, NC", "Ennice, NC", "Ernul, NC",
"Erwin, NC", "Etowah, NC", "Eure, NC", "Eureka, NC", "Evergreen, NC",
"Fair Bluff, NC", "Fairfield, NC", "Fairview, NC", "Faison, NC",
"Farmer, NC", "Farmville, NC", "Fayetteville, NC", "Ferguson, NC",
"Flat Rock, NC", "Fleetwood, NC", "Fletcher, NC", "Fontana Dam, NC",
"Fort Bragg, NC", "Fountain, NC", "Four Oaks, NC", "Frank, NC",
"Franklin, NC", "Franklinton, NC", "Franklinville, NC",
"Fuquay Varina, NC", "Garland, NC", "Garner, NC", "Garysburg, NC",
"Gaston, NC", "Gastonia, NC", "Gates, NC", "Gatesville, NC",
"George, NC", "Germanton, NC", "Gerton, NC", "Gibson, NC",
"Gibsonville, NC", "Glade Valley, NC", "Glen Raven, NC",
"Glenville, NC", "Gloucester, NC", "Godwin, NC", "Gold Hill, NC",
"Goldsboro, NC", "Goldston, NC", "Graham, NC", "Grandy, NC",
"Granite Falls, NC", "Grantham, NC", "Grantsboro, NC",
"Grassy Creek, NC", "Greenmountain, NC", "Greensboro, NC",
"Greenville, NC", "Grifton, NC", "Grimesland, NC", "Grover, NC",
"Halifax, NC", "Hallsboro, NC", "Hamilton, NC", "Hamlet, NC",
"Hampstead, NC", "Hamptonville, NC", "Harbinger, NC",
"Harkers Island, NC", "Harmony, NC", "Harrells, NC",
"Harrellsville, NC", "Harrisburg, NC", "Havelock, NC", "Haw River, NC",
"Hayesville, NC", "Hays, NC", "Hazelwood, NC", "Henderson, NC",
"Hendersonville, NC", "Henrico, NC", "Hickory, NC", "Hiddenite, NC",
"High Point, NC", "Highlands, NC", "Hillsborough, NC", "Hobbsville, NC",
"Hobgood, NC", "Hobucken, NC", "Hoffman, NC", "Holden Beach, NC",
"Hollister, NC", "Holly Springs, NC", "Hookerton, NC", "Hope Mills, NC",
"Horse Shoe, NC", "Hot Springs, NC", "Hubert, NC", "Hudson, NC",
"Hurdle Mills, NC", "Indian Trail, NC", "Iron Station, NC",
"Ivanhoe, NC", "Jackson, NC", "Jackson Springs, NC", "Jacksonville, NC",
"Jamestown, NC", "Jamesville, NC", "Jarvisburg, NC", "Jefferson, NC",
"Johnsonville, NC", "Jonesville, NC", "Julian, NC", "Kannapolis, NC",
"Kelford, NC", "Kelly, NC", "Kenansville, NC", "Kenly, NC",

"Kernersville, NC", "Kill Devil Hills, NC", "King, NC",
 "Kings Mountain, NC", "Kingstown, NC", "Kinston, NC", "Kittrell, NC",
 "Knightdale, NC", "Knotts Island, NC", "Kure Beach, NC",
 "La Grange, NC", "Lake Junaluska, NC", "Lake Lure, NC",
 "Lake Toxaway, NC", "Lake Waccamaw, NC", "Landis, NC", "Lansing, NC",
 "Lasker, NC", "Laurel Hill, NC", "Laurel Springs, NC", "Laurinburg, NC",
 "Lawndale, NC", "Lawsonville, NC", "Leasburg, NC", "Leicester, NC",
 "Leland, NC", "Lenoir, NC", "Lewiston Woodvil, NC", "Lewisville, NC",
 "Lexington, NC", "Liberty, NC", "Lilesville, NC", "Lillington, NC",
 "Linden, NC", "Linwood, NC", "Littleton, NC", "Locust, NC",
 "Longisland, NC", "Longwood, NC", "Louisburg, NC", "Lowell, NC",
 "Lowgap, NC", "Lowland, NC", "Lucama, NC", "Lumber Bridge, NC",
 "Lumberton, NC", "Macon, NC", "Madison, NC", "Maggie Valley, NC",
 "Magnolia, NC", "Maiden, NC", "Manson, NC", "Manteo, NC", "Maple, NC",
 "Maple Hill, NC", "Marble, NC", "Margarettsville, NC", "Marion, NC",
 "Mars Hill, NC", "Marshallberg, NC", "Marshville, NC", "Marston, NC",
 "Maxton, NC", "Mayodan, NC", "Maysville, NC", "Mc Grady, NC",
 "Mc Leansville, NC", "Mcdonald, NC", "Mebane, NC", "Merritt, NC",
 "Merry Hill, NC", "Middlesex, NC", "Middletown, NC", "Midland, NC",
 "Midway Park, NC", "Mill Spring, NC", "Millers Creek, NC", "Milton, NC",
 "Mocksville, NC", "Moncure, NC", "Monroe, NC", "Mooresboro, NC",
 "Mooresville, NC", "Moravian Falls, NC", "Morehead City, NC",
 "Morganton, NC", "Morrisville, NC", "Morven, NC", "Mount Airy, NC",
 "Mount Gilead, NC", "Mount Holly, NC", "Mount Olive, NC",
 "Mount Pleasant, NC", "Mount Ulla, NC", "Moyock, NC",
 "Murfreesboro, NC", "Nags Head, NC", "Nakina, NC", "Nashville, NC",
 "Nebo, NC", "New Bern, NC", "New Hill, NC", "New London, NC",
 "Newport, NC", "Newton, NC", "Newton Grove, NC", "Norlina, NC",
 "North Wilkesboro, NC", "Norwood, NC", "Oak City, NC", "Oak Island, NC",
 "Oak Ridge, NC", "Oakboro, NC", "Ocean Isle Beach, NC", "Ogden, NC",
 "Old Fort, NC", "Olin, NC", "Oriental, NC", "Orrum, NC", "Otto, NC",
 "Oxford, NC", "Palmyra, NC", "Pantego, NC", "Parkton, NC",
 "Peachland, NC", "Pelham, NC", "Pembroke, NC", "Pendleton, NC",
 "Penrose, NC", "Pfafftown, NC", "Pikeville, NC", "Pilot Mountain, NC",
 "Pine Hall, NC", "Pine Knoll Shore, NC", "Pinehurst, NC",
 "Pinetops, NC", "Pinetown, NC", "Pineville, NC", "Pink Hill, NC",
 "Pinnacle, NC", "Pisgah Forest, NC", "Pittsboro, NC",
 "Pleasant Garden, NC", "Pleasant Hill, NC", "Plymouth, NC",
 "Point Harbor, NC", "Polkton, NC", "Pollocksville, NC",
 "Poplar Branch, NC", "Powells Point, NC", "Princeton, NC",
 "Prospect Hill, NC", "Providence, NC", "Purlear, NC", "Raeford, NC",
 "Raleigh, NC", "Randleman, NC", "Red Springs, NC", "Reidsville, NC",
 "Research Triangl, NC", "Rich Square, NC", "Richfield, NC",
 "Richlands, NC", "Riegelwood, NC", "Roanoke Rapids, NC",
 "Roaring Gap, NC", "Roaring River, NC", "Robbins, NC",
 "Robbinsville, NC", "Robersonville, NC", "Rockingham, NC",
 "Rockwell, NC", "Rocky Mount, NC", "Rocky Point, NC", "Rolesville, NC",
 "Ronda, NC", "Roper, NC", "Rose Hill, NC", "Roseboro, NC", "Rosman, NC",
 "Rougemont, NC", "Rowland, NC", "Roxboro, NC", "Roxobel, NC",
 "Ruffin, NC", "Rural Hall, NC", "Rutherfordton, NC", "Saint Pauls, NC",
 "Salemberg, NC", "Salisbury, NC", "Saluda, NC", "Sandy Ridge, NC",
 "Sapphire, NC", "Saratoga, NC", "Scaly Mountain, NC",
 "Scotland Neck, NC", "Scranton, NC", "Seaboard, NC", "Seagrove, NC",
 "Sealevel, NC", "Selma, NC", "Semora, NC", "Seven Springs, NC",
 "Seymour Johnson, NC", "Shallotte, NC", "Shannon, NC", "Shawboro, NC",

"Shelby, NC", "Sherrills Ford, NC", "Shiloh, NC", "Siler City, NC",
 "Siloam, NC", "Sims, NC", "Smithfield, NC", "Smyrna, NC",
 "Sneads Ferry, NC", "Snow Camp, NC", "Snow Hill, NC", "Sophia, NC",
 "South Mills, NC", "Southern Pines, NC", "Southern Shores, NC",
 "Sparta, NC", "Spencer, NC", "Spindale, NC", "Spring Hope, NC",
 "Spring Lake, NC", "Spruce Pine, NC", "Stacy, NC", "Staley, NC",
 "Stallings, NC", "Stanfield, NC", "Stanley, NC", "Stantonsburg, NC",
 "Star, NC", "State Road, NC", "Statesville, NC", "Stedman, NC",
 "Stella, NC", "Stem, NC", "Stokes, NC", "Stokesdale, NC",
 "Stoneville, NC", "Stony Point, NC", "Stumpy Point, NC",
 "Sugar Grove, NC", "Summerfield, NC", "Sunbury, NC", "Sunset Beach, NC",
 "Surf City, NC", "Swanquarter, NC", "Swansboro, NC", "Sylva, NC",
 "Tabor City, NC", "Tapoco, NC", "Tar Heel, NC", "Tarawa Terrace, NC",
 "Tarboro, NC", "Taylorsville, NC", "Teachey, NC", "Terrell, NC",
 "Thomasville, NC", "Thurmond, NC", "Timberlake, NC", "Tobaccoville, NC",
 "Todd, NC", "Topton, NC", "Traphill, NC", "Trenton, NC", "Trinity, NC",
 "Triplett, NC", "Troutman, NC", "Troy, NC", "Tryon, NC",
 "Tuckasegee, NC", "Turkey, NC", "Tyner, NC", "Unaka, NC",
 "Union Grove, NC", "Union Mills, NC", "Valdese, NC", "Vale, NC",
 "Valle Crucis, NC", "Vanceboro, NC", "Vandemere, NC", "Vass, NC",
 "Vilas, NC", "Wade, NC", "Wadesboro, NC", "Wagram, NC",
 "Wake Forest, NC", "Walkertown, NC", "Wallace, NC", "Walnut, NC",
 "Walnut Cove, NC", "Walstonburg, NC", "Warne, NC",
 "Warren Wilson Co, NC", "Warrensville, NC", "Warrenton, NC",
 "Washington, NC", "Watha, NC", "Waynesville, NC", "Weaverville, NC",
 "Weddington, NC", "Weldon, NC", "Wendell, NC", "Wesleyan College, NC",
 "West End, NC", "West Jefferson, NC", "Westfield, NC", "Whitakers, NC",
 "White Oak, NC", "Whiteville, NC", "Whitsett, NC", "Whittier, NC",
 "Wilkesboro, NC", "Willard, NC", "Williamston, NC", "Willow Spring, NC",
 "Wilmington, NC", "Wilson, NC", "Windsor, NC", "Wingate, NC",
 "Winnabow, NC", "Winston Salem, NC", "Winterville, NC", "Winton, NC",
 "Woodleaf, NC", "Wrightsville Bea, NC", "Yadkinville, NC",
 "Yanceyville, NC", "Youngsville, NC", "Zebulon, NC", "Zionville, NC",
 "Zirconia, NC", "Absaraka, ND", "Adams, ND", "Adrian, ND", "Alfred, ND",
 "Alice, ND", "Alkabo, ND", "Almont, ND", "Ambrose, ND", "Amenia, ND",
 "Amidon, ND", "Anamoose, ND", "Aneta, ND", "Antler, ND", "Appam, ND",
 "Ardoch, ND", "Arena, ND", "Argusville, ND", "Arnegard, ND",
 "Arthur, ND", "Arvilla, ND", "Ashley, ND", "Ayr, ND", "Backoo, ND",
 "Baker, ND", "Baldwin, ND", "Balfour, ND", "Bantry, ND", "Barney, ND",
 "Barton, ND", "Bathgate, ND", "Battlevue, ND", "Beach, ND",
 "Belcourt, ND", "Belden, ND", "Benedict, ND", "Bentley, ND",
 "Bergen, ND", "Berlin, ND", "Berwick, ND", "Beulah, ND", "Binford, ND",
 "Bisbee, ND", "Bismarck, ND", "Blaisdell, ND", "Blanchard, ND",
 "Bonetrail, ND", "Bordulac, ND", "Bottineau, ND", "Bowdon, ND",
 "Bowesmont, ND", "Bowman, ND", "Braddock, ND", "Brampton, ND",
 "Brantford, ND", "Breien, ND", "Bremen, ND", "Briarwood, ND",
 "Brinsmade, ND", "Brocket, ND", "Buchanan, ND", "Bucyrus, ND",
 "Buffalo, ND", "Burlington, ND", "Burnstad, ND", "Burt, ND",
 "Butte, ND", "Buxton, ND", "Caledonia, ND", "Calio, ND", "Calvin, ND",
 "Cannon Ball, ND", "Carbury, ND", "Carpio, ND", "Carson, ND",
 "Cartwright, ND", "Casselton, ND", "Cayuga, ND", "Chaffee, ND",
 "Charlson, ND", "Chaseley, ND", "Christine, ND", "Churchs Ferry, ND",
 "Clifford, ND", "Coleharbor, ND", "Colfax, ND", "Colgan, ND",
 "Colgate, ND", "Concrete, ND", "Cooperstown, ND", "Coteau, ND",
 "Coulee, ND", "Courtenay, ND", "Crete, ND", "Crosby, ND", "Crystal, ND",

"Cummings, ND", "Dahlen, ND", "Davenport, ND", "Dawson, ND",
 "Deering, ND", "Delamere, ND", "Denhoff, ND", "Des Lacs, ND",
 "Devils Lake, ND", "Dickey, ND", "Dodge, ND", "Donnybrook, ND",
 "Douglas, ND", "Doyon, ND", "Drake, ND", "Driscoll, ND",
 "Dunn Center, ND", "Durbin, ND", "Dwight, ND", "Eckelson, ND",
 "Edmore, ND", "Edmunds, ND", "Egeland, ND", "Eldridge, ND",
 "Ellendale, ND", "Elliott, ND", "Embsden, ND", "Emerado, ND",
 "Emmet, ND", "Emrick, ND", "Enderlin, ND", "Englevale, ND", "Erie, ND",
 "Fairdale, ND", "Fairmount, ND", "Fargo, ND", "Fessenden, ND",
 "Fillmore, ND", "Fingal, ND", "Finley, ND", "Flora, ND", "Forbes, ND",
 "Fordville, ND", "Forest River, ND", "Forman, ND", "Fort Clark, ND",
 "Fort Yates, ND", "Foxholm, ND", "Fredonia, ND", "Fryburg, ND",
 "Fullerton, ND", "Gackle, ND", "Galesburg, ND", "Gardar, ND",
 "Gardena, ND", "Gardner, ND", "Gascoyne, ND", "Geneseo, ND",
 "Gladstone, ND", "Glen Ullin, ND", "Golden Valley, ND", "Golva, ND",
 "Goodrich, ND", "Gorham, ND", "Grace City, ND", "Grand Forks, ND",
 "Grand Rapids, ND", "Grandin, ND", "Granville, ND", "Grassy Butte, ND",
 "Great Bend, ND", "Guelph, ND", "Hague, ND", "Hamar, ND", "Hamberg, ND",
 "Hamilton, ND", "Hamlet, ND", "Hampden, ND", "Hankinson, ND",
 "Hannah, ND", "Hannover, ND", "Hansboro, ND", "Harlow, ND",
 "Hastings, ND", "Hatton, ND", "Havana, ND", "Hazelton, ND", "Hazen, ND",
 "Heaton, ND", "Hebron, ND", "Heil, ND", "Heimdal, ND", "Hensel, ND",
 "Hickson, ND", "Honeyford, ND", "Hoople, ND", "Huff, ND", "Hunter, ND",
 "Hurdsfield, ND", "Joliet, ND", "Juanita, ND", "Karlsruhe, ND",
 "Keene, ND", "Kelso, ND", "Kempston, ND", "Kensal, ND", "Kief, ND",
 "Killdeer, ND", "Kindred, ND", "Kintyre, ND", "Kloten, ND", "Knox, ND",
 "Kramer, ND", "Kulm, ND", "Lake Williams, ND", "Langdon, ND",
 "Lankin, ND", "Lansford, ND", "Lark, ND", "Larson, ND", "Lawton, ND",
 "Leal, ND", "Lefor, ND", "Lehr, ND", "Leith, ND", "Leonard, ND",
 "Lignite, ND", "Lincoln, ND", "Litchville, ND", "Loma, ND",
 "Lorraine, ND", "Luverne, ND", "Maida, ND", "Makoti, ND", "Mandan, ND",
 "Mandaree, ND", "Manfred, ND", "Manning, ND", "Mantador, ND",
 "Manvel, ND", "Mapes, ND", "Marion, ND", "Marmarth, ND", "Martin, ND",
 "Max, ND", "Maxbass, ND", "Mayville, ND", "Maza, ND", "McCanna, ND",
 "Mcclusky, ND", "Mcgregor, ND", "Mchenry, ND", "Mckenzie, ND",
 "McLeod, ND", "Medina, ND", "Medora, ND", "Mekinock, ND", "Menoken, ND",
 "Mercer, ND", "Merricourt, ND", "Milton, ND", "Minnewaukan, ND",
 "Minot, ND", "Minot Afb, ND", "Moffit, ND", "Monango, ND",
 "Mooreton, ND", "Mountain, ND", "Mylo, ND", "Nanson, ND",
 "Napoleon, ND", "Nash, ND", "Nech, ND", "New England, ND",
 "New Hradec, ND", "Newburg, ND", "Niagara, ND", "Nome, ND",
 "Noonan, ND", "North River, ND", "Northgate, ND", "Nortonville, ND",
 "Norwich, ND", "Oberon, ND", "Oriska, ND", "Orr, ND", "Osnabrock, ND",
 "Overly, ND", "Page, ND", "Palermo, ND", "Park River, ND",
 "Parshall, ND", "Pekin, ND", "Penn, ND", "Perth, ND", "Petersburg, ND",
 "Pettibone, ND", "Pisek, ND", "Plaza, ND", "Pleasant Lake, ND",
 "Portal, ND", "Portland, ND", "Prosper, ND", "Raleigh, ND", "Raub, ND",
 "Rawson, ND", "Reeder, ND", "Regan, ND", "Regent, ND", "Reynolds, ND",
 "Rhame, ND", "Richardton, ND", "Riverdale, ND", "Riverside, ND",
 "Rocklake, ND", "Rolla, ND", "Roseglen, ND", "Ross, ND", "Ruso, ND",
 "Rutland, ND", "Saint Anthony, ND", "Saint John, ND",
 "Saint Michael, ND", "Saint Thomas, ND", "San Haven, ND", "Sanborn, ND",
 "Sarles, ND", "Sawyer, ND", "Selfridge, ND", "Sentinel Butte, ND",
 "Sharon, ND", "Sheldon, ND", "Sherwood, ND", "Sheyenne, ND",
 "Shields, ND", "Sibley, ND", "South Heart, ND", "Southam, ND",

"Spiritwood, ND", "Springbrook, ND", "Stanton, ND", "Starkweather, ND",
 "Steele, ND", "Sterling, ND", "Stirum, ND", "Strasburg, ND",
 "Streeter, ND", "Surrey, ND", "Sutton, ND", "Sykeston, ND",
 "Tappen, ND", "Taylor, ND", "Temple, ND", "Temvik, ND", "Thompson, ND",
 "Tolley, ND", "Tower City, ND", "Trenton, ND", "Trotters, ND",
 "Turtle Lake, ND", "Tuttle, ND", "Underwood, ND", "Upham, ND",
 "Valley City, ND", "Velva, ND", "Venturia, ND", "Verona, ND",
 "Voss, ND", "Walcott, ND", "Wales, ND", "Walum, ND", "Warwick, ND",
 "Washburn, ND", "Watford City, ND", "Webster, ND", "Werner, ND",
 "Westhope, ND", "Wheelock, ND", "White Earth, ND", "Whitman, ND",
 "Willow City, ND", "Wilton, ND", "Wimbledon, ND", "Windsor, ND",
 "Wing, ND", "Wolford, ND", "Wolseth, ND", "Woodworth, ND",
 "Wyndmere, ND", "Ypsilanti, ND", "Zahl, ND", "Zap, ND", "Zeeland, ND",
 "Abie, NE", "Adams, NE", "Agnew, NE", "Ainsworth, NE", "Albion, NE",
 "Alda, NE", "Alexandria, NE", "Allen, NE", "Alliance, NE", "Alma, NE",
 "Almeria, NE", "Alvo, NE", "Amelia, NE", "Ames, NE", "Amherst, NE",
 "Angora, NE", "Ansley, NE", "Arapahoe, NE", "Arcadia, NE", "Archer, NE",
 "Arlington, NE", "Arnold, NE", "Arthur, NE", "Ashby, NE", "Ashland, NE",
 "Ashton, NE", "Atkinson, NE", "Atlanta, NE", "Auburn, NE", "Aurora, NE",
 "Avoca, NE", "Axtell, NE", "Ayr, NE", "Bancroft, NE", "Bartlett, NE",
 "Bartley, NE", "Bassett, NE", "Battle Creek, NE", "Bayard, NE",
 "Beatrice, NE", "Beaver City, NE", "Beaver Crossing, NE", "Bee, NE",
 "Beemer, NE", "Belden, NE", "Belgrade, NE", "Bellevue, NE",
 "Bellwood, NE", "Belvidere, NE", "Benedict, NE", "Benkelman, NE",
 "Bennet, NE", "Bennington, NE", "Bertrand, NE", "Berwyn, NE",
 "Big Springs, NE", "Bingham, NE", "Bladen, NE", "Blair, NE",
 "Bloomfield, NE", "Bloomington, NE", "Blue Hill, NE",
 "Blue Springs, NE", "Boelus, NE", "Bradshaw, NE", "Brady, NE",
 "Brainard, NE", "Brewster, NE", "Bridgeport, NE", "Bristow, NE",
 "Broadwater, NE", "Brock, NE", "Broken Bow, NE", "Brownlee, NE",
 "Brownville, NE", "Brule, NE", "Bruning, NE", "Bruno, NE",
 "Brunswick, NE", "Burchard, NE", "Burr, NE", "Burwell, NE",
 "Bushnell, NE", "Butte, NE", "Byron, NE", "Cairo, NE", "Callaway, NE",
 "Cambridge, NE", "Campbell, NE", "Carleton, NE", "Carroll, NE",
 "Cedar Bluffs, NE", "Cedar Rapids, NE", "Center, NE",
 "Central City, NE", "Ceresco, NE", "Chadron, NE", "Chambers, NE",
 "Champion, NE", "Chapman, NE", "Chappell, NE", "Chester, NE",
 "Clarks, NE", "Clarkson, NE", "Clatonia, NE", "Clay Center, NE",
 "Clearwater, NE", "Cody, NE", "Coleridge, NE", "Colon, NE",
 "Comstock, NE", "Concord, NE", "Cook, NE", "Cordova, NE",
 "Cortland, NE", "Cotesfield, NE", "Cozad, NE", "Crab Orchard, NE",
 "Craig, NE", "Crawford, NE", "Creighton, NE", "Creston, NE",
 "Crete, NE", "Crofton, NE", "Crookston, NE", "Culbertson, NE",
 "Curtis, NE", "Dakota City, NE", "Dalton, NE", "Danbury, NE",
 "Dannebrog, NE", "Davenport, NE", "Davey, NE", "Dawson, NE",
 "Daykin, NE", "De Witt, NE", "Decatur, NE", "Denton, NE", "Deshler, NE",
 "Deweese, NE", "Dickens, NE", "Diller, NE", "Dix, NE", "Dixon, NE",
 "Dodge, NE", "Doniphan, NE", "Dorchester, NE", "Douglas, NE",
 "Du Bois, NE", "Dunbar, NE", "Dunning, NE", "Dwight, NE", "Eagle, NE",
 "Eddyville, NE", "Edgar, NE", "Edison, NE", "Elba, NE", "Elgin, NE",
 "Elk Creek, NE", "Elkhorn, NE", "Ellsworth, NE", "Elm Creek, NE",
 "Elmwood, NE", "Elsie, NE", "Elsmere, NE", "Elwood, NE", "Elyria, NE",
 "Emerson, NE", "Emmet, NE", "Enders, NE", "Endicott, NE", "Ericson, NE",
 "Eustis, NE", "Ewing, NE", "Exeter, NE", "Fairbury, NE",
 "Fairfield, NE", "Fairmont, NE", "Falls City, NE", "Farnam, NE",

"Farwell, NE", "Filley, NE", "Firth, NE", "Fordyce, NE",
 "Fort Calhoun, NE", "Foster, NE", "Franklin, NE", "Fremont, NE",
 "Friend, NE", "Fullerton, NE", "Funk, NE", "Garland, NE",
 "Garrison, NE", "Geneva, NE", "Genoa, NE", "Gering, NE", "Gibbon, NE",
 "Gilead, NE", "Giltner, NE", "Glenvil, NE", "Goehner, NE", "Gordon, NE",
 "Gothenburg, NE", "Grafton, NE", "Grand Island, NE", "Grant, NE",
 "Greeley, NE", "Greenwood, NE", "Gresham, NE", "Gretna, NE",
 "Guide Rock, NE", "Gurley, NE", "Haigler, NE", "Hallam, NE",
 "Halsey, NE", "Hamlet, NE", "Hampton, NE", "Hardy, NE",
 "Harrisburg, NE", "Harrison, NE", "Hartington, NE", "Harvard, NE",
 "Hastings, NE", "Hay Springs, NE", "Hayes Center, NE", "Hazard, NE",
 "Heartwell, NE", "Hebron, NE", "Hemingford, NE", "Henderson, NE",
 "Hendley, NE", "Henry, NE", "Herman, NE", "Hershey, NE", "Hildreth, NE",
 "Holbrook, NE", "Holdrege, NE", "Holland, NE", "Holmesville, NE",
 "Holstein, NE", "Hooper, NE", "Hordville, NE", "Hoskins, NE",
 "Howells, NE", "Hubbard, NE", "Hubbell, NE", "Humboldt, NE",
 "Humphrey, NE", "Huntley, NE", "Hyannis, NE", "Imperial, NE",
 "Inavale, NE", "Indianola, NE", "Inland, NE", "Inman, NE", "Ithaca, NE",
 "Jackson, NE", "Jansen, NE", "Johnson, NE", "Johnstown, NE",
 "Julian, NE", "Juniata, NE", "Kearney, NE", "Kenesaw, NE",
 "Kennard, NE", "Keystone, NE", "Kilgore, NE", "Kimball, NE",
 "Lakeside, NE", "Lamar, NE", "Laurel, NE", "Lawrence, NE",
 "Lebanon, NE", "Leigh, NE", "Lemoyne, NE", "Leshara, NE",
 "Lewellen, NE", "Lewiston, NE", "Lexington, NE", "Liberty, NE",
 "Lincoln, NE", "Lindsay, NE", "Linwood, NE", "Lisco, NE",
 "Litchfield, NE", "Lodgepole, NE", "Long Pine, NE", "Loomis, NE",
 "Louisville, NE", "Loup City, NE", "Lyman, NE", "Lynch, NE",
 "Lyons, NE", "Macy, NE", "Madison, NE", "Madrid, NE", "Magnet, NE",
 "Malcolm, NE", "Malmo, NE", "Marquette, NE", "Marsland, NE",
 "Martell, NE", "Maskell, NE", "Mason City, NE", "Max, NE",
 "Maxwell, NE", "Maywood, NE", "Mc Cook, NE", "Mc Cool Junction, NE",
 "Mclean, NE", "Mead, NE", "Meadow Grove, NE", "Merna, NE",
 "Merriman, NE", "Milburn, NE", "Milford, NE", "Millard, NE",
 "Miller, NE", "Milligan, NE", "Mills, NE", "Minatare, NE", "Minden, NE",
 "Mitchell, NE", "Monroe, NE", "Moorefield, NE", "Morrill, NE",
 "Morse Bluff, NE", "Mullen, NE", "Murdock, NE", "Murray, NE",
 "Naper, NE", "Naponee, NE", "Nebraska City, NE", "Nehawka, NE",
 "Neligh, NE", "Nemaha, NE", "Newcastle, NE", "Newman Grove, NE",
 "Newport, NE", "Nickerson, NE", "Nora, NE", "Norfolk, NE",
 "North Bend, NE", "North Loup, NE", "North Platte, NE", "Oak, NE",
 "Oakdale, NE", "Oakland, NE", "Obert, NE", "Oconto, NE", "Octavia, NE",
 "Odell, NE", "Odessa, NE", "Offutt A F B, NE", "Ogallala, NE",
 "Ohiowa, NE", "Omaha, NE", "Oneill, NE", "Ong, NE", "Orchard, NE",
 "Ord, NE", "Orleans, NE", "Osceola, NE", "Oshkosh, NE", "Osmond, NE",
 "Otoe, NE", "Overton, NE", "Oxford, NE", "Page, NE", "Palisade, NE",
 "Palmer, NE", "Palmyra, NE", "Papillion, NE", "Parks, NE",
 "Pawnee City, NE", "Paxton, NE", "Pender, NE", "Peru, NE",
 "Petersburg, NE", "Phillips, NE", "Pickrell, NE", "Pierce, NE",
 "Pilger, NE", "Plainview, NE", "Platte Center, NE", "Plattsmouth, NE",
 "Pleasant Dale, NE", "Pleasanton, NE", "Plymouth, NE", "Polk, NE",
 "Ponca, NE", "Potter, NE", "Prague, NE", "Primrose, NE", "Prosser, NE",
 "Purdum, NE", "Ragan, NE", "Ralston, NE", "Randolph, NE", "Ravenna, NE",
 "Red Cloud, NE", "Republican City, NE", "Reynolds, NE", "Richfield, NE",
 "Richland, NE", "Rising City, NE", "Riverdale, NE", "Riverton, NE",
 "Roca, NE", "Rockville, NE", "Rogers, NE", "Rosalie, NE", "Rose, NE",

"Roseland, NE", "Royal, NE", "Rulo, NE", "Rushville, NE", "Ruskin, NE",
 "Saint Edward, NE", "Saint Helena, NE", "Saint Libory, NE",
 "Saint Mary, NE", "Saint Paul, NE", "Salem, NE", "Sargent, NE",
 "Saronville, NE", "Schuyler, NE", "Scotia, NE", "Scottsbluff, NE",
 "Scribner, NE", "Seneca, NE", "Seward, NE", "Shelby, NE", "Shelton, NE",
 "Shickley, NE", "Shubert, NE", "Sidney, NE", "Silver Creek, NE",
 "Smithfield, NE", "South Sioux City, NE", "Spalding, NE", "Spencer, NE",
 "Springfield, NE", "Springview, NE", "Stamford, NE", "Stanton, NE",
 "Staplehurst, NE", "Stapleton, NE", "Steele City, NE", "Steinauer, NE",
 "Stella, NE", "Sterling, NE", "Stockville, NE", "Strang, NE",
 "Stratton, NE", "Stromsburg, NE", "Stuart, NE", "Sumner, NE",
 "Superior, NE", "Sutherland, NE", "Sutton, NE", "Swanton, NE",
 "Syracuse, NE", "Table Rock, NE", "Talmage, NE", "Tecumseh, NE",
 "Tekamah, NE", "Thurston, NE", "Tilden, NE", "Tobias, NE",
 "Trenton, NE", "Trumbull, NE", "Tryon, NE", "Ulysses, NE",
 "Unadilla, NE", "Union, NE", "Upland, NE", "Utica, NE", "Valentine, NE",
 "Valley, NE", "Valparaiso, NE", "Venango, NE", "Verdel, NE",
 "Verdigre, NE", "Verdon, NE", "Virginia, NE", "Waco, NE", "Wahoo, NE",
 "Wakefield, NE", "Wallace, NE", "Walthill, NE", "Walton, NE",
 "Waterbury, NE", "Waterloo, NE", "Wauneta, NE", "Wausa, NE",
 "Waverly, NE", "Wayne, NE", "Weeping Water, NE", "Wellfleet, NE",
 "West Point, NE", "Western, NE", "Westerville, NE", "Weston, NE",
 "Whitman, NE", "Whitney, NE", "Wilber, NE", "Wilcox, NE",
 "Wilsonville, NE", "Winnebago, NE", "Winnetoon, NE", "Winside, NE",
 "Wisner, NE", "Wolbach, NE", "Wood Lake, NE", "Wood River, NE",
 "Wymore, NE", "Wynot, NE", "York, NE", "Yutan, NE", "Allenstown, NH",
 "Alstead, NH", "Alton, NH", "Alton Bay, NH", "Amherst, NH",
 "Andover, NH", "Antrim, NH", "Ashland, NH", "Ashuelot, NH",
 "Atkinson, NH", "Auburn, NH", "Barnstead, NH", "Barrington, NH",
 "Bartlett, NH", "Bath, NH", "Bedford, NH", "Beebe River, NH",
 "Belmont, NH", "Bennington, NH", "Berlin, NH", "Bethlehem, NH",
 "Boscawen, NH", "Bow, NH", "Bradford, NH", "Brentwood, NH",
 "Bristol, NH", "Brookline, NH", "Canaan, NH", "Candia, NH",
 "Canterbury, NH", "Center Barnstead, NH", "Center Conway, NH",
 "Center Harbor, NH", "Center Ossipee, NH", "Center Sandwich, NH",
 "Center Strafford, NH", "Center Tuftonbor, NH", "Charlestown, NH",
 "Chester, NH", "Chesterfield, NH", "Chocorua, NH", "Claremont, NH",
 "Colebrook, NH", "Concord, NH", "Conway, NH", "Cornish, NH",
 "Danbury, NH", "Danville, NH", "Deerfield, NH", "Derry, NH",
 "Dublin, NH", "Dunbarton, NH", "East Andover, NH", "East Hampstead, NH",
 "East Hebron, NH", "East Lempster, NH", "East Rochester, NH",
 "East Sullivan, NH", "East Swanzey, NH", "East Wakefield, NH",
 "Enfield, NH", "Epping, NH", "Epsom, NH", "Errol, NH", "Etna, NH",
 "Farmington, NH", "Fitzwilliam, NH", "Francestown, NH", "Franconia, NH",
 "Franklin, NH", "Freedom, NH", "Fremont, NH", "Gilford, NH",
 "Gilmanton, NH", "Gilmanton Iron W, NH", "Gilsum, NH", "Glen, NH",
 "Gonic, NH", "Gorham, NH", "Goshen, NH", "Grafton, NH", "Grantham, NH",
 "Greenfield, NH", "Greenland, NH", "Groveton, NH", "Hampstead, NH",
 "Hampton, NH", "Hampton Falls, NH", "Hancock, NH", "Hanover, NH",
 "Harrisville, NH", "Haverhill, NH", "Hebron, NH", "Henniker, NH",
 "Hill, NH", "Hillsboro, NH", "Hinsdale, NH", "Hollis, NH",
 "Hooksett, NH", "Hopkinton, NH", "Hudson, NH", "Intervale, NH",
 "Jackson, NH", "Jaffrey, NH", "Jefferson, NH", "Kingston, NH",
 "Lancaster, NH", "Lebanon, NH", "Lee, NH", "Lincoln, NH", "Lisbon, NH",
 "Littleton, NH", "Londonderry, NH", "Lyme, NH", "Lyndeborough, NH",

"Madbury, NH", "Madison, NH", "Manchester, NH", "Marlborough, NH",
 "Marlow, NH", "Mason, NH", "Meredith, NH", "Meriden, NH",
 "Merrimack, NH", "Milan, NH", "Milford, NH", "Milton Mills, NH",
 "Mirror Lake, NH", "Monroe, NH", "Mont Vernon, NH",
 "Moultonborough, NH", "Munsonville, NH", "Nashua, NH", "New Boston, NH",
 "New Castle, NH", "New Durham, NH", "New Hampton, NH",
 "New Ipswich, NH", "New London, NH", "Newington, NH", "Newmarket, NH",
 "Newport, NH", "Newton, NH", "North Conway, NH", "North Hampton, NH",
 "North Haverhill, NH", "North Sandwich, NH", "North Stratford, NH",
 "North Walpole, NH", "North Woodstock, NH", "Northwood, NH",
 "Nottingham, NH", "Orford, NH", "Ossipee, NH", "Pelham, NH",
 "Peterborough, NH", "Piermont, NH", "Pike, NH", "Pittsburg, NH",
 "Pittsfield, NH", "Plainfield, NH", "Plaistow, NH", "Plymouth, NH",
 "Raymond, NH", "Richmond, NH", "Rindge, NH", "Rochester, NH",
 "Rollinsford, NH", "Rumney, NH", "Rye, NH", "Salem, NH",
 "Salisbury, NH", "Sanbornton, NH", "Sanbornville, NH", "Sandown, NH",
 "Seabrook, NH", "Silver Lake, NH", "Somersworth, NH",
 "South Acworth, NH", "South Effingham, NH", "South Hampton, NH",
 "South Tamworth, NH", "Spofford, NH", "Stoddard, NH", "Strafford, NH",
 "Stratham, NH", "Sunapee, NH", "Surry, NH", "Tamworth, NH",
 "Temple, NH", "Tilton, NH", "Troy, NH", "Union, NH", "Walpole, NH",
 "Warner, NH", "Warren, NH", "Washington, NH", "Weare, NH",
 "Wentworth, NH", "West Chesterfiel, NH", "West Lebanon, NH",
 "West Nottingham, NH", "West Ossipee, NH", "West Springfield, NH",
 "West Swanzey, NH", "Westmoreland, NH", "Whitefield, NH",
 "Wilmot Flat, NH", "Wilton, NH", "Windham, NH", "Wolfeboro, NH",
 "Woodsville, NH", "Allendale, NJ", "Allenhurst, NJ", "Allentown, NJ",
 "Alpha, NJ", "Alpine, NJ", "Andover, NJ", "Annandale, NJ",
 "Atlantic City, NJ", "Atlantic Highlan, NJ", "Audubon, NJ",
 "Augusta, NJ", "Avalon, NJ", "Avenel, NJ", "Avon By The Sea, NJ",
 "Barnegat, NJ", "Barrington, NJ", "Basking Ridge, NJ", "Batsto, NJ",
 "Bay Head, NJ", "Bayonne, NJ", "Bayville, NJ", "Beachwood, NJ",
 "Bedminster, NJ", "Belford, NJ", "Belle Mead, NJ", "Belleville, NJ",
 "Bellmawr, NJ", "Belvidere, NJ", "Bergenfield, NJ",
 "Berkeley Heights, NJ", "Berlin, NJ", "Bernardsville, NJ",
 "Beverly, NJ", "Blairstown, NJ", "Bloomfield, NJ", "Bloomington, NJ",
 "Bloomsbury, NJ", "Bogota, NJ", "Boonton, NJ", "Bordentown, NJ",
 "Bound Brook, NJ", "Bradley Beach, NJ", "Branchville, NJ", "Brick, NJ",
 "Bridgeport, NJ", "Bridgewater, NJ", "Brielle, NJ", "Brigantine, NJ",
 "Browns Mills, NJ", "Budd Lake, NJ", "Buena, NJ", "Burlington, NJ",
 "Califon, NJ", "Camden, NJ", "Cape May Court H, NJ", "Carlstadt, NJ",
 "Carneys Point, NJ", "Carteret, NJ", "Cedar Grove, NJ",
 "Cedar Knolls, NJ", "Cedarville, NJ", "Chatham, NJ", "Chatsworth, NJ",
 "Cherry Hill, NJ", "Chester, NJ", "Cinnaminson, NJ", "Clark, NJ",
 "Clarksboro, NJ", "Clarksburg, NJ", "Clayton, NJ", "Cliffside Park, NJ",
 "Cliffwood, NJ", "Clifton, NJ", "Clinton, NJ", "Closter, NJ",
 "Collingswood, NJ", "Colonia, NJ", "Colts Neck, NJ", "Columbia, NJ",
 "Columbus, NJ", "Cookstown, NJ", "Corbin City, NJ", "Cranbury, NJ",
 "Cranford, NJ", "Creamridge, NJ", "Cresskill, NJ", "Crosswicks, NJ",
 "Dayton, NJ", "Deal, NJ", "Delair, NJ", "Delanco, NJ", "Delmont, NJ",
 "Demarest, NJ", "Denville, NJ", "Deptford, NJ", "Dorothy, NJ",
 "Dumont, NJ", "East Brunswick, NJ", "East Hanover, NJ",
 "East Orange, NJ", "East Rutherford, NJ", "Eastampton Twp, NJ",
 "Eatontown, NJ", "Edgewater, NJ", "Edison, NJ", "Egg Harbor City, NJ",
 "Elizabeth, NJ", "Elmer, NJ", "Elmwood Park, NJ", "Emerson, NJ",

"Englewood, NJ", "Englewood Cliffs, NJ", "Essex Fells, NJ",
 "Estell Manor, NJ", "Fair Haven, NJ", "Fair Lawn, NJ", "Fairfield, NJ",
 "Fairview, NJ", "Fanwood, NJ", "Far Hills, NJ", "Farmingdale, NJ",
 "Flanders, NJ", "Flemington, NJ", "Florence, NJ", "Florham Park, NJ",
 "Fords, NJ", "Forked River, NJ", "Fort Dix, NJ", "Fort Hancock, NJ",
 "Fort Lee, NJ", "Fort Monmouth, NJ", "Franklin, NJ",
 "Franklin Lakes, NJ", "Franklin Park, NJ", "Franklinville, NJ",
 "Fredon Township, NJ", "Freehold, NJ", "Frenchtown, NJ", "Garfield, NJ",
 "Garwood, NJ", "Gibbsboro, NJ", "Gibbstown, NJ", "Gillette, NJ",
 "Gladstone, NJ", "Glassboro, NJ", "Glen Gardner, NJ", "Glen Ridge, NJ",
 "Glen Rock, NJ", "Glendora, NJ", "Glenwood, NJ", "Gloucester City, NJ",
 "Great Meadows, NJ", "Green Brook, NJ", "Green Village, NJ",
 "Greystone Park, NJ", "Guttenberg, NJ", "Hackensack, NJ",
 "Hackettstown, NJ", "Haddon Heights, NJ", "Haddonfield, NJ",
 "Haledon, NJ", "Hamburg, NJ", "Hamilton, NJ", "Hampton, NJ",
 "Harrington Park, NJ", "Harvey Cedars, NJ", "Hasbrouck Height, NJ",
 "Haskell, NJ", "Haworth, NJ", "Hawthorne, NJ", "Hazlet, NJ",
 "Heislerville, NJ", "Helmetta, NJ", "Hewitt, NJ", "High Bridge, NJ",
 "Highland Lakes, NJ", "Highland Park, NJ", "Hightstown, NJ",
 "Hillsdale, NJ", "Hillside, NJ", "Ho Ho Kus, NJ", "Hoboken, NJ",
 "Holmdel, NJ", "Hopatcong, NJ", "Hopewell, NJ", "Howell, NJ",
 "Imlaystown, NJ", "Irvington, NJ", "Iselin, NJ", "Island Heights, NJ",
 "Jackson, NJ", "Jamesburg, NJ", "Jersey City, NJ", "Jobstown, NJ",
 "Keansburg, NJ", "Kearny, NJ", "Keasbey, NJ", "Kendall Park, NJ",
 "Kenilworth, NJ", "Kenvil, NJ", "Keyport, NJ", "Kingston, NJ",
 "Kinnelon, NJ", "Lafayette, NJ", "Lake Hiawatha, NJ",
 "Lake Hopatcong, NJ", "Lakehurst Naec, NJ", "Lakewood, NJ",
 "Lambertville, NJ", "Landing, NJ", "Landisville, NJ",
 "Lanoka Harbor, NJ", "Laurel Springs, NJ", "Laurence Harbor, NJ",
 "Lavallette, NJ", "Lawnside, NJ", "Lawrenceville, NJ", "Layton, NJ",
 "Lebanon, NJ", "Ledgewood, NJ", "Leesburg, NJ", "Leonardo, NJ",
 "Leonia, NJ", "Lincoln Park, NJ", "Lincroft, NJ", "Linden, NJ",
 "Linwood, NJ", "Little Ferry, NJ", "Little Silver, NJ",
 "Livingston, NJ", "Lodi, NJ", "Long Branch, NJ", "Long Valley, NJ",
 "Longport, NJ", "Lumberton, NJ", "Lyndhurst, NJ", "Madison, NJ",
 "Magnolia, NJ", "Mahwah, NJ", "Malaga, NJ", "Manahawkin, NJ",
 "Manalapan, NJ", "Manasquan, NJ", "Mantoloking, NJ", "Mantua, NJ",
 "Manville, NJ", "Maple Shade, NJ", "Maplewood, NJ", "Margate City, NJ",
 "Marlboro, NJ", "Marlton, NJ", "Marmora, NJ", "Martinsville, NJ",
 "Matawan, NJ", "Mays Landing, NJ", "Maywood, NJ", "Mc Guire Afb, NJ",
 "Medford Lakes, NJ", "Mendham, NJ", "Mercerville, NJ",
 "Merchantville, NJ", "Metuchen, NJ", "Mickleton, NJ", "Middlesex, NJ",
 "Midland Park, NJ", "Milford, NJ", "Millburn, NJ", "Millington, NJ",
 "Milltown, NJ", "Millville, NJ", "Milmay, NJ", "Milton, NJ",
 "Mine Hill, NJ", "Minotola, NJ", "Monmouth Beach, NJ",
 "Monmouth Junctio, NJ", "Monroeville, NJ", "Montague, NJ",
 "Montclair, NJ", "Montvale, NJ", "Montville, NJ", "Moonachie, NJ",
 "Moorestown, NJ", "Morganville, NJ", "Morristown, NJ",
 "Mount Arlington, NJ", "Mount Ephraim, NJ", "Mount Laurel, NJ",
 "Mount Royal, NJ", "Mountain Lakes, NJ", "Mountainside, NJ",
 "Mullica Hill, NJ", "National Park, NJ", "Neptune City, NJ",
 "Neshanic Station, NJ", "Netcong, NJ", "New Brunswick, NJ",
 "New Egypt, NJ", "New Milford, NJ", "New Monmouth, NJ",
 "New Providence, NJ", "New Vernon, NJ", "Newark, NJ", "Newfield, NJ",
 "Newfoundland, NJ", "Newport, NJ", "Newtonville, NJ",

"North Arlington, NJ", "North Bergen, NJ", "North Branch, NJ",
 "North Brunswick, NJ", "North Cape May, NJ", "North Plainfield, NJ",
 "North Wildwood, NJ", "Northfield, NJ", "Norwood, NJ", "Nutley, NJ",
 "Oakhurst, NJ", "Oakland, NJ", "Oaklyn, NJ", "Ocean, NJ",
 "Ocean City, NJ", "Ocean Gate, NJ", "Ocean Grove, NJ", "Ocean View, NJ",
 "Oceanport, NJ", "Ogdensburg, NJ", "Old Bridge, NJ", "Old Tappan, NJ",
 "Oradell, NJ", "Orange, NJ", "Osbornsville, NJ", "Oxford, NJ",
 "Palisades Park, NJ", "Palmyra, NJ", "Paramus, NJ", "Park Ridge, NJ",
 "Parlin, NJ", "Parsippany, NJ", "Passaic, NJ", "Paterson, NJ",
 "Pattenburg, NJ", "Paulsboro, NJ", "Pedricktown, NJ", "Pemberton, NJ",
 "Pennington, NJ", "Pennsville, NJ", "Pequannock, NJ",
 "Perrineville, NJ", "Perth Amboy, NJ", "Pine Beach, NJ",
 "Pine Brook, NJ", "Piscataway, NJ", "Pitman, NJ", "Pittstown, NJ",
 "Plainsboro, NJ", "Pleasantville, NJ", "Pompton Lakes, NJ",
 "Pompton Plains, NJ", "Port Monmouth, NJ", "Port Murray, NJ",
 "Port Norris, NJ", "Port Reading, NJ", "Port Republic, NJ",
 "Princeton, NJ", "Princeton Juncti, NJ", "Princeton Univer, NJ",
 "Rahway, NJ", "Ramsey, NJ", "Randolph, NJ", "Raritan, NJ",
 "Richland, NJ", "Ridgefield, NJ", "Ridgefield Park, NJ",
 "Ridgewood, NJ", "Ringoes, NJ", "Ringwood, NJ", "Rio Grande, NJ",
 "River Edge, NJ", "Riverdale, NJ", "Rockaway, NJ", "Rockleigh, NJ",
 "Rocky Hill, NJ", "Roebling, NJ", "Roseland, NJ", "Roselle, NJ",
 "Roselle Park, NJ", "Rosemont, NJ", "Runnemede, NJ", "Rutherford, NJ",
 "Saddle Brook, NJ", "Salem, NJ", "Sayreville, NJ", "Scotch Plains, NJ",
 "Sea Bright, NJ", "Sea Girt, NJ", "Seabrook, NJ", "Seaside Heights, NJ",
 "Seaside Park, NJ", "Secaucus, NJ", "Sewaren, NJ", "Sewell, NJ",
 "Short Hills, NJ", "Shrewsbury, NJ", "Sicklerville, NJ", "Skillman, NJ",
 "Smithville, NJ", "Somerdale, NJ", "Somers Point, NJ", "Somerset, NJ",
 "South Bound Broo, NJ", "South Hackensack, NJ", "South Orange, NJ",
 "South Plainfield, NJ", "South River, NJ", "Southampton, NJ",
 "Sparta, NJ", "Spotswood, NJ", "Spring Lake, NJ", "Springfield, NJ",
 "Stanhope, NJ", "Stewartsville, NJ", "Stirling, NJ", "Stockholm, NJ",
 "Stockton, NJ", "Stone Harbor, NJ", "Stratford, NJ", "Strathmere, NJ",
 "Suburban, NJ", "Succasunna, NJ", "Summit, NJ", "Sussex, NJ",
 "Swedesboro, NJ", "Teaneck, NJ", "Tenafly, NJ", "Teterboro, NJ",
 "Thorofare, NJ", "Three Bridges, NJ", "Titusville, NJ",
 "Toms River, NJ", "Totowa, NJ", "Towaco, NJ", "Townsend's Inlet, NJ",
 "Trenton, NJ", "Tuckerton, NJ", "Union, NJ", "Upper Saddle Riv, NJ",
 "Vauxhall, NJ", "Ventnor City, NJ", "Vernon, NJ", "Verona, NJ",
 "Villas, NJ", "Vineland, NJ", "Voorhees, NJ", "Waldwick, NJ",
 "Wall, NJ", "Wallington, NJ", "Wallpack Center, NJ", "Wanaque, NJ",
 "Waretown, NJ", "Warren, NJ", "Washington, NJ", "Waterford Works, NJ",
 "Wayne, NJ", "Weehawken, NJ", "Wenonah, NJ", "West Berlin, NJ",
 "West Caldwell, NJ", "West Creek, NJ", "West Long Branch, NJ",
 "West Milford, NJ", "West Orange, NJ", "West Paterson, NJ",
 "West Trenton, NJ", "Westfield, NJ", "Westville, NJ", "Wharton, NJ",
 "Whippany, NJ", "Whitehouse Stati, NJ", "Whiting, NJ",
 "Williamstown, NJ", "Willingboro, NJ", "Winslow, NJ", "Wood Ridge, NJ",
 "Woodbridge, NJ", "Woodbury Heights, NJ", "Woodstown, NJ",
 "Wrightstown, NJ", "Wyckoff, NJ", "Yardville, NJ", "Abiquiu, NM",
 "Alameda, NM", "Alamo, NM", "Alamogordo, NM", "Albert, NM",
 "Albuquerque, NM", "Algodones, NM", "Amistad, NM", "Animas, NM",
 "Anton Chico, NM", "Aragon, NM", "Arrey, NM", "Arroyo Hondo, NM",
 "Arroyo Seco, NM", "Artesia, NM", "Aztec, NM", "Bard, NM",
 "Bell Ranch, NM", "Bent, NM", "Bernalillo, NM", "Bingham, NM",

"Blanco, NM", "Bloomfield, NM", "Bluewater, NM", "Bosque, NM",
"Bosque Farms, NM", "Boys Ranch, NM", "Brimhall, NM", "Broadview, NM",
"Buckhorn, NM", "Bueyeros, NM", "Caballo, NM", "Canoncito, NM",
"Capitan, NM", "Caprock, NM", "Capulin, NM", "Carlsbad, NM",
"Carrizozo, NM", "Casa Blanca, NM", "Causey, NM", "Cedar Crest, NM",
"Cedarvale, NM", "Central, NM", "Cerrillos, NM", "Chacon, NM",
"Chama, NM", "Chamisal, NM", "Chaparral, NM", "Cimarron, NM",
"Clayton, NM", "Cleveland, NM", "Cloudcroft, NM", "Clovis, NM",
"Cochiti Pueblo, NM", "Conchas Dam, NM", "Continental Divi, NM",
"Corona, NM", "Corrales, NM", "Costilla, NM", "Counselor, NM",
"Crossroads, NM", "Crownpoint, NM", "Cuba, NM", "Cubero, NM",
"Cuchillo, NM", "Cuervo, NM", "Cundiyo, NM", "Datil, NM", "Deming, NM",
"Derry, NM", "Des Moines, NM", "Dexter, NM", "Dixon, NM", "Dulce, NM",
"Eagle Nest, NM", "Edgewood, NM", "El Rito, NM", "Elida, NM",
"Embudo, NM", "Encino, NM", "Espanola, NM", "Estancia, NM",
"Eunice, NM", "Farmington, NM", "Fence Lake, NM", "Flora Vista, NM",
"Floyd, NM", "Folsom, NM", "Fort Sumner, NM", "Fruitland, NM",
"Gallina, NM", "Gallup, NM", "Garfield, NM", "Garita, NM",
"Gladstone, NM", "Glencoe, NM", "Glenwood, NM", "Glorieta, NM",
"Grady, NM", "Grants, NM", "Grenville, NM", "Guadalupita, NM",
"Hagerman, NM", "Hatch, NM", "Hernandez, NM", "Hillsboro, NM",
"Hobbs, NM", "Holloman Air For, NM", "Hondo, NM", "Hope, NM",
"House, NM", "Hurley, NM", "Jal, NM", "Jarales, NM", "Jemez Pueblo, NM",
"Jemez Springs, NM", "Kirtland, NM", "Kirtland A F B E, NM",
"La Jara, NM", "La Loma, NM", "La Luz, NM", "La Madera, NM",
"La Mesa, NM", "La Plata, NM", "Lajoya, NM", "Lake Arthur, NM",
"Lamy, NM", "Las Cruces, NM", "Las Vegas, NM", "Ledoux, NM",
"Lemitar, NM", "Lincoln, NM", "Lindrith, NM", "Lingo, NM", "Logan, NM",
"Los Alamos, NM", "Los Lunas, NM", "Loving, NM", "Lovington, NM",
"Maljamar, NM", "Maxwell, NM", "Mayhill, NM", "Mc Alister, NM",
"Melrose, NM", "Mescalero, NM", "Mesilla Park, NM", "Mesquite, NM",
"Mexican Springs, NM", "Miami, NM", "Mills, NM", "Milnesand, NM",
"Mimbres, NM", "Montezuma, NM", "Monument, NM", "Mora, NM",
"Moriarty, NM", "Mount Dora, NM", "Mountainair, NM", "Nara Visa, NM",
"Navajo, NM", "Navajo Dam, NM", "Newkirk, NM", "Nogal, NM", "Ocate, NM",
"Oil Center, NM", "Ojo Caliente, NM", "Pastura, NM", "Pecos, NM",
"Penasco, NM", "Pep, NM", "Peralta, NM", "Picacho, NM", "Pie Town, NM",
"Pinon, NM", "Placitas, NM", "Pojoaque Valley, NM", "Polvadera, NM",
"Ponderosa, NM", "Portales, NM", "Prewitt, NM", "Puerto De Luna, NM",
"Quay, NM", "Quemado, NM", "Questa, NM", "Ramah, NM",
"Ranchos De Taos, NM", "Raton, NM", "Regina, NM", "Reserve, NM",
"Ribera, NM", "Rincon, NM", "Rio Rancho, NM", "Road Forks, NM",
"Rociada, NM", "Rogers, NM", "Roswell, NM", "Roy, NM", "Ruidoso, NM",
"Ruidoso Downs, NM", "Rutherford, NM", "Sacramento, NM",
"Saint Vrain, NM", "Salem, NM", "San Acacia, NM", "San Cristobal, NM",
"San Jon, NM", "San Jose, NM", "San Juan Pueblo, NM", "San Lorenzo, NM",
"San Mateo, NM", "San Patricio, NM", "Sandia Park, NM",
"Santa Cruz, NM", "Santa Fe, NM", "Santo Domingo Pu, NM", "Sapello, NM",
"Seboyeta, NM", "Sedan, NM", "Seneca, NM", "Shiprock, NM",
"Silver City, NM", "Socorro, NM", "Solano, NM", "Springer, NM",
"Stanley, NM", "Stead, NM", "Taiban, NM", "Taos, NM", "Tatum, NM",
"Tererro, NM", "Texico, NM", "Thoreau, NM", "Tierra Amarilla, NM",
"Tijeras, NM", "Tinnie, NM", "Toadlena, NM", "Tohatchi, NM",
"Trementina, NM", "Truth Or Consequ, NM", "Tucumcari, NM",
"Tularosa, NM", "Vadito, NM", "Valdez, NM", "Vallecitos, NM",

"Valmora, NM", "Vanadium, NM", "Vaughn, NM", "Veguita, NM",
 "Wagon Mound, NM", "Waterflow, NM", "Weed, NM", "White Sands Miss, NM",
 "Willard, NM", "Williamsburg, NM", "Winston, NM", "Yeso, NM",
 "Zia Pueblo, NM", "Zuni, NM", "Alamo, NV", "Amargosa Valley, NV",
 "Austin, NV", "Baker, NV", "Battle Mountain, NV", "Beowawe, NV",
 "Boulder City, NV", "Caliente, NV", "Carlin, NV", "Carson City, NV",
 "Cottonwood Cove, NV", "Dayton, NV", "Deeth, NV", "Denio, NV",
 "Ely, NV", "Empire, NV", "Eureka, NV", "Fallon, NV", "Fernley, NV",
 "Gabbs, NV", "Gardnerville, NV", "Gerlach, NV", "Glenbrook, NV",
 "Golconda, NV", "Goldfield, NV", "Goodsprings, NV", "Hawthorne, NV",
 "Henderson, NV", "Hiko, NV", "Incline Village, NV",
 "Indian Springs, NV", "Jackpot, NV", "Jiggs, NV", "Las Vegas, NV",
 "Laughlin, NV", "Lovelock, NV", "Lund, NV", "Luning, NV", "Minden, NV",
 "Moundhouse, NV", "Mountain City, NV", "Nixon, NV",
 "North Las Vegas, NV", "Oasis, NV", "Orovada, NV", "Overton, NV",
 "Pahrump, NV", "Paradise Valley, NV", "Pioche, NV", "Reno, NV",
 "Round Mountain, NV", "Ruby Valley, NV", "Schurz, NV",
 "Silver Springs, NV", "Silverpeak, NV", "Smith, NV", "Sparks, NV",
 "Sun Valley, NV", "Tonopah, NV", "Tuscarora, NV", "Unionville, NV",
 "Virginia City, NV", "Wadsworth, NV", "Wellington, NV",
 "Winnemucca, NV", "Yerington, NV", "Accord, NY", "Acra, NY",
 "Adams Basin, NY", "Adams Center, NY", "Addison, NY", "Adirondack, NY",
 "Afton, NY", "Akron, NY", "Alabama, NY", "Albany, NY", "Albertson, NY",
 "Albion, NY", "Alcove, NY", "Alden, NY", "Alden Manor, NY",
 "Alder Creek, NY", "Alexander, NY", "Alfred, NY", "Alfred Station, NY",
 "Allegany, NY", "Alma, NY", "Almond, NY", "Alpine, NY", "Alplaus, NY",
 "Altamont, NY", "Altmar, NY", "Altona, NY", "Amawalk, NY", "Amenia, NY",
 "Ames, NY", "Amherst, NY", "Amityville, NY", "Ancram, NY",
 "Ancramdale, NY", "Andes, NY", "Andover, NY", "Angelica, NY",
 "Angola, NY", "Antwerp, NY", "Apalachin, NY", "Appleton, NY",
 "Arcade, NY", "Ardsley, NY", "Argyle, NY", "Arkport, NY",
 "Arkville, NY", "Arlington, NY", "Armonk, NY", "Ashland, NY",
 "Ashville, NY", "Astoria, NY", "Athens, NY", "Athol, NY", "Atlanta, NY",
 "Atlantic Beach, NY", "Attica, NY", "Au Sable Chasm, NY",
 "Au Sable Forks, NY", "Auburn, NY", "Aurora, NY", "Austerlitz, NY",
 "Ava, NY", "Averill Park, NY", "Avon, NY", "Bainbridge, NY",
 "Baldwin, NY", "Baldwinsville, NY", "Ballston Lake, NY",
 "Ballston Spa, NY", "Bangor, NY", "Bardonia, NY", "Barker, NY",
 "Barneveld, NY", "Barrytown, NY", "Barryville, NY", "Barton, NY",
 "Basom, NY", "Batavia, NY", "Bayberry, NY", "Bayport, NY",
 "Bayside, NY", "Bayville, NY", "Beacon, NY", "Bear Mountain, NY",
 "Beaver Dams, NY", "Beaver River, NY", "Bedford, NY",
 "Bedford Hills, NY", "Belfast, NY", "Bellerose, NY", "Belleville, NY",
 "Bellona, NY", "Bellport, NY", "Belmont, NY", "Bemus Point, NY",
 "Bergen, NY", "Berkshire, NY", "Berlin, NY", "Berne, NY",
 "Bernhards Bay, NY", "Bethel, NY", "Bethpage, NY", "Big Flats, NY",
 "Binghamton, NY", "Black Creek, NY", "Black River, NY", "Blasdell, NY",
 "Blauvelt, NY", "Bliss, NY", "Bloomfield, NY", "Bloomington, NY",
 "Bloomville, NY", "Blossvale, NY", "Blue Mountain La, NY", "Blue Point, NY",
 "Bluff Point, NY", "Bohemia, NY", "Boiceville, NY", "Bolivar, NY",
 "Bolton Landing, NY", "Bombay, NY", "Boonville, NY", "Boston, NY",
 "Bouckville, NY", "Bovina Center, NY", "Bowmansville, NY",
 "Bradford, NY", "Brainard, NY", "Branchport, NY", "Brant Lake, NY",
 "Brasher Falls, NY", "Breesport, NY", "Brewerton, NY", "Brewster, NY",

"Briarcliff Manor, NY", "Bridgeport, NY", "Brier Hill, NY",
 "Brightwaters, NY", "Brisben, NY", "Broadalbin, NY", "Brockport, NY",
 "Brocton, NY", "Bronx, NY", "Bronxville, NY", "Brookfield, NY",
 "Brookhaven, NY", "Brooklyn, NY", "Brooklyn Navy Ya, NY",
 "Brooktondale, NY", "Brushton, NY", "Buchanan, NY", "Buffalo, NY",
 "Burdett, NY", "Burke, NY", "Burlington Flats, NY", "Burnt Hills, NY",
 "Burt, NY", "Buskirk, NY", "Byron, NY", "Cadosia, NY", "Cadyville, NY",
 "Cairo, NY", "Calcium, NY", "Caledonia, NY", "Callicoon, NY",
 "Calverton, NY", "Cambria Heights, NY", "Cambridge, NY", "Camden, NY",
 "Cameron, NY", "Cameron Mills, NY", "Camillus, NY", "Campbell, NY",
 "Campbell Hall, NY", "Canaan, NY", "Canandaigua, NY", "Canaseraga, NY",
 "Canastota, NY", "Candor, NY", "Caneadea, NY", "Canisteo, NY",
 "Canton, NY", "Cape Vincent, NY", "Carle Place, NY", "Carlisle, NY",
 "Carmel, NY", "Caroga Lake, NY", "Carthage, NY", "Cassadaga, NY",
 "Cassville, NY", "Castile, NY", "Castle Creek, NY",
 "Castleton On Hud, NY", "Castorland, NY", "Cato, NY", "Catskill, NY",
 "Cattaraugus, NY", "Cayuga, NY", "Cayuta, NY", "Cazenovia, NY",
 "Cedarhurst, NY", "Center Moriches, NY", "Centereach, NY",
 "Centerport, NY", "Central Bridge, NY", "Central Islip, NY",
 "Central Square, NY", "Central Valley, NY", "Ceres, NY",
 "Chadwicks, NY", "Chaffee, NY", "Champlain, NY", "Chappaqua, NY",
 "Charlotteville, NY", "Chase Mills, NY", "Chateaugay, NY",
 "Chatham, NY", "Chaumont, NY", "Chazy, NY", "Cheektowaga, NY",
 "Chemung, NY", "Chenango Forks, NY", "Cherry Creek, NY",
 "Cherry Grove, NY", "Cherry Valley, NY", "Chester, NY",
 "Chestertown, NY", "Chestnut Ridge, NY", "Chichester, NY",
 "Childwold, NY", "Chittenango, NY", "Churubusco, NY", "Cicero, NY",
 "Cincinnatus, NY", "Circleville, NY", "Clarence, NY",
 "Clarence Center, NY", "Clarksville, NY", "Claryville, NY",
 "Claverack, NY", "Clay, NY", "Clayville, NY", "Clemons, NY",
 "Cleveland, NY", "Clifton, NY", "Clifton Park, NY",
 "Clifton Springs, NY", "Climax, NY", "Clinton, NY",
 "Clinton Corners, NY", "Clintondale, NY", "Clyde, NY", "Clymer, NY",
 "Cobleskill, NY", "Cochecton Center, NY", "Coeymans Hollow, NY",
 "Cohocton, NY", "Cohoes, NY", "Cold Brook, NY", "Cold Spring, NY",
 "Cold Spring Harb, NY", "Colden, NY", "College Point, NY",
 "Collins, NY", "Colton, NY", "Commack, NY", "Comstock, NY",
 "Conesus, NY", "Conewango Valley, NY", "Congers, NY", "Conklin, NY",
 "Constable, NY", "Constableville, NY", "Constantia, NY",
 "Cook Falls, NY", "Cooperstown, NY", "Copake, NY", "Copake Falls, NY",
 "Copenhagen, NY", "Copiague, NY", "Coram, NY", "Corfu, NY",
 "Corinth, NY", "Corning, NY", "Cornwall, NY", "Cornwall On Huds, NY",
 "Cornwallville, NY", "Corona, NY", "Cortland, NY",
 "Cortlandt Manor, NY", "Cossayuna, NY", "Cottekill, NY",
 "Cowlesville, NY", "Coxsackie, NY", "Crarryville, NY", "Croghan, NY",
 "Cropseyville, NY", "Cross River, NY", "Croton On Hudson, NY",
 "Crown Point, NY", "Cuba, NY", "Cuddebackville, NY", "Cutchogue, NY",
 "Cuyler, NY", "Dale, NY", "Dalton, NY", "Dansville, NY",
 "Darien Center, NY", "Davenport, NY", "Davenport Center, NY",
 "Davis Park, NY", "Dayton, NY", "De Kalb Junction, NY", "De Lancey, NY",
 "De Peyster, NY", "De Ruyter, NY", "De Witt, NY", "Deansboro, NY",
 "Deer Park, NY", "Degrasse, NY", "Delanson, NY", "Delevan, NY",
 "Delmar, NY", "Denver, NY", "Depew, NY", "Deposit, NY", "Derby, NY",
 "Dewittville, NY", "Dexter, NY", "Diamond Point, NY",
 "Dickinson Center, NY", "Dix Hills, NY", "Dobbs Ferry, NY",

"Dolgeville, NY", "Dormansville, NY", "Dover Plains, NY",
"Downsville, NY", "Dresden, NY", "Dryden, NY", "Duaneburg, NY",
"Dundee, NY", "Durham, NY", "Durhamville, NY", "Eagle Bay, NY",
"Earlton, NY", "Earlville, NY", "East Aurora, NY", "East Berne, NY",
"East Bethany, NY", "East Branch, NY", "East Chatham, NY",
"East Concord, NY", "East Durham, NY", "East Elmhurst, NY",
"East Freetown, NY", "East Greenbush, NY", "East Greenwich, NY",
"East Hampton, NY", "East Islip, NY", "East Jewett, NY",
"East Marion, NY", "East Meadow, NY", "East Meredith, NY",
"East Moriches, NY", "East Nassau, NY", "East Norwich, NY",
"East Otto, NY", "East Quogue, NY", "East Rochester, NY",
"East Rockaway, NY", "East Springfield, NY", "East Syracuse, NY",
"East White Plain, NY", "East Windham, NY", "East Worcester, NY",
"Eastchester, NY", "Eastport, NY", "Eaton, NY", "Eddyville, NY",
"Eden, NY", "Edinburg, NY", "Edmeston, NY", "Edwards, NY", "Elba, NY",
"Elbridge, NY", "Eldred, NY", "Elizabethtown, NY", "Elizaville, NY",
"Elka Park, NY", "Ellenburg Center, NY", "Ellenburg Depot, NY",
"Ellenville, NY", "Ellicottville, NY", "Ellisburg, NY", "Elma, NY",
"Elmira, NY", "Elmira Heights, NY", "Elmsford, NY", "Elwood, NY",
"Endwell, NY", "Erieville, NY", "Erin, NY", "Esperance, NY",
"Essex, NY", "Evans Mills, NY", "Fabius, NY", "Fairport, NY",
"Falconer, NY", "Fallsburg, NY", "Far Rockaway, NY",
"Farmersville Sta, NY", "Farmingville, NY", "Fayetteville, NY",
"Felts Mills, NY", "Feura Bush, NY", "Fillmore, NY", "Findley Lake, NY",
"Fishers Island, NY", "Fishkill, NY", "Floral Park, NY", "Florida, NY",
"Flushing, NY", "Fly Creek, NY", "Fonda, NY", "Forest Hills, NY",
"Forestburgh, NY", "Forestport, NY", "Forestville, NY", "Fort Ann, NY",
"Fort Covington, NY", "Fort Drum, NY", "Fort Edward, NY",
"Fort Johnson, NY", "Fort Plain, NY", "Fort Totten, NY",
"Fosterdale, NY", "Frankfort, NY", "Franklin, NY",
"Franklin Square, NY", "Franklinville, NY", "Fredonia, NY",
"Freedom, NY", "Freehold, NY", "Freeport, NY", "Freeville, NY",
"Fremont Center, NY", "Fresh Meadows, NY", "Frewsburg, NY",
"Friendship, NY", "Frontenac, NY", "Fulton, NY", "Fultonham, NY",
"Fultonville, NY", "Gainesville, NY", "Galway, NY", "Gansevoort, NY",
"Garden City, NY", "Gardiner, NY", "Garnerville, NY",
"Garrattsville, NY", "Garrison, NY", "Gasport, NY", "Geneseo, NY",
"Geneva, NY", "Genoa, NY", "Georgetown, NY", "Germantown, NY",
"Gerry, NY", "Getzville, NY", "Ghent, NY", "Gilbertsville, NY",
"Gilboa, NY", "Glen Aubrey, NY", "Glen Cove, NY", "Glen Head, NY",
"Glen Oaks, NY", "Glen Spey, NY", "Glen Wild, NY", "Glenfield, NY",
"Glenford, NY", "Glenmont, NY", "Glenwood, NY", "Gloversville, NY",
"Godeffroy, NY", "Goshen, NY", "Gouverneur, NY", "Governors Island, NY",
"Gowanda, NY", "Grahamsville, NY", "Grand Gorge, NY",
"Grand Island, NY", "Granite Springs, NY", "Granville, NY",
"Great Neck, NY", "Great Valley, NY", "Greece, NY", "Green Island, NY",
"Greene, NY", "Greenfield Cente, NY", "Greenfield Park, NY",
"Greenlawn, NY", "Greenport, NY", "Greenvale, NY", "Greenville, NY",
"Greenwood, NY", "Greenwood Lake, NY", "Greig, NY", "Grossinger, NY",
"Groton, NY", "Groveland, NY", "Guilderland, NY", "Guilford, NY",
"Hadley, NY", "Hagaman, NY", "Hague, NY", "Halcott Center, NY",
"Halesite, NY", "Hamburg, NY", "Hamden, NY", "Hamilton, NY",
"Hamlin, NY", "Hammond, NY", "Hammondsport, NY", "Hampton, NY",
"Hampton Bays, NY", "Hannacroix, NY", "Hannibal, NY",
"Harpersfield, NY", "Harpursville, NY", "Harriman, NY", "Harris, NY",

"Harrison, NY", "Harrisville, NY", "Hartford, NY", "Hartsdale, NY",
 "Hartwick, NY", "Hastings, NY", "Hastings On Huds, NY", "Hauppauge, NY",
 "Haverstraw, NY", "Hawthorne, NY", "Hayt Corners, NY", "Heathcote, NY",
 "Hector, NY", "Hemlock, NY", "Hempstead, NY", "Henderson, NY",
 "Henrietta, NY", "Herkimer, NY", "Hermon, NY", "Heuvelton, NY",
 "Hewlett, NY", "Hicksville, NY", "High Falls, NY", "Highland, NY",
 "Highland Falls, NY", "Highland Lake, NY", "Highland Mills, NY",
 "Hillburn, NY", "Hillsdale, NY", "Hillside Manor, NY", "Hilton, NY",
 "Himrod, NY", "Hobart, NY", "Hoffmeister, NY", "Hogansburg, NY",
 "Holbrook, NY", "Holland, NY", "Holland Patent, NY", "Hollis, NY",
 "Holmes, NY", "Holtsville, NY", "Homer, NY", "Honeoye, NY",
 "Honeoye Falls, NY", "Hoosick Falls, NY", "Hopewell Junction, NY",
 "Hornell, NY", "Horseheads, NY", "Hortonville, NY", "Houghton, NY",
 "Howes Cave, NY", "Hubbardsville, NY", "Hudson, NY", "Hudson Falls, NY",
 "Huguenot, NY", "Hulberton, NY", "Hunt, NY", "Hunter, NY", "Hurley, NY",
 "Hurleyville, NY", "Hyde Park, NY", "Ilion, NY", "Indian Lake, NY",
 "Inlet, NY", "Interlaken, NY", "Inwood, NY", "Ionia, NY", "Irving, NY",
 "Irvington, NY", "Ischua, NY", "Island Park, NY", "Islip, NY",
 "Islip Terrace, NY", "Ithaca, NY", "Ithaca College, NY",
 "Jackson Heights, NY", "Jamaica, NY", "Jamestown, NY", "Jamesville, NY",
 "Jasper, NY", "Java Center, NY", "Java Village, NY", "Jay, NY",
 "Jefferson, NY", "Jefferson Valley, NY", "Jeffersonville, NY",
 "Jericho, NY", "Jewett, NY", "Johnsburg, NY", "Johnson City, NY",
 "Johnsonville, NY", "Johnstown, NY", "Jordan, NY", "Jordanville, NY",
 "Katonah, NY", "Keene, NY", "Keeseville, NY", "Kelly Corners, NY",
 "Kendall, NY", "Kenmore, NY", "Kennedy, NY", "Kenoza Lake, NY",
 "Kent, NY", "Kerhonkson, NY", "Kew Gardens, NY", "Kiamesha Lake, NY",
 "Kill Buck, NY", "Kinderhook, NY", "King Ferry, NY", "Kings Park, NY",
 "Kings Point Cont, NY", "Kirkville, NY", "Kirkwood, NY", "Kismet, NY",
 "Krumville, NY", "La Fargeville, NY", "La Fayette, NY",
 "Lackawanna, NY", "Lacona, NY", "Lagrangeville, NY", "Lake George, NY",
 "Lake Grove, NY", "Lake Hill, NY", "Lake Huntington, NY",
 "Lake Katrine, NY", "Lake Luzerne, NY", "Lake Peekskill, NY",
 "Lake Pleasant, NY", "Lake Ronkonkoma, NY", "Lake View, NY",
 "Lakeville, NY", "Lakewood, NY", "Lancaster, NY", "Lanesville, NY",
 "Lansing, NY", "Larchmont, NY", "Latham, NY", "Laurel, NY",
 "Laurens, NY", "Lawrence, NY", "Lawrenceville, NY", "Lawtons, NY",
 "Lawyersville, NY", "Le Roy, NY", "Lebanon, NY", "Lee Center, NY",
 "Leeds, NY", "Leicester, NY", "Levittown, NY", "Lew Beach, NY",
 "Lewis, NY", "Lewiston, NY", "Liberty, NY", "Lima, NY", "Limestone, NY",
 "Lindenhurst, NY", "Lindley, NY", "Linwood, NY", "Lisbon, NY",
 "Lisle, NY", "Little Falls, NY", "Little Genesee, NY",
 "Little Neck, NY", "Little Valley, NY", "Liverpool, NY",
 "Livingston Manor, NY", "Livonia, NY", "Loch Sheldrake, NY",
 "Locke, NY", "Lockport, NY", "Lockwood, NY", "Locust Valley, NY",
 "Lodi, NY", "Long Beach, NY", "Long Eddy, NY", "Long Lake, NY",
 "Lorraine, NY", "Loudonville, NY", "Lowman, NY", "Lynbrook, NY",
 "Lyndonville, NY", "Lyon Mountain, NY", "Lyons, NY", "Lyons Falls, NY",
 "Mac Dougall, NY", "Macedon, NY", "Machias, NY", "Madison, NY",
 "Madrid, NY", "Mahopac, NY", "Maine, NY", "Malden Bridge, NY",
 "Mallory, NY", "Malone, NY", "Malverne, NY", "Mamaroneck, NY",
 "Manchester, NY", "Manlius, NY", "Mannsville, NY", "Manorville, NY",
 "Maplecrest, NY", "Marathon, NY", "Marcellus, NY", "Marcy, NY",
 "Marietta, NY", "Marilla, NY", "Marion, NY", "Marlboro, NY",
 "Martville, NY", "Maryland, NY", "Masonville, NY", "Maspeth, NY",

"Massapequa Park, NY", "Massena, NY", "Mastic, NY", "Mastic Beach, NY",
"Mattituck, NY", "Mattydale, NY", "Maybrook, NY", "Mayfair, NY",
"Mayfield, NY", "Mayville, NY", "Mc Connellsville, NY",
"Mc Donough, NY", "Mc Graw, NY", "Mc Kownville, NY",
"Mechanicville, NY", "Medford, NY", "Medina, NY", "Medusa, NY",
"Melrose, NY", "Melville, NY", "Memphis, NY", "Mendon, NY",
"Meredith, NY", "Meridale, NY", "Merrill, NY", "Mexico, NY",
"Middle Granville, NY", "Middle Grove, NY", "Middle Hope, NY",
"Middle Island, NY", "Middle Village, NY", "Middleburgh, NY",
"Middleport, NY", "Middlesex, NY", "Middleville, NY", "Mileses, NY",
"Milford, NY", "Mill Neck, NY", "Millbrook, NY", "Miller Place, NY",
"Millerton, NY", "Millport, NY", "Millwood, NY", "Milton, NY",
"Mineola, NY", "Minerva, NY", "Mineville, NY", "Minoa, NY",
"Modena, NY", "Mohawk, NY", "Mohegan Lake, NY", "Mohonk Lake, NY",
"Moir, NY", "Mongaup Valley, NY", "Monroe, NY", "Monsey, NY",
"Montauk, NY", "Montgomery, NY", "Monticello, NY", "Montour Falls, NY",
"Montrose, NY", "Mooers, NY", "Mooers Forks, NY", "Moravia, NY",
"Moriah, NY", "Moriah Center, NY", "Moriches, NY", "Morris, NY",
"Morrisonville, NY", "Morrisville, NY", "Mount Kisco, NY",
"Mount Marion, NY", "Mount Sinai, NY", "Mount Tremper, NY",
"Mount Upton, NY", "Mount Vernon, NY", "Mount Vision, NY",
"Mountain Dale, NY", "Munnsville, NY", "Napanoch, NY", "Naples, NY",
"Narrowsburg, NY", "Nassau, NY", "Natural Bridge, NY", "Nedrow, NY",
"Nesconset, NY", "Neversink, NY", "New Berlin, NY", "New City, NY",
"New Hamburg, NY", "New Hampton, NY", "New Hartford, NY",
"New Hyde Park, NY", "New Lebanon, NY", "New Lisbon, NY",
"New Rochelle, NY", "New Russia, NY", "New Windsor, NY",
"New Woodstock, NY", "New York, NY", "New York Mills, NY", "Newark, NY",
"Newark Valley, NY", "Newcomb, NY", "Newfane, NY", "Newfield, NY",
"Newport, NY", "Newton Falls, NY", "Niagara Falls, NY", "Nichols, NY",
"Nicholville, NY", "Nineveh, NY", "Niskayuna, NY", "Niverville, NY",
"Norfolk, NY", "North Babylon, NY", "North Bellmore, NY",
"North Blenheim, NY", "North Branch, NY", "North Brookfield, NY",
"North Chili, NY", "North Collins, NY", "North Creek, NY",
"North Granville, NY", "North Hudson, NY", "North Java, NY",
"North Lawrence, NY", "North Massapequa, NY", "North Merrick, NY",
"North Pitcher, NY", "North Pole, NY", "North Rose, NY",
"North Salem, NY", "North Syracuse, NY", "North Tarrytown, NY",
"North Tonawanda, NY", "North Woodmere, NY", "Northport, NY",
"Norton Hill, NY", "Norwich, NY", "Norwood, NY", "Nunda, NY",
"Nyack, NY", "Oak Beach, NY", "Oak Hill, NY", "Oakdale, NY",
"Oakfield, NY", "Oceanside, NY", "Odessa, NY", "Ogdensburg, NY",
"Old Bethpage, NY", "Old Chatham, NY", "Old Forge, NY",
"Old Westbury, NY", "Olean, NY", "Oliverea, NY", "Olmstedville, NY",
"Onchiota, NY", "Oneida, NY", "Oneonta, NY", "Onondaga, NY",
"Ontario, NY", "Orangeburg, NY", "Orchard Park, NY", "Orient, NY",
"Oriskany, NY", "Oriskany Falls, NY", "Ossining, NY", "Oswegatchie, NY",
"Oswego, NY", "Otego, NY", "Otisville, NY", "Ouaquaga, NY", "Owego, NY",
"Owls Head, NY", "Oyster Bay, NY", "Ozone Park, NY", "Painted Post, NY",
"Palatine Bridge, NY", "Palenville, NY", "Palisades, NY", "Palmyra, NY",
"Panama, NY", "Panorama, NY", "Paradox, NY", "Parish, NY",
"Parishville, NY", "Parksville, NY", "Patterson, NY",
"Pattersonville, NY", "Paul Smiths, NY", "Pawling, NY",
"Pearl River, NY", "Pelham, NY", "Penfield, NY", "Penn Yan, NY",
"Pennellville, NY", "Perry, NY", "Perrysburg, NY", "Peru, NY",

"Phelps, NY", "Philadelphia, NY", "Phoenicia, NY", "Phoenix, NY",
 "Piercefield, NY", "Piermont, NY", "Pilot Knob, NY", "Pine Bush, NY",
 "Pine City, NY", "Pine Hill, NY", "Pine Island, NY", "Pine Plains, NY",
 "Pine Valley, NY", "Piseco, NY", "Pitcher, NY", "Pittsford, NY",
 "Plainview, NY", "Plandome, NY", "Plattsburgh, NY",
 "Pleasant Valley, NY", "Pleasantville, NY", "Plessis, NY",
 "Plymouth, NY", "Poestenkill, NY", "Point Vivian, NY", "Poland, NY",
 "Pomona, NY", "Pond Eddy, NY", "Port Byron, NY", "Port Ewen, NY",
 "Port Henry, NY", "Port Jefferson, NY", "Port Jefferson S, NY",
 "Port Jervis, NY", "Port Leyden, NY", "Port Washington, NY",
 "Portageville, NY", "Porter Corners, NY", "Portland, NY",
 "Portlandville, NY", "Portville, NY", "Potsdam, NY", "Pottersville, NY",
 "Poughquag, NY", "Pound Ridge, NY", "Prattsburg, NY", "Prattsville, NY",
 "Preble, NY", "Preston Hollow, NY", "Pulaski, NY", "Pulteney, NY",
 "Purchase, NY", "Purdys, NY", "Purling, NY", "Putnam Station, NY",
 "Putnam Valley, NY", "Queens Village, NY", "Queensbury, NY",
 "Randolph, NY", "Ransomville, NY", "Raquette Lake, NY", "Ravena, NY",
 "Red Creek, NY", "Red Hook, NY", "Redfield, NY", "Redford, NY",
 "Redwood, NY", "Rego Park, NY", "Remsen, NY", "Rensselaer, NY",
 "Rensselaer Falls, NY", "Rensselaerville, NY", "Rexford, NY",
 "Rexville, NY", "Rhinebeck, NY", "Richfield Spring, NY", "Richford, NY",
 "Richland, NY", "Richmond Hill, NY", "Richmondville, NY",
 "Richville, NY", "Ridge, NY", "Ridgewood, NY", "Ripley, NY",
 "Riverhead, NY", "Rochester, NY", "Rock City Falls, NY",
 "Rock Glen, NY", "Rock Hill, NY", "Rock Stream, NY", "Rock Tavern, NY",
 "Rockville Centre, NY", "Rocky Point, NY", "Rodman, NY",
 "Roessleville, NY", "Rome, NY", "Roosevelt, NY", "Roseboom, NY",
 "Rosedale, NY", "Rosendale, NY", "Roslyn, NY", "Roslyn Heights, NY",
 "Rotterdam, NY", "Rotterdam Juncti, NY", "Round Lake, NY",
 "Round Top, NY", "Rouses Point, NY", "Roxbury, NY", "Rushford, NY",
 "Rushville, NY", "Rye, NY", "Rye Brook, NY", "S Ozone Park, NY",
 "S Richmond Hill, NY", "Sackets Harbor, NY", "Sag Harbor, NY",
 "Saint Huberts, NY", "Saint James, NY", "Saint Johnsville, NY",
 "Saint Regis Fall, NY", "Salamanca, NY", "Salem, NY",
 "Salisbury Center, NY", "Salisbury Mills, NY", "Salt Point, NY",
 "Sanborn, NY", "Sand Lake, NY", "Sandy Creek, NY",
 "Sanitaria Spring, NY", "Saranac, NY", "Saranac Lake, NY",
 "Sardinia, NY", "Saugerties, NY", "Sauquoit, NY", "Savannah, NY",
 "Savona, NY", "Schaghticoke, NY", "Schenectady, NY", "Schenevus, NY",
 "Schodack Landing, NY", "Schoharie, NY", "Schroon Lake, NY",
 "Schuyler Falls, NY", "Schuylerville, NY", "Scio, NY", "Scotchtown, NY",
 "Scottsburg, NY", "Scottsville, NY", "Sea Cliff, NY", "Seaford, NY",
 "Selden, NY", "Selkirk, NY", "Seneca Falls, NY", "Setauket, NY",
 "Severance, NY", "Shady, NY", "Shandaken, NY", "Sharon Springs, NY",
 "Shelter Island, NY", "Shelter Island H, NY", "Sherburne, NY",
 "Sherman, NY", "Sherrill, NY", "Shirley, NY", "Shokan, NY",
 "Shoreham, NY", "Shortsville, NY", "Shrub Oak, NY", "Shushan, NY",
 "Sidney, NY", "Sidney Center, NY", "Silver Bay, NY", "Silver Creek, NY",
 "Sinclairville, NY", "Skaneateles, NY", "Slate Hill, NY",
 "Slaterville Spri, NY", "Slingerlands, NY", "Sloansville, NY",
 "Smithtown, NY", "Smithville, NY", "Smithville Flats, NY", "Smyrna, NY",
 "Sodus, NY", "Sodus Point, NY", "Solvay, NY", "Somers, NY",
 "Sound Beach, NY", "South Cairo, NY", "South Colton, NY",
 "South Dayton, NY", "South Edmeston, NY", "South Fallsburg, NY",
 "South Farmingdal, NY", "South Glens Fall, NY", "South Kortright, NY",

"South New Berlin, NY", "South Otselic, NY", "South Plymouth, NY",
"South Road, NY", "South Salem, NY", "South Wales, NY",
"Southampton, NY", "Southfields, NY", "Southold, NY", "Sparkill, NY",
"Sparrowbush, NY", "Speculator, NY", "Spencer, NY", "Spencerport, NY",
"Spencertown, NY", "Sprakers, NY", "Springfield Cent, NY",
"Springfield Gard, NY", "Springville, NY", "Springwater, NY",
"Staatsburg, NY", "Stafford, NY", "Stamford, NY", "Stanfordville, NY",
"Stanley, NY", "Star Lake, NY", "Staten Island, NY", "Stephentown, NY",
"Sterling, NY", "Sterlington, NY", "Stillwater, NY", "Stittville, NY",
"Stockton, NY", "Stone Ridge, NY", "Stony Brook, NY", "Stony Creek, NY",
"Stony Point, NY", "Stormville, NY", "Stratford, NY",
"Strykersville, NY", "Stuyvesant, NY", "Suffern, NY", "Summit, NY",
"Sundown, NY", "Sunmount, NY", "Sunnyside, NY", "Sunny Stony Brook, NY",
"Surprise, NY", "Swain, NY", "Swan Lake, NY", "Swormville, NY",
"Syosset, NY", "Syracuse, NY", "Taberg, NY", "Taconic Lake, NY",
"Tannersville, NY", "Tappan, NY", "Theresa, NY", "Thiells, NY",
"Thompson Ridge, NY", "Thomson, NY", "Thornwood, NY",
"Three Mile Bay, NY", "Ticonderoga, NY", "Tillson, NY", "Tivoli, NY",
"Tomkins Cove, NY", "Tonawanda, NY", "Treadwell, NY", "Troupsburg, NY",
"Troy, NY", "Trumansburg, NY", "Truxton, NY", "Tuckahoe, NY",
"Tully, NY", "Turin, NY", "Tuscarora, NY", "Tuxedo Park, NY",
"Twelve Corners, NY", "Ulster Park, NY", "Unadilla, NY",
"Union Springs, NY", "Uniondale, NY", "Upper Jay, NY",
"Upper Saint Regi, NY", "Utica, NY", "Valatie, NY", "Valhalla, NY",
"Valley Cottage, NY", "Valley Falls, NY", "Valley Stream, NY",
"Valois, NY", "Van Buren Bay, NY", "Van Etten, NY",
"Van Hornesville, NY", "Varysburg, NY", "Venice Center, NY",
"Verbank, NY", "Vermontville, NY", "Vernon, NY", "Vernon Center, NY",
"Verona, NY", "Vestal, NY", "Veterans Adminis, NY", "Victor, NY",
"Voorheesville, NY", "Waccabuc, NY", "Waddington, NY",
"Wading River, NY", "Wadsworth, NY", "Walden, NY", "Wallace, NY",
"Wallkill, NY", "Walton, NY", "Walworth, NY", "Wanakena, NY",
"Wantagh, NY", "Warners, NY", "Warnerville, NY", "Warrensburg, NY",
"Warsaw, NY", "Warwick, NY", "Washingtonville, NY", "Wassaic, NY",
"Water Mill, NY", "Waterford, NY", "Waterloo, NY", "Waterport, NY",
"Watertown, NY", "Waterville, NY", "Watervliet, NY", "Watkins Glen, NY",
"Waverly, NY", "Wayland, NY", "Webster, NY", "Weedsport, NY",
"Wells, NY", "Wells Bridge, NY", "Wellsburg, NY", "Wellsville, NY",
"West Babylon, NY", "West Brentwood, NY", "West Burlington, NY",
"West Charlton, NY", "West Chazy, NY", "West Coxsackie, NY",
"West Edmeston, NY", "West Falls, NY", "West Fulton, NY",
"West Haverstraw, NY", "West Hempstead, NY", "West Henrietta, NY",
"West Hurley, NY", "West Islip, NY", "West Kill, NY", "West Leyden, NY",
"West Monroe, NY", "West Nyack, NY", "West Oneonta, NY",
"West Point, NY", "West Rush, NY", "West Sand Lake, NY",
"West Sayville, NY", "West Seneca, NY", "West Shokan, NY",
"West Stockholm, NY", "West Valley, NY", "West Winfield, NY",
"Westbury, NY", "Westdale, NY", "Westerlo, NY", "Westernville, NY",
"Westfield, NY", "Westford, NY", "Westgate, NY", "Westhampton, NY",
"Westhampton Beac, NY", "Westmoreland, NY", "Westport, NY",
"Westtown, NY", "Wevertown, NY", "Whallonsburg, NY",
"Wheatley Heights, NY", "White Creek, NY", "White Lake, NY",
"White Plains, NY", "White Sulphur Sp, NY", "Whitehall, NY",
"Whitesboro, NY", "Whitestone, NY", "Whitesville, NY",
"Whitney Point, NY", "Willet, NY", "Williamson, NY", "Williamstown, NY",

"Williamsville, NY", "Williston Park, NY", "Willow, NY",
 "Willsboro, NY", "Willseyville, NY", "Wilmington, NY", "Wilson, NY",
 "Wilton, NY", "Windham, NY", "Windsor, NY", "Wingdale, NY",
 "Winthrop, NY", "Wolcott, NY", "Woodbourne, NY", "Woodbury, NY",
 "Woodgate, NY", "Woodhaven, NY", "Woodhull, NY", "Woodmere, NY",
 "Woodridge, NY", "Woodside, NY", "Woodstock, NY", "Woodville, NY",
 "Worcester, NY", "Wurtsboro, NY", "Wynantskill, NY", "Wyoming, NY",
 "Yaphank, NY", "Yonkers, NY", "Yorktown Heights, NY", "Yorkville, NY",
 "Youngstown, NY", "Youngsville, NY", "Yulan, NY", "Aberdeen, OH",
 "Ada, OH", "Adams Mills, OH", "Adamsville, OH", "Addyston, OH",
 "Adena, OH", "Akron, OH", "Albany, OH", "Alexandria, OH", "Alger, OH",
 "Alledonia, OH", "Allensville, OH", "Alliance, OH", "Alvada, OH",
 "Alvordton, OH", "Amanda, OH", "Amelia, OH", "Amesville, OH",
 "Amlin, OH", "Amsterdam, OH", "Anderson, OH", "Andover, OH", "Anna, OH",
 "Ansonia, OH", "Antioch, OH", "Antwerp, OH", "Apple Creek, OH",
 "Arcadia, OH", "Archbold, OH", "Arlington, OH", "Armstrong Mills, OH",
 "Ashland, OH", "Ashley, OH", "Ashtabula, OH", "Ashville, OH",
 "Athens, OH", "Atwater, OH", "Austinburg, OH", "Austintown, OH",
 "Avon, OH", "Avon Lake, OH", "Bainbridge, OH", "Baltic, OH",
 "Baltimore, OH", "Batavia, OH", "Bay Village, OH", "Beach City, OH",
 "Beachwood, OH", "Beallsville, OH", "Beaver, OH", "Beavercreek, OH",
 "Bedford, OH", "Bellaire, OH", "Bellbrook, OH", "Belle Center, OH",
 "Bellefontaine, OH", "Bellevue, OH", "Bellville, OH", "Belmont, OH",
 "Beloit, OH", "Belpre, OH", "Berea, OH", "Bergholz, OH", "Berkey, OH",
 "Berlin Center, OH", "Berlin Heights, OH", "Bethel, OH", "Bethesda, OH",
 "Beverly, OH", "Bexley, OH", "Bidwell, OH", "Big Prairie, OH",
 "Blacklick, OH", "Blanchester, OH", "Bloomdale, OH", "Bloomington, OH",
 "Bloomington, OH", "Bloomville, OH", "Blue Creek, OH", "Blue Rock, OH",
 "Bluffton, OH", "Boardman, OH", "Bolivar, OH", "Bono, OH",
 "Botkins, OH", "Bowerston, OH", "Bowling Green, OH", "Bradford, OH",
 "Bradner, OH", "Brecksville, OH", "Brewster, OH", "Bridgeport, OH",
 "Brilliant, OH", "Brinkhaven, OH", "Bristolville, OH",
 "Broadview Height, OH", "Brookfield, OH", "Brooklyn, OH",
 "Brookpark, OH", "Brookville, OH", "Brunswick, OH", "Bryan, OH",
 "Bucyrus, OH", "Burbank, OH", "Burghill, OH", "Burgoon, OH",
 "Burton, OH", "Butler, OH", "Byesville, OH", "Cable, OH",
 "Calcutta, OH", "Caldwell, OH", "Caledonia, OH", "Camden, OH",
 "Camp Dennison, OH", "Campbell, OH", "Canal Fulton, OH",
 "Canal Winchester, OH", "Canfield, OH", "Canton, OH", "Cardington, OH",
 "Carey, OH", "Carlisle, OH", "Carroll, OH", "Carrollton, OH",
 "Carrothers, OH", "Carthage, OH", "Casstown, OH", "Castalia, OH",
 "Castine, OH", "Cecil, OH", "Cedarville, OH", "Centerburg, OH",
 "Centerville, OH", "Chagrin Falls, OH", "Chandlersville, OH",
 "Chardon, OH", "Chatfield, OH", "Chesapeake, OH", "Cheshire, OH",
 "Chesterhill, OH", "Chesterland, OH", "Chillicothe, OH",
 "Chippewa Lake, OH", "Cincinnati, OH", "Circleville, OH",
 "Clarington, OH", "Clarksburg, OH", "Clarksville, OH", "Claysville, OH",
 "Clayton, OH", "Cleveland, OH", "Cleveland Height, OH", "Cleves, OH",
 "Clinton, OH", "Cloverdale, OH", "Clyde, OH", "Coldwater, OH",
 "College Corner, OH", "College Hill, OH", "Collins, OH",
 "Columbia Station, OH", "Columbiana, OH", "Columbus, OH",
 "Columbus Grove, OH", "Conesville, OH", "Conneaut, OH", "Conover, OH",
 "Continental, OH", "Convoy, OH", "Coolville, OH", "Copley, OH",
 "Cortland, OH", "Coshocton, OH", "Covington, OH", "Creola, OH",
 "Crestline, OH", "Creston, OH", "Cridersville, OH", "Crooksville, OH",

"Croton, OH", "Crown City, OH", "Cumberland, OH", "Curtice, OH",
"Custar, OH", "Cutler, OH", "Cuyahoga Falls, OH", "Cygnet, OH",
"Dalton, OH", "Danville, OH", "Day Heights, OH", "Dayton, OH",
"De Graff, OH", "Deerfield, OH", "Defiance, OH", "Delaware, OH",
"Dellroy, OH", "Delphos, OH", "Delta, OH", "Dennison, OH",
"Deshler, OH", "Dexter, OH", "Dexter City, OH", "Diamond, OH",
"Dillonvale, OH", "Dola, OH", "Dorset, OH", "Dover, OH",
"Doylestown, OH", "Dublin, OH", "Duncan Falls, OH", "Dundee, OH",
"Dunkirk, OH", "East Canton, OH", "East Cleveland, OH",
"East Liberty, OH", "East Orwell, OH", "East Palestine, OH",
"East Rochester, OH", "East Sparta, OH", "Eaton, OH", "Edgerton, OH",
"Edgewater, OH", "Edison, OH", "Edon, OH", "Eldorado, OH", "Elida, OH",
"Elliston, OH", "Elmore, OH", "Elmwood Place, OH", "Elyria, OH",
"Enon, OH", "Euclid, OH", "Excello, OH", "Fairborn, OH",
"Fairfield, OH", "Fairlawn, OH", "Fairport Harbor, OH",
"Fairview Park, OH", "Farmdale, OH", "Farmersville, OH", "Fayette, OH",
"Fayetteville, OH", "Felicity, OH", "Findlay, OH", "Fleming, OH",
"Fletcher, OH", "Flushing, OH", "Fort Jennings, OH", "Fort Loramie, OH",
"Fort Recovery, OH", "Fostoria, OH", "Fowler, OH", "Frankfort, OH",
"Franklin Furnace, OH", "Frazeysburg, OH", "Fredericksburg, OH",
"Fredericktown, OH", "Freeport, OH", "Fremont, OH", "Fresno, OH",
"Fulton, OH", "Gahanna, OH", "Galena, OH", "Galion, OH",
"Gallipolis, OH", "Galloway, OH", "Gambier, OH", "Garfield Heights, OH",
"Garrettsville, OH", "Gates Mills, OH", "Geneva, OH", "Genoa, OH",
"Georgetown, OH", "Germantown, OH", "Gibsonburg, OH", "Gilboa, OH",
"Girard, OH", "Glendale, OH", "Glenford, OH", "Glenmont, OH",
"Glouster, OH", "Gnadenhutten, OH", "Goshen, OH", "Grafton, OH",
"Grand Rapids, OH", "Granville, OH", "Green Springs, OH",
"Greenfield, OH", "Greenhills, OH", "Greenville, OH", "Greenwich, OH",
"Groesbeck, OH", "Grove City, OH", "Groveport, OH", "Grover Hill, OH",
"Guernsey, OH", "Guysville, OH", "Hamden, OH", "Hamersville, OH",
"Hamilton, OH", "Hamler, OH", "Hammondsville, OH", "Hanoverton, OH",
"Harpster, OH", "Harrison, OH", "Harrod, OH", "Hartville, OH",
"Haskins, OH", "Haviland, OH", "Heath, OH", "Hebron, OH", "Hemlock, OH",
"Hicksville, OH", "Hide A Way Hills, OH", "Hilliard, OH",
"Hillsboro, OH", "Hinckley, OH", "Hiram, OH", "Holgate, OH",
"Holland, OH", "Hollansburg, OH", "Holmesville, OH", "Homerville, OH",
"Homeworth, OH", "Hopedale, OH", "Hopewell, OH", "Houston, OH",
"Howard, OH", "Hubbard, OH", "Huber Heights, OH", "Hudson, OH",
"Huntsburg, OH", "Huntsville, OH", "Idaho, OH", "Independence, OH",
"Irondale, OH", "Irononton, OH", "Irwin, OH", "Isle Saint Georg, OH",
"Jackson, OH", "Jackson Belden, OH", "Jackson Center, OH",
"Jamestown, OH", "Jefferson, OH", "Jeffersonville, OH", "Jenera, OH",
"Jeromesville, OH", "Jerusalem, OH", "Jewett, OH", "Johnstown, OH",
"Junction City, OH", "Kansas, OH", "Kelleys Island, OH",
"Kensington, OH", "Kent, OH", "Kenton, OH", "Kettering, OH",
"Killbuck, OH", "Kingston, OH", "Kingsville, OH", "Kinsman, OH",
"Kitts Hill, OH", "La Rue, OH", "Lacarne, OH", "Lagrange, OH",
"Lake Milton, OH", "Lakeside, OH", "Lakeview, OH", "Lakeville, OH",
"Lancaster, OH", "Latham, OH", "Laura, OH", "Laurelville, OH",
"Leavittsburg, OH", "Leesburg, OH", "Leetonia, OH", "Leipsic, OH",
"Lewisburg, OH", "Lewistown, OH", "Lewisville, OH", "Lexington, OH",
"Liberty Center, OH", "Lima, OH", "Lincoln, OH", "Lincoln Village, OH",
"Lindenwald, OH", "Lindsey, OH", "Lisbon, OH", "Litchfield, OH",
"Little Hocking, OH", "Lockbourne, OH", "Lockland, OH", "Lodi, OH",

"Logan, OH", "London, OH", "Londonderry, OH", "Long Bottom, OH",
 "Lorain, OH", "Lore City, OH", "Loudonville, OH", "Louisville, OH",
 "Loveland, OH", "Lowell, OH", "Lowellville, OH", "Lucas, OH",
 "Lucasville, OH", "Luckey, OH", "Ludlow Falls, OH", "Lynchburg, OH",
 "Lyndhurst Mayfie, OH", "Lyons, OH", "Macedonia, OH", "Macksburg, OH",
 "Madeira, OH", "Madison, OH", "Madisonville, OH",
 "Magnetic Springs, OH", "Magnolia, OH", "Maineville, OH", "Malinta, OH",
 "Malta, OH", "Malvern, OH", "Manchester, OH", "Mansfield, OH",
 "Mantua, OH", "Maple Heights, OH", "Maplewood, OH", "Marengo, OH",
 "Maria Stein, OH", "Marietta, OH", "Marion, OH", "Mark Center, OH",
 "Marshallville, OH", "Martel, OH", "Martins Ferry, OH",
 "Martinsburg, OH", "Martinsville, OH", "Marysville, OH", "Mason, OH",
 "Massillon, OH", "Masury, OH", "Maumee, OH", "Mc Clure, OH",
 "Mc Comb, OH", "Mc Connelssville, OH", "Mc Cutchenville, OH",
 "Mc Dermott, OH", "Mc Donald, OH", "Mechanicsburg, OH",
 "Mechanicstown, OH", "Medina, OH", "Medway, OH", "Melmore, OH",
 "Mendon, OH", "Mentor, OH", "Metamora, OH", "Miami University, OH",
 "Miamisburg, OH", "Middle Point, OH", "Middlefield, OH",
 "Middleport, OH", "Middletown, OH", "Midland, OH", "Midpark, OH",
 "Milan, OH", "Milford Center, OH", "Millbury, OH", "Millersburg, OH",
 "Millersport, OH", "Millersville, OH", "Millfield, OH",
 "Mineral Ridge, OH", "Minerva, OH", "Minford, OH", "Mingo Junction, OH",
 "Minster, OH", "Mogadore, OH", "Monclova, OH", "Monroe, OH",
 "Monroeville, OH", "Montpelier, OH", "Montville, OH", "Moorefield, OH",
 "Morrall, OH", "Morrow, OH", "Moscow, OH", "Mount Blanchard, OH",
 "Mount Cory, OH", "Mount Gilead, OH", "Mount Orab, OH",
 "Mount Perry, OH", "Mount Sterling, OH", "Mount Vernon, OH",
 "Mount Victory, OH", "Munroe Falls, OH", "Napoleon, OH", "Nashport, OH",
 "Navarre, OH", "Negley, OH", "Nelsonville, OH", "Nevada, OH",
 "New Albany, OH", "New Bavaria, OH", "New Bloomington, OH",
 "New Boston, OH", "New Bremen, OH", "New Carlisle, OH",
 "New Concord, OH", "New Holland, OH", "New Knoxville, OH",
 "New Lebanon, OH", "New Lexington, OH", "New London, OH",
 "New Madison, OH", "New Marshfield, OH", "New Matamoras, OH",
 "New Middletown, OH", "New Paris, OH", "New Philadelphia, OH",
 "New Plymouth, OH", "New Richmond, OH", "New Riegel, OH",
 "New Springfield, OH", "New Straitsville, OH", "New Vienna, OH",
 "New Washington, OH", "New Waterford, OH", "New Weston, OH",
 "Newark, OH", "Newbury, OH", "Newcomerstown, OH", "Newport, OH",
 "Newton Falls, OH", "Newtown, OH", "Ney, OH", "Niles, OH", "Noble, OH",
 "North Baltimore, OH", "North Bend, OH", "North Benton, OH",
 "North Bloomfield, OH", "North Canton, OH", "North Fairfield, OH",
 "North Industry, OH", "North Jackson, OH", "North Lawrence, OH",
 "North Lewisburg, OH", "North Lima, OH", "North Olmsted, OH",
 "North Ridgeville, OH", "North Royalton, OH", "Northfield, OH",
 "Northwood, OH", "Norton, OH", "Norwalk, OH", "Norwich, OH",
 "Norwood, OH", "Nova, OH", "Novelty, OH", "Oak Harbor, OH",
 "Oak Hill, OH", "Oakwood, OH", "Oberlin, OH", "Ohio City, OH",
 "Okeana, OH", "Olmsted Falls, OH", "Oregon, OH", "Oregonia, OH",
 "Orient, OH", "Orrville, OH", "Ostrander, OH", "Otterbien Home, OH",
 "Ottoville, OH", "Otway, OH", "Pandora, OH", "Paris, OH",
 "Parkdale, OH", "Parma, OH", "Pataskala, OH", "Patriot, OH",
 "Patterson, OH", "Paulding, OH", "Payne, OH", "Pedro, OH",
 "Peebles, OH", "Pemberville, OH", "Peninsula, OH", "Pennsville, OH",
 "Perry, OH", "Perrysburg, OH", "Perrysville, OH", "Petersburg, OH",

"Philo, OH", "Phoneton, OH", "Pickerington, OH", "Piedmont, OH",
 "Pierpont, OH", "Pioneer, OH", "Piqua, OH", "Plain City, OH",
 "Pleasant City, OH", "Pleasant Hill, OH", "Pleasant Plain, OH",
 "Pleasantville, OH", "Plymouth, OH", "Poland, OH", "Polk, OH",
 "Pomeroy, OH", "Port Clinton, OH", "Port Washington, OH", "Portage, OH",
 "Portland, OH", "Portsmouth, OH", "Powhatan Point, OH",
 "Proctorville, OH", "Prospect, OH", "Put In Bay, OH", "Quaker City, OH",
 "Quincy, OH", "Racine, OH", "Radcliff, OH", "Radnor, OH", "Rarden, OH",
 "Ravenna, OH", "Rawson, OH", "Ray, OH", "Rayland, OH", "Raymond, OH",
 "Reedsville, OH", "Reminderville, OH", "Reno, OH", "Republic, OH",
 "Reynoldsburg, OH", "Richfield, OH", "Richmond, OH",
 "Richmond Dale, OH", "Richmond Heights, OH", "Richwood, OH",
 "Ridgeway, OH", "Rinard Mills, OH", "Ripley, OH", "Risingsun, OH",
 "Rittman, OH", "Roaming Shores, OH", "Rock Camp, OH", "Rockbridge, OH",
 "Rockford, OH", "Rocky River, OH", "Rogers, OH", "Rootstown, OH",
 "Roseville, OH", "Rossburg, OH", "Rossford, OH", "Rossville, OH",
 "Roundhead, OH", "Rudolph, OH", "Rushsylvania, OH", "Rushville, OH",
 "Russells Point, OH", "Russellville, OH", "Russia, OH", "Rutland, OH",
 "Sabina, OH", "Saint Bernard, OH", "Saint Clairsvill, OH",
 "Saint Henry, OH", "Saint Louisville, OH", "Saint Marys, OH",
 "Saint Paris, OH", "Salem, OH", "Salesville, OH", "Salineville, OH",
 "Sandusky, OH", "Sarahsville, OH", "Sardinia, OH", "Sardis, OH",
 "Savannah, OH", "Saylor Park, OH", "Scio, OH", "Scott, OH",
 "Scottown, OH", "Seaman, OH", "Sebring, OH", "Selma, OH",
 "Senecaville, OH", "Seville, OH", "Shade, OH", "Shadyside, OH",
 "Sharonville, OH", "Shawnee, OH", "Shawnee Hills, OH",
 "Sheffield Lake, OH", "Shelby, OH", "Shepard, OH", "Sherrods, OH",
 "Sherwood, OH", "Shiloh, OH", "Shinrock, OH", "Shore, OH", "Shreve, OH",
 "Sidney, OH", "Smithville, OH", "Solon, OH", "Somerset, OH",
 "Somerton, OH", "Somerville, OH", "Sonora, OH", "South Amherst, OH",
 "South Bloomingvi, OH", "South Euclid, OH", "South Lebanon, OH",
 "South Point, OH", "South Salem, OH", "South Solon, OH",
 "South Vienna, OH", "South Webster, OH", "Southington, OH",
 "Sparta, OH", "Spencer, OH", "Spencerville, OH", "Spring Valley, OH",
 "Springboro, OH", "Springfield, OH", "Sterling, OH", "Stewart, OH",
 "Stone Creek, OH", "Stout, OH", "Stoutsville, OH", "Stow, OH",
 "Strasburg, OH", "Streetsboro, OH", "Strongsville, OH", "Struthers, OH",
 "Stryker, OH", "Sugar Grove, OH", "Sugarcreek, OH", "Sullivan, OH",
 "Summerfield, OH", "Sunbury, OH", "Swanton, OH", "Sycamore, OH",
 "sylvania, OH", "Taft, OH", "Tallmadge, OH", "Terrace Park, OH",
 "The Plains, OH", "Thompson, OH", "Thornville, OH", "Thurman, OH",
 "Tiffin, OH", "Tiltonsville, OH", "Tippecanoe, OH", "Tiro, OH",
 "Toledo, OH", "Toronto, OH", "Trenton, OH", "Trotwood, OH", "Troy, OH",
 "Twinsburg, OH", "Uhrichsville, OH", "Union, OH", "Union City, OH",
 "Uniontown, OH", "Upper Arlington, OH", "Upper Sandusky, OH",
 "Urbana, OH", "Utica, OH", "Valley City, OH", "Van Buren, OH",
 "Van Wert, OH", "Vandalia, OH", "Vanlue, OH", "Venedocia, OH",
 "Vermilion, OH", "Versailles, OH", "Vickery, OH", "Vienna, OH",
 "Vincent, OH", "Vinton, OH", "Wadsworth, OH", "Wakeman, OH",
 "Walbridge, OH", "Waldo, OH", "Walhonding, OH", "Wapakoneta, OH",
 "Warner, OH", "Warren, OH", "Warsaw, OH", "Washington Court, OH",
 "Washingtonville, OH", "Waterford, OH", "Waterloo, OH",
 "Waterville, OH", "Wauseon, OH", "Waverly, OH", "Wayne, OH",
 "Waynesburg, OH", "Waynesfield, OH", "Waynesville, OH",
 "Wellington, OH", "Wellston, OH", "Wellsville, OH",

"West Alexandria, OH", "West Carrollton, OH", "West Chester, OH",
 "West Farmington, OH", "West Jefferson, OH", "West Lafayette, OH",
 "West Liberty, OH", "West Manchester, OH", "West Mansfield, OH",
 "West Milton, OH", "West Salem, OH", "West Union, OH", "West Unity, OH",
 "West Worthington, OH", "Western Hills, OH", "Westerville, OH",
 "Westlake, OH", "Weston, OH", "Westwood, OH", "Wharton, OH",
 "Wheelerburg, OH", "Whipple, OH", "Whitehall, OH", "Whitehouse, OH",
 "Wickliffe, OH", "Willard, OH", "Williamsburg, OH", "Williamsfield, OH",
 "Williamsport, OH", "Willoughby, OH", "Willow Wood, OH",
 "Willowick, OH", "Willshire, OH", "Wilmington, OH", "Wilmot, OH",
 "Winchester, OH", "Windham, OH", "Windsor, OH", "Wingett Run, OH",
 "Wintersville, OH", "Woodstock, OH", "Woodville, OH", "Wooster, OH",
 "Worthington, OH", "Xenia, OH", "Yellow Springs, OH", "Yorkshire, OH",
 "Yorkville, OH", "Youngstown, OH", "Zanesfield, OH", "Zoarville, OH",
 "Ada, OK", "Adair, OK", "Agra, OK", "Alex, OK", "Aline, OK",
 "Allen, OK", "Altus, OK", "Alva, OK", "Amber, OK", "Ames, OK",
 "Amorita, OK", "Anadarko, OK", "Antlers, OK", "Apache, OK",
 "Arapaho, OK", "Arcadia, OK", "Arkoma, OK", "Asher, OK", "Atoka, OK",
 "Atwood, OK", "Baker, OK", "Balko, OK", "Barnsdall, OK",
 "Bartlesville, OK", "Bearden, OK", "Beggs, OK", "Bennington, OK",
 "Bernice, OK", "Bessie, OK", "Bethany, OK", "Bethel, OK",
 "Big Cabin, OK", "Billings, OK", "Binger, OK", "Bison, OK", "Bixby, OK",
 "Blackwell, OK", "Blair, OK", "Blanchard, OK", "Blanco, OK",
 "Bluejacket, OK", "Boise City, OK", "Bokchito, OK", "Bokoshe, OK",
 "Boley, OK", "Boswell, OK", "Boynton, OK", "Bradley, OK", "Braggs, OK",
 "Braman, OK", "Bristow, OK", "Broken Arrow, OK", "Broken Bow, OK",
 "Bunch, OK", "Burbank, OK", "Burlington, OK", "Burneyville, OK",
 "Butler, OK", "Byars, OK", "Byron, OK", "Cache, OK", "Caddo, OK",
 "Calera, OK", "Calumet, OK", "Calvin, OK", "Camargo, OK", "Cameron, OK",
 "Canadian, OK", "Caney, OK", "Canton, OK", "Canute, OK", "Capron, OK",
 "Carmen, OK", "Carnegie, OK", "Carney, OK", "Carrier, OK", "Carter, OK",
 "Cartwright, OK", "Cashion, OK", "Castle, OK", "Catoosa, OK",
 "Cement, OK", "Centrahoma, OK", "Chandler, OK", "Chattanooga, OK",
 "Checotah, OK", "Chelsea, OK", "Cherokee, OK", "Chester, OK",
 "Chickasha, OK", "Choctaw, OK", "Chouteau, OK", "Claremore, OK",
 "Clayton, OK", "Clearview, OK", "Cleo Springs, OK", "Cleveland, OK",
 "Clinton, OK", "Coalgate, OK", "Colbert, OK", "Colcord, OK",
 "Coleman, OK", "Collinsville, OK", "Colony, OK", "Comanche, OK",
 "Commerce, OK", "Cookson, OK", "Copan, OK", "Cordell, OK", "Corn, OK",
 "Council Hill, OK", "Covington, OK", "Coweta, OK", "Coyle, OK",
 "Crawford, OK", "Crescent, OK", "Cushing, OK", "Custer City, OK",
 "Cyril, OK", "Dacoma, OK", "Daisy, OK", "Davidson, OK", "Davis, OK",
 "Deer Creek, OK", "Del City, OK", "Delaware, OK", "Depew, OK",
 "Devol, OK", "Dewey, OK", "Dill City, OK", "Douglas, OK", "Dover, OK",
 "Drummond, OK", "Drumright, OK", "Duke, OK", "Duncan, OK", "Durant, OK",
 "Durham, OK", "Dustin, OK", "Eagle City, OK", "Eagletown, OK",
 "Earlsboro, OK", "Edmond, OK", "El Reno, OK", "Eldorado, OK",
 "Elgin, OK", "Elk City, OK", "Elmer, OK", "Elmore City, OK",
 "Elmwood, OK", "Enid, OK", "Erick, OK", "Eucha, OK", "Eufaula, OK",
 "Fairfax, OK", "Fairland, OK", "Fairmont, OK", "Fargo, OK",
 "Farris, OK", "Faxon, OK", "Fay, OK", "Felt, OK", "Finley, OK",
 "Fittstown, OK", "Fitzhugh, OK", "Fletcher, OK", "Foraker, OK",
 "Forgan, OK", "Fort Cobb, OK", "Fort Gibson, OK", "Fort Sill, OK",
 "Fort Supply, OK", "Fort Towson, OK", "Foss, OK", "Foster, OK",
 "Frederick, OK", "Freedom, OK", "Gage, OK", "Garber, OK", "Garvin, OK",

"Gate, OK", "Geary, OK", "Geronimo, OK", "Glencoe, OK", "Glenpool, OK",
"Goltry, OK", "Goodwell, OK", "Gore, OK", "Gotebo, OK", "Gould, OK",
"Gracemont, OK", "Grady, OK", "Graham, OK", "Grandfield, OK",
"Granite, OK", "Grant, OK", "Greenfield, OK", "Grove, OK",
"Guthrie, OK", "Guymon, OK", "Hammon, OK", "Harden City, OK",
"Hardesty, OK", "Harmon, OK", "Harrah, OK", "Hartshorne, OK",
"Haskell, OK", "Hastings, OK", "Haywood, OK", "Headrick, OK",
"Healdton, OK", "Heavener, OK", "Helena, OK", "Hendrix, OK",
"Hennepin, OK", "Hennessey, OK", "Hinton, OK", "Hitchcock, OK",
"Hobart, OK", "Hodgen, OK", "Hoffman, OK", "Holdenville, OK",
"Hollis, OK", "Hollister, OK", "Hominy, OK", "Honobia, OK", "Howe, OK",
"Hoyt, OK", "Hugo, OK", "Hulbert, OK", "Hunter, OK", "Hydro, OK",
"Idabel, OK", "Indiahoma, OK", "Indianola, OK", "Inola, OK",
"Isabella, OK", "Jay, OK", "Jenks, OK", "Jennings, OK", "Jet, OK",
"Jones, OK", "Kansas, OK", "Kaw City, OK", "Kellyville, OK",
"Kendrick, OK", "Kenefic, OK", "Kenton, OK", "Keota, OK", "Keyes, OK",
"Kingfisher, OK", "Kingston, OK", "Kinta, OK", "Kiowa, OK",
"Knowles, OK", "Konawa, OK", "Kremlin, OK", "Lahoma, OK", "Lamar, OK",
"Lamont, OK", "Lane, OK", "Laverne, OK", "Lawton, OK", "Leach, OK",
"Lebanon, OK", "Leedey, OK", "Lenapah, OK", "Leon, OK", "Lexington, OK",
"Lindsay, OK", "Loco, OK", "Locust Grove, OK", "Logan, OK",
"Lone Grove, OK", "Lone Wolf, OK", "Longdale, OK", "Lookeba, OK",
"Loveland, OK", "Loyal, OK", "Lucien, OK", "Luther, OK", "Macomb, OK",
"Manchester, OK", "Mannford, OK", "Mannsville, OK", "Maramec, OK",
"Marietta, OK", "Marland, OK", "Marlow, OK", "Marshall, OK", "Maud, OK",
"May, OK", "Maysville, OK", "Mc Loud, OK", "Mc Millan, OK",
"Mcalester, OK", "Mccurtain, OK", "Mead, OK", "Medford, OK",
"Meeker, OK", "Meno, OK", "Meridian, OK", "Miami, OK",
"Midwest City, OK", "Milburn, OK", "Mill Creek, OK", "Milo, OK",
"Minco, OK", "Moore, OK", "Mooreland, OK", "Morris, OK", "Morrison, OK",
"Mounds, OK", "Mountain Park, OK", "Mountain View, OK", "Moyers, OK",
"Muldrow, OK", "Mulhall, OK", "Muse, OK", "Muskogee, OK", "Mustang, OK",
"Mutual, OK", "Nardin, OK", "Nash, OK", "Nashoba, OK", "New Lima, OK",
"Newalla, OK", "Newcastle, OK", "Nichols Hills, OK", "Ninnekah, OK",
"Noble, OK", "Norman, OK", "Nowata, OK", "Oaks, OK", "Ochelata, OK",
"Octavia, OK", "Okarche, OK", "Okeene, OK", "Oklahoma City, OK",
"Okmulgee, OK", "Oktaha, OK", "Olustee, OK", "Omega, OK", "Oologah, OK",
"Optima, OK", "Orienta, OK", "Orlando, OK", "Osage, OK", "Oscar, OK",
"Overbrook, OK", "Owasso, OK", "Paden, OK", "Paoli, OK",
"Park Hill, OK", "Pauls Valley, OK", "Pawhuska, OK", "Pawnee, OK",
"Peckham, OK", "Peggs, OK", "Perkins, OK", "Perry, OK", "Picher, OK",
"Piedmont, OK", "Pittsburg, OK", "Pocasset, OK", "Pocola, OK",
"Ponca City, OK", "Pond Creek, OK", "Porter, OK", "Porum, OK",
"Poteau, OK", "Prague, OK", "Proctor, OK", "Prue, OK", "Pryor, OK",
"Purcell, OK", "Putnam, OK", "Quapaw, OK", "Quinton, OK", "Ralston, OK",
"Ramona, OK", "Randlett, OK", "Ratliff City, OK", "Rattan, OK",
"Red Oak, OK", "Red Rock, OK", "Reed, OK", "Reydon, OK", "Ringling, OK",
"Ringold, OK", "Ringwood, OK", "Ripley, OK", "Rocky, OK", "Roff, OK",
"Roland, OK", "Roosevelt, OK", "Rosston, OK", "Rubottom, OK",
"Rufe, OK", "Rush Springs, OK", "Ryan, OK", "S Coffeyville, OK",
"Salina, OK", "Sallisaw, OK", "Sand Springs, OK", "Sapulpa, OK",
"Sasakwa, OK", "Sawyer, OK", "Sayre, OK", "Seiling, OK", "Selman, OK",
"Seminole, OK", "Sentinel, OK", "Shady Point, OK", "Sharon, OK",
"Shattuck, OK", "Shawnee, OK", "Skiatook, OK", "Snow, OK", "Snyder, OK",
"Soper, OK", "Southard, OK", "Sparks, OK", "Spavinaw, OK",

"Spencer, OK", "Spencerville, OK", "Sperry, OK", "Spiro, OK",
 "Springer, OK", "Stidham, OK", "Stigler, OK", "Stillwater, OK",
 "Stilwell, OK", "Strang, OK", "Stratford, OK", "Stringtown, OK",
 "Strong City, OK", "Stuart, OK", "Sulphur, OK", "Sweetwater, OK",
 "Taft, OK", "Tahlequah, OK", "Talala, OK", "Talihina, OK", "Taloga, OK",
 "Tecumseh, OK", "Temple, OK", "Terlton, OK", "Texhoma, OK",
 "Texola, OK", "Thackerville, OK", "Thomas, OK", "Tinker Afb, OK",
 "Tipton, OK", "Tishomingo, OK", "Tom, OK", "Tonkawa, OK", "Tryon, OK",
 "Tulsa, OK", "Tupelo, OK", "Tuskahoma, OK", "Tussy, OK", "Tuttle, OK",
 "Twin Oaks, OK", "Tyrone, OK", "Union City, OK", "Valliant, OK",
 "Verden, OK", "Vernon, OK", "Vian, OK", "Vici, OK", "Vinita, OK",
 "Vinson, OK", "Wagoner, OK", "Wakita, OK", "Walters, OK", "Wanette, OK",
 "Wann, OK", "Wapanucka, OK", "Wardville, OK", "Warner, OK",
 "Warr Acres, OK", "Washington, OK", "Watonga, OK", "Watson, OK",
 "Watts, OK", "Waukomis, OK", "Waurika, OK", "Wayne, OK", "Waynoka, OK",
 "Weatherford, OK", "Webbers Falls, OK", "Welch, OK", "Weleetka, OK",
 "Welling, OK", "Wellston, OK", "Welty, OK", "Westville, OK",
 "Wetumka, OK", "Whitefield, OK", "Whitesboro, OK", "Wilburton, OK",
 "Willow, OK", "Wister, OK", "Woodward, OK", "Wright City, OK",
 "Wyandotte, OK", "Wynnewood, OK", "Wynona, OK", "Yale, OK", "Yukon, OK",
 "Adams, OR", "Adel, OR", "Adrian, OR", "Agness, OR", "Albany, OR",
 "Aloha, OR", "Alsea, OR", "Amity, OR", "Antelope, OR", "Applegate, OR",
 "Arlington, OR", "Arock, OR", "Ashland, OR", "Ashwood, OR",
 "Astoria, OR", "Athena, OR", "Aurora, OR", "Azalea, OR", "Bandon, OR",
 "Banks, OR", "Bay City, OR", "Beatty, OR", "Beaver, OR",
 "Beavercreek, OR", "Beaverton, OR", "Bend, OR", "Blachly, OR",
 "Black Butte Ranc, OR", "Blodgett, OR", "Boardman, OR", "Bonanza, OR",
 "Bonneville, OR", "Boring, OR", "Bridal Veil, OR", "Brightwood, OR",
 "Broadbent, OR", "Brogan, OR", "Brooks, OR", "Brothers, OR",
 "Brownsville, OR", "Burns, OR", "Butte Falls, OR", "Buxton, OR",
 "Camas Valley, OR", "Camp Sherman, OR", "Canby, OR", "Canyon City, OR",
 "Canyonville, OR", "Carlton, OR", "Cascadia, OR", "Cave Junction, OR",
 "Cedar Hills, OR", "Central Point, OR", "Charleston, OR",
 "Cheshire, OR", "Chiloquin, OR", "Clackamas, OR", "Cloverdale, OR",
 "Coburg, OR", "Colton, OR", "Columbia City, OR", "Condon, OR",
 "Coquille, OR", "Corbett, OR", "Cornelius, OR", "Corvallis, OR",
 "Cottage Grove, OR", "Cove, OR", "Crane, OR", "Crescent, OR",
 "Creswell, OR", "Crooked River Ra, OR", "Culp Creek, OR", "Culver, OR",
 "Dairy, OR", "Dallas, OR", "Days Creek, OR", "Dayton, OR",
 "Dayville, OR", "Deer Island, OR", "Depoe Bay, OR", "Detroit, OR",
 "Dexter, OR", "Diamond Lake, OR", "Dorena, OR", "Drain, OR",
 "Drewsey, OR", "Dundee, OR", "Eagle Creek, OR", "Eagle Point, OR",
 "Echo, OR", "Eddyville, OR", "Elgin, OR", "Elkton, OR", "Elmira, OR",
 "Enterprise, OR", "Estacada, OR", "Eugene, OR", "Fall Creek, OR",
 "Falls City, OR", "Florence, OR", "Fort Rock, OR", "Foster, OR",
 "Fox, OR", "Friend, OR", "Gales Creek, OR", "Garden Home, OR",
 "Gardiner, OR", "Gaston, OR", "Gates, OR", "Gearhart, OR",
 "Gervais, OR", "Gilchrist, OR", "Gladstone, OR", "Glendale, OR",
 "Glenwood, OR", "Glide, OR", "Gold Hill, OR", "Grand Ronde, OR",
 "Grants Pass, OR", "Grass Valley, OR", "Greenleaf, OR", "Gresham, OR",
 "Haines, OR", "Halfway, OR", "Halsey, OR", "Hammond, OR", "Harbor, OR",
 "Harper, OR", "Harrisburg, OR", "Hebo, OR", "Helix, OR", "Heppner, OR",
 "Hereford, OR", "Hermiston, OR", "Hillsboro, OR", "Hood River, OR",
 "Hubbard, OR", "Huntington, OR", "Idanha, OR", "Idleld Park, OR",
 "Imbler, OR", "Imnaha, OR", "Independence, OR", "Ione, OR",

"Ironside, OR", "Irrigon, OR", "Jamieson, OR", "Jefferson, OR",
 "John Day, OR", "Jordan Valley, OR", "Joseph, OR", "Junction City, OR",
 "Juntura, OR", "Keizer, OR", "Keno, OR", "Kent, OR", "Kerby, OR",
 "Kimberly, OR", "Kinzua, OR", "Klamath Falls, OR", "La Grande, OR",
 "La Pine, OR", "Lafayette, OR", "Lake Oswego, OR", "Lakeside, OR",
 "Lakeview, OR", "Langlois, OR", "Lawen, OR", "Leaburg, OR",
 "Lebanon, OR", "Lexington, OR", "Lincoln City, OR", "Logsden, OR",
 "Long Creek, OR", "Lorane, OR", "Lostine, OR", "Lowell, OR",
 "Lyons, OR", "Madras, OR", "Malin, OR", "Manning, OR", "Mapleton, OR",
 "Marcola, OR", "Maupin, OR", "Mc Kenzie Bridge, OR", "Mcminnville, OR",
 "Mcrary, OR", "Medford, OR", "Medical Springs, OR", "Merlin, OR",
 "Merrill, OR", "Mill City, OR", "Milton Freewater, OR", "Milwaukie, OR",
 "Mitchell, OR", "Molalla, OR", "Monmouth, OR", "Monroe, OR",
 "Monument, OR", "Moro, OR", "Mosier, OR", "Mount Angel, OR",
 "Mount Hood Parkd, OR", "Mount Vernon, OR", "Mulino, OR",
 "Myrtle Creek, OR", "Myrtle Point, OR", "Nehalem, OR", "Neotsu, OR",
 "Neskowin, OR", "New Pine Creek, OR", "Newberg, OR", "Newport, OR",
 "North Bend, OR", "North Powder, OR", "Noti, OR", "Nyssa, OR",
 "O Brien, OR", "Oak Grove, OR", "Oakland, OR", "Oakridge, OR",
 "Odell, OR", "Ontario, OR", "Oregon City, OR", "Oretech, OR",
 "Otis, OR", "Paisley, OR", "Paulina, OR", "Pendleton, OR",
 "Philomath, OR", "Phoenix, OR", "Pilot Rock, OR", "Pistol River, OR",
 "Pleasant Hill, OR", "Plush, OR", "Port Orford, OR", "Portland, OR",
 "Post, OR", "Powell Butte, OR", "Powers, OR", "Prairie City, OR",
 "Prineville, OR", "Prospect, OR", "Rainier, OR", "Redmond, OR",
 "Remote, OR", "Richland, OR", "Rickreall, OR", "Riddle, OR",
 "Riley, OR", "Ritter, OR", "Riverside, OR", "Rockaway, OR",
 "Rockwood Corners, OR", "Rogue River, OR", "Roseburg, OR", "Rufus, OR",
 "Saint Helens, OR", "Saint Paul, OR", "Salem, OR", "Sandy, OR",
 "Scappoose, OR", "Scio, OR", "Scotts Mills, OR", "Scottsburg, OR",
 "Seal Rock, OR", "Selma, OR", "Seneca, OR", "Shady Cove, OR",
 "Shaniko, OR", "Shedd, OR", "Sheridan, OR", "Sherwood, OR",
 "Siletz, OR", "Silver Lake, OR", "Silverton, OR", "Sixes, OR",
 "South Beach, OR", "Spray, OR", "Springfield, OR", "Stanfield, OR",
 "Stayton, OR", "Sublimity, OR", "Summer Lake, OR", "Summerville, OR",
 "Sumpter, OR", "Sunny Valley, OR", "Sunriver, OR", "Sutherlin, OR",
 "Sweet Home, OR", "Swishhome, OR", "Talent, OR", "Tangent, OR",
 "Tenmile, OR", "The Dalles, OR", "Tidewater, OR", "Tigard, OR",
 "Tillamook, OR", "Tiller, OR", "Timber, OR", "Timberline Lodge, OR",
 "Toledo, OR", "Tolovana Park, OR", "Trail, OR", "Troutdale, OR",
 "Tualatin, OR", "Turner, OR", "Umpqua, OR", "Union, OR", "Unity, OR",
 "Vale, OR", "Veneta, OR", "Vernonia, OR", "Vida, OR", "Waldport, OR",
 "Wallowa, OR", "Walton, OR", "Wamic, OR", "Warm Springs, OR",
 "Warren, OR", "Warrenton, OR", "Wasco, OR", "Welches, OR",
 "West Linn, OR", "West Main, OR", "West Stayton, OR", "Westfall, OR",
 "Westfir, OR", "Westlake, OR", "Weston, OR", "Westport, OR",
 "White City, OR", "Wilderville, OR", "Willamina, OR", "Williams, OR",
 "Wilsonville, OR", "Winchester Bay, OR", "Winston, OR", "Woodburn, OR",
 "Yachats, OR", "Yamhill, OR", "Yoncalla, OR", "Zigzag, OR",
 "Aaronsburg, PA", "Abbottstown, PA", "Acme, PA", "Adah, PA",
 "Adamsburg, PA", "Adamstown, PA", "Adamsville, PA", "Addison, PA",
 "Adrian, PA", "Airville, PA", "Akron, PA", "Albrightsville, PA",
 "Alburtis, PA", "Aldenville, PA", "Aleppo, PA", "Allegheny, PA",
 "Allenport, PA", "Allensville, PA", "Allentown, PA", "Allenwood, PA",
 "Allison, PA", "Allison Park, PA", "Allport, PA", "Altoona, PA",

"Alum Bank, PA", "Alverton, PA", "Amity, PA", "Andreas, PA",
"Anita, PA", "Annville, PA", "Apollo, PA", "Ardara, PA", "Ardmore, PA",
"Armagh, PA", "Armbrust, PA", "Arnold, PA", "Arona, PA", "Arsenal, PA",
"Artemas, PA", "Ashland, PA", "Ashley, PA", "Ashville, PA",
"Aspers, PA", "Aspinwall, PA", "Aston, PA", "Atglen, PA", "Athens, PA",
"Atlantic, PA", "Auburn, PA", "Aultman, PA", "Austin, PA", "Avella, PA",
"Avoca, PA", "Avondale, PA", "Avonmore, PA", "Baden, PA",
"Bainbridge, PA", "Bakerstown, PA", "Bala Cynwyd, PA", "Bally, PA",
"Barnesboro, PA", "Barnesville, PA", "Barree, PA", "Barto, PA",
"Bartonsville, PA", "Bath, PA", "Beach Lake, PA", "Beallsville, PA",
"Bear Lake, PA", "Beaver, PA", "Beaver Meadows, PA",
"Beaver Springs, PA", "Beavertown, PA", "Beccaria, PA",
"Bechtelsville, PA", "Bedford, PA", "Beech Creek, PA", "Belleville, PA",
"Bellevue, PA", "Bellwood, PA", "Benezett, PA", "Bensalem, PA",
"Bentleyville, PA", "Benton, PA", "Berlin, PA", "Bernville, PA",
"Berrysburg, PA", "Berwick, PA", "Berwyn, PA", "Bessemer, PA",
"Bethel, PA", "Bethel Park, PA", "Bethlehem, PA", "Bethton, PA",
"Big Cove Tannery, PA", "Biglerville, PA", "Bird In Hand, PA",
"Birdsboro, PA", "Black Lick, PA", "Blain, PA", "Blairs Mills, PA",
"Blairsville, PA", "Blakeslee, PA", "Blandon, PA", "Blawnox, PA",
"Bloomfield, PA", "Bloomsburg, PA", "Blossburg, PA",
"Blue Ridge Summi, PA", "Boalsburg, PA", "Boiling Springs, PA",
"Bolivar, PA", "Bon Aire, PA", "Boothwyn, PA", "Boston, PA",
"Boswell, PA", "Boyers, PA", "Boyertown, PA", "Brackenridge, PA",
"Brackney, PA", "Bradenville, PA", "Bradford, PA", "Bradfordwoods, PA",
"Branchdale, PA", "Breezewood, PA", "Breinigsville, PA",
"Brentwood, PA", "Bridgeport, PA", "Bridgeville, PA", "Brisbin, PA",
"Broad Top, PA", "Brockport, PA", "Brockton, PA", "Brockway, PA",
"Brodbecks, PA", "Brodheadsville, PA", "Brogue, PA", "Brookhaven, PA",
"Brookline, PA", "Broomall, PA", "Browndale, PA", "Bruin, PA",
"Brunnerville, PA", "Brush Valley, PA", "Bryn Mawr, PA",
"Buena Vista, PA", "Buffalo Mills, PA", "Bulger, PA", "Burnham, PA",
"Burnside, PA", "Burnt Cabins, PA", "Bushkill, PA", "Butztown, PA",
"Byrnedale, PA", "Cadogan, PA", "Cairnbrook, PA", "California, PA",
"Callensburg, PA", "Calvin, PA", "Cambridge Spring, PA", "Cammal, PA",
"Canadensis, PA", "Canton, PA", "Carlisle Barrack, PA", "Carlton, PA",
"Carmichaels, PA", "Carnegie, PA", "Carrolltown, PA", "Carson, PA",
"Carversville, PA", "Cassville, PA", "Caste Village, PA",
"Castle Shannon, PA", "Catasauqua, PA", "Catawissa, PA", "Cecil, PA",
"Cedar Run, PA", "Cedarhurst, PA", "Center Moreland, PA",
"Center Valley, PA", "Centerville, PA", "Central City, PA",
"Centralia, PA", "Centre Hall, PA", "Chadds Ford, PA", "Chalfont, PA",
"Chambersburg, PA", "Champion, PA", "Charleroi, PA", "Cheltenham, PA",
"Cherry Tree, PA", "Cherryville, PA", "Chester, PA",
"Chester Springs, PA", "Cheswick, PA", "Cheyney, PA", "Chicora, PA",
"Clarence, PA", "Clarendon, PA", "Clarington, PA", "Clarion, PA",
"Clarks Mills, PA", "Clarks Summit, PA", "Clarksburg, PA",
"Clarksville, PA", "Claysburg, PA", "Claysville, PA", "Clearfield, PA",
"Clearville, PA", "Cleona, PA", "Clinton, PA", "Clintonville, PA",
"Clymer, PA", "Coal Center, PA", "Coaldale, PA", "Coalport, PA",
"Coatesville, PA", "Coburn, PA", "Cochran, PA", "Cochranville, PA",
"Cocolamus, PA", "Cogan Station, PA", "Cokeburg, PA",
"College Misericord, PA", "Collegeville, PA", "Collingdale, PA",
"Colmar, PA", "Colonial Park, PA", "Columbia, PA",
"Columbia Cross R, PA", "Columbus, PA", "Colver, PA", "Commodore, PA",

"Concord, PA", "Conestoga, PA", "Conneaut Lake, PA",
 "Conneautville, PA", "Conway, PA", "Cooksburg, PA", "Coolspring, PA",
 "Coopersburg, PA", "Cooperstown, PA", "Coplay, PA", "Corliss, PA",
 "Corry, PA", "Corsica, PA", "Covington, PA", "Cowansville, PA",
 "Crafton, PA", "Craigsville, PA", "Cranberry, PA", "Cranesville, PA",
 "Creekside, PA", "Creighton, PA", "Cresco, PA", "Cresson, PA",
 "Cressona, PA", "Crosby, PA", "Cross Fork, PA", "Croydon, PA",
 "Crum Lynne, PA", "Crystal Spring, PA", "Cuddy, PA", "Curwensville, PA",
 "Daisytown, PA", "Dalmatia, PA", "Dalton, PA", "Damascus, PA",
 "Danielsville, PA", "Danville, PA", "Darlington, PA", "Darragh, PA",
 "Dauphin, PA", "Davidsville, PA", "Davistown, PA", "Dawson, PA",
 "Dayton, PA", "Delano, PA", "Delaware Water G, PA", "Delmont, PA",
 "Delta, PA", "Denver, PA", "Derrick City, PA", "Derry, PA", "Devon, PA",
 "Dickson City, PA", "Dilliner, PA", "Dillsburg, PA",
 "Dingmans Ferry, PA", "Donaldson, PA", "Donegal, PA", "Donora, PA",
 "Dornsife, PA", "Douglassville, PA", "Dover, PA", "Downingtown, PA",
 "Downtown, PA", "Doylesburg, PA", "Dravosburg, PA", "Dresher, PA",
 "Driftwood, PA", "Drumore, PA", "Drums, PA", "Dry Run, PA",
 "Du Bois, PA", "Dublin, PA", "Dudley, PA", "Duke Center, PA",
 "Dunbar, PA", "Duncannon, PA", "Duncansville, PA", "Dunlevy, PA",
 "Dunmore, PA", "Duquesne, PA", "Duryea, PA", "Dushore, PA",
 "Dysart, PA", "Eagleville, PA", "East Berlin, PA", "East Brady, PA",
 "East Earl, PA", "East Freedom, PA", "East Greenville, PA",
 "East Hickory, PA", "East Liberty, PA", "East Mc Keesport, PA",
 "East Millsboro, PA", "East Petersburg, PA", "East Pittsburgh, PA",
 "East Smithfield, PA", "East Springfield, PA", "East Stroudsburg, PA",
 "East Waterford, PA", "East York, PA", "Eau Claire, PA",
 "Ebensburg, PA", "Edinboro, PA", "Edinburg, PA", "Effort, PA",
 "Eighty Four, PA", "Elco, PA", "Eldred, PA", "Elizabeth, PA",
 "Elizabethtown, PA", "Elizabethville, PA", "Elkins Park, PA",
 "Elkland, PA", "Elliottsburg, PA", "Ellport, PA", "Ellsworth, PA",
 "Elverson, PA", "Elysburg, PA", "Emerald, PA", "Emlenton, PA",
 "Emmaus, PA", "Emporium, PA", "Enola, PA", "Enon Valley, PA",
 "Ephrata, PA", "Equinunk, PA", "Erie, PA", "Ernest, PA", "Erwinna, PA",
 "Espyville, PA", "Etna, PA", "Etters, PA", "Evans City, PA",
 "Evansville, PA", "Everett, PA", "Everson, PA", "Excelsior, PA",
 "Export, PA", "Exton, PA", "Eynon, PA", "Factoryville, PA",
 "Fairchance, PA", "Fairless Hills, PA", "Fairmount City, PA",
 "Fairoaks, PA", "Fairview, PA", "Fallentimber, PA", "Falls, PA",
 "Falls Creek, PA", "Fannettsburg, PA", "Farmington, PA", "Farrell, PA",
 "Fawn Grove, PA", "Fayette City, PA", "Fayetteville, PA",
 "Feasterville Tre, PA", "Felton, PA", "Fenelon, PA", "Finleyville, PA",
 "Fisher, PA", "Flinton, PA", "Florin, PA", "Flourtown, PA",
 "Fogelsville, PA", "Folcroft, PA", "Folsom, PA", "Fombell, PA",
 "Ford City, PA", "Forks Township, PA", "Forksville, PA",
 "Fort Hill, PA", "Fort Littleton, PA", "Fort Loudon, PA",
 "Fort Washington, PA", "Fountainville, PA", "Foxburg, PA",
 "Frackville, PA", "Franklin, PA", "Frazer, PA", "Frederick, PA",
 "Fredericksburg, PA", "Fredericktown, PA", "Fredonia, PA",
 "Freeburg, PA", "Freedom, PA", "Freeland, PA", "Freeport, PA",
 "Frenchville, PA", "Friedens, PA", "Friendsville, PA", "Fryburg, PA",
 "Furlong, PA", "Gaines, PA", "Galeton, PA", "Gallitzin, PA",
 "Ganister, PA", "Gap, PA", "Gardners, PA", "Garrett, PA", "Genesee, PA",
 "George School, PA", "Georgetown, PA", "Germansville, PA",
 "Gettysburg, PA", "Gibbon Glade, PA", "Gibsonia, PA", "Gifford, PA",

"Gilbert, PA", "Gilbertsville, PA", "Gillett, PA", "Girard, PA",
 "Girardville, PA", "Gladwyne, PA", "Glassport, PA", "Glen Campbell, PA",
 "Glen Hope, PA", "Glen Lyon, PA", "Glen Mills, PA", "Glen Richey, PA",
 "Glen Riddle Lima, PA", "Glen Rock, PA", "Glencoe, PA", "Glenmoore, PA",
 "Glenolden, PA", "Glenshaw, PA", "Glenside, PA", "Gordonville, PA",
 "Gouldsboro, PA", "Gowen City, PA", "Graceton, PA", "Grampian, PA",
 "Grand Valley, PA", "Grantville, PA", "Granville, PA",
 "Granville Summit, PA", "Grapeville, PA", "Grassflat, PA", "Gratz, PA",
 "Grays Landing, PA", "Graysville, PA", "Great Bend, PA", "Greeley, PA",
 "Green Lane, PA", "Green Park, PA", "Greencastle, PA", "Greensboro, PA",
 "Greensburg, PA", "Greenstone, PA", "Greentown, PA", "Grindstone, PA",
 "Grove City, PA", "Guys Mills, PA", "Gwynedd, PA", "Hadley, PA",
 "Halifax, PA", "Hallstead, PA", "Hamburg, PA", "Hamilton, PA",
 "Hamlin, PA", "Hampden, PA", "Hanover, PA", "Hanover Township, PA",
 "Harborcreek, PA", "Harford, PA", "Harleysville, PA", "Harmony, PA",
 "Harrisburg, PA", "Harrison City, PA", "Harrison Valley, PA",
 "Harrisonville, PA", "Harrisville, PA", "Hartstown, PA",
 "Harveys Lake, PA", "Harwick, PA", "Hastings, PA", "Hatboro, PA",
 "Hatfield, PA", "Haverford, PA", "Havertown, PA", "Hawk Run, PA",
 "Hawley, PA", "Hazelwood, PA", "Hazen, PA", "Hegins, PA", "Hellam, PA",
 "Hellertown, PA", "Henryville, PA", "Hereford, PA", "Herminie, PA",
 "Hermitage, PA", "Herndon, PA", "Herrick Center, PA", "Hershey, PA",
 "Hesston, PA", "Hickory, PA", "Highspire, PA", "Hiller, PA",
 "Hilliards, PA", "Hills Grove, PA", "Hilltown, PA", "Hokendauqua, PA",
 "Holbrook, PA", "Holland, PA", "Hollidaysburg, PA", "Hollsopple, PA",
 "Holmes, PA", "Holtwood, PA", "Home, PA", "Homewood, PA",
 "Honesdale, PA", "Honey Brook, PA", "Honey Grove, PA", "Hookstown, PA",
 "Hooversville, PA", "Hop Bottom, PA", "Hopewell, PA", "Hopwood, PA",
 "Horsham, PA", "Houston, PA", "Houtzdale, PA", "Howard, PA", "Huey, PA",
 "Hughesville, PA", "Hummelstown, PA", "Hunker, PA", "Hunlock Creek, PA",
 "Huntingdon, PA", "Huntingdon Valle, PA", "Huntington Mills, PA",
 "Hustontown, PA", "Hyde Park, PA", "Hyndman, PA", "Ickesburg, PA",
 "Imler, PA", "Imperial, PA", "Indian Head, PA", "Indiana, PA",
 "Indianola, PA", "Industry, PA", "Irvine, PA", "Irvona, PA",
 "Jackson, PA", "Jackson Center, PA", "Jacobus, PA", "James City, PA",
 "James Creek, PA", "Jamison, PA", "Jeannette, PA", "Jefferson, PA",
 "Jenners, PA", "Jessup, PA", "Jim Thorpe, PA", "Joffre, PA",
 "Johnsonburg, PA", "Johnstown, PA", "Jones Mills, PA", "Jonestown, PA",
 "Julian, PA", "Kane, PA", "Karns City, PA", "Karthaus, PA", "Kaska, PA",
 "Kelton, PA", "Kempton, PA", "Kennerdell, PA", "Kennett Square, PA",
 "Kersey, PA", "Kilbuck, PA", "King Of Prussia, PA", "Kingsley, PA",
 "Kingston, PA", "Kintnersville, PA", "Kinzers, PA", "Kirkwood, PA",
 "Kittanning, PA", "Klingerstown, PA", "Knox, PA", "Knoxville, PA",
 "Kossuth, PA", "Kresgeville, PA", "Kulpmont, PA", "Kunkletown, PA",
 "Kutztown, PA", "La Belle, PA", "La Jose, PA", "Laceyville, PA",
 "Lackawaxen, PA", "Lafayette Hill, PA", "Lairdsville, PA",
 "Lake Ariel, PA", "Lake City, PA", "Lake Como, PA", "Lake Harmony, PA",
 "Lake Lynn, PA", "Lakeville, PA", "Lakewood, PA", "Lamberton, PA",
 "Lancaster, PA", "Landenberg, PA", "Landisburg, PA", "Lansdale, PA",
 "Lansford, PA", "Large, PA", "Larimer, PA", "Latrobe, PA",
 "Laughlintown, PA", "Laurelton, PA", "Lawrence, PA",
 "Lawrenceville, PA", "Lawton, PA", "Layton, PA", "Le Raysville, PA",
 "Leck Kill, PA", "Leechburg, PA", "Leeper, PA", "Leesport, PA",
 "Leetsdale, PA", "Lemont Furnace, PA", "Lenhartsville, PA",
 "Lenoxville, PA", "Leola, PA", "Lester, PA", "Level Green, PA",

"Levittown, PA", "Lewis Run, PA", "Lewisberry, PA", "Lewisburg, PA",
"Lewistown, PA", "Liberty, PA", "Library, PA", "Lickingville, PA",
"Lilly, PA", "Limerick, PA", "Limestone, PA", "Lincoln Universi, PA",
"Linden, PA", "Line Lexington, PA", "Listonburg, PA",
"Little Marsh, PA", "Little Meadows, PA", "Littlestown, PA",
"Liverpool, PA", "Lock Haven, PA", "Loganton, PA", "Long Pond, PA",
"Lopez, PA", "Loretto, PA", "Loyalhanna, PA", "Loysburg, PA",
"Loysville, PA", "Lucernemines, PA", "Lucinda, PA", "Ludlow, PA",
"Lumberville, PA", "Lundys Lane, PA", "Lurgan, PA", "Luthersburg, PA",
"Luzerne, PA", "Lykens, PA", "Lyndora, PA", "Macarthur, PA",
"Macungie, PA", "Madera, PA", "Madison, PA", "Madisonburg, PA",
"Mahanoy City, PA", "Mainesburg, PA", "Manchester, PA", "Manheim, PA",
"Manns Choice, PA", "Manor, PA", "Manorville, PA", "Mansfield, PA",
"Maple Glen, PA", "Mapleton Depot, PA", "Marble, PA", "Marchand, PA",
"Marianna, PA", "Marienville, PA", "Marietta, PA", "Marion Center, PA",
"Marion Heights, PA", "Markleton, PA", "Markleysburg, PA", "Mars, PA",
"Marsteller, PA", "Martinsburg, PA", "Marwood, PA", "Marysville, PA",
"Matamoras, PA", "Mather, PA", "Mayfield, PA", "Mayport, PA",
"Mc Alisterville, PA", "Mc Clure, PA", "Mc Connellsburg, PA",
"Mc Donald, PA", "Mc Gees Mills, PA", "Mc Grann, PA", "Mc Kean, PA",
"Mc Kees Rocks, PA", "Mc Keesport, PA", "Mc Knight, PA",
"Mc Murray, PA", "Mc Sherrystown, PA", "Mc Veytown, PA", "Mcadoo, PA",
"Meadowbrook, PA", "Meadville, PA", "Mechanicsville, PA",
"Mehoopany, PA", "Melcroft, PA", "Mercer, PA", "Mercersburg, PA",
"Merion Station, PA", "Merrittstown, PA", "Mertztown, PA",
"Meshoppen, PA", "Meyersdale, PA", "Middleburg, PA",
"Middlebury Cente, PA", "Middletown, PA", "Midland, PA", "Midway, PA",
"Mifflin, PA", "Mifflinburg, PA", "Mifflintown, PA", "Mifflinville, PA",
"Milan, PA", "Milanville, PA", "Mildred, PA", "Milford, PA",
"Mill Creek, PA", "Mill Hall, PA", "Mill Run, PA", "Millersburg, PA",
"Millerstown, PA", "Millersville, PA", "Millerton, PA", "Millheim, PA",
"Millmont, PA", "Millrift, PA", "Mills, PA", "Millvale, PA",
"Millville, PA", "Milroy, PA", "Milton, PA", "Mineral Point, PA",
"Minersville, PA", "Mocanaqua, PA", "Mohnton, PA", "Mohrsville, PA",
"Monaca, PA", "Monessen, PA", "Monongahela, PA", "Monroeton, PA",
"Monroeville, PA", "Mont Alto, PA", "Mont Clare, PA", "Montandon, PA",
"Montgomery, PA", "Montgomeryville, PA", "Montoursville, PA",
"Montrose, PA", "Moon Twp, PA", "Moosic, PA", "Morgan, PA",
"Morgantown, PA", "Morris, PA", "Morris Run, PA", "Morrisdale, PA",
"Morton, PA", "Moscow, PA", "Moshannon, PA", "Mount Bethel, PA",
"Mount Carmel, PA", "Mount Holly Spri, PA", "Mount Jewett, PA",
"Mount Lebanon, PA", "Mount Oliver, PA", "Mount Penn, PA",
"Mount Pleasant, PA", "Mount Pleasant M, PA", "Mount Pocono, PA",
"Mount Union, PA", "Mount Washington, PA", "Mount Wolf, PA",
"Mountain Top, PA", "Mountville, PA", "Muir, PA", "Muncy, PA",
"Muncy Valley, PA", "Munhall, PA", "Munson, PA", "Murrysville, PA",
"Myerstown, PA", "Nanticoke, PA", "Nanty Glo, PA", "Narberth, PA",
"Narvon, PA", "Natrona, PA", "Nazareth, PA", "Needmore, PA",
"Neelyton, PA", "Neffsville, PA", "Nelson, PA", "Nescopeck, PA",
"Neshannock, PA", "Nesquehoning, PA", "Neville Island, PA",
"New Albany, PA", "New Alexandria, PA", "New Berlin, PA",
"New Bethlehem, PA", "New Bloomfield, PA", "New Brighton, PA",
"New Britain, PA", "New Castle, PA", "New Columbia, PA",
"New Cumberland, PA", "New Derry, PA", "New Eagle, PA",
"New Enterprise, PA", "New Florence, PA", "New Freedom, PA",

"New Freeport, PA", "New Galilee, PA", "New Germantown, PA",
"New Holland, PA", "New Hope, PA", "New Milford, PA",
"New Millport, PA", "New Oxford, PA", "New Paris, PA", "New Park, PA",
"New Providence, PA", "New Ringgold, PA", "New Salem, PA",
"New Stanton, PA", "New Tripoli, PA", "New Wilmington, PA",
"Newburg, PA", "Newfoundland, PA", "Newmanstown, PA", "Newport, PA",
"Newtown Square, PA", "Newville, PA", "Nicholson, PA", "Nicktown, PA",
"Ninepoints, PA", "Nineveh, PA", "Noblestown, PA", "Normalville, PA",
"Norristown, PA", "North Bingham, PA", "North East, PA",
"North Huntingdon, PA", "North Versailles, PA", "North Wales, PA",
"North Warren, PA", "Northampton, PA", "Northpoint, PA",
"Northumberland, PA", "Norwood, PA", "Nottingham, PA", "Noxen, PA",
"Oakland, PA", "Oakland Mills, PA", "Oakmont, PA", "Observatory, PA",
"Ogontz Campus, PA", "Ohiopyle, PA", "Oil City, PA", "Olanta, PA",
"Old Forge, PA", "Oley, PA", "Oliveburg, PA", "Oliver, PA",
"Olyphant, PA", "Orangeville, PA", "Orbisonia, PA", "Orefield, PA",
"Oreland, PA", "Ormsby, PA", "Orrstown, PA", "Orrtanna, PA",
"Orviston, PA", "Orwigsburg, PA", "Osceola, PA", "Osceola Mills, PA",
"Oswayo, PA", "Ottsville, PA", "Oxford, PA", "Palm, PA",
"Palmerton, PA", "Palmyra, PA", "Paoli, PA", "Paradise, PA",
"Paris, PA", "Park, PA", "Parker, PA", "Parkesburg, PA", "Parkhill, PA",
"Parkway Center, PA", "Patton, PA", "Paupack, PA", "Paxinos, PA",
"Peach Bottom, PA", "Peckville, PA", "Pen Argyl, PA", "Penbrook, PA",
"Penfield, PA", "Penllyn, PA", "Penn, PA", "Penn Hills, PA",
"Penn Run, PA", "Penndel, PA", "Pennsburg, PA", "Pennsylvania Fur, PA",
"Pequea, PA", "Perkasie, PA", "Perkiomenville, PA", "Petersburg, PA",
"Petrolia, PA", "Philadelphia, PA", "Philipsburg, PA",
"Phoenixville, PA", "Pilgrim Gardens, PA", "Pine Grove, PA",
"Pipersville, PA", "Pitcairn, PA", "Pitman, PA", "Pittsfield, PA",
"Pittston, PA", "Pleasant Gap, PA", "Pleasant Hall, PA",
"Pleasant Mount, PA", "Pleasantville, PA", "Plum, PA", "Plymouth, PA",
"Plymouth Meeting, PA", "Pocono Lake, PA", "Pocono Pines, PA",
"Pocono Summit, PA", "Point Marion, PA", "Polk, PA",
"Port Allegany, PA", "Port Carbon, PA", "Port Clinton, PA",
"Port Matilda, PA", "Port Royal, PA", "Port Trevorton, PA",
"Portersville, PA", "Portland Mills, PA", "Pottersdale, PA",
"Pottsville, PA", "Presque Isle, PA", "Presto, PA", "Preston Park, PA",
"Primos Secane, PA", "Prompton, PA", "Prospect, PA",
"Prospect Park, PA", "Prosperity, PA", "Pulaski, PA",
"Punxsutawney, PA", "Puritan, PA", "Quakake, PA", "Quakertown, PA",
"Quarryville, PA", "Racine, PA", "Radnor, PA", "Ralston, PA",
"Ramey, PA", "Rankin, PA", "Ranshaw, PA", "Reading, PA",
"Rebersburg, PA", "Rebuck, PA", "Rector, PA", "Red Hill, PA",
"Red Lion, PA", "Reeders, PA", "Reedsville, PA", "Reinholds, PA",
"Renfrew, PA", "Renovo, PA", "Republic, PA", "Rew, PA",
"Reynoldsville, PA", "Rices Landing, PA", "Richboro, PA",
"Richfield, PA", "Richland, PA", "Richlandtown, PA", "Ridley Park, PA",
"Riegelsville, PA", "Rillton, PA", "Ringgold, PA", "Ringtown, PA",
"Riverside, PA", "Rixford, PA", "Roaring Branch, PA",
"Roaring Spring, PA", "Robertsdale, PA", "Robeson, PA",
"Robinson, PA", "Rochester, PA", "Rochester Mills, PA", "Rock Glen, PA",
"Rockton, PA", "Rockwood, PA", "Rogersville, PA", "Rohrerstown, PA",
"Rome, PA", "Ronks, PA", "Roscoe, PA", "Roseto, PA", "Rossiter, PA",
"Rostraver, PA", "Roulette, PA", "Ruffs Dale, PA", "Rural Valley, PA",
"Rushville, PA", "Russell, PA", "Russellton, PA", "Sabinsville, PA",

"Sacramento, PA", "Saegertown, PA", "Saint Benedict, PA",
 "Saint Clair, PA", "Saint Marys, PA", "Saint Michael, PA",
 "Saint Thomas, PA", "Salisbury, PA", "Salix, PA", "Salladasburg, PA",
 "Saltsburg, PA", "Salunga, PA", "Sanatoga, PA", "Sandy Lake, PA",
 "Sarver, PA", "Saxonburg, PA", "Saxton, PA", "Saylorsburg, PA",
 "Sayre, PA", "Scenery Hill, PA", "Schellsburg, PA", "Schnecksville, PA",
 "Schuylkill Haven, PA", "Schwenksville, PA", "Sciota, PA",
 "Scotrun, PA", "Scottsdale, PA", "Scranton, PA", "Seanor, PA",
 "Selinsgrove, PA", "Sellersville, PA", "Seneca, PA",
 "Seven Valleys, PA", "Seward, PA", "Sewickley, PA", "Shade Gap, PA",
 "Shadyside, PA", "Sharon, PA", "Sharon Hill, PA", "Sharpsville, PA",
 "Shavertown, PA", "Sheffield, PA", "Shelocta, PA", "Shenandoah, PA",
 "Shenango, PA", "Sheppton, PA", "Shermans Dale, PA", "Shillington, PA",
 "Shinglehouse, PA", "Shippensburg, PA", "Shippenville, PA",
 "Shippingport, PA", "Shiremanstown, PA", "Shirleysburg, PA",
 "Shoemakersville, PA", "Shohola, PA", "Shrewsbury, PA", "Shunk, PA",
 "Sidman, PA", "Sigel, PA", "Simpson, PA", "Sinking Spring, PA",
 "Sinnamahoning, PA", "Six Mile Run, PA", "Slickville, PA", "Sligo, PA",
 "Slippery Rock, PA", "Slovan, PA", "Smethport, PA", "Smicksburg, PA",
 "Smithfield, PA", "Smithmill, PA", "Smock, PA", "Smoketown, PA",
 "Snow Shoe, PA", "Snydertown, PA", "Somerset, PA",
 "South Connellsvi, PA", "South Fork, PA", "South Gibson, PA",
 "South Hills, PA", "South Sterling, PA", "South Williamsport, PA",
 "Spangler, PA", "Spartansburg, PA", "Spraggs, PA", "Sprinkle Mills, PA",
 "Spring Church, PA", "Spring City, PA", "Spring Creek, PA",
 "Spring Glen, PA", "Spring Grove, PA", "Spring House, PA",
 "Spring Mills, PA", "Spring Run, PA", "Springboro, PA",
 "Springdale, PA", "Springfield, PA", "Springs, PA", "Springville, PA",
 "Spruce Creek, PA", "Squirrel Hill, PA", "St Clairsville, PA",
 "Stahlstown, PA", "Star Junction, PA", "Starford, PA", "Starlight, PA",
 "Starrucca, PA", "State College, PA", "Steelton, PA", "Sterling, PA",
 "Stevens, PA", "Stevensville, PA", "Stewartstown, PA", "Stillwater, PA",
 "Stockdale, PA", "Stoneboro, PA", "Stoystown, PA", "Strabane, PA",
 "Strasburg, PA", "Strattanville, PA", "Strongstown, PA",
 "Stroudsburg, PA", "Sugar Grove, PA", "Sugar Run, PA", "Sugarloaf, PA",
 "Summerhill, PA", "Summerville, PA", "Summit Hill, PA", "Sunbury, PA",
 "Susquehanna, PA", "Sutersville, PA", "Swarthmore, PA", "Swatara, PA",
 "Sweet Valley, PA", "Swiftwater, PA", "Swissvale, PA", "Sycamore, PA",
 "Sykesville, PA", "Tafton, PA", "Tamaqua, PA", "Tamiment, PA",
 "Tannersville, PA", "Tarentum, PA", "Tarrs, PA", "Taylor, PA",
 "Telford, PA", "Temple, PA", "Templeton, PA", "Terre Hill, PA",
 "Thomasville, PA", "Thompson, PA", "Thompsons town, PA", "Thorndale, PA",
 "Thornton, PA", "Three Springs, PA", "Tidioute, PA", "Timblin, PA",
 "Tioga, PA", "Tionesta, PA", "Titusville, PA", "Tobyhanna, PA",
 "Todd, PA", "Topton, PA", "Toughkenamon, PA", "Towanda, PA",
 "Tower City, PA", "Townville, PA", "Transfer, PA", "Trevorton, PA",
 "Trexler town, PA", "Trout Run, PA", "Troy, PA", "Tullytown, PA",
 "Turbotville, PA", "Turtle Creek, PA", "Turtlepoint, PA",
 "Twin Rocks, PA", "Tyler Hill, PA", "Tyrone, PA", "Ulster, PA",
 "Ulysses, PA", "Union City, PA", "Union Dale, PA", "Uniontown, PA",
 "Unityville, PA", "Upper Black Eddy, PA", "Upper Darby, PA",
 "Upper Saint Clai, PA", "Upperstrasburg, PA", "Uptown, PA", "Utica, PA",
 "Valencia, PA", "Valier, PA", "Valley View, PA", "Van Meter, PA",
 "Vanderbilt, PA", "Venango, PA", "Venetia, PA", "Venus, PA",
 "Verona, PA", "Villanova, PA", "Vintondale, PA", "Volant, PA",

"Vowinckel, PA", "W Mifflin Fin, PA", "Wall, PA", "Wallingford, PA",
 "Walnut Bottom, PA", "Walnutport, PA", "Waltersburg, PA", "Wampum, PA",
 "Wapwallopen, PA", "Warfordsburg, PA", "Warminster, PA",
 "Warren Center, PA", "Warrendale, PA", "Warrington, PA",
 "Warriors Mark, PA", "Washington, PA", "Washington Boro, PA",
 "Washington Cross, PA", "Waterfall, PA", "Waterford, PA",
 "Waterville, PA", "Watsontown, PA", "Wattsburg, PA", "Waymart, PA",
 "Waynesboro, PA", "Waynesburg, PA", "Weatherly, PA", "Weedville, PA",
 "Weissport, PA", "Wells Tannery, PA", "Wellsboro, PA", "Wellsville, PA",
 "Wernersville, PA", "Wescosville, PA", "Wesleyville, PA",
 "West Alexander, PA", "West Brownsville, PA", "West Chester, PA",
 "West Conshohocke, PA", "West Decatur, PA", "West Finley, PA",
 "West Grove, PA", "West Hazleton, PA", "West Lawn, PA",
 "West Middlesex, PA", "West Newton, PA", "West Pittston, PA",
 "West Springfield, PA", "West Sunbury, PA", "West View, PA",
 "West York, PA", "Westford, PA", "Westmoreland Cit, PA", "Westover, PA",
 "Westport, PA", "Wexford, PA", "White, PA", "White Haven, PA",
 "White Oak, PA", "Wiconisco, PA", "Wilburton, PA", "Wilcox, PA",
 "Wilkes Barre, PA", "Wilkinsburg, PA", "Williamstown, PA",
 "Willow Grove Nas, PA", "Willow Hill, PA", "Willow Street, PA",
 "Wilpen, PA", "Winburne, PA", "Wind Gap, PA", "Wind Ridge, PA",
 "Windber, PA", "Windsor, PA", "Winfield, PA", "Womelsdorf, PA",
 "Woodbury, PA", "Woodland, PA", "Woodlyn, PA", "Woodward, PA",
 "Woolrich, PA", "Wormleysburg, PA", "Worthville, PA",
 "Wrightsville, PA", "Wyalusing, PA", "Wyncote, PA", "Wynnewood, PA",
 "Wyoming, PA", "Wyomissing, PA", "Wysox, PA", "Yardley, PA",
 "Yeadon, PA", "Yeagertown, PA", "Yoe, PA", "York, PA", "York Haven, PA",
 "York Springs, PA", "Youngsville, PA", "Youngwood, PA", "Yukon, PA",
 "Zelienople, PA", "Zieglersville, PA", "Zion Grove, PA",
 "Zionsville, PA", "Ashaway, RI", "Barrington, RI", "Block Island, RI",
 "Bradford, RI", "Bristol, RI", "Central Falls, RI", "Centredale, RI",
 "Charlestown, RI", "Chepachet, RI", "Clayville, RI", "Coventry, RI",
 "Cranston, RI", "Cumberland, RI", "East Greenwich, RI",
 "East Providence, RI", "Exeter, RI", "Foster, RI", "Greene, RI",
 "Greenville, RI", "Harrisville, RI", "Hope, RI", "Jamestown, RI",
 "Kingston, RI", "Lincoln, RI", "Little Compton, RI", "Manville, RI",
 "Middletown, RI", "Narragansett, RI", "North Kingstown, RI",
 "North Scituate, RI", "North Smithfield, RI", "Oakland, RI",
 "Pascoag, RI", "Pawtucket, RI", "Peace Dale, RI", "Portsmouth, RI",
 "Providence, RI", "Prudence Island, RI", "Richmond, RI",
 "Riverside, RI", "Rumford, RI", "Saunderstown, RI", "Slatersville, RI",
 "Slocum, RI", "Smithfield, RI", "Tiverton, RI", "Warren, RI",
 "Warwick, RI", "West Greenwich, RI", "West Warwick, RI", "Westerly, RI",
 "Wood River Junct, RI", "Abbeville, SC", "Aiken, SC", "Alcolu, SC",
 "Allendale, SC", "Alvin, SC", "Anderson, SC", "Andrews, SC",
 "Awendaw, SC", "Aynor, SC", "Bamberg, SC", "Barnwell, SC",
 "Batesburg, SC", "Beech Island, SC", "Belton, SC", "Bennettsville, SC",
 "Bethune, SC", "Bishopville, SC", "Blackstock, SC", "Blackville, SC",
 "Blair, SC", "Blenheim, SC", "Bluffton, SC", "Blythewood, SC",
 "Bonneau, SC", "Bowman, SC", "Bradley, SC", "Branchville, SC",
 "Brunson, SC", "Bucksport, SC", "Buffalo, SC", "Burton, SC",
 "Cades, SC", "Calhoun Falls, SC", "Camden, SC", "Cameron, SC",
 "Campobello, SC", "Carlisle, SC", "Cassatt, SC", "Catawba, SC",
 "Cateechee, SC", "Cayce, SC", "Central, SC", "Chapin, SC",
 "Chappells, SC", "Charleston, SC", "Cheraw, SC", "Cherokee Falls, SC",

"Cherry Grove Bea, SC", "Chesnee, SC", "Chester, SC",
"Chesterfield, SC", "Clarks Hill, SC", "Clemson, SC", "Cleveland, SC",
"Clinton, SC", "Clio, SC", "Columbia, SC", "Conway, SC",
"Coosawatchie, SC", "Cope, SC", "Cordesville, SC", "Cordova, SC",
"Cottageville, SC", "Coward, SC", "Cowpens, SC", "Cross, SC",
"Cross Hill, SC", "Dalzell, SC", "Darlington, SC", "Denmark, SC",
"Dillon, SC", "Dorchester, SC", "Due West, SC", "Duncan, SC",
"Early Branch, SC", "Easley, SC", "Eastover, SC", "Edgefield, SC",
"Edgemoor, SC", "Edisto Island, SC", "Effingham, SC", "Ehrhardt, SC",
"Elgin, SC", "Elliott, SC", "Elloree, SC", "Enoree, SC", "Estill, SC",
"Eutawville, SC", "Fair Play, SC", "Fairfax, SC", "Florence, SC",
"Fork, SC", "Fort Lawn, SC", "Fort Motte, SC", "Fountain Inn, SC",
"Gable, SC", "Gadsden, SC", "Gaffney, SC", "Galivants Ferry, SC",
"Garnett, SC", "Gaston, SC", "Georgetown, SC", "Gilbert, SC",
"Glenn Springs, SC", "Graniteville, SC", "Great Falls, SC",
"Greeleyville, SC", "Green Pond, SC", "Green Sea, SC", "Greenville, SC",
"Greenwood, SC", "Greer, SC", "Gresham, SC", "Hampton, SC",
"Hardeeville, SC", "Harleyville, SC", "Hartsville, SC",
"Heath Springs, SC", "Hemingway, SC", "Hickory Grove, SC",
"Hilton Head Isla, SC", "Hodges, SC", "Holly Hill, SC",
"Honea Path, SC", "Hopkins, SC", "Huger, SC", "Inman, SC", "Irmo, SC",
"Islandton, SC", "Isle Of Palms, SC", "Iva, SC", "Jackson, SC",
"Jefferson, SC", "Jenkinsville, SC", "Jericho, SC", "Joanna, SC",
"Johns Island, SC", "Johnsonville, SC", "Johnston, SC", "Kelton, SC",
"Kershaw, SC", "Kinards, SC", "Kingstree, SC", "Ladson, SC",
"Lake City, SC", "Lake View, SC", "Lake Wylie, SC", "Lamar, SC",
"Lancaster, SC", "Landrum, SC", "Lane, SC", "Latta, SC",
"Leesville, SC", "Lexington, SC", "Liberty, SC", "Little Mountain, SC",
"Little River, SC", "Little Rock, SC", "Lodge, SC", "Lone Star, SC",
"Long Creek, SC", "Longs, SC", "Loris, SC", "Lowndesville, SC",
"Lugoff, SC", "Luray, SC", "Lyman, SC", "Lynchburg, SC", "Madison, SC",
"Marietta, SC", "Marion, SC", "Martin, SC", "Mauldin, SC", "Mc Bee, SC",
"Mc Clellanville, SC", "Mc Coll, SC", "Mc Connells, SC",
"Mc Cormick, SC", "Meggett, SC", "Modoc, SC", "Monetta, SC",
"Moore, SC", "Mount Carmel, SC", "Mount Croghan, SC", "Mount Holly, SC",
"Mount Pleasant, SC", "Mountain Rest, SC", "Mountville, SC",
"Mullins, SC", "Murrells Inlet, SC", "Myrtle Beach, SC", "Neeses, SC",
"Nesmith, SC", "New Ellenton, SC", "New Zion, SC", "Newberry, SC",
"Nichols, SC", "Ninety Six, SC", "North, SC", "North Charleston, SC",
"Norway, SC", "Oakley, SC", "Olanta, SC", "Olar, SC", "Ora, SC",
"Orangeburg, SC", "Oswego, SC", "Pacolet, SC", "Pageland, SC",
"Pamplico, SC", "Patrick, SC", "Pawleys Island, SC", "Paxville, SC",
"Pelion, SC", "Pelzer, SC", "Pendleton, SC", "Pickens, SC",
"Piedmont, SC", "Pineland, SC", "Pineville, SC", "Pinewood, SC",
"Pinopolis, SC", "Plum Branch, SC", "Pomaria, SC", "Port Royal, SC",
"Prosperity, SC", "Quinby, SC", "Ravenel, SC", "Reevesville, SC",
"Rembert, SC", "Richburg, SC", "Ridge Spring, SC", "Ridgeville, SC",
"Ridgeway, SC", "Rimini, SC", "Ritter, SC", "Rock Hill, SC",
"Roebuck, SC", "Round O, SC", "Rowesville, SC", "Ruby, SC",
"Ruffin, SC", "Saint Charles, SC", "Saint George, SC", "Salem, SC",
"Salley, SC", "Salters, SC", "Saluda, SC", "Santee, SC", "Scranton, SC",
"Seabrook, SC", "Sellers, SC", "Seneca, SC", "Sharon, SC",
"Shaw A F B, SC", "Shoals Junction, SC", "Shulerville, SC",
"Silverstreet, SC", "Simpsonville, SC", "Six Mile, SC", "Smoaks, SC",
"Smyrna, SC", "Society Hill, SC", "South Of The Bor, SC",

"Spartanburg, SC", "Springfield, SC", "St Helena Island, SC",
 "Starr, SC", "Sullivans Island, SC", "Summerton, SC", "Summerville, SC",
 "Sumter, SC", "Sunset, SC", "Surfside Beach, SC", "Swansea, SC",
 "Tamassee, SC", "Taylors, SC", "Tega Cay, SC", "Tigerville, SC",
 "Tillman, SC", "Timmonsville, SC", "Townville, SC",
 "Travelers Rest, SC", "Trenton, SC", "Troy, SC", "Turbeville, SC",
 "Ulmer, SC", "Union, SC", "Valley Falls, SC", "Vance, SC",
 "Varnville, SC", "Wadmalaw Island, SC", "Wagener, SC", "Walhalla, SC",
 "Wallace, SC", "Wando, SC", "Ward, SC", "Ware Shoals, SC",
 "Warrenville, SC", "Waterloo, SC", "Wedgefield, SC", "Wellford, SC",
 "West Columbia, SC", "West Union, SC", "Westville, SC", "Whitmire, SC",
 "Williamston, SC", "Williston, SC", "Windsor, SC", "Winnsboro, SC",
 "Woodruff, SC", "Yemassee, SC", "York, SC", "Aberdeen, SD",
 "Academy, SD", "Agar, SD", "Agency Village, SD", "Akaska, SD",
 "Albee, SD", "Alcester, SD", "Allen, SD", "Alpena, SD", "Altamont, SD",
 "Amherst, SD", "Andover, SD", "Arlington, SD", "Armour, SD",
 "Artas, SD", "Astoria, SD", "Athol, SD", "Aurora, SD", "Avon, SD",
 "Badger, SD", "Baltic, SD", "Bancroft, SD", "Barnard, SD", "Bath, SD",
 "Belle Fourche, SD", "Belvidere, SD", "Bemis, SD", "Beresford, SD",
 "Bethlehem, SD", "Big Stone City, SD", "Bison, SD", "Black Hawk, SD",
 "Blunt, SD", "Bonesteel, SD", "Bowdle, SD", "Box Elder, SD",
 "Bradley, SD", "Brandt, SD", "Brentford, SD", "Britton, SD",
 "Brookings, SD", "Bruce, SD", "Bryant, SD", "Buffalo, SD",
 "Buffalo Gap, SD", "Buffalo Ridge, SD", "Burbank, SD", "Butler, SD",
 "Canistota, SD", "Canova, SD", "Canton, SD", "Caputa, SD",
 "Carpenter, SD", "Carter, SD", "Carthage, SD", "Castlewood, SD",
 "Cavour, SD", "Cedarbutte, SD", "Centerville, SD", "Chamberlain, SD",
 "Chancellor, SD", "Cherry Creek, SD", "Chester, SD", "Claire City, SD",
 "Claremont, SD", "Clark, SD", "Clearfield, SD", "Colman, SD",
 "Colome, SD", "Colton, SD", "Columbia, SD", "Corona, SD", "Corsica, SD",
 "Corson, SD", "Cottonwood, SD", "Crazy Horse, SD", "Creighton, SD",
 "Cresbard, SD", "Crooks, SD", "Dakota Dunes, SD", "Dallas, SD",
 "Dante, SD", "Davis, SD", "De Smet, SD", "Deadwood, SD",
 "Dell Rapids, SD", "Delmont, SD", "Dempster, SD", "Denby, SD",
 "Dimock, SD", "Dixon, SD", "Doland, SD", "Dolton, SD", "Draper, SD",
 "Dupree, SD", "Eden, SD", "Edgemont, SD", "Egan, SD", "Elk Point, SD",
 "Elkton, SD", "Ellsworth Afb, SD", "Elm Springs, SD", "Emery, SD",
 "Enning, SD", "Erwin, SD", "Ethan, SD", "Fairburn, SD", "Fairfax, SD",
 "Fairview, SD", "Faith, SD", "Farmer, SD", "Fedora, SD",
 "Firesteel, SD", "Flandreau, SD", "Florence, SD", "Forestburg, SD",
 "Fort Meade, SD", "Fort Pierre, SD", "Fort Thompson, SD",
 "Frankfort, SD", "Frederick, SD", "Freeman, SD", "Fruitdale, SD",
 "Fulton, SD", "Gann Valley, SD", "Garden City, SD", "Garretson, SD",
 "Gary, SD", "Gayville, SD", "Geddes, SD", "Gettysburg, SD",
 "Glad Valley, SD", "Glencross, SD", "Glenham, SD", "Grenville, SD",
 "Groton, SD", "Hamill, SD", "Harrisburg, SD", "Harrison, SD",
 "Harrold, SD", "Hartford, SD", "Hayes, SD", "Hayti, SD", "Hazel, SD",
 "Hecla, SD", "Henry, SD", "Hereford, SD", "Hermosa, SD", "Herreid, SD",
 "Herrick, SD", "Hetland, SD", "Highmore, SD", "Hill City, SD",
 "Hitchcock, SD", "Holabird, SD", "Hosmer, SD", "Hot Springs, SD",
 "Houghton, SD", "Hoven, SD", "Hudson, SD", "Humboldt, SD", "Hurley, SD",
 "Huron, SD", "Ideal, SD", "Interior, SD", "Iona, SD", "Ipswich, SD",
 "Irene, SD", "Iroquois, SD", "Isabel, SD", "Java, SD", "Jefferson, SD",
 "Kadoka, SD", "Kaylor, SD", "Keldron, SD", "Kennebec, SD",
 "Keyapaha, SD", "Keystone, SD", "Kimball, SD", "Kranzburg, SD",

"Kyle, SD", "Labolt, SD", "Lake Andes, SD", "Lake City, SD",
 "Lake Norden, SD", "Lake Preston, SD", "Lane, SD", "Langford, SD",
 "Lebanon, SD", "Lemmon, SD", "Lennox, SD", "Leola, SD",
 "Lesterville, SD", "Letcher, SD", "Lily, SD", "Lodgepole, SD",
 "Long Valley, SD", "Longlake, SD", "Loomis, SD", "Lower Brule, SD",
 "Lucas, SD", "Ludlow, SD", "Madison, SD", "Mahto, SD", "Manderson, SD",
 "Mansfield, SD", "Marcus, SD", "Marion, SD", "Marty, SD", "Marvin, SD",
 "Mc Intosh, SD", "Mc Laughlin, SD", "Meadow, SD", "Meckling, SD",
 "Mellette, SD", "Menno, SD", "Milbank, SD", "Milesville, SD",
 "Miller, SD", "Mina, SD", "Miranda, SD", "Mission, SD",
 "Mission Hill, SD", "Mission Ridge, SD", "Mobridge, SD", "Monroe, SD",
 "Montrose, SD", "Morristown, SD", "Mound City, SD", "Mount Vernon, SD",
 "Mud Butte, SD", "Murdo, SD", "Nemo, SD", "New Effington, SD",
 "New Holland, SD", "New Underwood, SD", "Newell, SD", "Nisland, SD",
 "Norris, SD", "Northville, SD", "Nunda, SD", "Oelrichs, SD",
 "Okaton, SD", "Oldham, SD", "Olivet, SD", "Onaka, SD", "Onida, SD",
 "Opal, SD", "Oral, SD", "Orient, SD", "Ortley, SD", "Ottumwa, SD",
 "Owanka, SD", "Parade, SD", "Parker, SD", "Parkston, SD",
 "Parmelee, SD", "Peever, SD", "Philip, SD", "Piedmont, SD",
 "Pierpont, SD", "Pierre, SD", "Pine Ridge, SD", "Plainview, SD",
 "Plankinton, SD", "Pollock, SD", "Porcupine, SD", "Prairie City, SD",
 "Presho, SD", "Provo, SD", "Pukwana, SD", "Ralph, SD", "Ramona, SD",
 "Ravinia, SD", "Raymond, SD", "Redfield, SD", "Redowl, SD",
 "Ree Heights, SD", "Reliance, SD", "Renner, SD", "Reva, SD",
 "Rochford, SD", "Rockerville, SD", "Rockham, SD", "Roscoe, SD",
 "Rosholt, SD", "Roslyn, SD", "Roswell, SD", "Rutland, SD",
 "Saint Charles, SD", "Saint Francis, SD", "Saint Lawrence, SD",
 "Saint Onge, SD", "Salem, SD", "Scenic, SD", "Scotland, SD",
 "Selby, SD", "Seneca, SD", "Shadehill, SD", "Sherman, SD",
 "Silver City, SD", "Sinai, SD", "Sioux Falls, SD", "Sky Ranch, SD",
 "Smithwick, SD", "South Shore, SD", "Spearfish, SD",
 "Spearfish Canyon, SD", "Spencer, SD", "Springfield, SD",
 "Stickney, SD", "Stockholm, SD", "Stoneville, SD", "Strandburg, SD",
 "Stratford, SD", "Summit, SD", "Tabor, SD", "Tea, SD",
 "Timber Lake, SD", "Tolstoy, SD", "Toronto, SD", "Trail City, SD",
 "Trent, SD", "Tripp, SD", "Tulare, SD", "Turton, SD", "Tuthill, SD",
 "Twin Brooks, SD", "Tyndall, SD", "Utica, SD", "Vale, SD",
 "Valley Springs, SD", "Veblen, SD", "Verdon, SD", "Vermillion, SD",
 "Vetal, SD", "Viborg, SD", "Vienna, SD", "Virgil, SD", "Vivian, SD",
 "Volga, SD", "Volin, SD", "Wagner, SD", "Wakonda, SD", "Wakpala, SD",
 "Wall, SD", "Wallace, SD", "Wanblee, SD", "Ward, SD", "Warner, SD",
 "Wasta, SD", "Watauga, SD", "Watertown, SD", "Waubay, SD",
 "Waverly, SD", "Wentworth, SD", "Wessington, SD",
 "Wessington Sprin, SD", "Wetonka, SD", "Wewela, SD", "White, SD",
 "White Lake, SD", "White Owl, SD", "White River, SD", "Whitewood, SD",
 "Willow Lake, SD", "Wilmot, SD", "Winfred, SD", "Witten, SD",
 "Wolsey, SD", "Wood, SD", "Woonsocket, SD", "Worthing, SD",
 "Wounded Knee, SD", "Yale, SD", "Yankton, SD", "Zell, SD", "Zeona, SD",
 "Adams, TN", "Adamsville, TN", "Afton, TN", "Alamo, TN", "Alcoa, TN",
 "Alexandria, TN", "Algood, TN", "Allardt, TN", "Allons, TN",
 "Allred, TN", "Alpine, TN", "Altamont, TN", "Andersonville, TN",
 "Antioch, TN", "Apison, TN", "Ardmore, TN", "Arlington, TN",
 "Arrington, TN", "Ashland City, TN", "Athens, TN", "Atoka, TN",
 "Atwood, TN", "Auburntown, TN", "Baileyton, TN", "Baneberry, TN",
 "Bartlett, TN", "Bath Springs, TN", "Baxter, TN", "Bean Station, TN",

"Beech Bluff, TN", "Beechgrove, TN", "Beersheba Spring, TN",
 "Belfast, TN", "Bell Buckle, TN", "Bellevue, TN", "Bells, TN",
 "Belvidere, TN", "Benton, TN", "Bethel Springs, TN", "Bethpage, TN",
 "Big Rock, TN", "Big Sandy, TN", "Birchwood, TN", "Blaine, TN",
 "Bloomington, TN", "Bloomington Spri, TN", "Blountville, TN",
 "Bluff City, TN", "Bolivar, TN", "Bon Aqua, TN", "Bone Cave, TN",
 "Bradford, TN", "Bradyville, TN", "Brentwood, TN", "Brighton, TN",
 "Bristol, TN", "Brownsville, TN", "Bruceton, TN", "Brush Creek, TN",
 "Buchanan, TN", "Buena Vista, TN", "Buffalo Valley, TN",
 "Bulls Gap, TN", "Bumpus Mills, TN", "Burlison, TN", "Burns, TN",
 "Butler, TN", "Bybee, TN", "Byrdstown, TN", "Calhoun, TN", "Camden, TN",
 "Caryville, TN", "Castalian Spring, TN", "Cedar Grove, TN",
 "Cedar Hill, TN", "Celina, TN", "Centerville, TN", "Chapel Hill, TN",
 "Chapmansboro, TN", "Charleston, TN", "Charlotte, TN",
 "Chattanooga, TN", "Chestnut Mound, TN", "Christiana, TN",
 "Chuckey, TN", "Church Hill, TN", "Clairfield, TN", "Clarkrange, TN",
 "Clarksville, TN", "Cleveland, TN", "Clifton, TN", "Clinton, TN",
 "Coalmont, TN", "College Grove, TN", "Collierville, TN",
 "Collinwood, TN", "Colonial Heights, TN", "Columbia, TN", "Concord, TN",
 "Concord Farragut, TN", "Cordova, TN", "Cornersville, TN",
 "Corryton, TN", "Cosby, TN", "Cottage Grove, TN", "Cottontown, TN",
 "Counce, TN", "Covington, TN", "Cowan, TN", "Crab Orchard, TN",
 "Crawford, TN", "Cross Plains, TN", "Crump, TN", "Culleoka, TN",
 "Cumberland City, TN", "Cumberland Furna, TN", "Cumberland Gap, TN",
 "Cunningham, TN", "Cypress Inn, TN", "Dandridge, TN", "Darden, TN",
 "Dayton, TN", "Decatur, TN", "Decaturville, TN", "Decherd, TN",
 "Deer Lodge, TN", "Defeated, TN", "Del Rio, TN", "Delano, TN",
 "Denmark, TN", "Devonia, TN", "Dickel, TN", "Dickson, TN",
 "Dixon Springs, TN", "Dover, TN", "Dowelltown, TN", "Doyle, TN",
 "Dresden, TN", "Drummonds, TN", "Duck River, TN", "Duff, TN",
 "Dukedom, TN", "Dunlap, TN", "Dyer, TN", "Dyersburg, TN", "Eads, TN",
 "Eagleville, TN", "East Ridge, TN", "Eidson, TN", "Elizabethton, TN",
 "Elmwood, TN", "Elora, TN", "Englewood, TN", "Enville, TN", "Erin, TN",
 "Erwin, TN", "Estill Springs, TN", "Ethridge, TN", "Etowah, TN",
 "Eva, TN", "Evensville, TN", "Fairfield Glade, TN", "Fairview, TN",
 "Fall Branch, TN", "Farner, TN", "Fayetteville, TN", "Finger, TN",
 "Finley, TN", "Five Points, TN", "Flag Pond, TN", "Flatwoods, TN",
 "Flintville, TN", "Fort Campbell, TN", "Fort Pillow, TN",
 "Frankewing, TN", "Franklin, TN", "Friendship, TN", "Friendsville, TN",
 "Gadsden, TN", "Gainesboro, TN", "Gallatin, TN", "Gassaway, TN",
 "Gates, TN", "Gatlinburg, TN", "Georgetown, TN", "Germantown, TN",
 "Gleason, TN", "Goodlettsville, TN", "Goodspring, TN",
 "Gordonsville, TN", "Grand Junction, TN", "Grandview, TN",
 "Granville, TN", "Gray, TN", "Graysville, TN", "Greenback, TN",
 "Greenbrier, TN", "Greenfield, TN", "Grimsley, TN",
 "Gruetli Laager, TN", "Guild, TN", "Guys, TN", "Halls, TN",
 "Hampshire, TN", "Hampton, TN", "Harriman, TN", "Harrison, TN",
 "Harrogate, TN", "Hartford, TN", "Hartsville, TN", "Heiskell, TN",
 "Helenwood, TN", "Henderson, TN", "Hendersonville, TN", "Henry, TN",
 "Hermitage, TN", "Hickman, TN", "Hickory Hill, TN",
 "Hickory Valley, TN", "Hilham, TN", "Hillsboro, TN",
 "Hiwassee College, TN", "Hixson, TN", "Holladay, TN", "Hollow Rock, TN",
 "Hornbeak, TN", "Hornsby, TN", "Humboldt, TN", "Huntingdon, TN",
 "Huntland, TN", "Huntsville, TN", "Huron, TN", "Hurricane Mills, TN",
 "Indian Mound, TN", "Iron City, TN", "Jacks Creek, TN", "Jacksboro, TN",

"Jackson, TN", "Jamestown, TN", "Jefferson City, TN", "Jellico, TN",
 "Joelton, TN", "Johnson City, TN", "Jonesborough, TN", "Karns, TN",
 "Kelso, TN", "Kenton, TN", "Kimball, TN", "Kimberlin Height, TN",
 "Kimmings, TN", "Kingsport, TN", "Kingston, TN", "Kingston Springs, TN",
 "Knoxville, TN", "Kodak, TN", "Kyles Ford, TN", "La Vergne, TN",
 "Lafayette, TN", "Lake City, TN", "Lancaster, TN", "Lancing, TN",
 "Lascassas, TN", "Laurel Bloomery, TN", "Lavinia, TN",
 "Lawrenceburg, TN", "Lebanon, TN", "Lenoir City, TN", "Leoma, TN",
 "Lewisburg, TN", "Lexington, TN", "Livingston, TN", "Lobelville, TN",
 "Lookout Mountain, TN", "Loretto, TN", "Loudon, TN", "Louisville, TN",
 "Lowland, TN", "Luray, TN", "Luttrell, TN", "Lutts, TN",
 "Lynchburg, TN", "Lynn Garden, TN", "Lynnville, TN", "Madison, TN",
 "Manchester, TN", "Mansfield, TN", "Martin, TN", "Maryville, TN",
 "Mascot, TN", "Mason, TN", "Maynardville, TN", "Mc Donald, TN",
 "Mc Ewen, TN", "Mc Kenzie, TN", "Medina, TN", "Medon, TN",
 "Melrose, TN", "Memphis, TN", "Mercer, TN", "Michie, TN",
 "Middleton, TN", "Midway, TN", "Milan, TN", "Milledgeville, TN",
 "Millington, TN", "Milton, TN", "Minor Hill, TN", "Mohawk, TN",
 "Monroe, TN", "Monteagle, TN", "Monterey, TN", "Mooreburg, TN",
 "Morley, TN", "Morris Chapel, TN", "Morrison, TN", "Morristown, TN",
 "Moscow, TN", "Mosheim, TN", "Moss, TN", "Mount Carmel, TN",
 "Mount Juliet, TN", "Mount Pleasant, TN", "Mountain City, TN",
 "Mulberry, TN", "Murfreesboro, TN", "Nashville, TN",
 "New Johnsonville, TN", "New Market, TN", "New Tazewell, TN",
 "Newbern, TN", "Newcomb, TN", "Newport, TN", "Niota, TN",
 "Nolensville, TN", "Normandy, TN", "Nunnally, TN", "Oak Ridge, TN",
 "Oakdale, TN", "Oakfield, TN", "Oakland, TN", "Obion, TN", "Ocoee, TN",
 "Old Hickory, TN", "Oldfort, TN", "Olivehill, TN", "Oliver Springs, TN",
 "Oneida, TN", "Only, TN", "Ooltewah, TN", "Orlinda, TN",
 "Pall Mall, TN", "Palmer, TN", "Palmersville, TN", "Palmyra, TN",
 "Paris, TN", "Parrottsville, TN", "Parsons, TN", "Pegram, TN",
 "Pelham, TN", "Petersburg, TN", "Petros, TN", "Philadelphia, TN",
 "Pigeon Forge, TN", "Pikeville, TN", "Piney Flats, TN", "Pinson, TN",
 "Pioneer, TN", "Plaza, TN", "Pleasant Hill, TN", "Pleasant Shade, TN",
 "Pleasant View, TN", "Pleasantville, TN", "Pocahontas, TN",
 "Portland, TN", "Postelle, TN", "Powder Springs, TN", "Powell, TN",
 "Primm Springs, TN", "Prospect, TN", "Pulaski, TN", "Puryear, TN",
 "Quebeck, TN", "Ramer, TN", "Ravenscroft, TN", "Readyville, TN",
 "Reagan, TN", "Red Bank, TN", "Red Boiling Spri, TN", "Reliance, TN",
 "Riceville, TN", "Rickman, TN", "Riddleton, TN", "Ridgely, TN",
 "Ripley, TN", "Rives, TN", "Roan Mountain, TN", "Robbins, TN",
 "Rockford, TN", "Rockvale, TN", "Rockwood, TN", "Rogersville, TN",
 "Rossville, TN", "Royal, TN", "Russellville, TN", "Rutherford, TN",
 "Rutledge, TN", "Saint Joseph, TN", "Sale Creek, TN", "Saltillo, TN",
 "Santa Fe, TN", "Sardis, TN", "Saulsbury, TN", "Savannah, TN",
 "Scotts Hill, TN", "Selmer, TN", "Sequatchie, TN", "Sevierville, TN",
 "Sewanee, TN", "Seymour, TN", "Shady Valley, TN", "Sharon, TN",
 "Sharps Chapel, TN", "Sherwood, TN", "Shiloh, TN",
 "Signal Mountain, TN", "Silver Point, TN", "Smithville, TN",
 "Smyrna, TN", "Sneedville, TN", "Soddy Daisy, TN", "Somerville, TN",
 "South Fulton, TN", "South Pittsburg, TN", "Southside, TN",
 "Speedwell, TN", "Spencer, TN", "Spring City, TN", "Spring Hill, TN",
 "Springfield, TN", "Springville, TN", "Stanton, TN", "Stantonville, TN",
 "Stewart, TN", "Strawberry Plain, TN", "Sugar Tree, TN",
 "Summertown, TN", "Sunbright, TN", "Surgoinville, TN",

"Sweetwater, TN", "Taft, TN", "Talbott, TN", "Tallasse, TN",
 "Tazewell, TN", "Telford, TN", "Tellico Plains, TN", "Ten Mile, TN",
 "Tennessee Ridge, TN", "Thompsons Station, TN", "Thorn Hill, TN",
 "Tiptonville, TN", "Toone, TN", "Townsend, TN", "Tracy City, TN",
 "Trade, TN", "Treadway, TN", "Trenton, TN", "Trezevant, TN",
 "Trimble, TN", "Troy, TN", "Turtletown, TN", "Unicoi, TN",
 "Union City, TN", "Unionville, TN", "Vanleer, TN", "Vonore, TN",
 "Walland, TN", "Walling, TN", "Wartburg, TN", "Wartrace, TN",
 "Washburn, TN", "Washington College, TN", "Watauga, TN", "Watertown, TN",
 "Waverly, TN", "Waynesboro, TN", "Westmoreland, TN", "Westpoint, TN",
 "Westport, TN", "White Bluff, TN", "White House, TN",
 "Whites Creek, TN", "Whitesburg, TN", "Whiteside, TN", "Whiteville, TN",
 "Whitleyville, TN", "Whitwell, TN", "Wilder, TN", "Wildersville, TN",
 "Williamsport, TN", "Williston, TN", "Winchester, TN", "Winfield, TN",
 "Woodbury, TN", "Woodlawn, TN", "Wrigley, TN", "Yuma, TN", "Abbott, TX",
 "Abernathy, TX", "Abilene, TX", "Ackerly, TX", "Adkins, TX",
 "Adrian, TX", "Afton, TX", "Alamo, TX", "Alamo Heights, TX", "Alba, TX",
 "Albany, TX", "Albert, TX", "Aledo, TX", "Alice, TX", "Allen, TX",
 "Alleyton, TX", "Alpine, TX", "Alta Loma, TX", "Alton, TX",
 "Alvarado, TX", "Alvin, TX", "Alvord, TX", "Amarillo, TX", "Ames, TX",
 "Amherst, TX", "Anderson, TX", "Andice, TX", "Andrews, TX",
 "Angleton, TX", "Anna, TX", "Annona, TX", "Anson, TX", "Anthony, TX",
 "Anton, TX", "Apple Springs, TX", "Appleby, TX", "Aquila, TX",
 "Aransas Pass, TX", "Arcadia, TX", "Argyle, TX", "Arlington, TX",
 "Armstrong, TX", "Arp, TX", "Art, TX", "Arthur City, TX",
 "Asherton, TX", "Aspermont, TX", "Atascosa, TX", "Athens, TX",
 "Atlanta, TX", "Aubrey, TX", "Austin, TX", "Austonio, TX", "Avery, TX",
 "Avinger, TX", "Avoca, TX", "Axtell, TX", "Azle, TX", "Bacliff, TX",
 "Bagwell, TX", "Baird, TX", "Balch Springs, TX", "Balcones Heights, TX",
 "Ballinger, TX", "Balmorhea, TX", "Bandera, TX", "Bangs, TX",
 "Barksdale, TX", "Barnhart, TX", "Barnum, TX", "Barrett, TX",
 "Barry, TX", "Barstow, TX", "Bartlett, TX", "Bastrop, TX",
 "Batesville, TX", "Batson, TX", "Bayside, TX", "Baytown, TX",
 "Bayview, TX", "Beasley, TX", "Beaumont, TX", "Bebe, TX",
 "Beckville, TX", "Bedford, TX", "Bee House, TX", "Beeville, TX",
 "Bellaire, TX", "Bellevue, TX", "Bellmead, TX", "Bells, TX",
 "Bellville, TX", "Belton, TX", "Ben Franklin, TX", "Ben Wheeler, TX",
 "Benbrook, TX", "Bend, TX", "Bergheim, TX", "Bertram, TX", "Best, TX",
 "Beverly Hills, TX", "Big Bend National, TX", "Big Sandy, TX",
 "Big Wells, TX", "Bigfoot, TX", "Bishop, TX", "Bivins, TX", "Black, TX",
 "Blackwell, TX", "Blanco, TX", "Blanket, TX", "Bleiblerville, TX",
 "Blessing, TX", "Bloomburg, TX", "Blooming Grove, TX",
 "Bloomington, TX", "Blossom, TX", "Blue Ridge, TX", "Bluff Dale, TX",
 "Bluffton, TX", "Blum, TX", "Bogata, TX", "Boling, TX", "Bon Ami, TX",
 "Bon Wier, TX", "Bonanza, TX", "Bonham, TX", "Booker, TX", "Boston, TX",
 "Bovina, TX", "Bowie, TX", "Boyd, TX", "Brackettville, TX",
 "Brashear, TX", "Brazoria, TX", "Breckenridge, TX", "Bremond, TX",
 "Brenham, TX", "Bridge City, TX", "Bridgeport, TX", "Briggs, TX",
 "Briscoe, TX", "Broadus, TX", "Bronson, TX", "Bronte, TX",
 "Brookeland, TX", "Brookesmith, TX", "Brooks A F B, TX",
 "Brookshire, TX", "Brookston, TX", "Brownfield, TX", "Brownsville, TX",
 "Bruceville, TX", "Bruni, TX", "Bryan, TX", "Bryson, TX",
 "Buchanan Dam, TX", "Buckholts, TX", "Buda, TX", "Bula, TX", "Buna, TX",
 "Burkburnett, TX", "Burkett, TX", "Burkeville, TX", "Burleson, TX",
 "Burlington, TX", "Burnet, TX", "Burton, TX", "Byers, TX", "Bynum, TX",

"Caddo, TX", "Caddo Mills, TX", "Caldwell, TX", "Call, TX",
"Calliham, TX", "Calvert, TX", "Cameron, TX", "Camp Verde, TX",
"Camp Wood, TX", "Campbell, TX", "Campbellton, TX", "Canton, TX",
"Canutillo, TX", "Canyon, TX", "Canyon Lake, TX", "Carbon, TX",
"Carlsbad, TX", "Carlton, TX", "Carmine, TX", "Carrizo Springs, TX",
"Carrollton, TX", "Carswell Afb, TX", "Carthage, TX", "Castell, TX",
"Castle Hills, TX", "Castroville, TX", "Cat Spring, TX",
"Cedar Creek, TX", "Cedar Hill, TX", "Cedar Park, TX", "Celeste, TX",
"Celina, TX", "Center, TX", "Centerville, TX", "Chalk, TX",
"Chandler, TX", "Channelview, TX", "Channing, TX", "Chappell Hill, TX",
"Charlotte, TX", "Chatfield, TX", "Cherokee, TX", "Chester, TX",
"Chico, TX", "Chillicothe, TX", "Chilton, TX", "China Spring, TX",
"Chireno, TX", "Christoval, TX", "Cibolo, TX", "Cisco, TX",
"Clarendon, TX", "Clarksville, TX", "Clarksville City, TX",
"Claude, TX", "Clear Lake Shore, TX", "Cleburne, TX", "Cleveland, TX",
"Clint, TX", "Clodine, TX", "Clute, TX", "Clyde, TX", "Coahoma, TX",
"Cockrell Hill, TX", "Coldspring, TX", "Coleman, TX",
"College Station, TX", "Colleyville, TX", "Collinsville, TX",
"Colorado City, TX", "Columbus, TX", "Comanche, TX", "Comfort, TX",
"Commerce, TX", "Como, TX", "Comstock, TX", "Concan, TX",
"Concepcion, TX", "Concord, TX", "Cone, TX", "Conroe, TX",
"Converse, TX", "Cookville, TX", "Coolidge, TX", "Cooper, TX",
"Coppell, TX", "Corpus Christi, TX", "Corsicana, TX", "Cost, TX",
"Cotulla, TX", "Coupland, TX", "Covington, TX", "Coyanosa, TX",
"Crandall, TX", "Crane, TX", "Cranfills Gap, TX", "Crawford, TX",
"Creedmoor, TX", "Cresson, TX", "Crosbyton, TX", "Cross Plains, TX",
"Crowell, TX", "Crowley, TX", "Crystal Beach, TX", "Crystal City, TX",
"Cuero, TX", "Cumby, TX", "Cushing, TX", "Cut And Shoot, TX",
"Cypress, TX", "Cypress Mill, TX", "D Hanis, TX", "Daingerfield, TX",
"Dale, TX", "Dalhart, TX", "Dallas, TX", "Damon, TX", "Danbury, TX",
"Danevang, TX", "Davilla, TX", "Dawson, TX", "Dayton, TX",
"De Berry, TX", "De Kalb, TX", "De Leon, TX", "De Soto, TX",
"Decatur, TX", "Deer Park, TX", "Del Valle, TX", "Dell City, TX",
"Delmita, TX", "Denison, TX", "Denton, TX", "Denver City, TX",
"Deport, TX", "Dermott, TX", "Desdemona, TX", "Detroit, TX",
"Devers, TX", "Devine, TX", "Deweyville, TX", "Dialville, TX",
"Diboll, TX", "Dickens, TX", "Dike, TX", "Dilley, TX", "Dime Box, TX",
"Dimmitt, TX", "Dodd City, TX", "Dodson, TX", "Dogwood, TX",
"Donie, TX", "Donna, TX", "Doole, TX", "Doss, TX", "Douglass, TX",
"Douglassville, TX", "Driftwood, TX", "Dripping Springs, TX",
"Dryden, TX", "Dublin, TX", "Dumas, TX", "Dumont, TX",
"Duncanville, TX", "Dunlay, TX", "Dyess Afb, TX", "Eagle Lake, TX",
"Eagle Pass, TX", "Early, TX", "Earth, TX", "East Bernard, TX",
"East Texas Cente, TX", "Eastland, TX", "Ecletto, TX", "Ector, TX",
"Eddy, TX", "Eden, TX", "Edgewood, TX", "Edinburg, TX", "Edna, TX",
"Edom, TX", "El Campo, TX", "El Lago, TX", "El Paso, TX", "Elbert, TX",
"Eldorado, TX", "Electra, TX", "Elgin, TX", "Ellinger, TX",
"Elm Mott, TX", "Elmaton, TX", "Elmendorf, TX", "Emory, TX",
"Encinal, TX", "Encino, TX", "Ennis, TX", "Enochs, TX", "Eola, TX",
"Era, TX", "Eules, TX", "Eustace, TX", "Everman, TX", "Fairfield, TX",
"Falfurrias, TX", "Falls City, TX", "Farmers Branch, TX",
"Farmersville, TX", "Farwell, TX", "Fayetteville, TX", "Ferris, TX",
"Fieldton, TX", "Fife, TX", "Fischer, TX", "Flat, TX", "Flatonia, TX",
"Flint, TX", "Flomot, TX", "Florence, TX", "Floresville, TX",
"Flower Mound, TX", "Floydada, TX", "Fluvanna, TX", "Follett, TX",

"Forest, TX", "Forestburg, TX", "Forney, TX", "Forreston, TX",
 "Fort Bliss, TX", "Fort Davis, TX", "Fort Hancock, TX", "Fort Hood, TX",
 "Fort Mc Kavett, TX", "Fort Sam Houston, TX", "Fort Stockton, TX",
 "Fort Worth, TX", "Fowlerton, TX", "Franklin, TX", "Frankston, TX",
 "Fred, TX", "Fredericksburg, TX", "Fredonia, TX", "Freer, TX",
 "Freestone, TX", "Fresno, TX", "Friendswood, TX", "Frisco, TX",
 "Fritch, TX", "Frost, TX", "Fruitvale, TX", "Fulshear, TX",
 "Fulton, TX", "Gail, TX", "Galena Park, TX", "Galveston, TX",
 "Ganado, TX", "Garciasville, TX", "Garden City, TX", "Garden Ridge, TX",
 "Gardendale, TX", "Garland, TX", "Garrison, TX", "Garwood, TX",
 "Gary, TX", "George West, TX", "Georgetown, TX", "Giddings, TX",
 "Gillett, TX", "Gilmer, TX", "Girard, TX", "Gladewater, TX",
 "Glazier, TX", "Glen Rose, TX", "Godley, TX", "Goldsboro, TX",
 "Goldsmith, TX", "Goldthwaite, TX", "Goliad, TX", "Gonzales, TX",
 "Goodrich, TX", "Gordon, TX", "Gordonville, TX", "Goree, TX",
 "Gorman, TX", "Gouldbusk, TX", "Graford, TX", "Graham, TX",
 "Granbury, TX", "Grand Prairie, TX", "Grand Saline, TX",
 "Grandfalls, TX", "Grandview, TX", "Granger, TX", "Grangerland, TX",
 "Grapeland, TX", "Grapevine, TX", "Greenville, TX", "Grey Forest, TX",
 "Groesbeck, TX", "Groom, TX", "Groves, TX", "Groveton, TX",
 "Grulla, TX", "Gruver, TX", "Guerra, TX", "Gun Barrel City, TX",
 "Gunter, TX", "Gustine, TX", "Guy, TX", "Hale Center, TX",
 "Hallettsville, TX", "Hallsville, TX", "Haltom City, TX",
 "Hamilton, TX", "Hamlin, TX", "Hamshire, TX", "Hankamer, TX",
 "Happy, TX", "Hargill, TX", "Harker Heights, TX", "Harleton, TX",
 "Harlingen, TX", "Harper, TX", "Harrold, TX", "Hart, TX", "Hartley, TX",
 "Harwood, TX", "Haskell, TX", "Haslet, TX", "Hawkins, TX", "Hawley, TX",
 "Hearne, TX", "Heath, TX", "Hebbronville, TX", "Hedley, TX",
 "Hemphill, TX", "Hempstead, TX", "Henderson, TX", "Henrietta, TX",
 "Hereford, TX", "Hermleigh, TX", "Hewitt, TX", "Hext, TX", "Hico, TX",
 "Hidalgo, TX", "Higgins, TX", "Highland Village, TX", "Highlands, TX",
 "Hillister, TX", "Hillsboro, TX", "Hilltop Lakes, TX", "Hitchcock, TX",
 "Hobson, TX", "Hockley, TX", "Holland, TX", "Holliday, TX",
 "Hollywood Park, TX", "Honey Grove, TX", "Horizon City, TX",
 "Houston, TX", "Howe, TX", "Hubbard, TX", "Huffman, TX",
 "Hughes Springs, TX", "Hull, TX", "Humble, TX", "Hunt, TX",
 "Huntington, TX", "Huntsville, TX", "Hurst, TX", "Hutchins, TX",
 "Hutto, TX", "Hye, TX", "Idalou, TX", "Imperial, TX", "Industry, TX",
 "Inez, TX", "Ingleside, TX", "Ingram, TX", "Iola, TX", "Iowa Park, TX",
 "Ira, TX", "Iraan, TX", "Iredell, TX", "Irving, TX", "Italy, TX",
 "Itasca, TX", "Ivanhoe, TX", "Izoro, TX", "Jacinto City, TX",
 "Jacksboro, TX", "Jacksonville, TX", "Jarrell, TX", "Jayton, TX",
 "Jermyn, TX", "Jersey Village, TX", "Jewett, TX", "Joaquin, TX",
 "Johnson City, TX", "Jonesboro, TX", "Jonestown, TX", "Joshua, TX",
 "Jourdanton, TX", "Junction, TX", "Justin, TX", "Karnack, TX",
 "Karnes City, TX", "Kaufman, TX", "Keene, TX", "Keller, TX",
 "Kellerville, TX", "Kelly A F B, TX", "Keltys, TX", "Kempner, TX",
 "Kendalia, TX", "Kenedy, TX", "Kennard, TX", "Kennedale, TX",
 "Kent, TX", "Kerens, TX", "Kermit, TX", "Kerrville, TX", "Kilgore, TX",
 "Killeen, TX", "Kingsbury, TX", "Kingsland, TX", "Kingsville Naval, TX",
 "Kirby, TX", "Kirkland, TX", "Klein, TX", "Klondike, TX", "Knippa, TX",
 "Knott, TX", "Knox City, TX", "Kopperl, TX", "Kosse, TX", "Kountze, TX",
 "Kress, TX", "Krum, TX", "La Coste, TX", "La Feria, TX",
 "La Grange, TX", "La Marque, TX", "La Pryor, TX", "La Vernia, TX",
 "Ladonia, TX", "Laguna Park, TX", "Lake Creek, TX", "Lake Dallas, TX",

"Lake Jackson, TX", "Lake Kiowa, TX", "Lakehills, TX", "Lakeview, TX",
 "Lakeway, TX", "Lakewood Village, TX", "Lamesa, TX", "Lampasas, TX",
 "Lancaster, TX", "Laneville, TX", "Laredo, TX", "Larue, TX",
 "Laughlin A F B, TX", "Lawn, TX", "Leaday, TX", "League City, TX",
 "Leakey, TX", "Leander, TX", "Leary, TX", "Ledbetter, TX",
 "Leesburg, TX", "Leesville, TX", "Lenorah, TX", "Leon Valley, TX",
 "Leona, TX", "Leonard, TX", "Levelland, TX", "Lewisville, TX",
 "Lexington, TX", "Liberty Hill, TX", "Lincoln, TX", "Linden, TX",
 "Lindsay, TX", "Linn, TX", "Lipan, TX", "Lipscomb, TX",
 "Littlefield, TX", "Live Oak, TX", "Liverpool, TX", "Lockhart, TX",
 "Lockney, TX", "Lohn, TX", "Lolita, TX", "Lometa, TX", "London, TX",
 "Lone Oak, TX", "Lone Star, TX", "Long Branch, TX", "Longview, TX",
 "Loop, TX", "Lorraine, TX", "Lorena, TX", "Lorenzo, TX", "Lott, TX",
 "Louise, TX", "Lovelady, TX", "Loving, TX", "Lubbock, TX",
 "Lueders, TX", "Luling, TX", "Lyford, TX", "Lytle, TX",
 "Madisonville, TX", "Magnolia, TX", "Magnolia Springs, TX",
 "Malakoff, TX", "Malone, TX", "Manchaca, TX", "Manor, TX",
 "Mansfield, TX", "Manvel, TX", "Maple, TX", "Marathon, TX", "Marfa, TX",
 "Marietta, TX", "Marion, TX", "Markham, TX", "Marlin, TX",
 "Marquez, TX", "Marshall, TX", "Mart, TX", "Martindale, TX",
 "Maryneal, TX", "Mason, TX", "Matador, TX", "Matagorda, TX",
 "Mathis, TX", "Maud, TX", "Maxwell, TX", "May, TX", "Maypearl, TX",
 "Mc Camey, TX", "Mc Caulley, TX", "Mc Coy, TX", "Mc Dade, TX",
 "Mc Gregor, TX", "Mc Kinney, TX", "Mc Queeney, TX", "Mcadoo, TX",
 "Mcallen, TX", "Meadow, TX", "Medina, TX", "Melissa, TX", "Melvin, TX",
 "Memphis, TX", "Menard, TX", "Mentone, TX", "Mercedes, TX",
 "Mereta, TX", "Meridian, TX", "Merkel, TX", "Mertens, TX",
 "Mertzson, TX", "Mesquite, TX", "Mexia, TX", "Meyersville, TX",
 "Miami, TX", "Mico, TX", "Midfield, TX", "Midkiff, TX", "Midland, TX",
 "Midlothian, TX", "Midway, TX", "Milam, TX", "Milano, TX", "Miles, TX",
 "Milford, TX", "Millersview, TX", "Millsap, TX", "Mineola, TX",
 "Mineral Wells, TX", "Mingus, TX", "Mirando City, TX",
 "Missouri City, TX", "Mobeetie, TX", "Monahans, TX", "Monroe City, TX",
 "Montague, TX", "Montalba, TX", "Monte Alto, TX", "Montgomery, TX",
 "Moody, TX", "Moore, TX", "Moran, TX", "Morgan, TX", "Morse, TX",
 "Morton, TX", "Moscow, TX", "Moulton, TX", "Mount Calm, TX",
 "Mount Enterprise, TX", "Mount Pleasant, TX", "Mount Selman, TX",
 "Mount Vernon, TX", "Mountain Home, TX", "Mt Sylvan, TX",
 "Muenster, TX", "Muldoon, TX", "Muleshoe, TX", "Mullin, TX",
 "Munday, TX", "Murchison, TX", "Murphy, TX", "Naples, TX", "Nash, TX",
 "Natalia, TX", "Navasota, TX", "Nazareth, TX", "Nederland, TX",
 "Needville, TX", "Nemo, TX", "Nevada, TX", "New Caney, TX",
 "New Diana, TX", "New Ulm, TX", "New Waverly, TX", "Newark, TX",
 "Newcastle, TX", "Newton, TX", "Nixon, TX", "Nocona, TX", "Nolan, TX",
 "Nolanville, TX", "Nordheim, TX", "North Richland H, TX",
 "North Zulch, TX", "Norton, TX", "Novice, TX", "O Brien, TX",
 "Oakhurst, TX", "Oakville, TX", "Oakwood, TX", "Odem, TX", "Odessa, TX",
 "Odonnell, TX", "Oglesby, TX", "Oklaunion, TX", "Old Glory, TX",
 "Olmos Park, TX", "Olney, TX", "Olton, TX", "Omaha, TX",
 "Orange Grove, TX", "Ore City, TX", "Otto, TX", "Ovalo, TX",
 "Overton, TX", "Ovilla, TX", "Ozona, TX", "Paige, TX", "Paint Rock, TX",
 "Palacios, TX", "Palestine, TX", "Palmer, TX", "Palo Pinto, TX",
 "Pampa, TX", "Panhandle, TX", "Panorama Village, TX", "Paradise, TX",
 "Paris, TX", "Park Row, TX", "Pasadena, TX", "Pattonville, TX",
 "Pear Valley, TX", "Pearland, TX", "Pearsall, TX", "Pecan Gap, TX",

"Penelope, TX", "Pennington, TX", "Pep, TX", "Perrin, TX",
 "Perryton, TX", "Petersburg, TX", "Petroliia, TX", "Petty, TX",
 "Pflugerville, TX", "Pharr, TX", "Phillips, TX", "Pickton, TX",
 "Pilot Point, TX", "Pinehurst, TX", "Pineland, TX", "Pittsburg, TX",
 "Plains, TX", "Plainview, TX", "Plano, TX", "Plantersville, TX",
 "Pleasanton, TX", "Pledger, TX", "Point, TX", "Pointblank, TX",
 "Pollok, TX", "Ponder, TX", "Pontotoc, TX", "Poolville, TX",
 "Port Acres, TX", "Port Arthur, TX", "Port Isabel, TX",
 "Port Lavaca, TX", "Port Mansfield, TX", "Port Neches, TX",
 "Port O Connor, TX", "Porter, TX", "Portland, TX", "Post, TX",
 "Poteet, TX", "Poth, TX", "Pottsboro, TX", "Pottsville, TX",
 "Powderly, TX", "Powell, TX", "Prairie Hill, TX", "Premont, TX",
 "Presidio, TX", "Price, TX", "Priddy, TX", "Princeton, TX",
 "Prosper, TX", "Purdon, TX", "Purmela, TX", "Pyote, TX", "Quail, TX",
 "Quanah, TX", "Queen City, TX", "Quinlan, TX", "Quintana, TX",
 "Quitague, TX", "Quitman, TX", "Rainbow, TX", "Randolph A F B, TX",
 "Ranger, TX", "Ransom Canyon, TX", "Ravenna, TX", "Raymondville, TX",
 "Reagan, TX", "Realitos, TX", "Red Rock, TX", "Reese Air Force, TX",
 "Refugio, TX", "Reklaw, TX", "Rhome, TX", "Rice, TX", "Richards, TX",
 "Richardson, TX", "Richland, TX", "Richland Springs, TX", "Riesel, TX",
 "Ringgold, TX", "Rio Bravo, TX", "Rio Frio, TX", "Rio Grande City, TX",
 "Rio Hondo, TX", "Rio Vista, TX", "Riomedina, TX", "Rising Star, TX",
 "River Oaks, TX", "Riviera, TX", "Roaring Springs, TX",
 "Robert Lee, TX", "Robstown, TX", "Roby, TX", "Rochelle, TX",
 "Rochester, TX", "Rockdale, TX", "Rockland, TX", "Rockport, TX",
 "Rocksprings, TX", "Rockwood, TX", "Rogers, TX", "Roma, TX",
 "Roosevelt, TX", "Ropesville, TX", "Rosanky, TX", "Roscoe, TX",
 "Rosebud, TX", "Rosenberg, TX", "Rosharon, TX", "Rosston, TX",
 "Rotan, TX", "Round Mountain, TX", "Round Rock, TX", "Round Top, TX",
 "Rowena, TX", "Rowlett, TX", "Roxton, TX", "Royse City, TX", "Rule, TX",
 "Runge, TX", "Sabinal, TX", "Sachse, TX", "Sadler, TX", "Sagerton, TX",
 "Saginaw, TX", "Saint Hedwig, TX", "Saint Jo, TX", "Salado, TX",
 "Salt Flat, TX", "Saltillo, TX", "Sam Rayburn, TX", "San Angelo, TX",
 "San Antonio, TX", "San Augustine, TX", "San Benito, TX",
 "San Diego, TX", "San Isidro, TX", "San Juan, TX", "San Leon, TX",
 "San Marcos, TX", "San Perlita, TX", "San Saba, TX", "San Ygnacio, TX",
 "Sandia, TX", "Sandy, TX", "Sanger, TX", "Santa Anna, TX",
 "Santa Elena, TX", "Santa Rosa, TX", "Santo, TX", "Saratoga, TX",
 "Sargent, TX", "Sarita, TX", "Savoy, TX", "Schulenburg, TX",
 "Scotland, TX", "Scroggins, TX", "Scurry, TX", "Seadrift, TX",
 "Seagoville, TX", "Seagraves, TX", "Sealy, TX", "Sebastian, TX",
 "Segno, TX", "Seguin, TX", "Selma, TX", "Seminole, TX",
 "Seven Points, TX", "Seymour, TX", "Shallowater, TX",
 "Shavano Park, TX", "Sheffield, TX", "Shelbyville, TX", "Shepherd, TX",
 "Sheppard Afb, TX", "Sherman, TX", "Shiner, TX", "Shoreacres, TX",
 "Sidney, TX", "Sierra Blanca, TX", "Silsbee, TX", "Silver, TX",
 "Silverton, TX", "Simms, TX", "Singleton, TX", "Sinton, TX",
 "Sisterdale, TX", "Skellytown, TX", "Skidmore, TX", "Slocum, TX",
 "Smiley, TX", "Smithland, TX", "Smithville, TX", "Somerset, TX",
 "Somerville, TX", "Sonora, TX", "Sour Lake, TX", "South Houston, TX",
 "South Padre Isla, TX", "Spearman, TX", "Spicewood, TX",
 "Splendora, TX", "Spofford, TX", "Spring, TX", "Spring Branch, TX",
 "Springlake, TX", "Springtown, TX", "Spur, TX", "Spurger, TX",
 "Stafford, TX", "Stamford, TX", "Stanton, TX", "Star, TX",
 "Stephenville, TX", "Sterling City, TX", "Stinnett, TX",

"Stockdale, TX", "Stratford, TX", "Strawn, TX", "Streetman, TX",
 "Sudan, TX", "Sugar Land, TX", "Sullivan City, TX", "Sulphur Bluff, TX",
 "Sulphur Springs, TX", "Summerfield, TX", "Sumner, TX", "Sunray, TX",
 "Sunrise Beach, TX", "Sunset, TX", "Sutherland Sprin, TX", "Sweeny, TX",
 "Sweetwater, TX", "Sylvester, TX", "Taft, TX", "Tahoka, TX",
 "Talco, TX", "Talpa, TX", "Tarpley, TX", "Tarzan, TX", "Tatum, TX",
 "Taylor, TX", "Teague, TX", "Telegraph, TX", "Telephone, TX",
 "Tell, TX", "Temple, TX", "Tenaha, TX", "Tennessee Colony, TX",
 "Terlingua, TX", "Terrell, TX", "Texarkana, TX", "Texas City, TX",
 "Texline, TX", "The Colony, TX", "The Woodlands, TX", "Thorndale, TX",
 "Thornton, TX", "Thrall, TX", "Three Rivers, TX", "Throckmorton, TX",
 "Tilden, TX", "Timpson, TX", "Tioga, TX", "Tivoli, TX", "Tokio, TX",
 "Tolar, TX", "Tomball, TX", "Tow, TX", "Trent, TX", "Trenton, TX",
 "Trinidad, TX", "Trinity, TX", "Trophy Club, TX", "Troup, TX",
 "Troy, TX", "Truscott, TX", "Turkey, TX", "Turnersville, TX",
 "Turnertown, TX", "Tuscola, TX", "Twitty, TX", "Tye, TX", "Tyler, TX",
 "Tynan, TX", "Uhland, TX", "Utopia, TX", "Uvalde, TX",
 "V A Hospital, TX", "Valentine, TX", "Valera, TX", "Valley Mills, TX",
 "Valley Spring, TX", "Valley View, TX", "Van, TX", "Van Alstyne, TX",
 "Van Vleck, TX", "Vancourt, TX", "Vanderpool, TX", "Vealmoor, TX",
 "Vega, TX", "Venus, TX", "Vera, TX", "Verhalen, TX", "Vernon, TX",
 "Victoria, TX", "Vidor, TX", "Vigo Park, TX", "Village, TX", "Voca, TX",
 "Von Ormy, TX", "Waco, TX", "Wadsworth, TX", "Waelder, TX",
 "Wake Village, TX", "Wall, TX", "Waller, TX", "Wallis, TX",
 "Wallisville, TX", "Walnut Springs, TX", "Warren, TX", "Washington, TX",
 "Waskom, TX", "Watauga, TX", "Waxahachie, TX", "Wayside, TX",
 "Weatherford, TX", "Webster, TX", "Weimar, TX", "Weinert, TX",
 "Welch, TX", "Wellington, TX", "Wells, TX", "Weslaco, TX", "West, TX",
 "West Columbia, TX", "West Lake Hills, TX", "West Orange, TX",
 "West Point, TX", "Westbrook, TX", "Westhoff, TX", "Wetmore, TX",
 "Wharton, TX", "Wheeler, TX", "White Deer, TX", "White Settlement, TX",
 "Whiteface, TX", "Whitehouse, TX", "Whitesboro, TX", "Whitewright, TX",
 "Whitsett, TX", "Whitt, TX", "Wichita Falls, TX", "Wiergate, TX",
 "Wildorado, TX", "Wilford Hall U S, TX", "Willis, TX",
 "Willow City, TX", "Wills Point, TX", "Wilmer, TX", "Wilson, TX",
 "Wimberley, TX", "Windcrest, TX", "Windom, TX", "Windthorst, TX",
 "Winfield, TX", "Wingate, TX", "Wink, TX", "Winnie, TX",
 "Winnsboro, TX", "Winona, TX", "Winters, TX", "Wolfe City, TX",
 "Wolfforth, TX", "Woodsboro, TX", "Woodson, TX", "Woodway, TX",
 "Wortham, TX", "Wrightsboro, TX", "Wylie, TX", "Yancey, TX",
 "Yantis, TX", "Yoakum, TX", "Yorktown, TX", "Zapata, TX", "Zavalla, TX",
 "Zephyr, TX", "Alpine, UT", "Alta, UT", "Altamont, UT", "Alton, UT",
 "Altonah, UT", "American Fork, UT", "Aneth, UT", "Antimony, UT",
 "Austin, UT", "Axtell, UT", "Beaver, UT", "Beryl, UT", "Big Water, UT",
 "Bingham Canyon, UT", "Blanding, UT", "Bluebell, UT", "Boulder, UT",
 "Bountiful, UT", "Brian Head, UT", "Bridgeland, UT", "Brigham City, UT",
 "Bryce Canyon, UT", "Bullfrog, UT", "Cedar Valley, UT",
 "Centerfield, UT", "Centerville, UT", "Central, UT", "Clarkston, UT",
 "Clearfield, UT", "Coalville, UT", "Collinston, UT", "Corinne, UT",
 "Cornish, UT", "Cottonwood, UT", "Croydon, UT", "Dammeron Valley, UT",
 "Delta, UT", "Deweyville, UT", "Draper, UT", "Duchesne, UT",
 "Dugway, UT", "Dutch John, UT", "East Carbon, UT", "Eden, UT",
 "Ephraim, UT", "Escalante, UT", "Eureka, UT", "Fairview, UT",
 "Farmington, UT", "Fayette, UT", "Ferron, UT", "Fielding, UT",
 "Fillmore, UT", "Fort Duchesne, UT", "Fremont, UT", "Garden City, UT",

"Garland, UT", "Garrison, UT", "Genola, UT", "Glendale, UT",
 "Grantsville, UT", "Green River, UT", "Greenville, UT",
 "Grouse Creek, UT", "Gunnison, UT", "Hanksville, UT", "Hanna, UT",
 "Heber City, UT", "Helper, UT", "Hill Air Force B, UT", "Hinckley, UT",
 "Holladay, UT", "Honeyville, UT", "Hooper, UT", "Huntington, UT",
 "Huntsville, UT", "Hurricane, UT", "Hyrum, UT", "Jensen, UT",
 "Joseph, UT", "Kamas, UT", "Kaysville, UT", "Kearns, UT",
 "Kingston, UT", "Laketown, UT", "Lapoint, UT", "Lark, UT", "Layton, UT",
 "Lehi, UT", "Lewiston, UT", "Lindon, UT", "Logan, UT", "Magna, UT",
 "Manila, UT", "Manti, UT", "Mantua, UT", "Mapleton, UT",
 "Marysvale, UT", "Mendon, UT", "Mexican Hat, UT", "Midvale, UT",
 "Midway, UT", "Milford, UT", "Moab, UT", "Modena, UT", "Mona, UT",
 "Monticello, UT", "Monument Valley, UT", "Morgan, UT",
 "Mount Carmel, UT", "Mount Pleasant, UT", "Mountain Home, UT",
 "Murray, UT", "Myton, UT", "Neola, UT", "Nephi, UT", "Newcastle, UT",
 "North Salt Lake, UT", "Oasis, UT", "Ogden, UT", "Orderville, UT",
 "Orem, UT", "Panguitch, UT", "Paradise, UT", "Paragonah, UT",
 "Park City, UT", "Park Valley, UT", "Parowan, UT", "Payson, UT",
 "Peoa, UT", "Pine Valley, UT", "Pintura, UT", "Pleasant Grove, UT",
 "Price, UT", "Providence, UT", "Provo, UT", "Randlett, UT",
 "Randolph, UT", "Richmond, UT", "Roosevelt, UT", "Roy, UT",
 "Rush Valley, UT", "Salina, UT", "Salt Lake City, UT", "Sandy, UT",
 "Sevier, UT", "Smithfield, UT", "Snowville, UT", "South Salt Lake, UT",
 "Spanish Fork, UT", "Springville, UT", "St George, UT", "Stockton, UT",
 "Summit, UT", "Syracuse, UT", "Tabiona, UT", "Talmage, UT",
 "Teasdale, UT", "Thompson, UT", "Tooele, UT", "Torrey, UT",
 "Tremonton, UT", "Trenton, UT", "Tridell, UT", "Trout Creek, UT",
 "Venice, UT", "Vernal, UT", "Vernon, UT", "Veyo, UT", "Vineyard, UT",
 "Wallsburg, UT", "Washington, UT", "Wellington, UT", "Wellsville, UT",
 "West Jordan, UT", "West Valley City, UT", "Whiterocks, UT",
 "Willard, UT", "Woodland Hills, UT", "Woodruff, UT", "Woods Cross, UT",
 "Abingdon, VA", "Accomac, VA", "Advance Mills, VA", "Afton, VA",
 "Airlie, VA", "Alberene, VA", "Alberta, VA", "Aldie, VA",
 "Alexandria, VA", "Alleghany, VA", "Allisonia, VA", "Altavista, VA",
 "Alton, VA", "Alum Ridge, VA", "Amelia Court Hou, VA", "Amherst, VA",
 "Amissville, VA", "Ammon, VA", "Ampthill, VA", "Annandale, VA",
 "Appomattox, VA", "Ararat, VA", "Arcola, VA", "Arlington, VA",
 "Aroda, VA", "Arvonja, VA", "Ashburn, VA", "Ashland, VA",
 "Assawoman, VA", "Atkins, VA", "Austinville, VA", "Axton, VA",
 "Aylett, VA", "Aylor, VA", "Baileys Crossroa, VA", "Bandy, VA",
 "Barhamsville, VA", "Barren Springs, VA", "Baskerville, VA",
 "Bassett, VA", "Bastian, VA", "Basye, VA", "Bavon, VA", "Beaverdam, VA",
 "Beaverlett, VA", "Bedford, VA", "Bee, VA", "Belle Haven, VA",
 "Belle View, VA", "Bellevue, VA", "Bent Mountain, VA", "Bergton, VA",
 "Berryville, VA", "Big Island, VA", "Big Stone Gap, VA",
 "Birchleaf, VA", "Birdsnest, VA", "Blackridge, VA", "Blackstone, VA",
 "Blackwater, VA", "Blackwater Bridg, VA", "Blairs, VA", "Bland, VA",
 "Bloxom, VA", "Blue Grass, VA", "Blue Ridge, VA", "Bluefield, VA",
 "Bluemont, VA", "Bohannon, VA", "Bolar, VA", "Bon Air, VA",
 "Boones Mill, VA", "Boonesville, VA", "Boston, VA", "Bowers Hill, VA",
 "Bowling Green, VA", "Boyce, VA", "Boyd Tavern, VA", "Boydton, VA",
 "Boykins, VA", "Bracey, VA", "Branchville, VA", "Brandy Station, VA",
 "Bremo Bluff, VA", "Bridgewater, VA", "Brightwood, VA", "Bristol, VA",
 "Bristow, VA", "Broad Run, VA", "Broadford, VA", "Broadway, VA",
 "Brodnax, VA", "Brookneal, VA", "Browntown, VA", "Bruington, VA",

"Buckingham, VA", "Buena Vista, VA", "Buffalo Junction, VA",
 "Bumpass, VA", "Burgess, VA", "Burke, VA", "Burkeville, VA",
 "Burnleys, VA", "Burnsville, VA", "Burr Hill, VA", "Bybee, VA",
 "Callands, VA", "Callao, VA", "Callaway, VA", "Cana, VA",
 "Cape Charles, VA", "Capron, VA", "Caret, VA", "Carrollton, VA",
 "Carrsville, VA", "Carson, VA", "Cascade, VA", "Cashes Corner, VA",
 "Castleton, VA", "Castlewood, VA", "Catawba, VA", "Catharpin, VA",
 "Catlett, VA", "Cauthornville, VA", "Cave Spring, VA",
 "Cedar Bluff, VA", "Center Cross, VA", "Centreville, VA", "Ceres, VA",
 "Champlain, VA", "Chance, VA", "Chantilly, VA", "Charles City, VA",
 "Charlotte Court, VA", "Charlottesville, VA", "Chase City, VA",
 "Chatham, VA", "Chesapeake, VA", "Chester, VA", "Chesterfield, VA",
 "Chilhowie, VA", "Chincoteague, VA", "Christiansburg, VA",
 "Church Road, VA", "Church View, VA", "Churchville, VA",
 "Clarksville, VA", "Claudville, VA", "Clear Brook, VA", "Cleveland, VA",
 "Clifton, VA", "Clifton Forge, VA", "Clinchco, VA", "Clinchport, VA",
 "Clintwood, VA", "Clover, VA", "Cloverdale, VA", "Cobham, VA",
 "Coeburn, VA", "Coleman Falls, VA", "Collinsville, VA", "Cologne, VA",
 "Colonial Heights, VA", "Columbia, VA", "Community, VA", "Conaway, VA",
 "Concord, VA", "Conicville, VA", "Copper Hill, VA", "Council, VA",
 "Courtland, VA", "Covesville, VA", "Craigsville, VA", "Crewe, VA",
 "Criders, VA", "Crimora, VA", "Cripple Creek, VA", "Critz, VA",
 "Crockett, VA", "Crozier, VA", "Crystal Hill, VA", "Cullen, VA",
 "Cumberland, VA", "Dabneys, VA", "Dahlgren, VA", "Dale City, VA",
 "Daleville, VA", "Damascus, VA", "Dante, VA", "Danville, VA",
 "Davenport, VA", "Deerfield, VA", "Delaplane, VA", "Deltaville, VA",
 "Dendron, VA", "Dewitt, VA", "Diggs, VA", "Dinwiddie, VA",
 "Disputanta, VA", "Doe Hill, VA", "Dolphin, VA", "Doswell, VA",
 "Drakes Branch, VA", "Draper, VA", "Drewryville, VA", "Dry Fork, VA",
 "Dryden, VA", "Dublin, VA", "Dugspur, VA", "Dumfries, VA", "Dundas, VA",
 "Dungannon, VA", "Dunn Loring, VA", "Dutton, VA", "Eagle Rock, VA",
 "Earlsville, VA", "Ebony, VA", "Edinburg, VA", "Eggleston, VA",
 "Elberon, VA", "Elk Creek, VA", "Elkton, VA", "Elkwood, VA",
 "Emporia, VA", "Engleside, VA", "Esmont, VA", "Etlan, VA",
 "Ettrick, VA", "Evington, VA", "Ewing, VA", "Exeter, VA", "Exmore, VA",
 "Faber, VA", "Fairfax, VA", "Fairfax Station, VA", "Fairfield, VA",
 "Fairlawn, VA", "Falls Church, VA", "Falls Mills, VA", "Falmouth, VA",
 "Fancy Gap, VA", "Farmville, VA", "Farnham, VA", "Fentress, VA",
 "Ferrum, VA", "Fieldale, VA", "Fincastle, VA", "Fleet, VA",
 "Flint Hill, VA", "Forest, VA", "Fork Union, VA", "Fort Belvoir, VA",
 "Fort Blackmore, VA", "Fort Defiance, VA", "Fort Lee, VA",
 "Foster Falls, VA", "Franconia, VA", "Franklin, VA", "Franktown, VA",
 "Fredericksburg, VA", "Freeman, VA", "Fries, VA", "Front Royal, VA",
 "Fulks Run, VA", "Gainesville, VA", "Galax, VA", "Gasburg, VA",
 "Gate City, VA", "Geer, VA", "Glade Spring, VA", "Gladehill, VA",
 "Gladstone, VA", "Gladys, VA", "Glasgow, VA", "Glen Allen, VA",
 "Glen Lyn, VA", "Gloucester Point, VA", "Goldbond, VA", "Goldvein, VA",
 "Goochland, VA", "Goode, VA", "Goodview, VA", "Gore, VA", "Goshen, VA",
 "Grafton, VA", "Great Falls, VA", "Green Bay, VA", "Greenbackville, VA",
 "Greenbush, VA", "Greenville, VA", "Greenwood, VA", "Gretna, VA",
 "Grottoes, VA", "Grundy, VA", "Gum Spring, VA", "Gwynn, VA",
 "Hague, VA", "Halifax, VA", "Hallwood, VA", "Hamilton, VA",
 "Hampton, VA", "Handsom, VA", "Hanover, VA", "Hardy, VA",
 "Hardyville, VA", "Harrisonburg, VA", "Hartfield, VA", "Hayes, VA",
 "Haymarket, VA", "Haysi, VA", "Haywood, VA", "Head Waters, VA",

"Heathsville, VA", "Henry, VA", "Herndon, VA", "Highland Springs, VA",
"Hightown, VA", "Hillsboro, VA", "Hillsville, VA", "Hiltons, VA",
"Hinton, VA", "Hollins, VA", "Hopewell, VA", "Horntown, VA",
"Horsey, VA", "Hot Springs, VA", "Howardsville, VA", "Howertons, VA",
"Huddleston, VA", "Hume, VA", "Huntly, VA", "Hurley, VA", "Hurt, VA",
"Hustle, VA", "Independence, VA", "Indian Neck, VA",
"Indian Valley, VA", "Ingram, VA", "Ironto, VA", "Irvington, VA",
"Ivanhoe, VA", "Ivor, VA", "Jamaica, VA", "James Store, VA",
"Jarratt, VA", "Java, VA", "Jefferson Manor, VA", "Jeffersonton, VA",
"Jenkins Bridge, VA", "Jetersville, VA", "Jewell Valley, VA",
"Jonesville, VA", "Keeling, VA", "Keene, VA", "Keezletown, VA",
"Kenbridge, VA", "Kents Store, VA", "Keokee, VA", "Keysville, VA",
"Kilmarnock, VA", "King And Queen C, VA", "King George, VA",
"King William, VA", "Kinsale, VA", "Lake Anne, VA", "Lakeridge, VA",
"Lakeside, VA", "Lambsburg, VA", "Lancaster, VA", "Laneview, VA",
"Lanexa, VA", "Laurel Fork, VA", "Lebanon, VA", "Lebanon Church, VA",
"Leesburg, VA", "Lennig, VA", "Leon, VA", "Lexington, VA", "Lignum, VA",
"Linden, VA", "Linville, VA", "Lithia, VA", "Little Plymouth, VA",
"Locust Dale, VA", "Locust Grove, VA", "Locust Hill, VA",
"Locustville, VA", "Long Island, VA", "Loretto, VA", "Lottsburg, VA",
"Louisa, VA", "Lovettsville, VA", "Lovingston, VA", "Lowesville, VA",
"Lowry, VA", "Lunenburg, VA", "Luray, VA", "Lynch Station, VA",
"Lynchburg, VA", "Machipongo, VA", "Madison Heights, VA",
"Manakin Sabot, VA", "Manassas, VA", "Manassas Park, VA", "Manquin, VA",
"Mappsville, VA", "Marion, VA", "Markham, VA", "Marshall, VA",
"Martinsville, VA", "Mascot, VA", "Mason Neck, VA", "Mathews, VA",
"Maurertown, VA", "Mavisdale, VA", "Mc Clure, VA", "Mc Dowell, VA",
"Mc Gaheysville, VA", "Mc Kenney, VA", "Mc Lean, VA",
"Meadows Of Dan, VA", "Meadowview, VA", "Mears, VA",
"Mechanicsville, VA", "Meherrin, VA", "Melfa, VA", "Mendota, VA",
"Merrimac, VA", "Middlebrook, VA", "Middleburg, VA", "Middletown, VA",
"Midland, VA", "Midlothian, VA", "Miles, VA", "Milford, VA",
"Millboro Spring, VA", "Mine Run, VA", "Mineral, VA",
"Mission Home, VA", "Mitchells, VA", "Mobjack, VA", "Moneta, VA",
"Monroe, VA", "Montebello, VA", "Monterey, VA", "Montevideo, VA",
"Montezuma, VA", "Montford, VA", "Montpelier, VA", "Montross, VA",
"Montvale, VA", "Moon, VA", "Morrisville, VA", "Mosby, VA",
"Moseley, VA", "Mount Crawford, VA", "Mount Sidney, VA",
"Mount Solon, VA", "Mountain Lake, VA", "Mouth Of Wilson, VA",
"Mustoe, VA", "Narrows, VA", "Natural Bridge, VA",
"Natural Bridge S, VA", "Naval Amphibious, VA", "Nelson, VA",
"New Canton, VA", "New Castle, VA", "New Church, VA", "New Kent, VA",
"New Market, VA", "New Point, VA", "Newport, VA", "Newport News, VA",
"Newsoms, VA", "Newtown, VA", "Nickelsville, VA", "Nokesville, VA",
"Nomini Grove, VA", "Nora, VA", "Norfolk, VA", "North, VA",
"North Springfiel, VA", "Norton, VA", "Oak Grove, VA", "Oak Hall, VA",
"Oakton, VA", "Oilville, VA", "Onancock, VA", "Onemo, VA", "Onley, VA",
"Orkney Springs, VA", "Paeonian Springs, VA", "Paint Bank, VA",
"Painter, VA", "Pamplin, VA", "Paris, VA", "Parksley, VA",
"Partlow, VA", "Patrick Springs, VA", "Patterson, VA", "Pearisburg, VA",
"Penhook, VA", "Pennington Gap, VA", "Petersburg, VA", "Phenix, VA",
"Pilgrims Knob, VA", "Pilot, VA", "Pimmit, VA", "Pinero, VA",
"Piney River, VA", "Pittsville, VA", "Plain View, VA", "Poquoson, VA",
"Port Republic, VA", "Port Royal, VA", "Portsmouth, VA", "Pound, VA",
"Pounding Mill, VA", "Powhatan, VA", "Pratts, VA", "Prince George, VA",

"Prospect, VA", "Providence Forge, VA", "Pulaski, VA", "Quantico, VA",
"Quinton, VA", "Raccoon Ford, VA", "Radiant, VA", "Randolph, VA",
"Raphine, VA", "Rapidan, VA", "Rappahannock Aca, VA", "Raven, VA",
"Rawlings, VA", "Red House, VA", "Red Oak, VA", "Reedville, VA",
"Regency, VA", "Reliance, VA", "Remington, VA", "Remlik, VA",
"Reston, VA", "Reva, VA", "Rhoadesville, VA", "Rice, VA",
"Rich Creek, VA", "Richardsville, VA", "Richlands, VA", "Richmond, VA",
"Ridge, VA", "Ridgeway, VA", "Rileyville, VA", "Riner, VA",
"Ringgold, VA", "Ripplemead, VA", "Rixeyville, VA", "Roanoke, VA",
"Rockbridge Baths, VA", "Rockfish, VA", "Rockville, VA",
"Rocky Gap, VA", "Rocky Mount, VA", "Rose Hill, VA", "Rosedale, VA",
"Roseland, VA", "Round Hill, VA", "Rowe, VA", "Rural Retreat, VA",
"Rustburg, VA", "Ruther Glen, VA", "Saint Charles, VA",
"Saint Davids Chu, VA", "Saint Paul, VA", "Salem, VA", "Saltville, VA",
"Saluda, VA", "Sandston, VA", "Sandy Hook, VA", "Sandy Level, VA",
"Sanford, VA", "Saxe, VA", "Schuyler, VA", "Scottsburg, VA",
"Scottsville, VA", "Seaford, VA", "Sedley, VA", "Seven Corners, VA",
"Seven Mile Ford, VA", "Shadow, VA", "Shanghai, VA", "Shawsville, VA",
"Shenandoah, VA", "Shenandoah Caver, VA", "Sherando, VA",
"Simpsons, VA", "Skippers, VA", "Skipwith, VA", "Smithfield, VA",
"Snell, VA", "Somerset, VA", "South Boston, VA", "South Hill, VA",
"Speedwell, VA", "Spencer, VA", "Sperryville, VA", "Spottswood, VA",
"Spout Spring, VA", "Spring Grove, VA", "Springfield, VA",
"Sprouses Corner, VA", "Stafford, VA", "Staffordsville, VA",
"Stanley, VA", "Stanleytown, VA", "Star Tannery, VA",
"Stephens City, VA", "Stephenson, VA", "Sterling, VA",
"Stevensburg, VA", "Stevensville, VA", "Stewartsville, VA",
"Stonega, VA", "Stony Creek, VA", "Strasburg, VA", "Stuart, VA",
"Stuarts Draft, VA", "Suffolk, VA", "Sugar Grove, VA", "Sumerduck, VA",
"Supply, VA", "Surry, VA", "Sutherland, VA", "Sutherlin, VA",
"Swoope, VA", "Swords Creek, VA", "Syria, VA", "Syringa, VA",
"Tabb, VA", "Tamworth, VA", "Tangier, VA", "Tannersville, VA",
"Tappahannock, VA", "Tazewell, VA", "Temperanceville, VA",
"Thaxton, VA", "The Plains, VA", "Timberlake, VA", "Timberville, VA",
"Tiptop, VA", "Toano, VA", "Toms Brook, VA", "Trammel, VA",
"Triangle, VA", "Triplet, VA", "Trout Dale, VA", "Troutville, VA",
"Troy, VA", "Tye River, VA", "Union Hall, VA", "Unionville, VA",
"University, VA", "Uno, VA", "Upperville, VA", "Valentines, VA",
"Vansant, VA", "Verona, VA", "Vesuvius, VA", "Victoria, VA",
"Vienna, VA", "Viewtown, VA", "Virgilina, VA", "Virginia Beach, VA",
"Wake, VA", "Wakefield, VA", "Walkerton, VA", "Wallops Island, VA",
"Walters, VA", "Warfield, VA", "Warner, VA", "Washington, VA",
"Water View, VA", "Waterford, VA", "Waverly, VA", "Waynesboro, VA",
"Weber City, VA", "Weems, VA", "Wellington, VA", "West Augusta, VA",
"West End, VA", "West Mclean, VA", "West Point, VA",
"West Springfield, VA", "Weyers Cave, VA", "Whitacre, VA",
"White Plains, VA", "White Post, VA", "Whitethorne, VA", "Whitetop, VA",
"Whitewood, VA", "Wicomico Church, VA", "Williamsburg, VA",
"Willis, VA", "Wilsons, VA", "Winchester, VA", "Windmill Point, VA",
"Windsor, VA", "Wingina, VA", "Wintergreen, VA", "Wirtz, VA",
"Wise, VA", "Woodbridge, VA", "Woodford, VA", "Woodlawn, VA",
"Woodrow Wilson, VA", "Woodrum, VA", "Woodstock, VA", "Woodville, VA",
"Woolwine, VA", "Wylliesburg, VA", "Wytheville, VA", "Yale, VA",
"Yancey Mills, VA", "Yorktown, VA", "Zuni, VA", "Adamant, VT",
"Albany, VT", "Alburg, VT", "Arlington, VT", "Averill, VT",

"Bakersfield, VT", "Barnet, VT", "Barre, VT", "Barton, VT",
 "Beecher Falls, VT", "Bellows Falls, VT", "Belmont, VT",
 "Bennington, VT", "Bethel, VT", "Bolton Valley, VT", "Bondville, VT",
 "Bradford, VT", "Brandon, VT", "Brattleboro, VT", "Bread Loaf, VT",
 "Bridgewater, VT", "Bridgewater Corn, VT", "Bridport, VT",
 "Bristol, VT", "Bromley Mtn, VT", "Brookfield, VT", "Brownsville, VT",
 "Burlington, VT", "Cabot, VT", "Calais, VT", "Cambridge, VT",
 "Cambridgeport, VT", "Canaan, VT", "Castleton, VT", "Cavendish, VT",
 "Center Rutland, VT", "Charlotte, VT", "Chelsea, VT", "Chester, VT",
 "Chittenden, VT", "Colchester, VT", "Concord, VT", "Corinth, VT",
 "Coventry, VT", "Craftsbury, VT", "Craftsbury Commo, VT",
 "Cuttingsville, VT", "Danby, VT", "Danville, VT", "Derby, VT",
 "Derby Line, VT", "Dorset, VT", "East Arlington, VT", "East Barre, VT",
 "East Berkshire, VT", "East Burke, VT", "East Calais, VT",
 "East Charleston, VT", "East Corinth, VT", "East Dorset, VT",
 "East Dover, VT", "East Fairfield, VT", "East Hardwick, VT",
 "East Haven, VT", "East Montpelier, VT", "East Randolph, VT",
 "East Thetford, VT", "East Wallingford, VT", "Eden, VT",
 "Eden Mills, VT", "Enosburg Falls, VT", "Essex Junction, VT",
 "Fair Haven, VT", "Fairfax, VT", "Fairfield, VT", "Fairlee, VT",
 "Ferrisburg, VT", "Florence, VT", "Franklin, VT", "Gaysville, VT",
 "Gilman, VT", "Glover, VT", "Grafton, VT", "Grand Isle, VT",
 "Graniteville, VT", "Granville, VT", "Greensboro, VT",
 "Greensboro Bend, VT", "Groton, VT", "Guildhall, VT", "Hancock, VT",
 "Hardwick, VT", "Hartland, VT", "Highgate Center, VT", "Hinesburg, VT",
 "Hubbardton, VT", "Huntington, VT", "Hyde Park, VT", "Irasburg, VT",
 "Island Pond, VT", "Isle La Motte, VT", "Jacksonville, VT",
 "Jamaica, VT", "Jay Peak, VT", "Jericho Center, VT", "Johnson, VT",
 "Killington, VT", "Leicester Juncti, VT", "Lowell, VT", "Ludlow, VT",
 "Lunenburg, VT", "Lyndon Center, VT", "Lyndonville, VT",
 "Manchester Cente, VT", "Marshfield, VT", "Middletown Sprin, VT",
 "Milton, VT", "Montgomery Cente, VT", "Montpelier, VT", "Moretown, VT",
 "Morgan Ctr, VT", "Morrisville, VT", "Mount Holly, VT",
 "Mount Snow, VT", "New Haven, VT", "Newbury, VT", "Newfane, VT",
 "Newport, VT", "Newport Center, VT", "North Bennington, VT",
 "North Clarendon, VT", "North Concord, VT", "North Ferrisburg, VT",
 "North Hartland, VT", "North Hero, VT", "North Montpelier, VT",
 "North Pomfret, VT", "North Pownal, VT", "North Springfiel, VT",
 "Norton, VT", "Norwich, VT", "Orleans, VT", "Orwell, VT", "Pawlet, VT",
 "Peacham, VT", "Perkinsville, VT", "Peru, VT", "Pittsfield, VT",
 "Pittsford, VT", "Plainfield, VT", "Plymouth, VT", "Post Mills, VT",
 "Poultney, VT", "Pownal, VT", "Proctor, VT", "Proctorsville, VT",
 "Putney, VT", "Randolph, VT", "Randolph Center, VT", "Reading, VT",
 "Readsboro, VT", "Richford, VT", "Ripton, VT", "Riverton, VT",
 "Rochester, VT", "Roxbury, VT", "Rutland, VT", "Ryegate, VT",
 "Saint Albans, VT", "Saint Johnsbury, VT", "Salisbury, VT",
 "Saxtons River, VT", "Shaftsbury, VT", "Sharon, VT", "Sheffield, VT",
 "Shelburne, VT", "Sheldon, VT", "Shoreham, VT", "Smugglers Notch, VT",
 "South Burlington, VT", "South Hero, VT", "South Londonderr, VT",
 "South Pomfret, VT", "South Royalton, VT", "South Ryegate, VT",
 "South Strafford, VT", "South Woodstock, VT", "Springfield, VT",
 "Stamford, VT", "Starksboro, VT", "Stockbridge, VT", "Stowe, VT",
 "Strafford, VT", "Sugarbush Valley, VT", "Sutton, VT", "Swanton, VT",
 "Taftsville, VT", "Thetford Center, VT", "Townshend, VT", "Troy, VT",
 "Tunbridge, VT", "Underhill, VT", "Univ Of Vermont, VT",

"Vergennes, VT", "Vernon, VT", "Vershire, VT", "Waitsfield, VT",
 "Wallingford, VT", "Wardsboro, VT", "Washgtin, VT", "Waterbury, VT",
 "Waterbury Center, VT", "Waterville, VT", "Wells, VT",
 "Wells River, VT", "West Burke, VT", "West Charleston, VT",
 "West Danville, VT", "West Fairlee, VT", "West Glover, VT",
 "West Halifax, VT", "West Hartford, VT", "West Pawlet, VT",
 "West Rupert, VT", "West Rutland, VT", "West Topsham, VT",
 "West Townshend, VT", "West Wardsboro, VT", "Westfield, VT",
 "Westford, VT", "Weston, VT", "White River Junc, VT", "Whitingham, VT",
 "Williamstown, VT", "Williamsville, VT", "Williston, VT",
 "Wilmington, VT", "Windsor, VT", "Winooski, VT", "Wolcott, VT",
 "Woodbury, VT", "Woodstock, VT", "Worcester, VT", "Aberdeen, WA",
 "Acme, WA", "Addy, WA", "Algona, WA", "Allyn, WA", "Almira, WA",
 "Amanda Park, WA", "Amboy, WA", "Anacortes, WA", "Anatone, WA",
 "Anderson Island, WA", "Appleton, WA", "Ariel, WA", "Arlington, WA",
 "Ashford, WA", "Asotin, WA", "Auburn, WA", "Bainbridge Islan, WA",
 "Baring, WA", "Battle Ground, WA", "Bear Creek, WA", "Beaux Arts, WA",
 "Beaver, WA", "Bellevue, WA", "Bellingham, WA", "Benge, WA",
 "Benton City, WA", "Beverly, WA", "Bickleton, WA", "Black Diamond, WA",
 "Blaine, WA", "Bonney Lake, WA", "Bothell, WA", "Bow, WA", "Boyd, WA",
 "Bremerton, WA", "Brewster, WA", "Bridgeport, WA", "Brier, WA",
 "Brinon, WA", "Brush Prairie, WA", "Buckley, WA", "Burien, WA",
 "Burlington, WA", "Camas, WA", "Carbonado, WA", "Carlton, WA",
 "Carnation, WA", "Carson, WA", "Cascade Park, WA", "Cashmere, WA",
 "Castle Rock, WA", "Cathlamet, WA", "Centerville, WA", "Centralia, WA",
 "Chattaroy, WA", "Chehalis, WA", "Chelan, WA", "Cheney, WA",
 "Chewelah, WA", "Chimacum, WA", "Cinebar, WA", "Clallam Bay, WA",
 "Clarkston, WA", "Clayton, WA", "Cle Elum, WA", "Clinton, WA",
 "Colbert, WA", "Colfax, WA", "College Place, WA", "Colton, WA",
 "Colville, WA", "Concrete, WA", "Connell, WA", "Cook, WA",
 "Copalis Beach, WA", "Copalis Crossing, WA", "Cosmopolis, WA",
 "Cougar, WA", "Coulee City, WA", "Coulee Dam, WA", "Coupeville, WA",
 "Coville, WA", "Creston, WA", "Cunningham, WA", "Curlew, WA",
 "Curtis, WA", "Cusick, WA", "Custer, WA", "Danville, WA",
 "Darrington, WA", "Davenport, WA", "Dayton, WA", "Deer Park, WA",
 "Des Moines, WA", "Duvall, WA", "East Wenatchee, WA", "Eastsound, WA",
 "Eatonville, WA", "Edmonds, WA", "Edwall, WA", "Elbe, WA",
 "Electric City, WA", "Elk, WA", "Ellensburg, WA", "Elma, WA",
 "Eltopia, WA", "Endicott, WA", "Entiat, WA", "Enumclaw, WA",
 "Ephrata, WA", "Espanola, WA", "Ethel, WA", "Evans, WA", "Everett, WA",
 "Everson, WA", "Fairchild Air Fo, WA", "Fairfield, WA", "Fall City, WA",
 "Farmington, WA", "Federal Way, WA", "Felida, WA", "Ferndale, WA",
 "Fife, WA", "Fircrest, WA", "Ford, WA", "Forks, WA", "Fort Lewis, WA",
 "Fox Island, WA", "Freeland, WA", "Friday Harbor, WA", "Fruitland, WA",
 "Garfield, WA", "Gifford, WA", "Gig Harbor, WA", "Glacier, WA",
 "Glenoma, WA", "Glenwood, WA", "Goldendale, WA", "Graham, WA",
 "Grand Coulee, WA", "Grandview, WA", "Granger, WA", "Granite Falls, WA",
 "Grapeview, WA", "Grayland, WA", "Grays River, WA", "Greenacres, WA",
 "Greenbank, WA", "Hansville, WA", "Harrah, WA", "Harrington, WA",
 "Hartline, WA", "Hazel Dell, WA", "Home, WA", "Hoodsport, WA",
 "Hoquiam, WA", "Humptulips, WA", "Hunters, WA", "Ilwaco, WA",
 "Inchelium, WA", "Ione, WA", "Issaquah, WA", "Kalama, WA", "Keller, WA",
 "Kelso, WA", "Kennewick, WA", "Kent, WA", "Kettle Falls, WA",
 "Kingston, WA", "Kirkland, WA", "Klickitat, WA", "La Center, WA",
 "La Conner, WA", "Lacey, WA", "Lacrosse, WA", "Lake Stevens, WA",

"Lakewood Center, WA", "Lamont, WA", "Langley, WA", "Latah, WA",
 "Leavenworth, WA", "Liberty Lake, WA", "Lilliwaup, WA", "Lincoln, WA",
 "Lind, WA", "Lk Forest Park, WA", "Long Beach, WA", "Longbranch, WA",
 "Longview, WA", "Loomis, WA", "Loon Lake, WA", "Lopez, WA",
 "Lowden, WA", "Lummi Island, WA", "Lyle, WA", "Lyman, WA", "Lynden, WA",
 "Lynnwood, WA", "Mabton, WA", "Malaga, WA", "Malo, WA", "Mansfield, WA",
 "Manson, WA", "Maple Valley, WA", "Marlin, WA", "Marysville, WA",
 "Matlock, WA", "Mattawa, WA", "Mazama, WA", "Mc Cleary, WA", "Mead, WA",
 "Mercer Island, WA", "Mesa, WA", "Metaline Falls, WA", "Methow, WA",
 "Mica, WA", "Mill Creek, WA", "Milton, WA", "Mineral, WA",
 "Moclips, WA", "Monroe, WA", "Montesano, WA", "Morton, WA",
 "Moses Lake, WA", "Mossyrock, WA", "Mount Vernon, WA",
 "Mountlake Terrac, WA", "Moxee, WA", "Mukilteo, WA", "Naselle, WA",
 "Newman Lake, WA", "Newport, WA", "Nine Mile Falls, WA", "Nordland, WA",
 "Normandy Park, WA", "North Bend, WA", "Northport, WA",
 "Oak Harbor, WA", "Oakesdale, WA", "Oakville, WA", "Ocean City, WA",
 "Ocean Park, WA", "Odessa, WA", "Okanogan, WA", "Olalla, WA",
 "Olga, WA", "Olympia, WA", "Omak, WA", "Onalaska, WA", "Orchards, WA",
 "Orondo, WA", "Oroville, WA", "Orting, WA", "Otis Orchards, WA",
 "Outlook, WA", "Pacific, WA", "Pacific Beach, WA", "Packwood, WA",
 "Palisades, WA", "Palouse, WA", "Parkland, WA", "Pasco, WA",
 "Pateros, WA", "Paterson, WA", "Pe Ell, WA", "Peshastin, WA",
 "Plymouth, WA", "Point Roberts, WA", "Pomeroy, WA", "Port Angeles, WA",
 "Port Hadlock, WA", "Port Ludlow, WA", "Port Townsend, WA",
 "Poulsbo, WA", "Prescott, WA", "Prosser, WA", "Puget Sound Nava, WA",
 "Pullman, WA", "Puyallup, WA", "Quilcene, WA", "Quinault, WA",
 "Quincy, WA", "Rainier, WA", "Randle, WA", "Ravensdale, WA",
 "Raymond, WA", "Reardan, WA", "Redmond, WA", "Renton, WA",
 "Republic, WA", "Rice, WA", "Richland, WA", "Ridgefield, WA",
 "Ritzville, WA", "Riverside, WA", "Rochester, WA", "Rock Island, WA",
 "Rockford, WA", "Rockport, WA", "Roosevelt, WA", "Rosalia, WA",
 "Rosburg, WA", "Roy, WA", "Royal City, WA", "Ryderwood, WA",
 "Saint John, WA", "Salkum, WA", "Seabeck, WA", "Seatac, WA",
 "Seattle, WA", "Sedro Woolley, WA", "Sekiu, WA", "Selah, WA",
 "Sequim, WA", "Shelton, WA", "Silver Creek, WA", "Silverdale, WA",
 "Silverlake, WA", "Skamokawa, WA", "Skykomish, WA", "Snohomish, WA",
 "Snoqualmie, WA", "Soap Lake, WA", "South Bend, WA",
 "South Park Villa, WA", "Spanaway, WA", "Spangle, WA", "Spokane, WA",
 "Sprague, WA", "Springdale, WA", "Stanwood, WA", "Stehekin, WA",
 "Steilacoom, WA", "Stevenson, WA", "Sultan, WA", "Sumas, WA",
 "Sunnyside, WA", "Suquamish, WA", "Tacoma, WA", "Taholah, WA",
 "Tahuya, WA", "Tekoa, WA", "Tenino, WA", "Terrace Heights, WA",
 "Thornton, WA", "Thorp, WA", "Tieton, WA", "Tokeland, WA", "Toledo, WA",
 "Tonasket, WA", "Toppenish, WA", "Toutle, WA", "Trout Lake, WA",
 "Tukwila, WA", "Tumtum, WA", "Twisp, WA", "Underwood, WA", "Union, WA",
 "Union Gap, WA", "Uniontown, WA", "Usk, WA", "Vader, WA", "Valley, WA",
 "Valleyford, WA", "Vancouver, WA", "Vashon, WA", "Vaughn, WA",
 "Veradale, WA", "Wahkiacus, WA", "Waitsburg, WA", "Walla Walla, WA",
 "Wapato, WA", "Warden, WA", "Washougal, WA", "Washtucna, WA",
 "Waterville, WA", "Wauconda, WA", "Waverly, WA", "Wellpinit, WA",
 "Wenatchee, WA", "Westport, WA", "Whidbey Island N, WA",
 "White Pass, WA", "White Salmon, WA", "White Swan, WA",
 "Wide Hollow, WA", "Wilbur, WA", "Winlock, WA", "Winthrop, WA",
 "Woodinville, WA", "Woodland, WA", "Woodway, WA", "Yacolt, WA",
 "Yakima, WA", "Yelm, WA", "Zillah, WA", "Abbotsford, WI", "Abrams, WI",

"Adams, WI", "Adell, WI", "Albany, WI", "Algoma, WI", "Allenton, WI",
 "Allouez, WI", "Alma, WI", "Alma Center, WI", "Almena, WI",
 "Almond, WI", "Altoona, WI", "Alvin, WI", "Amberg, WI", "Amherst, WI",
 "Amherst Junction, WI", "Aniwa, WI", "Antigo, WI", "Appleton, WI",
 "Arcadia, WI", "Arena, WI", "Argyle, WI", "Arkansaw, WI", "Arkdale, WI",
 "Arlington, WI", "Armstrong Creek, WI", "Arpin, WI", "Ashwaubenon, WI",
 "Athelstane, WI", "Auburndale, WI", "Augusta, WI", "Avalon, WI",
 "Avoca, WI", "Babcock, WI", "Bagley, WI", "Baileys Harbor, WI",
 "Baldwin, WI", "Balsam Lake, WI", "Bancroft, WI", "Bangor, WI",
 "Baraboo, WI", "Barnes, WI", "Barneveld, WI", "Barron, WI",
 "Barronett, WI", "Bay City, WI", "Bay View, WI", "Bayfield, WI",
 "Bear Creek, WI", "Beaver, WI", "Beaver Dam, WI", "Beldenville, WI",
 "Belgium, WI", "Belleville, WI", "Belmont, WI", "Benton, WI",
 "Berlin, WI", "Big Bend, WI", "Birchwood, WI", "Birnamwood, WI",
 "Black Earth, WI", "Black River Fall, WI", "Blair, WI",
 "Blanchardville, WI", "Bloom City, WI", "Bloomer, WI",
 "Bloomington, WI", "Blue Mounds, WI", "Blue River, WI", "Boscobel, WI",
 "Boulder Junction, WI", "Bowler, WI", "Boyceville, WI", "Boyd, WI",
 "Brandon, WI", "Brantwood, WI", "Briggsville, WI", "Brillion, WI",
 "Bristol, WI", "Brodhead, WI", "Brookfield, WI", "Brooklyn, WI",
 "Browntown, WI", "Bruce, WI", "Brule, WI", "Brussels, WI", "Bryant, WI",
 "Burlington, WI", "Burnett, WI", "Butler, WI", "Butternut, WI",
 "Cable, WI", "Cadott, WI", "Caledonia, WI", "Cambria, WI",
 "Cambridge, WI", "Cameron, WI", "Campbellsport, WI", "Canton, WI",
 "Caroline, WI", "Cascade, WI", "Casco, WI", "Cashton, WI",
 "Cassville, WI", "Catawba, WI", "Cato, WI", "Cavour, WI",
 "Cazenovia, WI", "Cecil, WI", "Cedar Grove, WI", "Cedarburg, WI",
 "Center Valley, WI", "Centuria, WI", "Chaseburg, WI", "Chelsea, WI",
 "Chetek, WI", "Chili, WI", "Chilton, WI", "Chippewa Falls, WI",
 "Clam Falls, WI", "Clam Lake, WI", "Clayton, WI", "Clear Lake, WI",
 "Cleveland, WI", "Clinton, WI", "Clintonville, WI", "Cobb, WI",
 "Colby, WI", "Coleman, WI", "Colfax, WI", "Colgate, WI", "Coloma, WI",
 "Columbus, WI", "Combined Locks, WI", "Comstock, WI", "Conover, WI",
 "Conrath, WI", "Coon Valley, WI", "Cornell, WI", "Cornucopia, WI",
 "Cottage Grove, WI", "Crandon, WI", "Cross Plains, WI", "Cuba City, WI",
 "Cudahy, WI", "Cumberland, WI", "Curtiss, WI", "Cushing, WI",
 "Custer, WI", "Cutler, WI", "Dairyland, WI", "Dallas, WI", "Dalton, WI",
 "Dane, WI", "Darlen, WI", "Darlington, WI", "De Forest, WI",
 "De Pere, WI", "Deer Park, WI", "Deerbrook, WI", "Deerfield, WI",
 "Delafield, WI", "Delavan, WI", "Delta, WI", "Denmark, WI",
 "Deronda, WI", "Dodge, WI", "Dodgeville, WI", "Dorchester, WI",
 "Dousman, WI", "Downing, WI", "Dresser, WI", "Drummond, WI",
 "Dunbar, WI", "Durand, WI", "Eagle, WI", "Eagle River, WI", "Earl, WI",
 "East Troy, WI", "Eastman, WI", "Eau Claire, WI", "Eau Galle, WI",
 "Eden, WI", "Edgerton, WI", "Egg Harbor, WI", "Eland, WI", "Elcho, WI",
 "Eldorado, WI", "Eleva, WI", "Elk Mound, WI", "Elkhart Lake, WI",
 "Elkhorn, WI", "Ellison Bay, WI", "Ellsworth, WI", "Elm Grove, WI",
 "Elmwood, WI", "Elroy, WI", "Elton, WI", "Emerald, WI", "Endeavor, WI",
 "Ettrick, WI", "Evansville, WI", "Evergreen, WI", "Exeland, WI",
 "Fairchild, WI", "Fall Creek, WI", "Fall River, WI", "Fence, WI",
 "Fennimore, WI", "Fenwood, WI", "Ferryville, WI", "Fifield, WI",
 "Fish Creek, WI", "Fitchburg, WI", "Florence, WI", "Fontana, WI",
 "Forest Junction, WI", "Forestville, WI", "Fort Atkinson, WI",
 "Fountain City, WI", "Fox Lake, WI", "Foxboro, WI", "Francis Creek, WI",
 "Franklin, WI", "Franksville, WI", "Fremont, WI", "Friendship, WI",

"Galesville, WI", "Gays Mills, WI", "Genoa, WI", "Genoa City, WI",
 "Germantown, WI", "Gillett, WI", "Gillingham, WI", "Gilman, WI",
 "Gleason, WI", "Glen Haven, WI", "Glenbeulah, WI", "Glenwood City, WI",
 "Glidden, WI", "Goodman, WI", "Gordon, WI", "Grafton, WI",
 "Grand Marsh, WI", "Grand View, WI", "Granton, WI", "Gratiot, WI",
 "Green Bay, WI", "Green Lake, WI", "Greendale, WI", "Greenfield, WI",
 "Greenleaf, WI", "Greenville, WI", "Greenwood, WI", "Gresham, WI",
 "Hager City, WI", "Hales Corners, WI", "Hamburg, WI", "Hammond, WI",
 "Hancock, WI", "Harshaw, WI", "Hartford, WI", "Hartland, WI",
 "Hatley, WI", "Hawkins, WI", "Hazel Green, WI", "Hazelhurst, WI",
 "Helenville, WI", "Herbster, WI", "Hertel, WI", "Hewitt, WI",
 "High Bridge, WI", "Highland, WI", "Hilbert, WI", "Hillpoint, WI",
 "Hillsdale, WI", "Holcombe, WI", "Hollandale, WI", "Holmen, WI",
 "Horicon, WI", "Hortonville, WI", "Howard, WI", "Howards Grove, WI",
 "Hubertus, WI", "Hudson, WI", "Humbird, WI", "Hurley, WI",
 "Independence, WI", "Ingram, WI", "Iola, WI", "Irma, WI",
 "Iron Belt, WI", "Iron Ridge, WI", "Iron River, WI", "Ixonia, WI",
 "Jackson, WI", "Janesville, WI", "Jefferson, WI", "Jim Falls, WI",
 "Johnson Creek, WI", "Juda, WI", "Junction City, WI", "Juneau, WI",
 "Kansasville, WI", "Kaukauna, WI", "Kellnersville, WI", "Kendall, WI",
 "Kennan, WI", "Kenosha, WI", "Keshena, WI", "Kewaskum, WI",
 "Kewaunee, WI", "Kiel, WI", "Kimberly, WI", "King, WI", "Kingston, WI",
 "Knapp, WI", "Knowles, WI", "Kohler, WI", "Krakow, WI", "La Crosse, WI",
 "La Pointe, WI", "La Valle, WI", "Lac Du Flambeau, WI", "Ladysmith, WI",
 "Lake Geneva, WI", "Lake Mills, WI", "Lake Nebagamon, WI",
 "Lake Tomahawk, WI", "Lakewood, WI", "Lancaster, WI",
 "Land O Lakes, WI", "Lannon, WI", "Laona, WI", "Larsen, WI",
 "Leopolis, WI", "Lily, WI", "Linden, WI", "Little Chute, WI",
 "Little Suamico, WI", "Livingston, WI", "Lodi, WI", "Loganville, WI",
 "Lone Rock, WI", "Loretta, WI", "Lowell, WI", "Loyal, WI", "Lublin, WI",
 "Luck, WI", "Luxemburg, WI", "Lyndon Station, WI", "Madison, WI",
 "Maiden Rock, WI", "Malone, WI", "Manawa, WI", "Manitowish Water, WI",
 "Manitowoc, WI", "Maple, WI", "Marathon, WI", "Marengo, WI",
 "Maribel, WI", "Marinette, WI", "Marion, WI", "Markesan, WI",
 "Marquette, WI", "Marshall, WI", "Marshfield, WI", "Mather, WI",
 "Mauston, WI", "Mayville, WI", "Mazomanie, WI", "Mc Farland, WI",
 "Medford, WI", "Mellen, WI", "Melrose, WI", "Menasha, WI",
 "Menomonee Falls, WI", "Menomonie, WI", "Mequon, WI", "Mercer, WI",
 "Merrill, WI", "Merrillan, WI", "Merrimac, WI", "Middleton, WI",
 "Milladore, WI", "Milltown, WI", "Milton, WI", "Milwaukee, WI",
 "Mindoro, WI", "Mineral Point, WI", "Minocqua, WI", "Minong, WI",
 "Mishicot, WI", "Modena, WI", "Monico, WI", "Monona, WI", "Monroe, WI",
 "Montello, WI", "Montfort, WI", "Monticello, WI", "Moquah, WI",
 "Mosinee, WI", "Mount Calvary, WI", "Mount Hope, WI", "Mount Horeb, WI",
 "Mountain, WI", "Mukwonago, WI", "Muscoda, WI", "Muskego, WI",
 "Nashotah, WI", "Navarino, WI", "Necedah, WI", "Neenah, WI",
 "Neillsville, WI", "Nekoosa, WI", "Nelson, WI", "Neopit, WI",
 "Neosho, WI", "Neshkoro, WI", "New Auburn, WI", "New Berlin, WI",
 "New Franken, WI", "New Glarus, WI", "New Holstein, WI",
 "New Lisbon, WI", "New London, WI", "New Post, WI", "New Richmond, WI",
 "Newton, WI", "Niagara, WI", "North Fond Du La, WI",
 "North Freedom, WI", "North Prairie, WI", "North Woods Beac, WI",
 "Northfield, WI", "Norwalk, WI", "Oak Creek, WI", "Oakfield, WI",
 "Oconomowoc, WI", "Oconto, WI", "Oconto Falls, WI", "Ogdensburg, WI",
 "Ogema, WI", "Ojibwa, WI", "Okauchee, WI", "Omro, WI", "Onalaska, WI",

"Oneida, WI", "Ontario, WI", "Oostburg, WI", "Oregon, WI",
 "Orfordville, WI", "Osceola, WI", "Oshkosh, WI", "Osseo, WI",
 "Owen, WI", "Oxford, WI", "Palmyra, WI", "Pardeeville, WI",
 "Park Falls, WI", "Pearson, WI", "Pelican Lake, WI", "Pembine, WI",
 "Pence, WI", "Pepin, WI", "Peshtigo, WI", "Pewaukee, WI", "Phelps, WI",
 "Phillips, WI", "Pickerel, WI", "Pickett, WI", "Pine River, WI",
 "Pittsville, WI", "Plain, WI", "Plainfield, WI", "Platteville, WI",
 "Plover, WI", "Plum City, WI", "Plymouth, WI", "Poplar, WI",
 "Port Edwards, WI", "Port Washington, WI", "Port Wing, WI",
 "Portage, WI", "Porterfield, WI", "Potosi, WI", "Pound, WI",
 "Poy Sippi, WI", "Poynette, WI", "Prairie Du Chien, WI",
 "Prairie Du Sac, WI", "Prairie Farm, WI", "Prentice, WI",
 "Prescott, WI", "Princeton, WI", "Pulaski, WI", "Racine, WI",
 "Radisson, WI", "Randolph, WI", "Random Lake, WI", "Readstown, WI",
 "Redgranite, WI", "Reedsburg, WI", "Reedsville, WI", "Reeseville, WI",
 "Rewey, WI", "Rib Lake, WI", "Richfield, WI", "Ridgeland, WI",
 "Ridgeway, WI", "Ringle, WI", "Rio, WI", "Ripon, WI", "River Falls, WI",
 "Roberts, WI", "Rock Springs, WI", "Rockland, WI", "Rosendale, WI",
 "Rosholt, WI", "Rothschild, WI", "Rubicon, WI", "Rudolph, WI",
 "Saint Cloud, WI", "Saint Croix Fall, WI", "Saint Germain, WI",
 "Saint Joseph, WI", "Saint Nazianz, WI", "Salem, WI", "Sand Creek, WI",
 "Saron, WI", "Sauk City, WI", "Saukville, WI", "Saxon, WI",
 "Sayner, WI", "Scandinavia, WI", "Schofield, WI", "Seymour, WI",
 "Sharon, WI", "Shawano, WI", "Sheboygan, WI", "Sheboygan Falls, WI",
 "Sheldon, WI", "Shell Lake, WI", "Shiocton, WI", "Shopiere, WI",
 "Shorewood, WI", "Shullsburg, WI", "Siren, WI", "Sister Bay, WI",
 "Slinger, WI", "Sobieski, WI", "Soldiers Grove, WI", "Somerset, WI",
 "South Byron, WI", "South Milwaukee, WI", "South Wayne, WI",
 "Sparta, WI", "Spencer, WI", "Spooner, WI", "Spring Green, WI",
 "Spring Valley, WI", "Stanley, WI", "Star Prairie, WI",
 "Stetsonville, WI", "Steuben, WI", "Stevens Point, WI", "Stiles, WI",
 "Stitzer, WI", "Stockholm, WI", "Stoddard, WI", "Stone Lake, WI",
 "Stoughton, WI", "Stratford, WI", "Strum, WI", "Sturgeon Bay, WI",
 "Sturtevant, WI", "Sullivan, WI", "Summit Lake, WI", "Sun Prairie, WI",
 "Superior, WI", "Suring, WI", "Sussex, WI", "Taycheedah, WI",
 "Taylor, WI", "Theresa, WI", "Thorp, WI", "Three Lakes, WI",
 "Tigerton, WI", "Tilleda, WI", "Tomahawk, WI", "Tony, WI",
 "Townsend, WI", "Trego, WI", "Trempealeau, WI", "Trevor, WI",
 "Tripoli, WI", "Turtle Lake, WI", "Twin Lakes, WI", "Two Rivers, WI",
 "Underhill, WI", "Union Grove, WI", "Unity, WI", "Upson, WI",
 "Valders, WI", "Van Dyne, WI", "Verona, WI", "Vesper, WI",
 "Victory, WI", "Viola, WI", "Viroqua, WI", "Wabeno, WI", "Waldo, WI",
 "Wales, WI", "Walworth, WI", "Warrens, WI", "Washburn, WI",
 "Washington Islan, WI", "Waterloo, WI", "Watertown, WI", "Waubeka, WI",
 "Waukesha, WI", "Waumandee, WI", "Waunakee, WI", "Waupaca, WI",
 "Waupun, WI", "Wausau, WI", "Wausaukee, WI", "Wautoma, WI",
 "Wauwatosa, WI", "Wauzeka, WI", "Webster, WI", "Wentworth, WI",
 "West Allis, WI", "West Bend, WI", "West Lima, WI",
 "West Milwaukee, WI", "West Salem, WI", "Westboro, WI", "Westby, WI",
 "Westfield, WI", "Weyauwega, WI", "Weyerhaeuser, WI", "Wheeler, WI",
 "White Lake, WI", "Whitehall, WI", "Whitelaw, WI", "Whitewater, WI",
 "Wild Rose, WI", "Willard, WI", "Williams Bay, WI", "Wilson, WI",
 "Wilton, WI", "Winchester, WI", "Wind Lake, WI", "Windsor, WI",
 "Winneconne, WI", "Wisconsin Dells, WI", "Wisconsin Rapids, WI",
 "Withee, WI", "Wittenberg, WI", "Wonewoc, WI", "Woodman, WI",

"Woodruff, WI", "Woodville, WI", "Wrightstown, WI", "Wyeville, WI",
 "Yuba, WI", "Abraham, WV", "Adrian, WV", "Advent, WV", "Albright, WV",
 "Alexander, WV", "Algoma, WV", "Alkol, WV", "Alum Bridge, WV",
 "Alum Creek, WV", "Alvy, WV", "Amboy, WV", "Ameagle, WV", "Amigo, WV",
 "Amma, WV", "Ansted, WV", "Anthony, WV", "Apple Grove, WV",
 "Arbovale, WV", "Arbuckle, WV", "Arnett, WV", "Arnoldsburg, WV",
 "Arthur, WV", "Artie, WV", "Asbury, WV", "Asco, WV", "Ashford, WV",
 "Ashton, WV", "Athens, WV", "Auburn, WV", "Augusta, WV", "Auto, WV",
 "Baisden, WV", "Baker, WV", "Bald Knob, WV", "Baldwin, WV",
 "Ballard, WV", "Ballengee, WV", "Bandytown, WV", "Barboursville, WV",
 "Barnabus, WV", "Barrett, WV", "Bartow, WV", "Baxter, WV", "Beaver, WV",
 "Beckley, WV", "Beeson, WV", "Belington, WV", "Belleville, WV",
 "Belva, WV", "Bens Run, WV", "Bentree, WV", "Benwood, WV", "Berea, WV",
 "Bethany, WV", "Beverly, WV", "Big Bend, WV", "Big Chimney, WV",
 "Big Creek, WV", "Big Otter, WV", "Big Run, WV", "Bim, WV",
 "Birch River, WV", "Blacksville, WV", "Blandville, WV", "Bloomery, WV",
 "Bloomingrose, WV", "Blount, WV", "Bluewell, WV", "Bob White, WV",
 "Boggs, WV", "Bolair, WV", "Bolt, WV", "Bomont, WV", "Bozoo, WV",
 "Bramwell, WV", "Branchland, WV", "Brandywine, WV", "Breedon, WV",
 "Brenton, WV", "Bridgeport, WV", "Bristol, WV", "Broaddus, WV",
 "Brohard, WV", "Bruceton Mills, WV", "Buckeye, WV", "Buffalo, WV",
 "Bunker Hill, WV", "Burlington, WV", "Burnwell, WV", "Cabin Creek, WV",
 "Cabins, WV", "Cairo, WV", "Caldwell, WV", "Calvin, WV", "Camden, WV",
 "Cameron, WV", "Camp Creek, WV", "Canvas, WV", "Capon Bridge, WV",
 "Carbon, WV", "Cascade, WV", "Cedar Grove, WV", "Center Point, WV",
 "Century, WV", "Chapmanville, WV", "Charles Town, WV", "Charleston, WV",
 "Charmco, WV", "Cherry Run, WV", "Chester, WV", "Circleville, WV",
 "Clay, WV", "Clear Creek, WV", "Clear Fork, WV", "Clem, WV",
 "Cleveland, WV", "Clifftop, WV", "Clintonville, WV", "Clio, WV",
 "Clothier, WV", "Coal Mountain, WV", "Coalton, WV", "Coburn, WV",
 "Colcord, WV", "Colliers, WV", "Comfort, WV", "Cool Ridge, WV",
 "Copen, WV", "Core, WV", "Corinth, WV", "Corley, WV", "Costa, WV",
 "Cottageville, WV", "Cove Gap, WV", "Cowen, WV", "Coxs Mills, WV",
 "Crab Orchard, WV", "Craigsville, WV", "Crawford, WV", "Crawley, WV",
 "Creston, WV", "Cross Lanes, WV", "Crum, WV", "Culloden, WV",
 "Cunard, WV", "Cyclone, WV", "Dallas, WV", "Daniels, WV",
 "Danville, WV", "Davin, WV", "Davis, WV", "Davisville, WV",
 "Dawson, WV", "Dehue, WV", "Delray, WV", "Diamond, WV", "Diana, WV",
 "Dille, WV", "Dingess, WV", "Dixie, WV", "Dorcas, WV", "Dorothy, WV",
 "Dott, WV", "Drennen, WV", "Droop, WV", "Dry Creek, WV", "Dryfork, WV",
 "Duck, WV", "Duhring, WV", "Dunbar, WV", "Duncan, WV", "Dunlow, WV",
 "Dunmore, WV", "Durbin, WV", "Earling, WV", "East Gulf, WV",
 "East Lynn, WV", "Edmond, WV", "Egdon, WV", "Elgood, WV",
 "Elizabeth, WV", "Elk Garden, WV", "Elkins, WV", "Ellamore, WV",
 "Elm Grove, WV", "Elmira, WV", "Elton, WV", "Enterprise, WV",
 "Erbacon, WV", "Evans, WV", "Fairdale, WV", "Fairview, WV",
 "Falling Rock, WV", "Falling Waters, WV", "Falls Mill, WV",
 "Fanrock, WV", "Farmington, WV", "Fenwick, WV", "Ferrellsburg, WV",
 "Fisher, WV", "Five Forks, WV", "Flat Top, WV", "Floe, WV",
 "Flower, WV", "Fola, WV", "Follansbee, WV", "Folsom, WV",
 "Fort Ashby, WV", "Fort Gay, WV", "Fort Seybert, WV", "Fort Spring, WV",
 "Foster, WV", "Frame, WV", "Franklin, WV", "Fraziers Bottom, WV",
 "Friendly, WV", "Gallipolis Ferry, WV", "Gandeeville, WV",
 "Gap Mills, WV", "Garrison, WV", "Gassaway, WV", "Gauley Bridge, WV",
 "Gauley Mills, WV", "Gay, WV", "Gem, WV", "Gerrardstown, WV",

"Ghent, WV", "Gilbert, WV", "Gilboa, WV", "Given, WV", "Glace, WV",
 "Glady, WV", "Glen, WV", "Glen Dale, WV", "Glen Daniel, WV",
 "Glen Easton, WV", "Glen Fork, WV", "Glen Rogers, WV", "Glendon, WV",
 "Glenwood, WV", "Gordon, WV", "Gormanian, WV", "Grafton, WV",
 "Grantsville, WV", "Grassy Meadows, WV", "Great Cacapon, WV",
 "Green Bank, WV", "Green Spring, WV", "Green Sulphur Sp, WV",
 "Greenville, WV", "Greenwood, WV", "Griffithsville, WV",
 "Hambleton, WV", "Hamlin, WV", "Hampden, WV", "Hancock, WV",
 "Hanover, WV", "Hansford, WV", "Harman, WV", "Harmony, WV",
 "Harpers Ferry, WV", "Harrison, WV", "Harvey, WV", "Hastings, WV",
 "Hazelgreen, WV", "Heaters, WV", "Helvetia, WV", "Henderson, WV",
 "Hendricks, WV", "Herndon, WV", "Hernshaw, WV", "Herold, WV",
 "Hewett, WV", "Hico, WV", "High View, WV", "Highland, WV", "Hinton, WV",
 "Hopemont, WV", "Horner, WV", "Horse Shoe Run, WV", "Hundred, WV",
 "Hunt, WV", "Huntington, WV", "Hurricane, WV", "Huttonsville, WV",
 "Jaeger, WV", "Independence, WV", "Indian Mills, WV", "Indore, WV",
 "Inwood, WV", "Jacksonburg, WV", "Jane Lew, WV", "Jesse, WV", "Job, WV",
 "Jodie, WV", "Jolo, WV", "Josephine, WV", "Julian, WV", "Junction, WV",
 "Kanawha Falls, WV", "Kanawha Head, WV", "Kasson, WV",
 "Kearneysville, WV", "Kegley, WV", "Kenova, WV", "Kentuck, WV",
 "Kerens, WV", "Kermit, WV", "Keslers Cross La, WV", "Kessler, WV",
 "Kieffer, WV", "Kimberly, WV", "Kincaid, WV", "Kingwood, WV",
 "Kirby, WV", "Lahmansville, WV", "Lake, WV", "Lanham, WV",
 "Lansing, WV", "Lashmeet, WV", "Lavalette, WV", "Lawton, WV",
 "Left Hand, WV", "Lehew, WV", "Leivasy, WV", "Lenore, WV", "Lerona, WV",
 "Lesage, WV", "Lester, WV", "Letart, WV", "Letter Gap, WV",
 "Levels, WV", "Lewisburg, WV", "Liberty, WV", "Lima, WV", "Linden, WV",
 "Lindside, WV", "Linn, WV", "Littleton, WV", "Lizemores, WV",
 "Lobata, WV", "Lockney, WV", "Lookout, WV", "Looneyville, WV",
 "Lost City, WV", "Lost Creek, WV", "Lost River, WV", "Lumberport, WV",
 "Maben, WV", "Mabie, WV", "Macfarlan, WV", "Madison, WV", "Mahone, WV",
 "Maidsville, WV", "Malden, WV", "Mammoth, WV", "Manheim, WV",
 "Mannington, WV", "Marfrance, WV", "Marianna, WV", "Marmet, WV",
 "Martinsburg, WV", "Mason, WV", "Matheny, WV", "Mathias, WV",
 "Maxwelton, WV", "Maysel, WV", "Maysville, WV", "Mc Dowell, WV",
 "Mc Mechen, WV", "Meador, WV", "Meadow Bluff, WV", "Meadow Bridge, WV",
 "Meadow Creek, WV", "Meadowbrook, WV", "Medley, WV", "Metz, WV",
 "Middlebourne, WV", "Midkiff, WV", "Milam, WV", "Mill Creek, WV",
 "Millstone, WV", "Millwood, WV", "Milton, WV", "Mineralwells, WV",
 "Mingo, WV", "Minnehaha Spring, WV", "Minnora, WV", "Mohawk, WV",
 "Monongah, WV", "Monterville, WV", "Montgomery, WV", "Montrose, WV",
 "Morrisvale, WV", "Moundsville, WV", "Mount Alto, WV",
 "Mount Carbon, WV", "Mount Clare, WV", "Mount Hope, WV",
 "Mount Lookout, WV", "Mount Storm, WV", "Mount Zion, WV", "Moyers, WV",
 "Mullens, WV", "Munday, WV", "Murraysville, WV", "Myra, WV",
 "Myrtle, WV", "Naoma, WV", "Napier, WV", "Nebo, WV", "Nellis, WV",
 "Neola, WV", "Nettie, WV", "New Creek, WV", "New Cumberland, WV",
 "New England, WV", "New Martinsville, WV", "New Milton, WV",
 "Newberne, WV", "Newburg, WV", "Newell, WV", "Nicut, WV", "Nimitz, WV",
 "Nitro, WV", "Nobe, WV", "Normantown, WV", "North Parkersbur, WV",
 "North Spring, WV", "Nutter Fort Ston, WV", "Oceana, WV", "Odd, WV",
 "Old Fields, WV", "Ona, WV", "Onego, WV", "Orgas, WV", "Orlando, WV",
 "Ovapa, WV", "Paden City, WV", "Palermo, WV", "Palestine, WV",
 "Parkersburg, WV", "Parsons, WV", "Patterson Creek, WV", "Paw Paw, WV",
 "Paynesville, WV", "Pecks Mill, WV", "Pence Springs, WV", "Perkins, WV",

"Peterstown, WV", "Petroleum, WV", "Peytona, WV", "Pickaway, WV",
 "Pickens, WV", "Piedmont, WV", "Pigeon, WV", "Pineville, WV",
 "Pipestem, WV", "Pliny, WV", "Poe, WV", "Point Pleasant, WV",
 "Points, WV", "Pond Gap, WV", "Pool, WV", "Powellton, WV",
 "Prichard, WV", "Proctor, WV", "Pullman, WV", "Purgitsville, WV",
 "Pursglove, WV", "Quick, WV", "Rachel, WV", "Racine, WV", "Radnor, WV",
 "Rainelle, WV", "Ramage, WV", "Ramsey, WV", "Ranger, WV", "Ranson, WV",
 "Ravencliff, WV", "Ravenswood, WV", "Reader, WV", "Red Creek, WV",
 "Red House, WV", "Reedsville, WV", "Reedy, WV", "Renick, WV",
 "Replete, WV", "Richwood, WV", "Ridgeview, WV", "Riffle, WV", "Rig, WV",
 "Rio, WV", "Ripley, WV", "Riverton, WV", "Rivesville, WV",
 "Roanoke, WV", "Robertsburg, WV", "Robinette, WV", "Robson, WV",
 "Rock Castle, WV", "Rock Cave, WV", "Rock Creek, WV", "Rockport, WV",
 "Romance, WV", "Romney, WV", "Ronceverte, WV", "Rosedale, WV",
 "Runa, WV", "Russelville, WV", "Saint Albans, WV", "Saint Marys, WV",
 "Salem, WV", "Salt Rock, WV", "Sand Ridge, WV", "Sandstone, WV",
 "Sandyville, WV", "Saulsville, WV", "Saxon, WV", "Scarbrow, WV",
 "Scherr, WV", "Scott Depot, WV", "Secondcreek, WV", "Selbyville, WV",
 "Seneca Rocks, WV", "Seth, WV", "Shanks, WV", "Shenandoah Junct, WV",
 "Shepherdstown, WV", "Sherman, WV", "Shinnston, WV", "Shock, WV",
 "Sias, WV", "Simon, WV", "Sissonville, WV", "Sistersville, WV",
 "Slab Fork, WV", "Slanesville, WV", "Slatyfork, WV", "Smithfield, WV",
 "Smithville, WV", "Smoot, WV", "Sod, WV", "South Charleston, WV",
 "Southside, WV", "Spanishburg, WV", "Spencer, WV", "Spring Dale, WV",
 "Springfield, WV", "Squire, WV", "Star City, WV", "Statts Mills, WV",
 "Stephenson, WV", "Stickney, WV", "Stony Bottom, WV",
 "Stouts Mills, WV", "Strange Creek, WV", "Streeter, WV",
 "Stumptown, WV", "Sugar Grove, WV", "Sullivan, WV", "Sumerco, WV",
 "Summersville, WV", "Summit Point, WV", "Surveyor, WV",
 "Sweet Springs, WV", "Sweetland, WV", "Swiss, WV", "Sylvester, WV",
 "Talcott, WV", "Tallmansville, WV", "Tanner, WV", "Tariff, WV",
 "Tennerton, WV", "Tesla, WV", "Thacker, WV", "Thomas, WV",
 "Thornton, WV", "Three Churches, WV", "Thurmond, WV", "Tioga, WV",
 "Toll Gate, WV", "Tornado, WV", "Triadelphia, WV", "Trout, WV",
 "Troy, WV", "True, WV", "Tunnelton, WV", "Turtle Creek, WV", "Uler, WV",
 "Union, WV", "Upper Tract, WV", "Upperglade, WV", "Valley Bend, WV",
 "Valley Fork, WV", "Valley Grove, WV", "Vallscreek, WV", "Van, WV",
 "Verner, WV", "Victor, WV", "Vienna, WV", "Volga, WV", "Wadestown, WV",
 "Waiteville, WV", "Walker, WV", "Walkersville, WV", "Wallace, WV",
 "Wallback, WV", "Walton, WV", "Wana, WV", "Wardensville, WV",
 "Waverly, WV", "Wayne, WV", "Wayside, WV", "Weirton, WV", "Welch, WV",
 "Wellsburg, WV", "Wendel, WV", "West Columbia, WV", "West Hamlin, WV",
 "West Logan, WV", "West Milford, WV", "West Union, WV", "Weston, WV",
 "Wharncliffe, WV", "White Oak, WV", "Whittaker, WV", "Wick, WV",
 "Wilbur, WV", "Wildcat, WV", "Wiley Ford, WV", "Wileyville, WV",
 "Williams Mountai, WV", "Williamson, WV", "Williamstown, WV",
 "Willow Island, WV", "Wilsie, WV", "Wilsondale, WV", "Winding Gulf, WV",
 "Winfield, WV", "Winifrede, WV", "Wolf Summit, WV", "Wolfcreek, WV",
 "Woodville, WV", "Worthington, WV", "Wymer, WV", "Yawkey, WV",
 "Acme, WY", "Afton, WY", "Aladdin, WY", "Albin, WY", "Alcova, WY",
 "Arapahoe, WY", "Arminto, WY", "Arvada, WY", "Auburn, WY", "Baggs, WY",
 "Bairoil, WY", "Banner, WY", "Basin, WY", "Bedford, WY", "Beulah, WY",
 "Bondurant, WY", "Boulder, WY", "Buffalo, WY", "Buford, WY",
 "Burlington, WY", "Burns, WY", "Carpenter, WY", "Casper, WY",
 "Centennial, WY", "Cheyenne, WY", "Chugwater, WY", "Clearmont, WY",

"Cody, WY", "Cokeville, WY", "Colter Bay, WY", "Cora, WY",
"Crowheart, WY", "Daniel, WY", "Dayton, WY", "Deaver, WY",
"Devils Tower, WY", "Dixon, WY", "Douglas, WY", "Dubois, WY",
"Encampment, WY", "Ethete, WY", "Etna, WY", "Evanston, WY",
"Evansville, WY", "Fishing Bridge, WY", "Fort Bridger, WY",
"Fort Laramie, WY", "Fort Washakie, WY", "Four Corners, WY",
"Freedom, WY", "Garrett, WY", "Gas Hills, WY", "Gillette, WY",
"Glendo, WY", "Glenrock, WY", "Grass Creek, WY", "Green River, WY",
"Greybull, WY", "Grover, WY", "Guernsey, WY", "Hanna, WY",
"Hartville, WY", "Hawk Springs, WY", "Hulett, WY", "Hyattville, WY",
"Jay Em, WY", "Jeffrey City, WY", "Jelm, WY", "Kaycee, WY",
"Keeline, WY", "Kelly, WY", "Kemmerer, WY", "Kinnear, WY",
"La Barge, WY", "Lagrange, WY", "Lance Creek, WY", "Laramie, WY",
"Lingle, WY", "Lonetree, WY", "Lost Springs, WY", "Lovell, WY",
"Lusk, WY", "Lyman, WY", "Lysite, WY", "Manderson, WY", "Marbleton, WY",
"Mc Fadden, WY", "Mc Kinnon, WY", "Medicine Bow, WY", "Meeteetse, WY",
"Meriden, WY", "Midwest, WY", "Moose, WY", "Moran, WY", "Newcastle, WY",
"Osage, WY", "Oshoto, WY", "Otto, WY", "Parkman, WY", "Pavillion, WY",
"Pine Bluffs, WY", "Pine Haven, WY", "Pinedale, WY", "Powell, WY",
"Ranchester, WY", "Rawlins, WY", "Recluse, WY", "Rock River, WY",
"Rock Springs, WY", "Rozet, WY", "Ryan Park, WY", "Savery, WY",
"Shawnee, WY", "Shell, WY", "Sheridan, WY", "Shoshoni, WY",
"Sinclair, WY", "Smoot, WY", "Story, WY", "Sundance, WY",
"Ten Sleep, WY", "Thayne, WY", "Tie Siding, WY", "Torrington, WY",
"Upton, WY", "Van Tassell, WY", "Veteran, WY", "Wamsutter, WY",
"Wapiti, WY", "Wheatland, WY", "Wilson, WY", "Worland, WY",
"Wright, WY", "Yoder, WY"

];

svg.min.js

svg.js is available for public use under the MIT license and can be found at <http://svgjs.com/>.

bootstrap.min.css

bootstrap.js is available for public use under the MIT license and can be found at <http://getbootstrap.com>.
