# Supplementary figures and images for: Estimation of individual cumulative ultraviolet exposure using a geographically-adjusted, openly-accessible tool
Source: BMC Dermatol. 2016 Jan 20;16:1. doi: 10.1186/s12895-016-0038-1 (PMC4721109; doi:10.1186/s12895-016-0038-1)

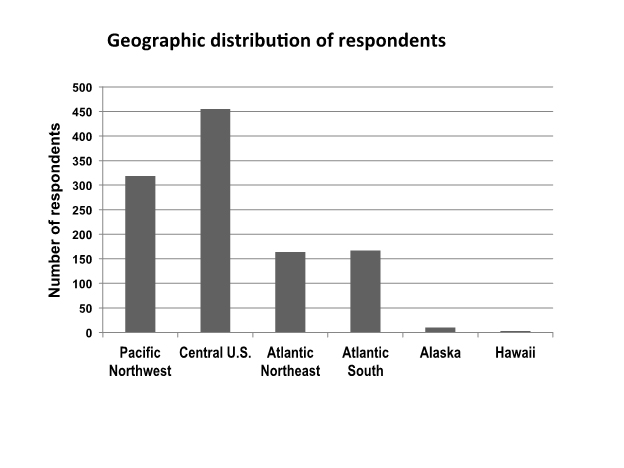

Supplement: Additional file 3: Figure S1. — The Pacific Northwest includes WA, OR and CA. Atlantic Northeast includes MA, NY, DE, RI, Washington DC, ME, CT and NH. Atlantic South includes FL, AL, LA, SC, NC, GA, VA. Central U.S. includes all other states besides those previously mentioned. (JPG 60 kb) [file 12895_2016_38_MOESM3_ESM.jpg]
