# Supplementary material for: Estimation of individual cumulative ultraviolet exposure using a geographically-adjusted, openly-accessible tool
Source: BMC Dermatol. 2016 Jan 20;16:1. doi: 10.1186/s12895-016-0038-1 (PMC4721109; doi:10.1186/s12895-016-0038-1)
Supplement: Additional file 4: Table S2. — Scoring Coefficients Used in Factor Analysis. (DOC 30 kb) [file 12895_2016_38_MOESM4_ESM.doc]

**Supplemental Table S2. Scoring Coefficients Used in Factor Analysis.**

| **Variable** | **Standardized Scoring Coefficient** |
| --- | --- |
| Shade seeking^1^ | 0.40 |
| Use of long-sleeved clothing^1^ | 0.28 |
| Use of hat^1^ | 0.23 |
| Use of sunscreen^1^ | 0.16 |
| History of blistering sunburns^2^ | -0.008 |
| Fitzpatrick skin type | -0.10 |
| Use of tanning bed^3^ | -0.11 |

^1^Responses were coded 0 – “Never,” 1 – “Rarely,” 2 – “Sometimes,” 3 – “Often,” 4 – “Always.”

^2^Responses were coded 0 – “0,” 1 – “1 – 5,” 2 – “6 – 10,” 3 – “greater than 10.”

^3^Responses were coded 0 – “0,” 1 – “1 – 5,” 2 – “6 – 10,” 3 – “11 – 100,” 4 – “greater than 100.”
